# Three‐Component 1,2‐Methylamidation of Alkynes via Coordinating Activation Strategy

**DOI:** 10.1002/open.202500151

**Published:** 2025-04-17

**Authors:** Jing Ren, Kaiyun Liu, Ning Wang, Jinlong Li, Xinyu Long, Chengming Li, Kaizhi Li

**Affiliations:** ^1^ Institute of BiopharmaceuticalsWest China Hospital Sichuan University 37 Guoxue Alley Chengdu 610041 P. R. China; ^2^ Institute of Organ Transplantation West China Hospital Sichuan University 37 Guoxue Alley Chengdu 610041 P. R. China

**Keywords:** alkynes, enamides, late‐stage functionalization, methylamidation, three‐component

## Abstract

The selective functionalization of carbon–carbon triple bonds with methyl groups remains a challenging task. Herein, the successful development of a novel copper‐catalyzed three‐component 1,2‐methylamidation of carbon–carbon triple bond is reported. The readily available coupling partners, picolinamides and alkynes with dicumyl peroxide, serve as both the methyl source and oxidant in this difunctional strategy to access methylated enamides; the substrate scope is broad, demonstrating good functional group compatibility. The synthetic utility of the reaction is also demonstrated through the 1,2‐methylamidation of alkynes via late‐stage functionalization of substrates bearing biologically relevant molecules.

## Graphical abstract

A copper‐catalyzed three‐component 1,2‐methylamidation of alkynes utilizing a novel coordinating activation strategy has been successfully developed.

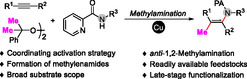

## 1 Introduction

**Figure 1:**
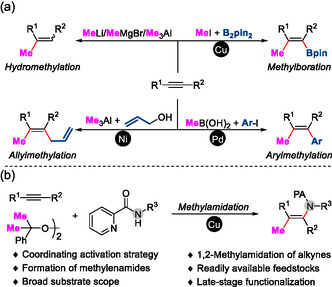
Background: methylation of alkyne motifs: a) Prior art: methylation of alkyne motifs and b) This work: methylamidation of alkyne motifs.

The widely recognized “magic methyl effect” represents the cutting‐edge in current medicinal chemistry research and has been extensively utilized to explore the pharmacology of privileged drug candidates.^[^ ^1^ ^]^ Consequently, the significance of a synthetic method for incorporating a methyl group into organic molecules has garnered considerable attention, prompting extensive efforts to develop methylation reaction methodologies over the past few decades.^[^ ^2^ ^]^ During this period, several useful methylation reagents, including methylmetal reagents, methyl halides, methylborons, peroxides, and others, have been developed.^[^ ^3^ ^]^ On the other hand, recent advancements in the 1,2‐difunctionalization reaction of alkynes have introduced diverse functional groups at specific positions on the double bonds, thereby rapidly increasing the complexity of common feedstock alkynes,^[^ ^4^ ^]^ while the efficient and selective methyl functionalization of carbon**–**carbon triple bonds remains underdeveloped. In this regard, some elaborate presentations on alkyne methylation exist, limited to hydromethylation,^[^ ^5^ ^]^ methylboration,^[^ ^6^ ^]^ allylmethylation,^[^ ^7^ ^]^ and arylmethylation^[^ ^8^ ^]^ (**Figure** **1**a). Despite these significant breakthroughs, the use of conventional methylating reagents for installing both methyl and other functional groups into alkyne molecules represents a worthwhile endeavor that is challenging and remains an unexplored research field.

Given the significance of enamides in biologically active compounds, natural products, and fine chemicals,^[^ ^9^ ^]^ a one‐step protocol for the three‐component 1,2‐methylamidation of alkynes to produce methylated enamides is highly desirable. Inspired by the coordinating activation strategy, which is an efficient and innovative tool in the field of N—H bond functionalization,^[^ ^10^ ^]^ we hypothesized that the metallacyclic species, which formed through the reaction between a metal catalyst and a substrate with a coordinating group, could efficiently capture the methylated vinyl radical generated in situ through the reaction of a methyl radical with an alkyne. Therefore, the 1,2‐methylamidation of alkynes under metal catalysis, when exposed to amines bearing a coordinating group and peroxides as the methyl source coupling partners, might provide a straightforward strategy for synthesizing methyl‐containing enamide scaffolds. Although strategically feasible, the two‐component *N*‐methylation of amides presents a significant challenge.^[^ ^11^ ^]^ We herein report a novel copper‐catalyzed three‐component 1,2‐methylamidation of alkynes using picolinamides and methylation reagent peroxides through a coordinating activation strategy, enabling the synthesis of various useful enamides with satisfactory yields (Figure 1b). Additionally, this protocol is applicable to various reactants originating from pharmaceuticals and natural compounds that possess proven biological activities, facilitating late‐stage modifications of biologically relevant motifs.

## 2 Results and Discussion

### 2.1 Optimization of the Reaction Conditions

Screening of reaction conditions was conducted by utilizing commercially available peroxides as methylation reagents,^[3a]^ thereby confirming the feasibility of the difunctional 1,2‐methylamidation reaction. The investigation began with the reaction of readily accessible *N*‐benzylpicolinamide **1a** and ethynylbenzene **2a**, with the assistance of copper catalysts and peroxides (**Table** **1**). It was found that using Cu(acac)_2_ as the catalyst with 1.5 equivalents of dicumyl peroxide (DCP) in benzene under an argon atmosphere at 120 °C for 12 h resulted in a 68% yield of the methylated enamide derivative **3a** upon isolation (Table 1, entry 3). The use of peroxides including DTBP, TBPB, and TBHP led to decreased yields (Table 1, entry 2). Comparable results were obtained with other copper catalysts (Table 1, entry 4). It was determined that other metal salts such as Ni(acac)_2_, Mn(acac)_2_, Fe(acac)_2_, and Co(acac)_2_ exhibited no reactivity toward this transformation (Table 1, entry 5). Regarding the solvent effect, benzene was determined to be the most effective solvent after evaluating several options (Table 1, entry 6). Reaction conducted under an air atmosphere resulted in a slight reduction in product yield (Table 1, entry 7). The reaction failed to produce any product without a copper catalyst (Table 1, entry 8).

**Table 1:** Optimization of the reaction conditions.

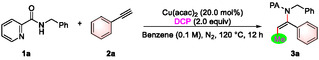		
Entrya)	Variation from standard conditions	Yield [%]b)
1	None	64
2c)	Other peroxide instead of DCP	22–41
3	1.5 equiv of DCP was used	68
4d,e)	Other copper salt instead of Cu(acac)_2_	Trace–60
5d,f)	Other metal salt instead of Cu(acac)_2_	n.d.
6d,g)	Other solvent instead of Benzene	Trace–62
7d)	Under air	59
8d)	No Cu(acac)_2_	n.d.

a) Reaction conditions: **1a** (0.10 mmol), **2a** (0.20 mmol, 2.0 eq.), DCP (0.20 mmol, 2.0 eq.), and Cu(acac)_2_ (0.01 mmol, 0.1 eq.) in benzene (0.1 M) at 120 °C for 12 h under N_2_ atmosphere.

b) Isolated yield.

c) DTBP, TBPB or TBHP was used instead of DCP.

d) 1.5 equiv of DCP was used.

e) CuBr_2_, CuCl, CuTc, CuSO_4_, Cu(OAc)_2_, Cu(hmacac)_2_, Cu(tfacac)_2_, or Cu(hfacac)_2_, was used instead of Cu(acac)_2_.

f) Ni(acac)_2_, Mn(acac)_2_, Co(acac)_2_or Fe(acac)_2_ was used instead of Cu(acac)_2_.

g) Toluene, 1,2‐DCE, PhCl, PhCF_3_, CH_3_CN, DMF, DMSO, THF, or EtOH was used instead of benzene. n.d. = no product detected.

### 2.2 Investigation of Reaction Scope

With established reaction conditions at hand, we proceeded to investigate the reaction scope of this three‐component 1,2‐methylamidation reaction using various alkynes (**Table** **2**). Generally, the reaction exhibited good compatibility across various functional groups, including halides (**3h**–**3L**), ether (**3m**–**3o** & **3v**), ester (**3p**), nitrile (**3q**), sulfamide (**3r**), amide (**3s**, **3t**), and carbamate (**3u**). To our satisfaction, 3‐thienyl‐substituted alkyne and 3‐pyridyl‐substituted alkyne exhibited smooth reactions and yielded satisfactory products (**3w**, **3x**). Unfortunately, when internal alkyne and alkyl‐substituted terminal alkyne were employed, no desired products were detected (**3y**, **3z**). The structure of compound **3g** was unequivocally identified through single‐crystal X‐Ray diffraction.^[^ ^12^ ^]^

**Table 2:** Scope of alkynes and picolinamides.

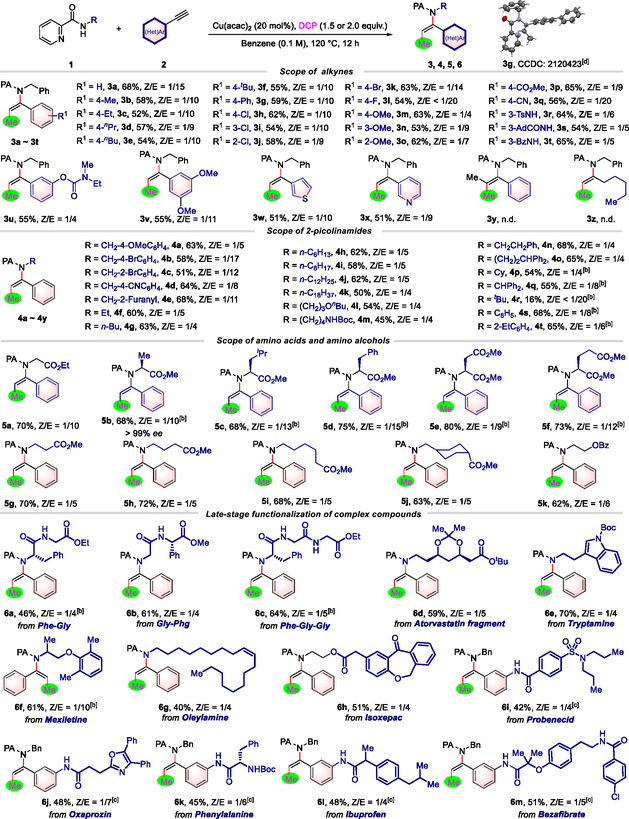

a) Reaction conditions: **1** (0.10 mmol), **2** (0.20 mmol), DCP (0.15 mmol), and Cu(acac)_2_ (0.02 mmol) in benzene (0.1 M) at 120 °C for 12 h under N_2_ atmosphere. All yields are isolated yields. The ratio of Z/E was determined by ^1^H NMR.

b) DCP (0.20 mmol) was used.

c) **2** (0.15 mmol) was used.

d) With thermal ellipsoids at 50% probability. The original structure of **3 g** is a dimer, and for clarity, only the monomer is shown here.

Next, we focused on exploring the scope of various picolinamide derivatives, with the findings also summarized in Table 2. We observed that a broad spectrum of picolinamides was compatible under this methylamidation reaction. Picolinamides bearing (hetero)benzyl groups (**4a**–**4e**), aryl groups (**4s**, **4t**), and tethered alkyl‐substituted amines (**4f**–**4r**) exhibited good compatibility and resulted in satisfactory products. Notably, picolinamides substituted with sterically hindered cyclohexyl and benzhydryl groups also reacted, producing **4p** and **4q** with yields of 54% and 55%, respectively. The reaction with

*N*‐(tert‐butyl)picolinamide was successful, albeit resulting in a low yield of **4r**. To our satisfaction, the reactions involving a broad range of natural and unnatural amino acids proceeded smoothly, resulting in the successful formation of the target compounds **5a**–**5j**.

To highlight the utility of this protocol in complex architecture editing, late‐stage modification of several substrate‐containing bioactive molecules was carried out. We are pleased to report that the current methylamidation reaction has been validated as a robust method. Therefore, the reaction of complex molecules anchored with picolinamides, such as peptides (dipeptide Phe‐Gly and Gly‐Phg, tripeptide Phe‐Gly‐Gly), natural compounds (tryptamine and oleylamine), and marketed drugs (atorvastatin, mexiletine, and isoxepac), with alkyne and peroxide yielded biologically important methylated enamides **6a**–**6h** in 40–70% yields (Table 2). Similarly, the reaction of pharmacophore‐coupled alkynes with N‐benzylpicolinamide and peroxide was found to be smooth, delivering methylated trisubstituted enamides containing amino acids (phenylalanine) and marketed drugs (probenecid, oxaprozin, ibuprofen, and bezafibrate) in acceptable yields (**6i**–**6m**, Table 2).

**Figure 2:**
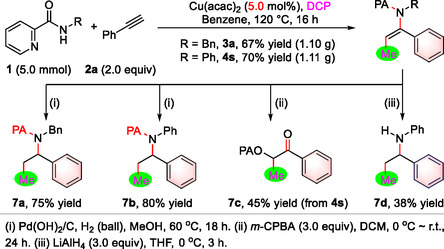
Scale‐up reactions and synthetic application.

Significantly, the protocol was successfully executed on a ≈1.1 g scale with a decreased catalyst loading, yet maintaining the yield integrity (**Figure** **2**). Subsequently, the synthetic flexibilities of the methylated enamides were investigated (Figure 2). The enamides were successfully reduced by hydrogen gas with the assistance of Pd(OH)_2_/C, resulting in benzylamines **7a** and **7b** in yields of 75% and 80%, respectively. Upon treatment of enamide **4s** with *m*‐chloroperoxybenzoic acid, α‐acyloxyketone **7c** was obtained with a yield of 45%. Importantly, the 2‐pyridylacyl auxiliary group of compound **4s** was efficiently removed under mild reaction conditions using LiAlH_4_, resulting in the desired product **7d**.

### 2.3 Mechanistic Study

**Figure 3:**
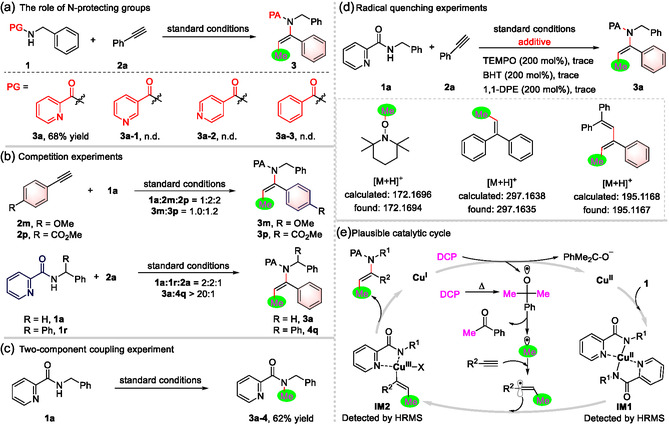
Mechanistic studies. a) The role of N‐protecting groups. b) Competition experiments. c) Two‐component coupling experiment. d) Radical quenching experiments. e) Plausible catalytic cycle.

To elucidate the mechanism, a series of controlled experiments were conducted (**Figure** **3**). Initially, various *N*‐protecting groups on phenylmethanamine were tested under optimized conditions. The results demonstrated that nonchelating protecting groups, including nicotinoyl, isonicotinoyl, and benzoyl, were ineffective in facilitating the reaction (Figure 3a). This further confirms the distinctiveness of our coordinating activation strategy. A competition experiment was carried out using aryl‐substituted alkynes with varying substituents (Figure 3b). The result showed that electron‐donating groups in aryl‐substituted alkynes resulted in lower reactivity. The reaction rate is highly sensitive to steric hindrance around the picolinamide, as evidenced by the competition experiment involving *N*‐benzyl‐substituted picolinamide **1a** and *N*‐benzhydryl‐substituted picolinamide **1r**, which exclusively yielded **3a** (Figure 3b) Notably, the coupling of *N*‐benzyl‐picolinamide **1a** with DCP without the alkyne led to the formation of an *N*‐methylation product (Figure 3c). This side reaction was unavoidable, with a small number of *N*‐methylation undesired products detected in each instance. Upon adding stoichiometric amounts of radical scavengers such as TEMPO and DPE to the model reaction under optimized conditions, the desired product **4a** was entirely inhibited. The presence of methyl and methylated vinyl radicals was subsequently verified by the identification of scavenger‐trapped adducts using high resolution mass spectrum, indicating the radical mechanism involved and suggesting the role of methyl and methylated vinyl radicals.

Based on the experimental findings and the compilation of relevant literature, a plausible reaction mechanism is depicted in Figure 3e. The initiation of this reaction involves the generation of a methyl radical from DCP, which may be catalyzed by copper(II) or induced by thermal decomposition.[^3^, ^13^] The subsequent addition of this radical to the alkyne is characterized by high regioselectivity, resulting in the formation of a methylated vinyl radical. Concurrently, picolinamide functions as a bidentate ligand, coordinating with copper(II) to form intermediate **IM1**. This coordinated species then traps the methylated vinyl radical, leading to the formation of a copper(III) intermediate **IM2**. Following this, a reductive elimination step occurs, resulting in the formation of the thermodynamically stable *trans*‐methylated enamide product. Finally, the low‐valent Cu(I) catalyst is reoxidized by DCP, thereby completing the catalytic cycle. This mechanism underscores the pivotal role of the coordination group in the reaction pathway, highlighting the importance of coordinating an activation strategy in the overall reaction mechanism.

## 3 Conclusion

In summary, we have established a novel three‐component process involving picolinamide derivatives, alkynes, and the methylation reagent DCP. This approach facilitates the modular synthesis of functionalized methyl‐containing enamide scaffolds. This protocol demonstrates a broad substrate scope for both picolinamide derivatives and alkynes, along with excellent functional group tolerance. The reaction‘s utility is further illustrated through the late‐stage modification of picolinamide and alkyne derivatives incorporating biologically relevant motifs. The strategy outlined here offers a straightforward approach to constructing methylated enamides, with potential applications in the biological sciences anticipated.

## Conflict of Interest

The authors declare no conflict of intetest.

## Supporting information

Supplementary Material

## Data Availability

The data that support the findings of this study are available from the corresponding author upon reasonable request.

## References

[1] a) For selected reviews, see: E. J. Barreiro , A. E. Kummerle , C. A. Fraga , Chem. Rev. 2011, 111, 5215; 21631125 10.1021/cr200060g

[2] a) For selected reviews, see: H. Schonherr , T. Cernak , Angew. Chem. 2013, 125, 12480; 10.1002/anie.201303207 24151256

[3] a) For selected reviews, see: Q. Dai , Y. Jiang , J.‐T. Yu , J. Cheng , Synthesis 2016, 48, 329;

[4] a) For selected reviews, see: V. P. Boyarskiy , D. S. Ryabukhin , N. A. A. Bokach , V. Vasilyev , Chem. Rev. 2016, 116, 5894; 27111159 10.1021/acs.chemrev.5b00514

[5] a) T. Tsuda , T. Yoshida , T. Saegusa , J. Org. Chem. 1988, 53, 607;

[6] a) R. Alfaro , A. Parra , J. Alemán , J. L. G. Ruano , M. Tortosa , J. Am. Chem. Soc. 2012, 134, 15165; 22950573 10.1021/ja307670k

[7] a) W. Li , S. Yu , J. Li , Y. Zhao , Angew. Chem. 2020, 132, 14510; 10.1002/anie.202006322 32449977

[8] a) S. Dutta , A. K. Sahoo , Angew. Chem. 2023, 135, e202300610; 10.1002/anie.202300610 36701082

[9] a) For selected references and reviews, see: R. Shen , C. T. Lin , E. J. Bowman , B. J. Bowman , J. A. Porco , J. Am. Chem. Soc. 2003, 125, 7889; 12823009 10.1021/ja0352350

[10] a) For selected examples on application of coordinating activation strategy in N—H bond functionalization, see: X. Li , B. Li , J. You , J. Lan , Org. Biomol. Chem. 2013, 11, 1925;23407643

[11] a) Q. Q. Xia , X. L. Liu , Y. J. Zhang , C. Chen , W. Z. Chen , Org. Lett. 2013, 15, 3326; 23789961 10.1021/ol401362k

[12] The data of crystal (CCDC: 2120423 for 3g) can be obtained free of charge from The Cambridge Crystallographic Data Center via http://www.ccdc.cam.ac.uk/data_request/cif (accessed: April 11, 2025).

[13] a) For selected examples, see: X. Bao , T. Yokoe , T. M. Ha , Q. Wang , J. Zhu , Nat. Commun. 2018, 9, 3725; 30213939 10.1038/s41467-018-06246-6 PMC6137206

