# Supplementary material for: Three‐Component 1,2‐Methylamidation of Alkynes via Coordinating Activation Strategy
Source: ChemistryOpen. 2025 Apr 17;14(10):e202500151. doi: 10.1002/open.202500151 (PMC12518046; doi:10.1002/open.202500151)
Supplement: Supplementary file 1 — Supplementary Material [file OPEN-14-e202500151-s001.pdf]

Supporting Information

Three-Component 1,2-Methylamidation of Alkynes via Coordinating Activation Strategy

Jing Ren,[a] Kaiyun Liu,[a] Ning Wang,[a] Jinlong Li,[a] Xinyu Long,[a] Chengming Li,[b] and Kaizhi Li[a,*]

[a]Institute of Biopharmaceuticals, West China Hospital, Sichuan University, 37 Guoxue Alley, Chengdu 610041, P. R. China. Email: kzli@scu.edu.cn

[b]Institute of Organ Transplantation, West China Hospital, Sichuan University, 37 Guoxue Alley, Chengdu 610041, P. R. China.

Table of contents

I. General remarks....2

II. Optimization of the 1,2-difunctional methylamidation of *N*-benzylpicolinamide **1a** with ethynylbenzene **2a**....2

III. General procedure for the 1,2-difunctional methylamidation of alkyne....4

IV. Synthetic manipulation.....4

V. Investigation of the reaction mechanism....6

VI. The method for crystal growth....11

VII. Unsuccessful substrates....13

VIII. Experimental data for the described substances....13

IX. References....50

X. Copies of NMR spectra....51

I. General remarks

NMR spectra were obtained on a Bruker AV II-400 MHz or a Varian Inova 400 MHz spectrometer. The aluminum block [9-hole inner diameter 26-27 mm, H200927 (Syhtnwre)] was used as heat source. The ^{1}H NMR (400 MHz) chemical shifts were measured relative to $CDCl_3$, Acetone-d_6, CD_3OD or TMS as the internal reference ($CDCl_3$: δ = 7.26 ppm, Acetone-d_6: δ = 2.05 ppm, CD_3OD: δ = 3.31 ppm, TMS: δ = 0.00 ppm). The ^{13}C NMR (100 MHz) chemical shifts were given using $CDCl_3$, Acetone-d_6 or CD_3OD as the internal standard ($CDCl_3$: δ = 77.16 ppm, Acetone-d_6: δ = 29.84, 206.26 ppm, CD_3OD: δ = 49.00 ppm). Chemical shifts δ are reported in ppm relative to residual solvent. Data are reported as follows: chemical shift, multiplicity (s = singlet, d = doublet, t = triplet, q = quartet, bs = broad singlet, m = multiplet), coupling constants (Hz), integration. High-resolution mass spectra (HRMS) were obtained with a high-resolution quadrupole-orbitrap tandem mass spectrometer (Q-Exactive plus; Thermo Fisher Scientific, Waltham, MA, USA) with electrospray ionization (ESI). Melting points were determined with XRC-1 and are uncorrected. X-Ray single-crystal diffraction data were collected on a Bruker D8 VENTURE single crystal diffraction.

Unless otherwise noted, all reagents and solvents were obtained from commercially available sources and used without further purification. Reactions were monitored by Thin Layer Chromatography (TLC) using UV light (254/365 nm) for detection. Products were purified by column chromatography, which was carried out on 200-300 mesh of silica gel purchased from Qing Dao Hai Yang Chemical Industry Co. 2-Picolinamide derivatives **1** were prepared according to the literature procedure.[1-3] Alkynes **2** were obtained from commercial suppliers and used without further purification.

II. Optimization of the 1,2-difunctional methylamidation of *N*-benzylpicolinamide 1a with ethynylbenzene 2a

An oven-dried Schlenk tube with a magnetic stir bar was charged with *N*-benzylpicolinamide **1a** (21.2 mg, 0.10 mmol, 1.0 equiv), ethynylbenzene **2a** (22.0 μl, 0.20 mmol, 2.0 equiv), methylation reagent, catalyst, and solvent under N_2 atmosphere. The tube was sealed with a teflon-coated cap and the reaction solution was heated at indicated temperature for indicated time. After being cooled to ambient temperature, the reaction mixture was quenched with 1 M sodium thiosulfate solution and the aqueous phase was extracted with EtOAc. The combined organic phases were washed with brine,

dried over Na_2SO_4. The solvent was removed under reduced pressure, and the residue was purified by column chromatography on silica gel (ethyl acetate/petroleum ether = 1/6, v/v) to provide the desired product **3a**.

Table S1: Optimization of the methylamidation of *N*-benzylpicolinamide **1a** with ethynylbenzene **2a**[a]

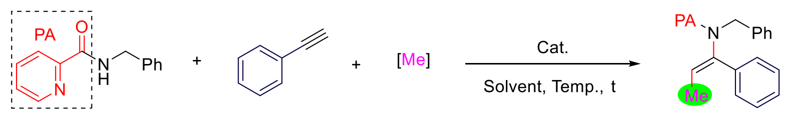

Entry	Cat. (equiv.)	[Me] (equiv.)	Solvent	Temp. (°C)	t (h)	Yield (%)[b]
1	$Cu(acac)_2$ (0.2)	DTBP (2.0)	Benzene	120	12	41
2	$Cu(acac)_2$ (0.2)	TBPB (2.0)	Benzene	120	12	40
3	$Cu(acac)_2$ (0.2)	TBHP (2.0)	Benzene	120	12	22
4	$Cu(acac)_2$ (0.2)	DCP (2.0)	Benzene	120	12	64
5	$Cu(acac)_2$ (0.1)	DCP (2.0)	Benzene	120	12	61
**6**	**$Cu(acac)_2$ (0.2)**	**DCP (1.5)**	**Benzene**	**120**	**12**	**68**
7	$Cu(acac)_2$ (0.2)	DCP (1.5)	Benzene	130	12	57
8	$Cu(acac)_2$ (0.2)	DCP (1.5)	Benzene	110	12	61
9	$Cu(acac)_2$ (0.2)	DCP (1.5)	Benzene	100	12	55
10	$Cu(acac)_2$ (0.2)	DCP (1.5)	Benzene	120	8	55
11	$Ni(acac)_2$ (0.2)	DCP (1.5)	Benzene	120	12	n.d.
12	$Co(acac)_2$ (0.2)	DCP (1.5)	Benzene	120	12	n.d.
13	$Mn(acac)_2$ (0.2)	DCP (1.5)	Benzene	120	12	n.d.
14	$Fe(acac)_2$ (0.2)	DCP (1.5)	Benzene	120	12	n.d.
15	$Cu(OAc)_2$ (0.2)	DCP (1.5)	Benzene	120	12	24
16	$CuBr_2$ (0.2)	DCP (1.5)	Benzene	120	12	26
17	CuCl (0.2)	DCP (1.5)	Benzene	120	12	22
18	CuTc (0.2)	DCP (1.5)	Benzene	120	12	23
19	$CuSO_4$ (0.2)	DCP (1.5)	Benzene	120	12	27
20	$Cu(hmacac)_2$ (0.2)	DCP (1.5)	Benzene	120	12	60
21	$Cu(tfacac)_2$ (0.2)	DCP (1.5)	Benzene	120	12	trace
22	$Cu(hfacac)_2$ (0.2)	DCP (1.5)	Benzene	120	12	trace
23	$Cu(acac)_2$ (0.2)	DCP (1.5)	$PhCF_3$	120	12	62
24	$Cu(acac)_2$ (0.2)	DCP (1.5)	PhCl	120	12	62
25	$Cu(acac)_2$ (0.2)	DCP (1.5)	Toluene	120	12	21
26	$Cu(acac)_2$ (0.2)	DCP (1.5)	1,2-DCE	120	12	trace
27	$Cu(acac)_2$ (0.2)	DCP (1.5)	THF	120	12	trace
28	$Cu(acac)_2$ (0.2)	DCP (1.5)	CH_3CN	120	12	32
29	$Cu(acac)_2$ (0.2)	DCP (1.5)	DMF	120	12	n.d.
30	$Cu(acac)_2$ (0.2)	DCP (1.5)	DMSO	120	12	n.d.
31	$Cu(acac)_2$ (0.2)	DCP (1.5)	EtOH	120	12	trace
32[c]	$Cu(acac)_2$ (0.2)	DCP (1.5)	Toluene/CH_3CN	120	12	47

33[d]	$Cu(acac)_2$ (0.2)	DCP (1.5)	Benzene	120	12	55
34[e]	$Cu(acac)_2$ (0.2)	DCP (1.5)	Benzene	120	12	58
35[f]	$Cu(acac)_2$ (0.2)	DCP (1.5)	Benzene	120	12	59
36[g]	$Cu(acac)_2$ (0.2)	DCP (1.5)	Benzene	120	12	53
37	--	DCP (1.5)	Benzene	120	12	n.d.
38	$Cu(acac)_2$ (0.2)	--	Benzene	120	12	n.d.

[a]Reaction conditions: **1a** (21.2 mg, 0.10 mmol, 1.0 equiv.), **2a** (22.0 μl, 0.20 mmol, 2.0 equiv.), cat., [Me] and solvent (1.0 mL) at indicated temperature under N_2. [b]Isolated yield after chromatographic purification. [c]Toluene/CH_3CN (0.5 mL/0.5 mL) was used. [d]1,10-Phenanthroline (0.2 equiv.) was added. [e]Bipyridine (0.2 equiv.) was added. [f]The reaction under an air atmosphere. [g]**2a** (1.5 equiv.) was used. [Me] = Methylation reagent. DTBP = Di-*tert*-butyl peroxide. TBHP = *tert*-Butyl hydroperoxide. TBPB = *tert*-Butyl peroxybenzoate. DCP = Dicumyl peroxide. $Cu(hmacac)_2$ = Copper(II) bis(2,2,6,6-tetramethyl-3,5-heptanedionate). $Cu(tfacac)_2$ = Copper(II) trifluoroacetylacetonate. $Cu(hfacac)_2$ = Copper(II) hexafluor-2,4-pentanedionate. n.d. = no product detected. PA = 2-Pyridylacyl.

III. General procedure for the 1,2-difunctional methylamidation of alkyne

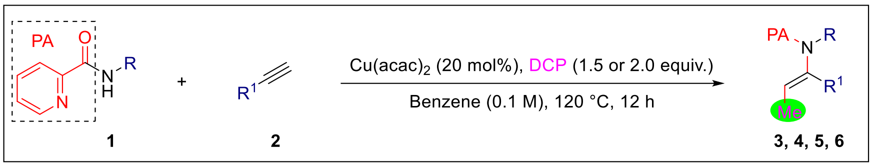

An oven-dried Schlenk tube with a magnetic stir bar was charged with 2-picolinamide derivative **1** (0.1 mmol, 1.0 equiv.), alkyne **2** (0.20 mmol, 2.0 equiv.), dicumyl peroxide (DCP, 0.15 mmol or 0.2 mmol, 1.5 equiv. or 2.0 equiv.), $Cu(acac)_2$ (5.2 mg, 0.02 mmol, 0.2 equiv.) and benzene (1.0 mL). Then the tube was sealed with a teflon-coated cap under N_2 atmosphere and the reaction mixture was stirred at room temperature for several minutes. Then the mixture was stirred at 120 °C for 12 h. After being cooled to ambient temperature, the reaction mixture was quenched with 1 M sodium thiosulfate solution and the aqueous phase was extracted with EtOAc. The combined organic phases were washed with brine, dried over Na_2SO_4. The solvent was removed under reduced pressure, and the residue was purified by flash chromatography on silica gel to provide the desired products.

IV. Synthetic manipulation

a) Gram-scale synthesis of 3a and 4x

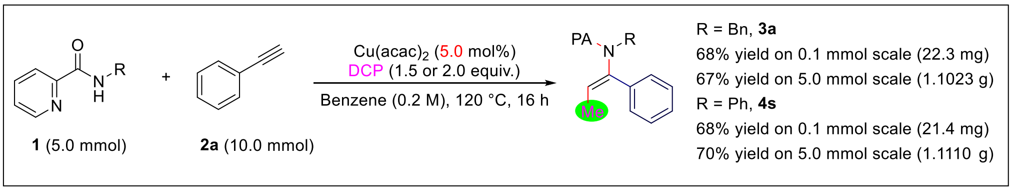

2-picolinamide derivative **1** (5.0 mmol, 1.0 equiv.), ethynylbenzene **2a** (10.0 mmol, 2.0 equiv., 1.02 g/1.10 ml), dicumyl peroxide (DCP, 7.5 mmol, 1.5 equiv. 2.03 g for **3a**; 2.0 equiv., 2.70 g for **4s**), Cu(acac)$_2$ (65.4 mg, 0.25 mmol, 0.05 equiv.) were added sequentially into a Schlenk tube under nitrogen, then the tube was capped with a rubber stopper. Benzene (25.0 mL) was then added by syringe. Then the tube was sealed with a teflon-coated cap under N_2 atmosphere and the reaction mixture was stirred at room temperature for several minutes. Then the mixture was stirred at 120 ^oC for 16 h. After being cooled to ambient temperature, the reaction mixture was quenched with 1 M sodium thiosulfate solution and the aqueous phase was extracted with EtOAc. The combined organic phases were washed with brine, dried over Na_2SO_4. The solvent was removed under reduced pressure, and the residue was purified by flash chromatography on silica gel to afford product **3a** (1.10 g, 67% yield) or **4s** (1.11 g, 70% yield).

b) Procedure for the hydrogenation of 3a or 4s

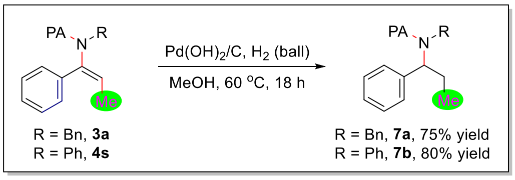

The product **3a** (65,7 mg, 0.2 mmol) or **4s** (62.9 mg, 0.2 mmol) and 10% Pd/C (24.0 mg) were added sequentially into a flask Schlenk tube under nitrogen, then the tube was capped by a rubber stopper. The nitrogen in the tube was then evacuated and backfilled with hydrogen by using a hydrogen balloon. MeOH (2.0 mL) was then added by syringe. The resulting mixture was stirred at 60 ^oC for 18 hours as monitored by TLC. Upon completion, solvent was removed under vacuum and the residue was purified by flash silica gel column chromatography using eluent petroleum ether/ethyl acetate as eluent to afford pure product **7a** (49.6 mg, 75% yield) or **7b** (51.2 mg, 80% yield).

c) The procedure for the synthesis of 7c

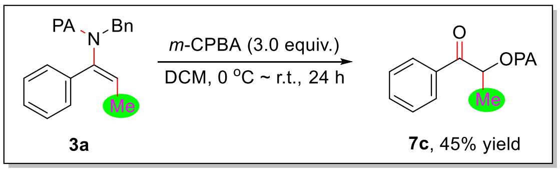

The product enamide **3a** (65.7 mg, 0.2 mmol, 1.0 equiv.) was added into a reaction tube. Then *m*-CPBA (103.5 mg, 0.6 mmol, 3.0 equiv) was added to the stirred solution of the enamide in CH_2Cl_2

(2.0 mL) at 0 °C and the resultant suspension was stirred for 30 min before warming to room temperature. The resulting mixture was stirred at room temperature for another 24 hours. Upon completion, the solvent was then removed under vacuum. The residue was purified directly by silica gel chromatography, eluting with ethyl acetate/petroleum ether to give α-pyridine-acyloxyketone **7c** (23.1 mg, 45% yield).

d) The procedure for the removal of PA group

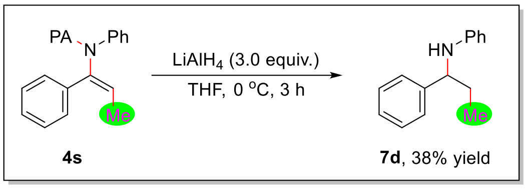

Compound enamide **4s** (62.9 mg, 0.2 mmol, 1.0 equiv.) was dissolved in anhydrous THF (1.5 mL) in an ovendried 10 mL vial at 0 °C, and $LiAlH_4$ (22.8 mg, 0.6 mmol, 3.0 equiv.) was added. The mixture was stirred at same temperature for 4 hours, then aq NH_4Cl (sat.) followed by 1 M NaOH (aq) was added. The mixture was extracted with CH_2Cl_2, the combined organic layers were dried with anhydrous Na_2SO_4 and concentrated in vacuo. The resulting residue was purified by silica gel flash chromatography to give **7d** as a colourless oil (16.0 mg, 38% yield).

V. Investigation of the reaction mechanism

a) The effect of N-protecting groups

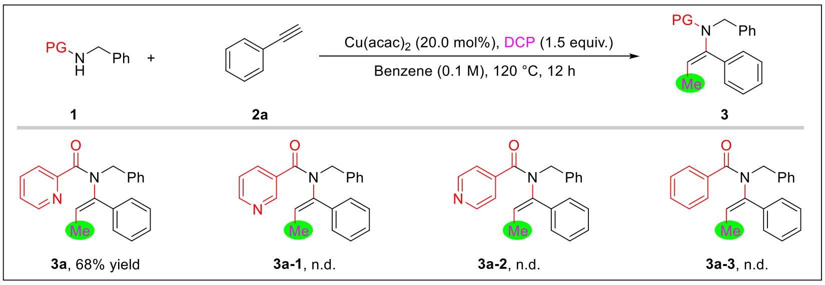

In a 25 mL Schlenk tube equipped with a stir bar was charged with **1** (0.10 mmol, 1.0 equiv.), **2a** (22.0 μl, 0.2 mmol, 2.0 equiv.), DCP (40.5 mg, 0.15 mmol, 1.5 equiv.) and Cu(acac)$_2$ (5.2 mg, 0.02 mmol, 0.2 equiv.) in benzene (1.0 mL). The reaction was stirred at 120 °C for 12 h under N_2 atmosphere. After being cooled to ambient temperature, the reaction mixture was quenched with 1 M sodium thiosulfate solution and the aqueous phase was extracted with EtOAc. The combined organic phases were washed with brine and dried over Na_2SO_4. The mixture was monitored by Thin Layer

Chromatography (TLC), then organic solvent was removed under reduced pressure and the residue was purified by flash silica gel column chromatography using petroleum ether and ethyl acetate as eluents to obtain the products **3** (if necessary).

b) Intermolecular competition experiments

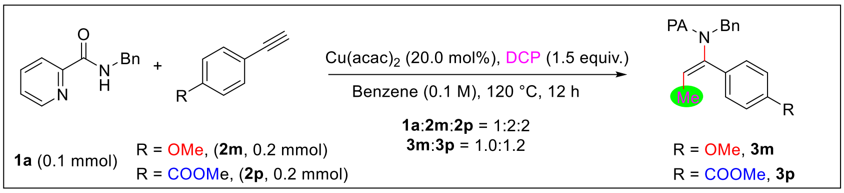

In a 25 mL Schlenk tube equipped with a stir bar was charged with **1a** (21.2 mg, 0.10 mmol, 1.0 equiv.), 1-ethynyl-4-methoxybenzene (**2m**, 26.4 mg, 0.2 mmol, 2.0 equiv), methyl 4-ethynylbenzoate (**2p**, 32.0 mg, 0.2 mmol, 2.0 equiv), DCP (40.5 mg, 0.15 mmol, 1.5 equiv.) and Cu(acac)$_2$ (5.2 mg, 0.02 mmol, 0.2 equiv.) in benzene (1.0 mL). The reaction was stirred at 120 °C for 12 h under N_2 atmosphere. After being cooled to ambient temperature, the reaction mixture was quenched with 1 M sodium thiosulfate solution and the aqueous phase was extracted with EtOAc. The combined organic phases were washed with brine, dried over Na_2SO_4. The solvent was removed under reduced pressure, and the residue was purified by flash silica gel column chromatography using petroleum ether and ethyl acetate as eluents to obtain the products **3m** and **3p** as the mixture. Then the mixture was detected by ^{1}H NMR to obtain the ratio.

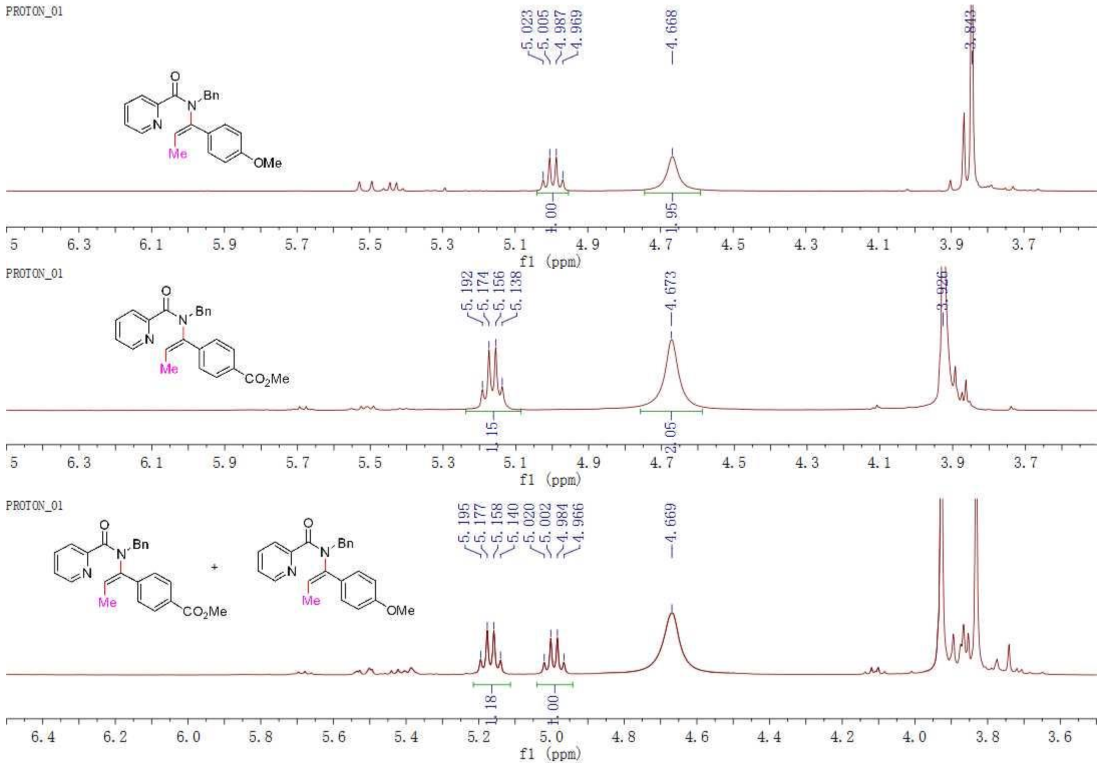

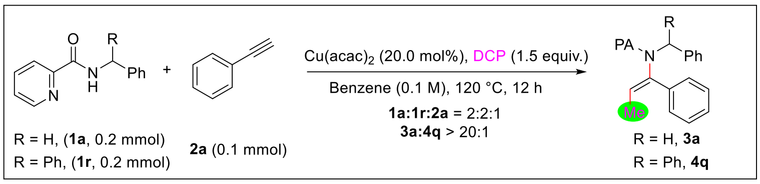

In a 25 mL Schlenk tube equipped with a stir bar was charged with **1a** (42.4 mg, 0.20 mmol, 2.0 equiv.), **1r** (55.6 mg, 0.20 mmol, 2.0 equiv.), **2a** (11.0 μl, 0.1 mmol, 1.0 equiv.), DCP (40.5 mg, 0.15 mmol, 1.5 equiv.) and Cu(acac)$_2$ (5.2 mg, 0.02 mmol, 0.2 equiv.) in benzene (1.0 mL). The reaction was stirred at 60 °C for 6 h under N_2 atmosphere. After being cooled to ambient temperature, the reaction mixture was quenched with 1 M sodium thiosulfate solution and the aqueous phase was extracted with EtOAc. The combined organic phases were washed with brine, dried over Na_2SO_4. The solvent was removed under reduced pressure, and the residue was purified by flash silica gel column chromatography using petroleum ether and ethyl acetate as eluents to obtain the product **3a** (13.8 mg, 42% yield), and no obvious product **4q**.

c) Two-component coupling experiment

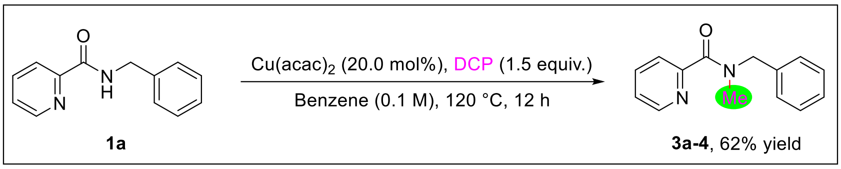

In a 25 mL Schlenk tube equipped with a stir bar was charged with **1a** (21.2 mg, 0.10 mmol, 1.0 equiv.), DCP (40.5 mg, 0.15 mmol, 1.5 equiv.) and Cu(acac)$_2$ (5.2 mg, 0.02 mmol, 0.2 equiv.) in benzene (1.0 mL). The reaction was stirred at 120 °C for 12 h under N_2 atmosphere. After being cooled to ambient temperature, the reaction mixture was quenched with 1 M sodium thiosulfate solution and the aqueous phase was extracted with EtOAc. The combined organic phases were washed with brine and dried over Na_2SO_4. The solvent was removed under reduced pressure, and the residue was purified by flash silica gel column chromatography using petroleum ether and ethyl acetate as eluents to obtain the products **3a-4** (14.0 mg, 62% yield).

d) Radical scavenger experiments

These reactions were conducted with general procedure with minor modification. In a 25 mL Schlenk tube equipped with a stir bar was charged with **1a** (21.2 mg, 0.10 mmol, 1.0 equiv.),

ethynylbenzene (**2a**, 22.0 ul, 0.2 mmol, 2.0 equiv), DCP (40.5 mg, 0.15 mmol, 1.5 equiv.), $Cu(acac)_2$ (5.2 mg, 0.02 mmol, 0.2 equiv.) and additive (0.2 mmol, 2.0 equiv.) in benzene (1.0 mL). The reaction was stirred at 120 °C for 12 h under N_2 atmosphere. After being cooled to ambient temperature, the crude mixture was firstly analyzed by HRMS (High Resolution Mass Spectrometry). Then the solvent was removed under reduced pressure, and the residue was purified by flash chromatography on silica gel to afford product **3a** to determine the yield (if necessary).

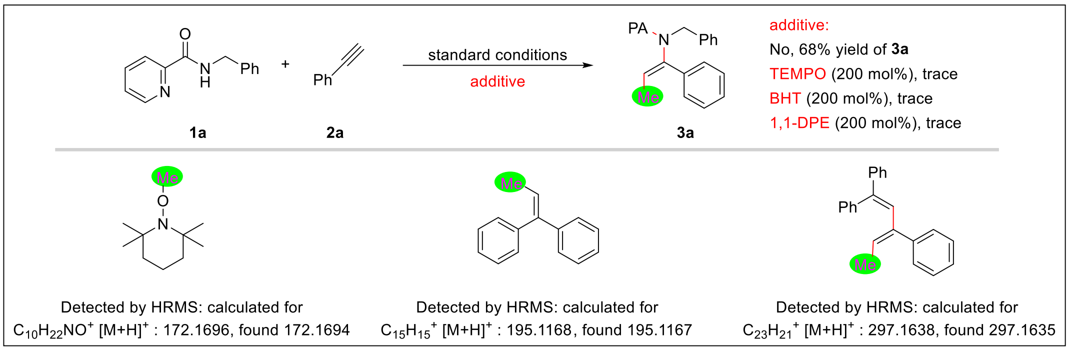

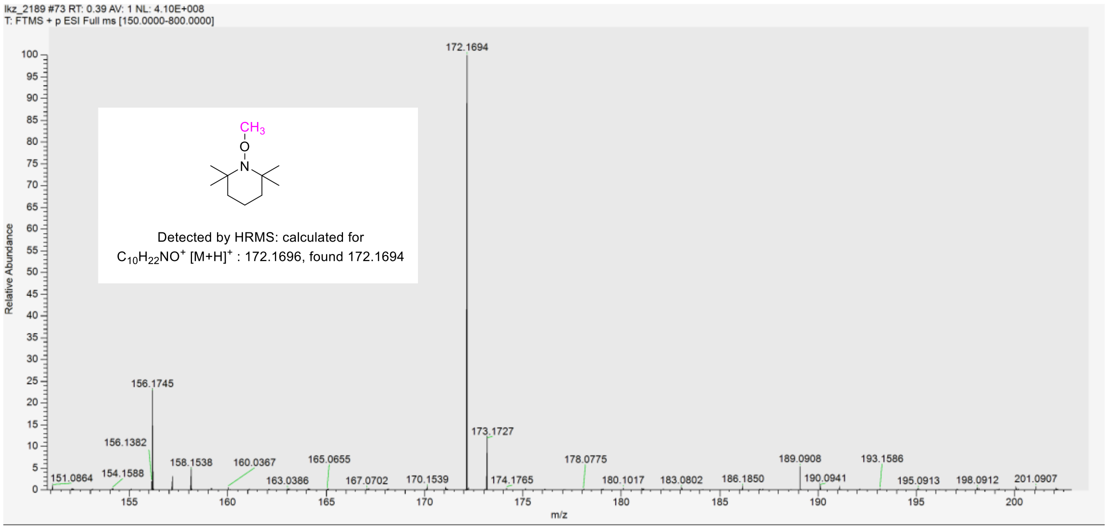

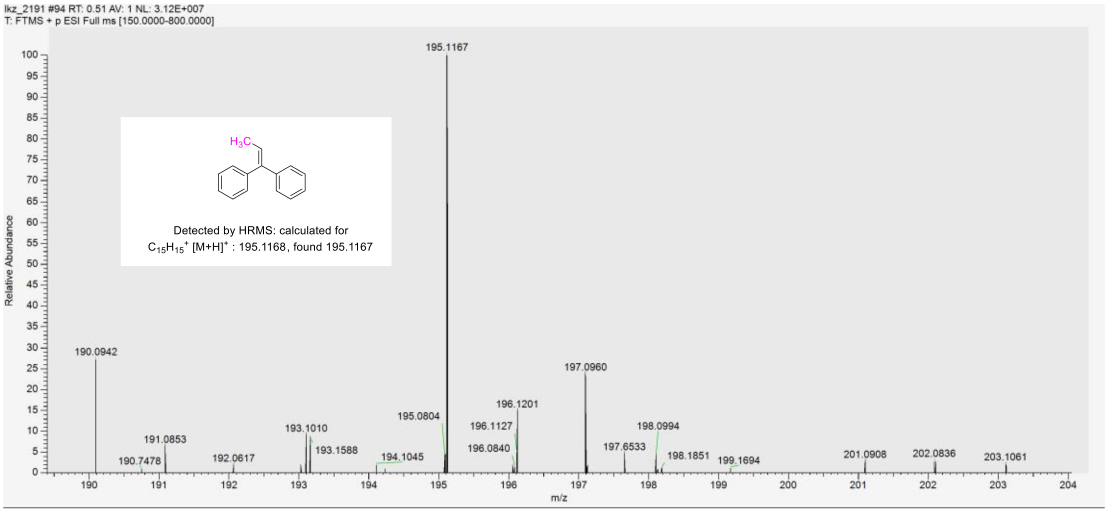

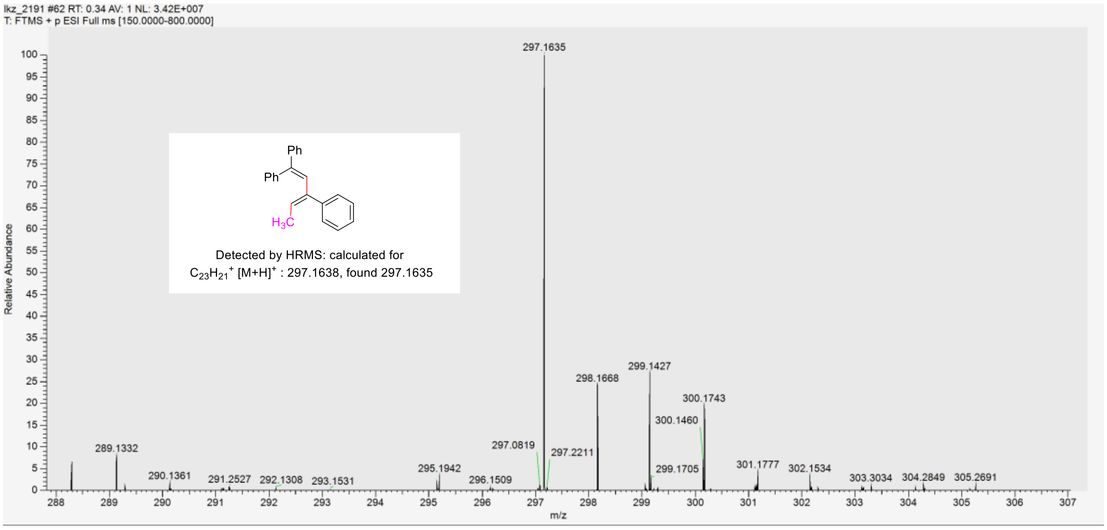

e) Proposed catalytic cycle

In a 25 mL Schlenk tube equipped with a stir bar was charged with **1a** (21.2 mg, 0.10 mmol, 1.0 equiv.), ethynylbenzene (**2a**, 22.0 ul, 0.2 mmol, 2.0 equiv), DCP (40.5 mg, 0.15 mmol, 1.5 equiv.), and $Cu(acac)_2$ (5.2 mg, 0.02 mmol, 0.2 equiv.) in benzene (1.0 mL). The reaction was stirred at 120 °C for 1 h under N_2 atmosphere. After being cooled to ambient temperature, the crude mixture was analyzed by HRMS (High Resolution Mass Spectrometry). The copper complexes **IM1a** and **IM2a** could be detected by HRMS analysis of the reaction mixtures.

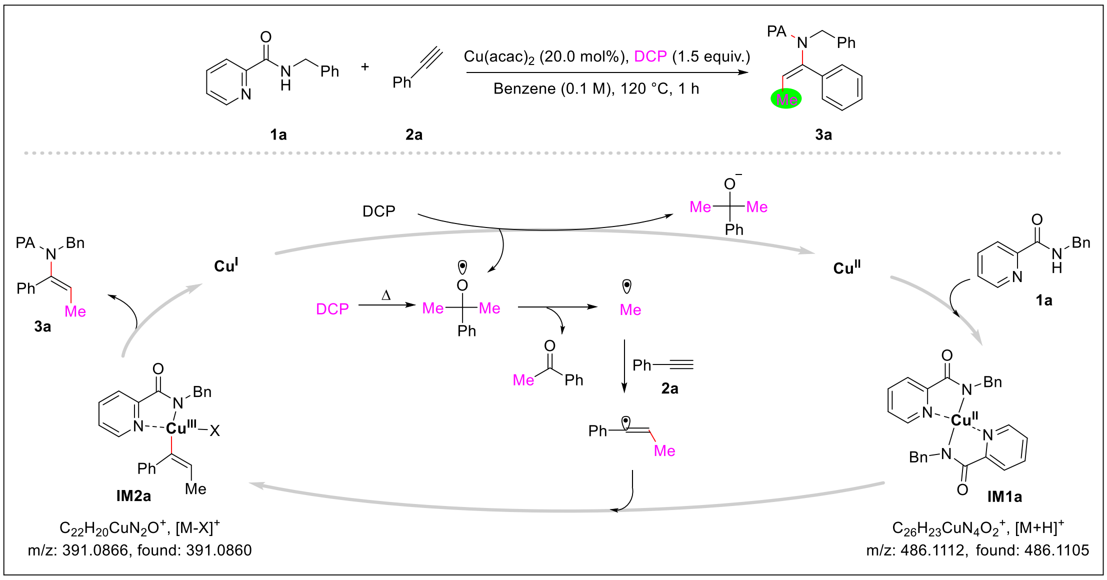

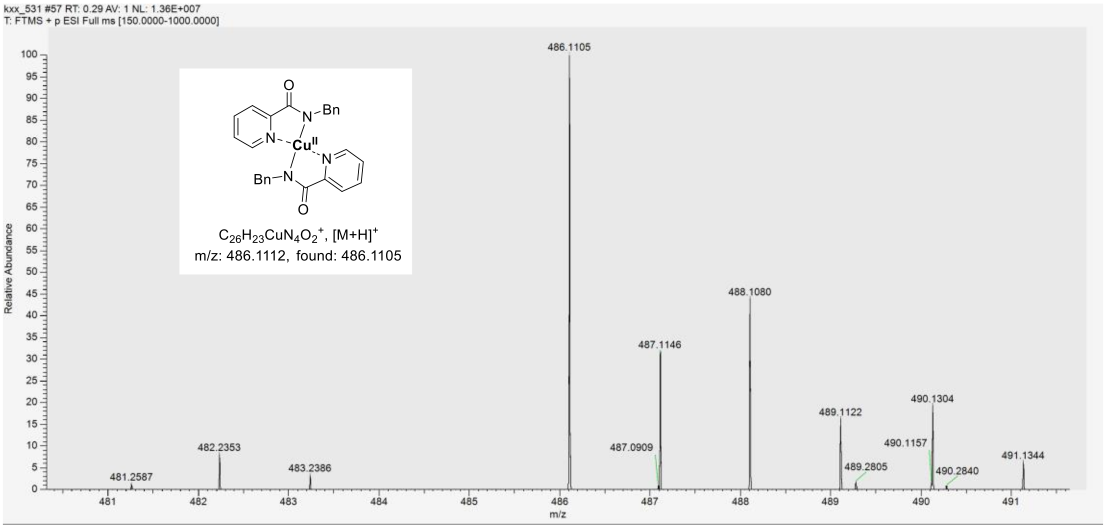

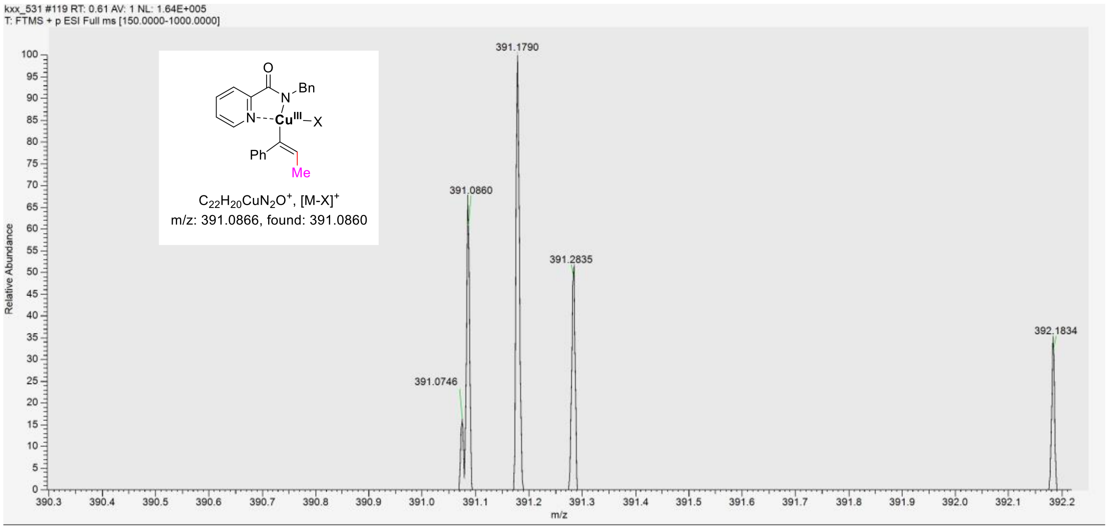

VI. The method for crystal growth

Procedure for the crystal growth of **3g**: To a 2.5 mL sample bottle containing **3g** (about 10 mg) was added ethyl acetate (about 0.5 mL), then 1.5 mL n-hexane was added slowly. The sample bottle was sealed by sealing film. Then several pinholes were made to slowly evaporate the solvents at room temperature. The desired light yellow crystals were obtained for several days (about 7-8 days).

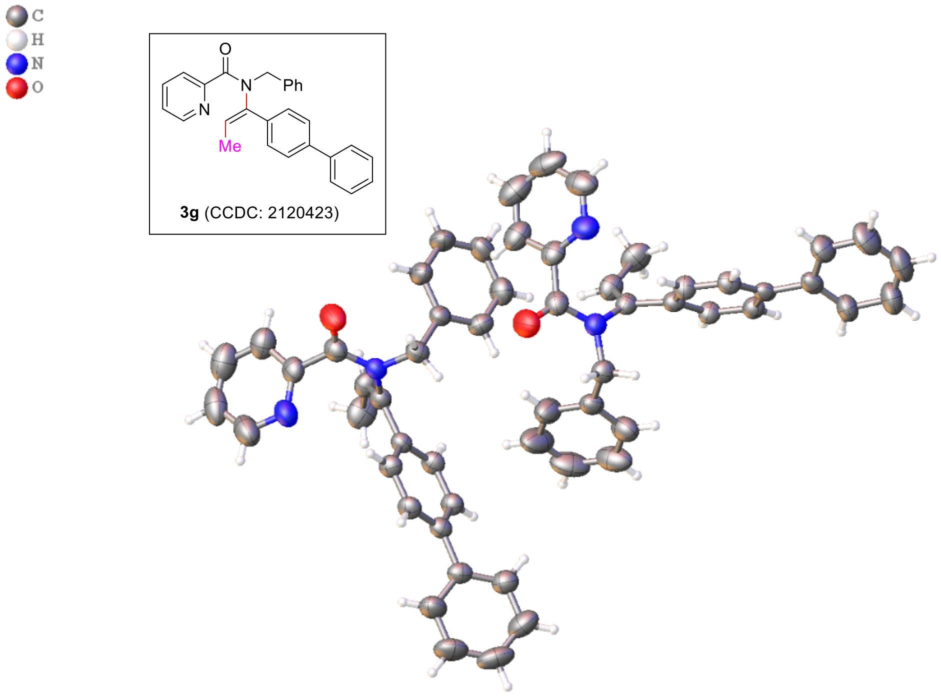

X-ray crystal structures of **3g** (The X-ray crystallographic structures are ORTEP representation with 50% probability thermal ellipsoids)

Table S2. Crystal data and structure refinement for **3g** (CCDC: 2120423)

Identification code	CCDC 2120423
Empirical formula	$C_{28}H_{24}N_2O$
Formula weight	404.49
Temperature/K	302.0
Crystal system	monoclinic
Space group	$P2_1$
a/Å	13.6892(13)
b/Å	11.1775(9)
c/Å	16.2855(16)
α/°	90
β/°	114.235(3)
γ/°	90
Volume/Å^3	2272.3(4)
Z	4
ρ_{calc}g/cm^3	1.182
μ/mm^{-1}	0.072
F(000)	856.0

Crystal size/mm^3	0.25 × 0.23 × 0.11
Radiation	MoKα (λ = 0.71073)
2Θ range for data collection/°	4.562 to 55.024
Index ranges	-17 ≤ h ≤ 17, -14 ≤ k ≤ 13, -21 ≤ l ≤ 21
Reflections collected	44409
Independent reflections	10014 [R_{int} = 0.1331, R_{sigma} = 0.1109]
Data/restraints/parameters	10014/1/561
Goodness-of-fit on F^2	0.963
Final R indexes [I>=2σ (I)]	R_1 = 0.0534, wR_2 = 0.1190
Final R indexes [all data]	R_1 = 0.1007, wR_2 = 0.1359
Largest diff. peak/hole / e Å^{-3}	0.17/-0.18
Flack parameter	-2.1(10)

VII. Unsuccessful substrates

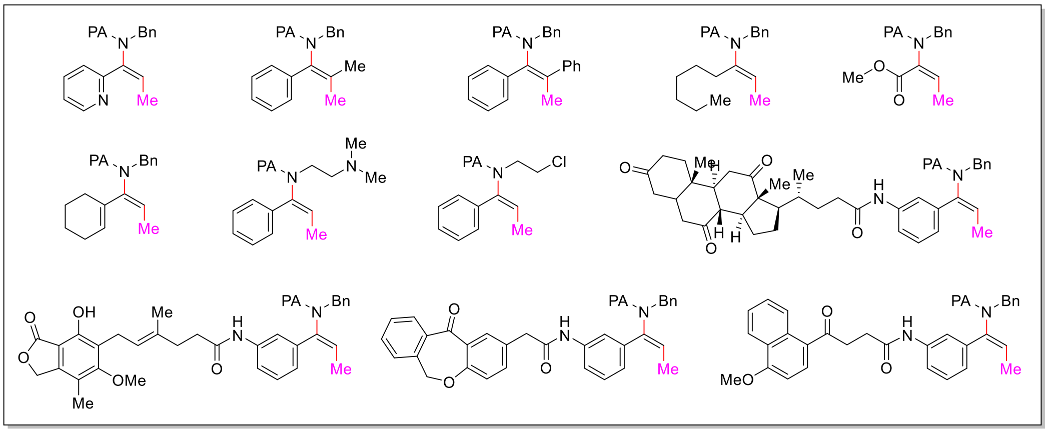

VIII. Experimental data for the described substances

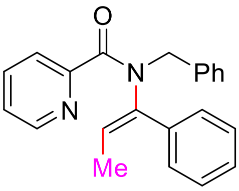

(*E*)-*N*-benzyl-*N*-(1-phenylprop-1-en-1-yl)picolinamide (**3a**)

Purification via column chromatography on silica gel (ethyl acetate/petroleum ether = 1/6, v/v) afforded **3a** as yellow oil (22.3 mg, 68% yield, Z/E = 1:15).

^{1}**H NMR** (400 MHz, $CDCl_3$): δ = 8.49 (d, J = 4.6 Hz, 1H), 7.64 (t, J = 7.6 Hz, 1H), 7.57 (d, J = 7.8 Hz, 1H),

7.49 (d, *J* = 7.1 Hz, 2H), 7.40–7.15 (m, 9H), 5.09 (q, *J* = 7.2 Hz, 1H), 4.68 (brs, 2H), 1.38 (d, *J* = 7.3 Hz, 3H) ppm.

^{13}**C NMR** (100 MHz, $CDCl_3$): *δ* = 169.65, 155.25, 148.35, 139.83, 137.42, 136.30, 135.20, 130.00, 129.09, 128.38, 128.32, 128.27, 127.32, 125.84, 123.88, 123.23, 49.80, 14.29 ppm.

HRMS (ESI^+): calcd for $C_{22}H_{21}N_2O^+$ $[M+H]^+$ 329.1648, found 329.1645.

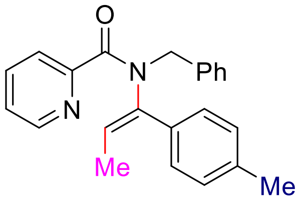

(*E*)-*N*-benzyl-*N*-(1-(*p*-tolyl)prop-1-en-1-yl)picolinamide (**3b**)

Purification via column chromatography on silica gel (ethyl acetate/petroleum ether = 1/6, v/v) afforded **3b** as yellow oil (20.0 mg, 58% yield, Z/E = 1:10).

^{1}**H NMR** (400 MHz, $CDCl_3$): *δ* = 8.49 (d, *J* = 4.6 Hz, 1H), 7.64 (td, *J* = 7.7, 1.6 Hz, 1H), 7.57 (d, *J* = 7.8 Hz, 1H), 7.39 (d, *J* = 8.0 Hz, 2H), 7.32–7.22 (m, 5H), 7.20–7.16 (m, 3H), 5.03 (q, *J* = 7.2 Hz, 1H), 4.69–4.67 (m, 2H), 2.39 (s, 3H), 1.36 (d, *J* = 7.3 Hz, 3H) ppm.

^{13}**C NMR** (100 MHz, $CDCl_3$): *δ* = 169.64, 155.33, 148.38, 139.65, 138.19, 137.46, 136.23, 132.16, 129.89, 129.11, 129.07, 128.35, 127.28, 125.62, 123.81, 123.11, 49.61, 21.43, 14.29 ppm.

HRMS (ESI^+): calcd for $C_{23}H_{23}N_2O^+$ $[M+H]^+$ 343.1805, found 343.1804.

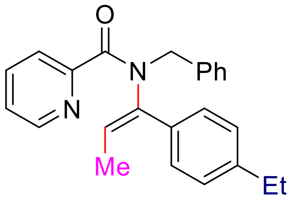

(*E*)-*N*-benzyl-*N*-(1-(4-ethylphenyl)prop-1-en-1-yl)picolinamide (**3c**)

Purification via column chromatography on silica gel (ethyl acetate/petroleum ether = 1/6, v/v) afforded **3c** as pale yellow oil (18.7 mg, 52% yield, Z/E = 1:10).

^{1}**H NMR** (400 MHz, $CDCl_3$): *δ* = 8.49 (d, *J* = 4.5 Hz, 1H), 7.63 (t, *J* = 7.6 Hz, 1H), 7.56 (d, *J* = 7.8 Hz, 1H), 7.41 (d, *J* = 7.9 Hz, 2H), 7.38–7.09 (m, 8H), 5.05 (q, *J* = 7.2 Hz, 1H), 4.68 (brs, 2H), 2.68 (q, *J* = 7.6 Hz, 2H), 1.38 (d, *J* = 7.2 Hz, 3H), 1.28 (t, *J* = 7.4 Hz, 3H) ppm.

^{13}**C NMR** (100 MHz, $CDCl_3$): *δ* = 169.62, 155.35, 148.37, 144.42, 139.69, 137.47, 136.18, 132.39, 129.91,

129.11, 128.31, 127.81, 127.25, 125.52, 123.75, 123.06, 49.66, 28.75, 15.51, 14.28 ppm.

HRMS (ESI$^+$): calcd for $C_{24}H_{25}N_2O^+$ [M+H]$^+$ 357.1961, found 357.1957.

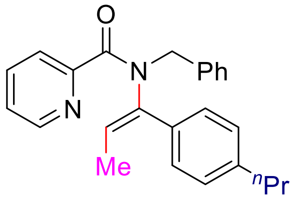

(*E*)-*N*-benzyl-*N*-(1-(4-propylphenyl)prop-1-en-1-yl)picolinamide (**3d**)

Purification via column chromatography on silica gel (ethyl acetate/petroleum ether = 1/7, v/v) afforded **3d** as yellow oil (21.3 mg, 57% yield, Z/E = 1:9).

^{1}**H NMR** (400 MHz, $CDCl_3$): δ = (d, *J* = 4.6 Hz, 1H), 7.62 (t, *J* = 7.6 Hz, 1H), 7.54 (d, *J* = 7.7 Hz, 1H), 7.37 (d, *J* = 7.9 Hz, 2H), 7.33–7.20 (m, 5H), 7.18–7.16 (m, 3H), 5.07 (q, *J* = 7.2 Hz, 1H), 4.68 (brs, 2H), 2.61 (t, *J* = 7.6, 2H), 1.72–1.63 (m, 2H), 1.38 (d, *J* = 7.2 Hz, 3H), 0.97 (t, *J* = 7.3 Hz, 3H) ppm.

^{13}**C NMR** (100 MHz, $CDCl_3$): δ = 169.64, 155.38, 148.38, 142.89, 139.79, 137.49, 136.19, 132.47, 129.79, 129.11, 128.40, 128.33, 127.26, 125.36, 123.75, 123.07, 49.80, 37.95, 24.53, 14.29, 14.00 ppm.

HRMS (ESI$^+$): calcd for $C_{25}H_{27}N_2O^+$ [M+H]$^+$ 371.2118, found 371.2114.

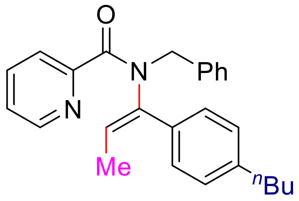

(*E*)-*N*-benzyl-*N*-(1-(4-butylphenyl)prop-1-en-1-yl)picolinamide (**3e**)

Purification via column chromatography on silica gel (ethyl acetate/petroleum ether = 1/7, v/v) afforded **3e** as yellow oil (20.9 mg, 54% yield, Z/E = 1:10).

^{1}**H NMR** (400 MHz, $CDCl_3$): δ = 8.49 (d, *J* = 4.2 Hz, 1H), 7.62 (t, *J* = 7.3 Hz,, 1H), 7.54 (d, *J* = 7.7 Hz, 1H), 7.37 (d, *J* = 7.7 Hz, 2H), 7.33–7.21 (m, 5H), 7.18–7.16 (m, 3H), 5.07 (q, *J* = 7.2 Hz, 1H), 4.69 (brs, 2H), 2.63 (t, *J* = 7.8 Hz, 2H), 1.67–1.59 (m, 3H), 1.42– 1.34 (m, 5H), 0.96 (t, *J* = 7.3 Hz, 3H) ppm.

^{13}**C NMR** (100 MHz, $CDCl_3$): δ = 169.63, 155.34, 148.38, 143.11, 139.75, 137.46, 136.18, 132.38, 129.78, 129.10, 128.34, 128.32, 127.25, 125.37, 123.75, 123.04, 49.77, 35.55, 33.62, 22.52, 14.29, 14.09 ppm.

HRMS (ESI$^+$): calcd for $C_{26}H_{29}N_2O^+$ [M+H]$^+$ 385.2274, found 385.2271.

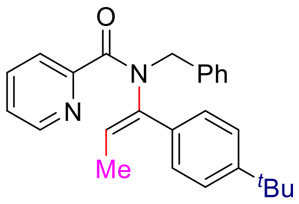

(*E*)-*N*-benzyl-*N*-(1-(4-(*tert*-butyl)phenyl)prop-1-en-1-yl)picolinamide (**3f**)

Purification via column chromatography on silica gel (ethyl acetate/petroleum ether = 1/7, v/v) afforded **3f** as yellow oil (21.2 mg, 55% yield, Z/E = 1:10).

^{1}**H NMR** (400 MHz, $CDCl_3$): δ = 8.49 (d, *J* = 4.7 Hz, 1H), 7.62 (td, *J* = 7.7, 1.6 Hz, 1H), 7.53 (d, *J* = 7.8 Hz, 1H), 7.42–7.34 (m, 4H), 7.33–7.22 (m, 5H), 7.19–7.14 (m, 1H), 5.07 (q, *J* = 7.3 Hz, 1H), 4.69 (brs, 2H), 1.39 (d, *J* = 7.3 Hz, 3H), 1.35 (s, 9H) ppm.

^{13}**C NMR** (100 MHz, $CDCl_3$): δ = 169.66, 155.41, 151.25, 148.45, 139.66, 137.52, 136.18, 132.16, 129.60, 129.18, 128.34, 127.28, 125.53, 125.23, 123.74, 123.02, 49.79, 34.79, 31.45, 14.34 ppm.

HRMS (ESI^+): calcd for $C_{26}H_{29}N_2O^+$ $[M+H]^+$ 385.2274, found 385.2270.

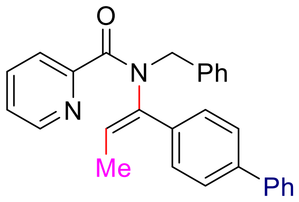

(*E*)-*N*-(1-([1,1'-biphenyl]-4-yl)prop-1-en-1-yl)-*N*-benzylpicolinamide (**3g**)

Purification via column chromatography on silica gel (ethyl acetate/petroleum ether = 1/6, v/v) afforded **3g** as a pale yellow solid (23.9 mg, 59% yield, Z/E = 1:10). M.p.: 112–115 °C.

^{1}**H NMR** (400 MHz, $CDCl_3$): δ = 8.51 (d, *J* = 4.4 Hz, 1H), 7.69–7.53 (m, 8H), 7.47 (t, *J* = 7.6 Hz, 2H), 7.42–7.23 (m, 6H), 7.23–7.16 (m, 1H), 5.12 (q, *J* = 7.2 Hz, 1H), 4.74 (brs, 2H), 1.43 (d, *J* = 7.3 Hz, 3H) ppm.

^{13}**C NMR** (100 MHz, $CDCl_3$): δ = 169.67, 155.23, 148.36, 140.98, 140.68, 139.59, 137.42, 136.32, 134.16, 130.42, 129.14, 128.96, 128.39, 127.61, 127.34, 127.16, 126.99, 126.05, 123.91, 123.27, 49.86, 14.38 ppm.

HRMS (ESI^+): calcd for $C_{28}H_{25}N_2O^+$ $[M+H]^+$ 405.1961, found 405.1957.

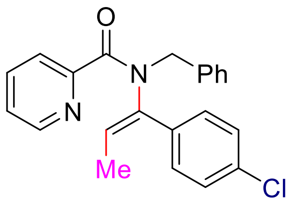

(*E*)-*N*-benzyl-*N*-(1-(4-chlorophenyl)prop-1-en-1-yl)picolinamide (**3h**)

Purification via column chromatography on silica gel (ethyl acetate/petroleum ether = 1/6, v/v) afforded **3h** as pale yellow oil (22.7 mg, 62% yield, Z/E = 1:10).

^{1}**H NMR** (400 MHz, $CDCl_3$): δ = 8.46 (d, J = 4.4 Hz, 1H), 7.67 (t, J = 7.6 Hz, 1H), 7.61 (d, J = 7.6 Hz, 1H), 7.46 (d, J = 8.2 Hz, 2H), 7.37–7.15 (m, 8H), 5.07 (q, J = 7.2 Hz, 1H), 4.67 (brs, 2H), 1.34 (d, J = 7.2 Hz, 3H) ppm.

^{13}**C NMR** (100 MHz, $CDCl_3$): δ = 169.55, 154.97, 148.17, 139.08, 137.25, 136.44, 134.08, 133.75, 131.46, 129.04, 128.52, 128.42, 127.40, 126.20, 124.08, 123.54, 49.79, 14.22 ppm.

HRMS (ESI^+): calcd for $C_{22}H_{20}{}^{35}ClN_2O^+$ $[M+H]^+$ 363.1259, found 363.1257; $C_{22}H_{20}{}^{37}ClN_2O^+$ $[M+H]^+$ 365.1229, found 365.1224.

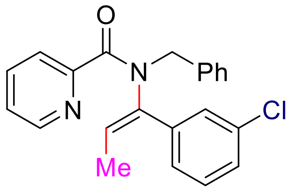

(*E*)-*N*-benzyl-*N*-(1-(3-chlorophenyl)prop-1-en-1-yl)picolinamide (**3i**)

Purification via column chromatography on silica gel (ethyl acetate/petroleum ether = 1/6, v/v) afforded **3i** as pale yellow oil (19.7 mg, 54% yield, Z/E = 1:10).

^{1}**H NMR** (400 MHz, $CDCl_3$): δ = 8.48 (d, J = 4.4 Hz, 1H), 7.67 (t, J = 7.6 Hz, 1H), 7.60 (d, J = 7.7 Hz, 1H), 7.51 (s, 1H), 7.39–7.18 (m, 9H), 5.11 (q, J = 7.2 Hz, 1H), 4.68 (brs, 2H), 1.37 (d, J = 7.2 Hz, 3H) ppm.

^{13}**C NMR** (100 MHz, $CDCl_3$): δ = 169.52, 154.86, 148.21, 139.00, 137.34, 137.25, 136.45, 134.23, 130.06, 129.53, 129.04, 128.45, 128.38, 138.26, 127.45, 126.52, 124.10, 123.57, 50.00, 14.24 ppm.

HRMS (ESI^+): calcd for $C_{22}H_{20}{}^{35}ClN_2O^+$ $[M+H]^+$ 363.1259, found 363.1256; $C_{22}H_{20}{}^{37}ClN_2O^+$ $[M+H]^+$ 365.1229, found 365.1223.

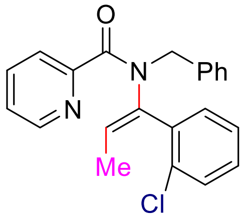

(*E*)-*N*-benzyl-*N*-(1-(2-chlorophenyl)prop-1-en-1-yl)picolinamide (**3j**)

Purification via column chromatography on silica gel (ethyl acetate/petroleum ether = 1/6, v/v) afforded **3j** as pale yellow oil (21.1 mg, 58% yield, Z/E = 1:9).

^{1}**H NMR** (400 MHz, $CDCl_3$): δ = 8.52 (d, J = 4.8 Hz, 1H), 8.02 (brs, 1H), 7.67 (td, J = 7.7, 1.6 Hz, 1H), 7.58 (d, J = 7.8 Hz, 1H), 7.42–7.14 (m, 8H), 6.95–6.74 (m, 1H), 5.33–5.32 (m, 1H), 4.71 (brs, 2H), 1.26 (brs, 3H) ppm.

^{13}**C NMR** (100 MHz, $CDCl_3$): δ = 169.43, 155.31, 148.06, 137.84, 136.58, 134.67, 134.34, 134.22, 132.78, 129.59, 128.39, 128.36, 128.33, 128.31, 127.06, 126.79, 124.03, 123.70, 50.94, 14.42 ppm.

HRMS (ESI$^+$): calcd for $C_{22}H_{20}{}^{35}ClN_2O^+$ [M+H]$^+$ 363.1259, found 363.1257; $C_{22}H_{20}{}^{37}ClN_2O^+$ [M+H]$^+$ 365.1229, found 365.1225.

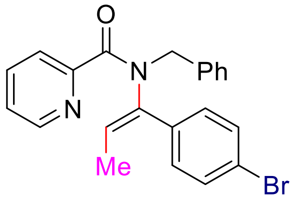

(*E*)-*N*-benzyl-*N*-(1-(4-bromophenyl)prop-1-en-1-yl)picolinamide (**3k**)

Purification via column chromatography on silica gel (ethyl acetate/petroleum ether = 1/6, v/v) afforded **3k** as pale yellow oil (25.6 mg, 63% yield, Z/E = 1:14).

^{1}**H NMR** (400 MHz, $CDCl_3$): δ = 8.45 (d, J = 4.5 Hz, 1H), 7.67 (td, J = 7.7, 1.4 Hz, 1H), 7.61 (d, J = 7.7 Hz, 1H), 7.51–7.46 (m, 2H), 7.39 (d, J = 8.4 Hz, 2H), 7.31–7.17 (m, 6H), 5.07 (q, J = 7.3 Hz, 1H), 4.66 (brs, 2H), 1.34 (d, J = 7.3 Hz, 3H) ppm.

^{13}**C NMR** (100 MHz, $CDCl_3$): δ = 169.55, 154.96, 148.17, 139.14, 137.25, 136.46, 134.24, 131.79, 131.50, 129.06, 128.44, 127.42, 126.28, 124.10, 123.57, 122.37, 49.80, 14.24 ppm.

HRMS (ESI$^+$): calcd for $C_{22}H_{20}{}^{79}BrN_2O^+$ [M+H]$^+$ 407.0754, found 407.0753; $C_{22}H_{20}{}^{81}BrN_2O^+$ [M+H]$^+$ 09.0733, found 09.0730.

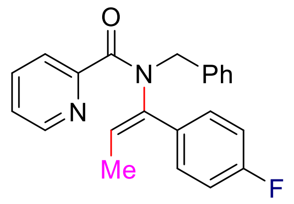

(*E*)-*N*-benzyl-*N*-(1-(4-fluorophenyl)prop-1-en-1-yl)picolinamide (**3l**)

Purification via column chromatography on silica gel (ethyl acetate/petroleum ether = 1/6, v/v) afforded **3l** as pale yellow oil (18.8 mg, 54% yield, Z/E = 1:23).

^{1}**H NMR** (400 MHz, $CDCl_3$): δ = 8.47 (d, *J* = 4.6 Hz, 1H), 7.67 (t, *J* = 7.6 Hz, 1H), 7.60 (d, *J* = 7.7 Hz, 1H), 7.49 (dd, *J* = 8.2, 5.7 Hz, 2H), 7.33–7.16 (m, 6H), 7.04 (t, *J* = 8.6 Hz, 2H), 5.06 (q, *J* = 7.2 Hz, 1H), 4.66 (brs, 2H), 1.34 (d, *J* = 7.2 Hz, 3H) ppm.

^{13}**C NMR** (100 MHz, $CDCl_3$): δ = 169.58, 162.53 (d, *J* = 248.1 Hz), 155.10, 148.20, 139.13, 137.32, 136.44, 131.91 (d, *J* = 8.0 Hz), 131.24 (d, *J* = 3.0 Hz), 129.04, 128.42, 127.38, 125.64, 124.04, 123.49, 115.26 (d, *J* = 21.4 Hz), 49.77, 14.21 ppm.

^{19}**F NMR** (376 MHz, $CDCl_3$) δ = -113.17 ppm.

HRMS (ESI^+): calcd for $C_{22}H_{20}FN_2O^+$ $[M+H]^+$ 347.1554, found 347.1550.

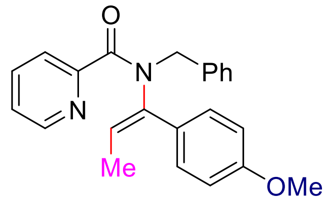

(*E*)-*N*-benzyl-*N*-(1-(4-methoxyphenyl)prop-1-en-1-yl)picolinamide (**3m**)

Purification via column chromatography on silica gel (ethyl acetate/petroleum ether = 1/5, v/v) afforded **3m** as yellow oil (20.3 mg, 57% yield, Z/E = 1:4).

^{1}**H NMR** (400 MHz, $CDCl_3$): δ = 8.48 (d, *J* = 4.6 Hz, 1H), 7.67–7.61 (m, 1H), 7.57 (d, *J* = 8.4 Hz, 1H), 7.43 (d, *J* = 8.6 Hz, 2H), 7.30–7.24 (m, 5H), 7.20–7.17 (m, 1H), 6.90 (d, *J* = 8.6 Hz, 2H), 5.00 (q, *J* = 7.2 Hz, 1H), 4.67 (brs, 2H), 3.84 (s, 3H), 1.35 (d, *J* = 7.2 Hz, 3H) ppm.

^{13}**C NMR** (100 MHz, $CDCl_3$): δ = 169.65, 159.50, 155.36, 148.35, 139.43, 137.48, 136.26, 131.31, 130.25, 129.12, 128.36, 127.29, 124.94, 123.83, 123.14, 113.71, 55.42, 49.62, 14.27 ppm.

HRMS (ESI^+): calcd for $C_{23}H_{23}N_2O_2^+$ $[M+H]^+$ 359.1754, found 359.1750.

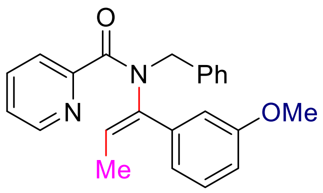

(*E*)-*N*-benzyl-*N*-(1-(3-methoxyphenyl)prop-1-en-1-yl)picolinamide (**3n**)

Purification via column chromatography on silica gel (ethyl acetate/petroleum ether = 1/5, v/v) afforded **3n** as yellow oil (19.0 mg, 53% yield, Z/E = 1:9).

^{1}**H NMR** (400 MHz, $CDCl_3$): δ = 8.49 (d, J = 4.3 Hz, 1H), 7.64 (t, J = 7.6 Hz, 1H), 7.56 (d, J = 7.7 Hz, 1H), 7.34–7.21 (m, 6H), 7.21–7.15 (m, 1H), 7.12 (s, 1H), 7.01 (d, J = 7.6 Hz, 1H), 6.86 (dd, J = 8.2, 2.6 Hz, 1H), 5.10 (q, J = 7.2 Hz, 1H), 4.69 (brs, 2H), 3.79 (s, 3H), 1.38 (d, J = 7.2 Hz, 3H) ppm.

^{13}**C NMR** (100 MHz, $CDCl_3$): δ = 169.61, 159.60, 155.22, 148.30, 139.69, 137.45, 136.69, 136.30, 129.18, 129.15, 128.38, 127.33, 125.95, 123.88, 123.27, 122.55, 115.17, 114.11, 55.39, 49.89, 14.31 ppm.

HRMS (ESI$^+$): calcd for $C_{23}H_{23}N_2O_2^+$ $[M+H]^+$ 359.1754, found 359.1748.

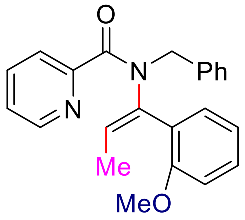

(*E*)-*N*-benzyl-*N*-(1-(2-methoxyphenyl)prop-1-en-1-yl)picolinamide (**3o**)

Purification via column chromatography on silica gel (ethyl acetate/petroleum ether = 1/5, v/v) afforded **3o** as yellow oil (22.1 mg, 62% yield, Z/E = 1:7).

^{1}**H NMR** (400 MHz, $CDCl_3$): δ = 8.53 (d, J = 4.3 Hz, 1H), 7.57 (t, J = 7.7 Hz, 1H), 7.45 (d, J = 7.7 Hz, 1H), 7.40–7.11 (m, 8H), 6.84 (t, J = 7.4 Hz, 2H), 5.31 (q, J = 7.0, 1H), 4.75 (brs, 2H), 3.77 (s, 3H), 1.26 (d, J = 7.0 Hz, 3H) ppm.

^{13}**C NMR** (100 MHz, $CDCl_3$): δ = 169.48, 157.26, 155.71, 148.35, 138.07, 136.77, 136.08, 132.04, 129.70, 128.63, 128.26, 126.98, 125.73, 124.21, 123.54, 123.08, 120.37, 110.65, 55.26, 51.12, 14.31 ppm.

HRMS (ESI$^+$): calcd for $C_{23}H_{23}N_2O_2^+$ $[M+H]^+$ 359.1754, found 359.1749.

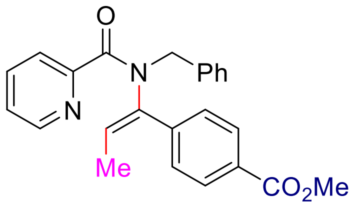

Methyl (*E*)-4-(1-(*N*-benzylpicolinamido)prop-1-en-1-yl)benzoate (**3p**)

Purification via column chromatography on silica gel (ethyl acetate/petroleum ether = 1/5, v/v) afforded **3p** as yellow oil (25.1 mg, 65% yield, Z/E = 1:9).

^{1}**H NMR** (400 MHz, $CDCl_3$): δ = 8.45 (d, J = 4.4 Hz, 1H), 8.01 (d, J = 8.4 Hz, 2H), 7.70–7.50 (m, 4H), 7.35–7.14 (m, 6H), 5.16 (q, J = 7.2 Hz, 1H), 4.67 (brs, 2H), 3.93 (s, 3H), 1.38 (d, J = 7.3 Hz, 3H) ppm.

^{13}**C NMR** (100 MHz, $CDCl_3$): δ = 169.53, 166.88, 154.81, 148.18, 140.06, 139.35, 137.14, 136.45, 129.99, 129.72, 129.50, 129.01, 128.43, 127.43, 127.07, 124.12, 123.55, 52.28, 50.05, 14.30 ppm.

HRMS (ESI^+): calcd for $C_{24}H_{23}N_2O_3^+$ $[M+H]^+$ 387.1703, found 387.1702.

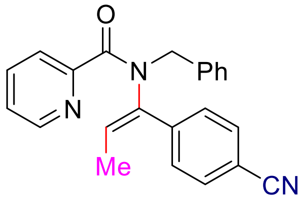

(*E*)-*N*-benzyl-*N*-(1-(4-cyanophenyl)prop-1-en-1-yl)picolinamide (**3q**)

Purification via column chromatography on silica gel (ethyl acetate/petroleum ether = 1/5, v/v) afforded **3q** as pale yellow oil (20.0 mg, 56% yield, Z/E = 1:20).

^{1}**H NMR** (400 MHz, $CDCl_3$): δ = 8.43 (d, J = 4.4 Hz, 1H), 7.74–7.56 (m, 6H), 7.33–7.15 (m, 6H), 5.18 (q, J = 7.3 Hz, 1H), 4.65 (brs, 2H), 1.37 (d, J = 7.3 Hz, 3H) ppm.

^{13}**C NMR** (100 MHz, $CDCl_3$): δ = 169.45, 154.47, 148.07, 140.25, 138.94, 136.94, 136.65, 132.03, 130.78, 128.98, 128.51, 127.76, 127.57, 124.37, 123.88, 118.86, 111.73, 50.13, 14.30 ppm.

HRMS (ESI^+): calcd for $C_{23}H_{20}N_3O^+$ $[M+H]^+$ 354.1601, found 354.1598.

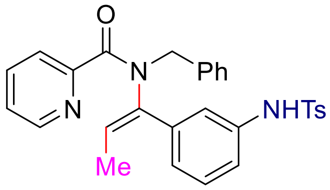

(*E*)-*N*-benzyl-*N*-(1-(3-((4-methylphenyl)sulfonamido)phenyl)prop-1-en-1-yl)picolinamide (**3r**)

Purification via column chromatography on silica gel (ethyl acetate/petroleum ether = 1/3, v/v) afforded **3r** as a pale yellow solid (32.1 mg, 64% yield, Z/E = 1:6). M.p.: 163–165 °C.

^{1}**H NMR** (400 MHz, $CDCl_3$): δ = 8.45 (d, J = 4.5 Hz, 1H), 7.84 (d, J = 7.9 Hz, 3H), 7.77–7.61 (m, 3H), 7.39 (t, J = 7.9 Hz, 1H), 7.35–7.19 (m, 9H), 7.07 (d, J = 7.8 Hz, 1H), 4.98 (q, J = 7.2 Hz, 1H), 4.55 (s, 2H), 2.42 (s, 5H), 1.25 (d, J = 7.1 Hz, 3H) ppm.

^{13}**C NMR** (100 MHz, $CDCl_3$): δ = 169.40, 154.69, 148.17, 145.21, 138.60, 137.12, 136.79, 136.49, 134.38, 133.67, 131.77, 131.31, 130.19, 129.77, 129.17, 129.12, 128.63, 128.46, 127.44, 127.06, 124.17, 123.56, 49.53, 21.80, 14.17 ppm.

HRMS (ESI^+): calcd for $C_{29}H_{28}N_3O_3S^+$ $[M+H]^+$ 498.1846, found 498.1843.

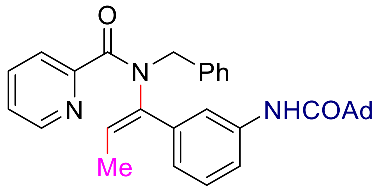

***N*-((*E*)-1-(3-((3*r*,5*r*,7*r*)-adamantane-1-carboxamido)phenyl)prop-1-en-1-yl)-*N*-benzylpicolinamide** (**3s**)

Purification via column chromatography on silica gel (ethyl acetate/petroleum ether = 1/4, v/v) afforded **3s** as a pale yellow solid (27.4 mg, 54% yield, Z/E = 1:5). M.p.: 147–150 °C.

^{1}**H NMR** (400 MHz, $CDCl_3$): δ = 8.48 (d, J = 4.5 Hz, 1H), 7.74 – 7.68 (m, 1H), 7.65 (t, J = 7.7 Hz, 1H), 7.57 (t, J = 8.0 Hz, 1H), 7.49 (s, 1H), 7.41–7.24 (m, 7H), 7.21–7.16 (m, 1H), 7.11 (d, J = 7.6 Hz, 1H), 5.10 (q, J = 7.2 Hz, 1H), 4.67 (brs, 2H), 2.11 (brs, 3H), 1.98 (brs, 6H), 1.76 (brs, 6H), 1.39 (d, J = 7.2 Hz, 3H) ppm.

^{13}**C NMR** (100 MHz, $CDCl_3$): δ = 176.31, 169.59, 155.10, 148.36, 139.35, 138.31, 137.38, 136.39, 135.83, 130.29, 129.14, 128.81, 128.39, 127.35, 126.52, 125.87, 123.92, 123.24, 121.05, 120.18, 49.92, 41.64, 39.37, 36.54, 28.25, 14.35 ppm.

HRMS (ESI^+): calcd for $C_{33}H_{36}N_3O_2^+$ $[M+H]^+$ 506.2802, found 506.2800.

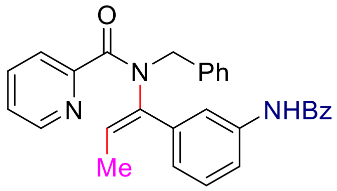

(*E*)-*N*-(1-(3-benzamidophenyl)prop-1-en-1-yl)-*N*-benzylpicolinamide (**3t**)

Purification via column chromatography on silica gel (ethyl acetate/petroleum ether = 1/3, v/v)

afforded **3t** as a pale yellow solid (29.0 mg, 65% yield, Z/E = 1:5). M.p.: 164–167 °C.

^{1}**H NMR** (400 MHz, $CDCl_3$): δ = 8.68 (brs, 1H), 8.45 (d, J = 4.5 Hz, 1H), 7.94 (t, J = 8.0 Hz, 3H), 7.75 (s, 1H), 7.60 (t, J = 7.6 Hz, 1H), 7.56–7.41 (m, 5H), 7.34 (t, J = 7.9 Hz, 2H), 7.28–7.18 (m, 6H), 7.15 (d, J = 5.4 Hz, 2H), 5.06 (q, J = 7.2 Hz, 1H), 4.63 (s, 2H), 1.39 (d, J = 7.3 Hz, 3H) ppm.

^{13}**C NMR** (100 MHz, $CDCl_3$): δ = 169.63, 166.19, 154.95, 148.42, 139.15, 138.70, 137.26, 136.34, 135.63, 135.04, 131.88, 129.02, 129.89, 128.74, 128.39, 127.44, 127.36, 126.88, 126.12, 123.92, 123.04, 121.30, 120.50, 49.68, 14.33 ppm.

HRMS (ESI^+): calcd for $C_{29}H_{26}N_3O_2^+$ $[M+H]^+$ 448.2020, found 448.2018.

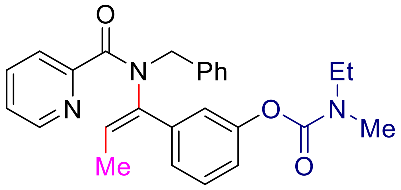

(*E*)-3-(1-(*N*-benzylpicolinamido)prop-1-en-1-yl)phenyl ethyl(methyl)carbamate (**3u**)

Purification via column chromatography on silica gel (ethyl acetate/petroleum ether = 1/3, v/v) afforded **3u** as yellow oil (23.6 mg, 55% yield, Z/E = 1:4).

^{1}**H NMR** (400 MHz, $CDCl_3$): δ = 8.49 (d, J = 4.3 Hz, 1H), 7.67–7.61 (m, 1H), 7.57 (d, J = 7.7 Hz, 1H), 7.40–7.21 (m, 8H), 7.21–7.15 (m, 1H), 7.10 (m, 1H), 5.09 (q J = 7.2 Hz, 1H), 4.69 (brs, 2H), 3.57–3.35 (m, 2H), 3.04 (d, J = 33.0 Hz, 3H), 1.38 (d, J = 7.3 Hz, 3H), 1.30–1.20 (m, 3H) ppm.

^{13}**C NMR** (100 MHz, $CDCl_3$): δ = 169.64, 162.00, 155.08, 151.63, 148.35, 139.17, 137.38, 136.36, 130.29, 129.12, 128.94, 128.38, 128.24, 127.30, 126.77, 126.55, 123.92, 123.24, 121.76, 49.85, 44.24, 34.40, 14.30, 13.39 ppm.

HRMS (ESI^+): calcd for $C_{26}H_{28}N_3O_3^+$ $[M+H]^+$ 430.2125, found 430.2124.

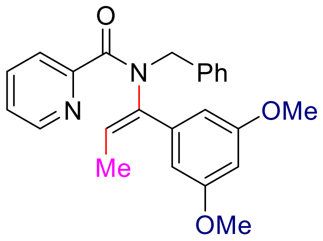

(*E*)-*N*-benzyl-*N*-(1-(3,5-dimethoxyphenyl)prop-1-en-1-yl)picolinamide (**3v**)

Purification via column chromatography on silica gel (ethyl acetate/petroleum ether = 1/3, v/v)

afforded **3v** as yellow oil (21.3 mg, 55% yield, Z/E = 1:11).

^{1}**H NMR** (400 MHz, $CDCl_3$): δ = 8.49 (d, J = 4.2 Hz, 1H), 7.64 (t, J = 7.7 Hz, 1H), 7.55 (d, J = 7.7 Hz, 1H), 7.34–7.23 (m, 5H), 7.20–7.17 (m, 1H), 6.64 (d, J = 1.9 Hz, 2H), 6.41 (t, J = 2.0 Hz, 1H), 5.10 (q, J = 7.2 Hz, 1H), 4.70 (brs, 2H), 3.76 (s, 6H), 1.39 (d, J = 7.2 Hz, 3H) ppm.

^{13}**C NMR** (100 MHz, $CDCl_3$): δ = 169.59, 160.59, 155.19, 148.26, 139.69, 137.46, 137.35, 136.31, 129.20, 128.38, 127.35, 125.96, 123.89, 123.32, 108.00, 100.49, 55.52, 49.97, 14.34 ppm.

HRMS (ESI^+): calcd for $C_{24}H_{25}N_2O_3^+$ $[M+H]^+$ 389.1860, found 389.1856.

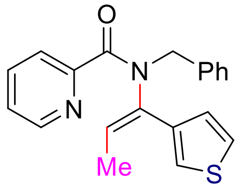

(*E*)-*N*-benzyl-*N*-(1-(thiophen-3-yl)prop-1-en-1-yl)picolinamide (**3w**)

Purification via column chromatography on silica gel (ethyl acetate/petroleum ether = 1/5, v/v) afforded **3w** as yellow oil (17.1 mg, 51% yield, Z/E = 1:10).

^{1}**H NMR** (400 MHz, $CDCl_3$): δ = 8.48 (d, J = 4.3 Hz, 1H), 7.63 (t, J = 7.7 Hz, 1H), 7.52 (d, J = 7.8 Hz, 1H), 7.45–7.21 (m, 7H), 7.21–7.14 (m, 2H), 5.08 (q, J = 7.2 Hz, 1H), 4.73 (brs, 2H), 1.42 (d, J = 7.2 Hz, 3H) ppm.

^{13}**C NMR** (100 MHz, $CDCl_3$): δ = 169.42, 155.10, 148.40, 137.38, 136.95, 136.24, 135.40, 129.19, 128.70, 128.39, 127.38, 126.15, 125.79, 125.20, 123.90, 123.00, 50.09, 14.18 ppm.

HRMS (ESI^+): calcd for $C_{20}H_{19}N_2OS^+$ $[M+H]^+$ 335.1213, found 335.1211.

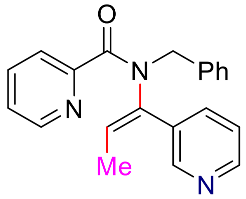

(*E*)-*N*-benzyl-*N*-(1-(pyridin-3-yl)prop-1-en-1-yl)picolinamide (**3x**)

Purification via column chromatography on silica gel (ethyl acetate/petroleum ether = 1/2, v/v) afforded **3x** as yellow oil (16.8 mg, 51% yield, Z/E = 1:9).

^{1}**H NMR** (400 MHz, $CDCl_3$): δ = 8.60 (s, 1H), 8.53 (d, J = 4.7 Hz, 1H), 8.45 (d, J = 4.3 Hz, 1H), 7.98 (d, J = 7.7 Hz, 1H), 7.71–7.65 (m, 2H), 7.34–7.16 (m, 7H), 5.16 (q, J = 7.3 Hz, 1H), 4.67 (brs, 2H), 1.37 (d, J =

7.2 Hz, 3H) ppm.

[13]**C NMR** (100 MHz, $CDCl_3$): δ = 169.48, 154.67, 151.24, 149.22, 148.11, 137.53, 137.45, 137.08, 136.59, 131.41, 129.05, 128.51, 127.54, 127.28, 124.29, 123.84, 123.41, 50.00, 14.17 ppm.

HRMS (ESI^+): calcd for $C_{21}H_{20}N_3O^+$ $[M+H]^+$ 330.1601, found 330.1597.

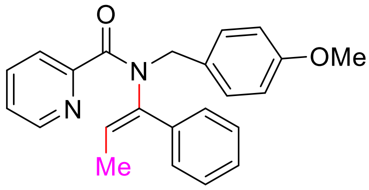

(*E*)-*N*-(4-methoxybenzyl)-*N*-(1-phenylprop-1-en-1-yl)picolinamide (**4a**)

Purification via column chromatography on silica gel (ethyl acetate/petroleum ether = 1/5, v/v) afforded **4a** as yellow oil (22.7 mg, 63% yield, Z/E = 1:5).

[1]**H NMR** (400 MHz, $CDCl_3$): δ = 8.48 (d, *J* = 4.6 Hz, 1H), 7.67–7.60 (m, 1H), 7.55 (d, *J* = 7.7 Hz, 1H), 7.49 (d, *J* = 7.1 Hz, 2H), 7.39–7.27 (m, 3H), 7.22 (d, *J* = 8.5 Hz, 2H), 7.17 (dd, *J* = 6.8, 5.2 Hz, 1H), 6.82 (d, *J* = 8.6 Hz, 2H), 5.06 (q, *J* = 7.2 Hz, 1H), 4.61 (brs, 2H), 3.79 (s, 3H), 1.38 (d, *J* = 7.3 Hz, 3H) ppm.

[13]**C NMR** (100 MHz, $CDCl_3$): δ = 169.55, 158.88, 155.35, 148.33, 139.75, 136.25, 135.27, 130.50, 130.01, 129.65, 128.30, 125.89, 123.81, 123.18, 114.21, 113.71, 55.32, 49.12, 14.29 ppm.

HRMS (ESI^+): calcd for $C_{23}H_{23}N_2O_2^+$ $[M+H]^+$ 359.1754, found 359.1750.

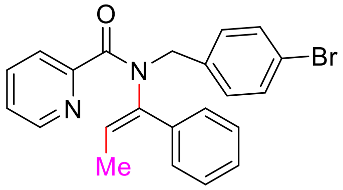

(*E*)-*N*-(4-bromobenzyl)-*N*-(1-phenylprop-1-en-1-yl)picolinamide (**4b**)

Purification via column chromatography on silica gel (ethyl acetate/petroleum ether = 1/5, v/v) afforded **4b** as yellow oil (23.7 mg, 58% yield, Z/E = 1:17).

[1]**H NMR** (400 MHz, $CDCl_3$): δ = 8.49 (d, *J* = 4.6 Hz, 1H), 7.66 (td, *J* = 7.7, 1.5 Hz, 1H), 7.58 (d, *J* = 7.8 Hz, 1H), 7.50 (d, *J* = 7.0 Hz, 2H), 7.44–7.29 (m, 5H), 7.23–7.12 (m, 3H), 5.04 (q, *J* = 7.2 Hz, 1H), 4.60 (brs, 2H), 1.38 (d, *J* = 7.2 Hz, 3H) ppm.

[13]**C NMR** (100 MHz, $CDCl_3$): δ = 169.68, 154.97, 148.38, 139.79, 136.46, 136.37, 134.97, 131.50, 130.91, 130.02, 128.42, 125.98, 124.03, 123.30, 121.36, 49.14, 14.29 ppm.

HRMS (ESI$^+$): calcd for $C_{22}H_{20}{}^{35}BrN_2O^+$ [M+H]$^+$ 407.0754, found 407.0753; $C_{22}H_{20}{}^{37}BrN_2O^+$ [M+H]$^+$ 409.0733, found 409.0729.

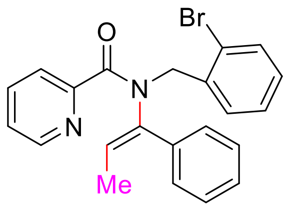

(*E*)-*N*-(2-bromobenzyl)-*N*-(1-phenylprop-1-en-1-yl)picolinamide (**4c**)

Purification via column chromatography on silica gel (ethyl acetate/petroleum ether = 1/5, v/v) afforded **4c** as yellow oil (23.3 mg, 57% yield, Z/E = 1:12).

^{1}**H NMR** (400 MHz, $CDCl_3$): δ = 8.51 (d, J = 4.4 Hz, 1H), 7.62 (t, J = 7.6 Hz, 1H), 7.50 (t, J = 8.4 Hz, 2H), 7.42 (d, J = 7.6 Hz, 1H), 7.37 (d, J = 7.5 Hz, 2H), 7.33–7.23 (m, 4H), 7.22–7.16 (m, 1H), 7.09 (t, J = 7.6 Hz, 1H), 5.36 (q, J = 14.2, 7.0 Hz, 1H), 4.91 (s, 2H), 1.42 (d, J = 7.1 Hz, 3H) ppm.

^{13}**C NMR** (100 MHz, $CDCl_3$): δ = 169.91, 155.19, 148.36, 140.51, 136.42, 136.33, 135.29, 132.76, 129.95, 129.79, 128.70, 128.19, 127.54, 127.22, 125.00, 123.96, 123.55, 123.16, 50.78, 14.30 ppm.

HRMS (ESI$^+$): calcd for $C_{22}H_{20}{}^{35}BrN_2O^+$ [M+H]$^+$ 407.0754, found 407.0753; $C_{22}H_{20}{}^{37}BrN_2O^+$ [M+H]$^+$ 409.0733, found 409.0728.

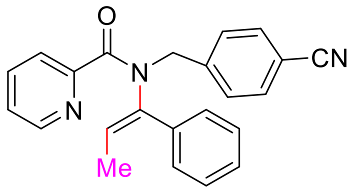

(*E*)-*N*-(4-cyanobenzyl)-*N*-(1-phenylprop-1-en-1-yl)picolinamide (**4d**)

Purification via column chromatography on silica gel (ethyl acetate/petroleum ether = 1/4, v/v) afforded **4d** as yellow oil (22.6 mg, 64% yield, Z/E = 1:8).

^{1}**H NMR** (400 MHz, $CDCl_3$): δ = 8.49 (d, J = 4.7 Hz, 1H), 7.68 (td, J = 7.7, 1.6 Hz, 1H), 7.61–7.55 (m, 3H), 7.50 (d, J = 6.8 Hz, 2H), 7.40–7.30 (m, 5H), 7.24–7.19 (m, 1H), 5.06 (q, J = 7.2 Hz, 1H), 4.68 (brs, 2H), 1.38 (d, J = 7.2 Hz, 3H) ppm.

^{13}**C NMR** (100 MHz, $CDCl_3$): δ = 169.78, 154.45, 148.38, 142.81, 139.91, 136.47, 134.62, 132.23, 129.91, 129.53, 128.56, 128.47, 125.84, 124.24, 123.33, 118.98, 111.12, 49.56, 14.26 ppm.

HRMS (ESI⁺): calcd for $C_{23}H_{20}N_3O^+$ [M+H]⁺ 354.1601, found 354.1598.

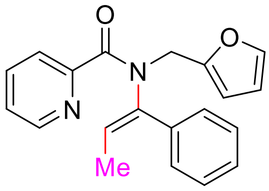

(*E*)-*N*-(furan-2-ylmethyl)-*N*-(1-phenylprop-1-en-1-yl)picolinamide (**4e**)

Purification via column chromatography on silica gel (ethyl acetate/petroleum ether = 1/5, v/v) afforded **4e** as yellow oil (21.7 mg, 68% yield, Z/E = 1:11).

^{1}**H NMR** (400 MHz, CD_3OD): δ = 8.44 (d, *J* = 4.5 Hz, 1H), 7.75 (t, *J* = 7.8 Hz, 1H), 7.49 (d, *J* = 7.8 Hz, 1H), 7.43 (brs, 1H), 7.37–7.25 (m, 6H), 6.35 (brs, 1H), 6.27 (d, *J* = 2.8 Hz, 1H), 5.37 (q, *J* = 7.2 Hz, 1H), 4.73 (brs, 2H), 1.43 (d, *J* = 7.3 Hz, 3H) ppm.

^{13}**C NMR** (100 MHz, CD_3OD): δ = 171.01, 155.83, 151.84, 149.36, 143.44, 140.84, 138.08, 136.22, 130.62, 129.36, 129.29, 127.06, 125.58, 124.20, 111.40, 110.19, 44.14, 14.39 ppm.

HRMS (ESI⁺): calcd for $C_{20}H_{19}N_2O_2^+$ [M+H]⁺ 319.1441, found 319.1440.

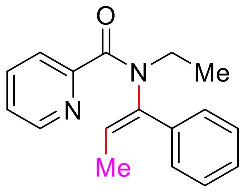

(*E*)-*N*-ethyl-*N*-(1-phenylprop-1-en-1-yl)picolinamide (**4f**)

Purification via column chromatography on silica gel (ethyl acetate/petroleum ether = 1/5, v/v) afforded **4f** as yellow oil (16.1 mg, 60% yield, Z/E = 1:5).

^{1}**H NMR** (400 MHz, $CDCl_3$): δ = 8.46 (d, *J* = 4.7 Hz, 1H), 7.66–7.50 (m, 4H), 7.39–7.27 (m, 3H), 7.18–7.15 (m, 1H), 5.26 (q, *J* = 7.2 Hz, 1H), 3.51 (dd, *J* = 13.3, 7.1 Hz, 2H), 1.47 (d, *J* = 7.3 Hz, 3H), 1.15 (t, *J* = 7.1 Hz, 3H) ppm.

^{13}**C NMR** (100 MHz, $CDCl_3$): δ = 169.21, 155.42, 148.31, 139.91, 136.24, 135.27, 129.81, 128.22, 127.06, 125.11, 123.73, 122.95, 41.05, 14.33, 12.52 ppm.

HRMS (ESI⁺): calcd for $C_{17}H_{19}N_2O^+$ [M+H]⁺ 267.1492, found 267.1491.

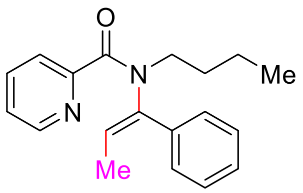

(*E*)-*N*-butyl-*N*-(1-phenylprop-1-en-1-yl)picolinamide (**4g**)

Purification via column chromatography on silica gel (ethyl acetate/petroleum ether = 1/5, v/v) afforded **4g** as yellow oil (17.8 mg, 60% yield, Z/E = 1:4).

^{1}**H NMR** (400 MHz, $CDCl_3$): δ = 8.46 (d, J = 4.7 Hz, 1H), 7.66–7.50 (m, 4H), 7.39–7.27 (m, 3H), 7.18–7.14 (m, 1H), 5.25 (q, J = 7.2 Hz, 1H), 3.43 (m, 2H), 1.60–1.52 (m, 2H), 1.45 (d, J = 7.2 Hz, 3H), 1.34–1.23 (m, 2H), 0.87 (t, J = 7.3 Hz, 3H) ppm.

^{13}**C NMR** (100 MHz, $CDCl_3$): δ = 169.39, 155.56, 148.28, 140.19, 136.23, 135.21, 129.82, 128.22, 127.12, 125.03, 123.66, 122.95, 45.78, 29.55, 20.20, 14.30, 13.98 ppm.

HRMS (ESI^+): calcd for $C_{19}H_{23}N_2O^+$ $[M+H]^+$ 295.1805, found 295.1803.

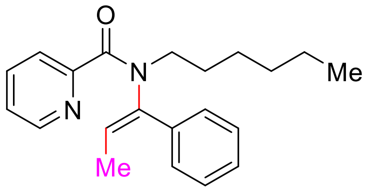

(*E*)-*N*-hexyl-*N*-(1-phenylprop-1-en-1-yl)picolinamide (**4h**)

Purification via column chromatography on silica gel (ethyl acetate/petroleum ether = 1/5, v/v) afforded **4h** as yellow oil (20.0 mg, 60% yield, Z/E = 1:5).

^{1}**H NMR** (400 MHz, $CDCl_3$): δ = 8.46 (d, J = 4.5 Hz, 1H), 7.67–7.49 (m, 4H), 7.40–7.27 (m, 3H), 7.16 (dd, J = 6.5, 5.2 Hz, 1H), 5.25 (q, J = 7.2 Hz, 1H), 3.43 (m, 2H), 1.59–1.54 (m, 2H), 1.46 (d, J = 7.3 Hz, 3H), 1.28–1.25 (m, 6H), 0.91–0.77 (m, 3H) ppm.

^{13}**C NMR** (100 MHz, $CDCl_3$): δ = 169.37, 155.61, 148.29, 140.27, 136.23, 135.27, 129.85, 128.23, 127.16, 124.99, 123.66, 122.99, 46.08, 31.63, 27.36, 26.62, 22.63, 14.30, 14.12 ppm.

HRMS (ESI^+): calcd for $C_{21}H_{27}N_2O^+$ $[M+H]^+$ 323.2118, found 323.2115.

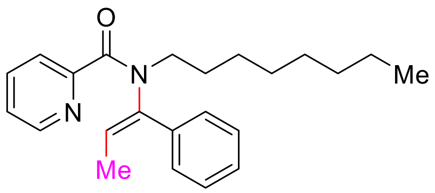

(*E*)-*N*-octyl-*N*-(1-phenylprop-1-en-1-yl)picolinamide (**4i**)

Purification via column chromatography on silica gel (ethyl acetate/petroleum ether = 1/6, v/v) afforded **4i** as yellow oil (20.3 mg, 58% yield, Z/E = 1:5).

^{1}**H NMR** (400 MHz, $CDCl_3$): δ = 8.46 (d, J = 4.5 Hz, 1H), 7.66–7.51 (m, 4H), 7.36 (t, J = 7.5 Hz, 2H), 7.30 (t, J = 7.5 Hz, 1H), 7.20–7.13 (m, 1H), 5.25 (q, J = 7.2 Hz, 1H), 3.42 (t, J = 6.6 Hz, 2H), 1.60–1.54 (m, 2H), 1.46 (d, J = 7.3 Hz, 3H), 1.28–1.24 (m, 10H), 0.86 (t, J = 8.4, 3H) ppm.

^{13}**C NMR** (100 MHz, $CDCl_3$): δ = 169.36, 155.62, 148.27, 140.29, 136.22, 135.28, 129.85, 128.22, 127.16, 124.96, 123.65, 122.99, 46.08, 31.89, 29.38, 29.26, 27.39, 26.94, 22.73, 14.29, 14.19 ppm.

HRMS (ESI$^+$): calcd for $C_{23}H_{31}N_2O^+$ [M+H]$^+$ 351.2431, found 351.2427.

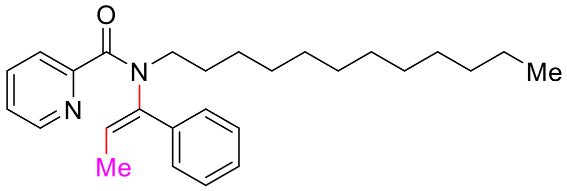

(*E*)-*N*-dodecyl-*N*-(1-phenylprop-1-en-1-yl)picolinamide (**4j**)

Purification via column chromatography on silica gel (ethyl acetate/petroleum ether = 1/6, v/v) afforded **4j** as yellow oil (25.2 mg, 62% yield, Z/E = 1:5).

^{1}**H NMR** (400 MHz, $CDCl_3$): δ = 8.46 (d, J = 4.7 Hz, 1H), 7.67–7.50 (m, 4H), 7.39–7.33 (m, 2H), 7.33–7.27 (m, 1H), 7.19–7.12 (m, 1H), 5.25 (q, J = 7.2 Hz, 1H), 3.43 (t, J = 7.2 Hz, 2H), 1.63–1.51 (m, 2H), 1.46 (d, J = 7.3 Hz, 3H), 1.24 (s, 18H), 0.87 (t, J = 6.8 Hz, 3H) ppm.

^{13}**C NMR** (100 MHz, $CDCl_3$): δ = 169.38, 155.65, 148.30, 140.32, 136.24, 135.32, 129.89, 128.25, 127.19, 124.98, 123.67, 123.03, 46.12, 32.04, 29.77, 29.76, 29.75, 29.72, 29.64, 29.47, 27.43, 26.98, 22.81, 14.32, 14.24 ppm.

HRMS (ESI$^+$): calcd for $C_{27}H_{39}N_2O^+$ [M+H]$^+$ 407.3057, found 407.3056.

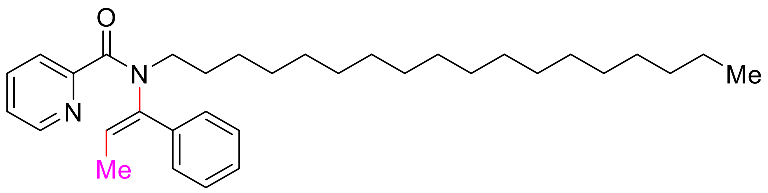

(*E*)-*N*-octadecyl-*N*-(1-phenylprop-1-en-1-yl)picolinamide (**4k**)

Purification via column chromatography on silica gel (ethyl acetate/petroleum ether = 1/6, v/v) afforded **4k** as yellow oil (24.6 mg, 50% yield, Z/E = 1:4).

^{1}**H NMR** (400 MHz, $CDCl_3$): δ = 8.47 (d, *J* = 4.5 Hz, 1H), 7.68–7.51 (m, 4H), 7.39–7.35 (m, 2H), 7.34–7.27 (m, 1H), 7.21–7.13 (m, 1H), 5.25 (q, *J* = 7.2 Hz, 1H), 3.42 (t, *J* = 7.2 Hz, 2H), 1.65–1.51 (m, 2H), 1.46 (d, *J* = 7.2 Hz, 3H), 1.33–1.14 (m, 30H), 0.88 (t, *J* = 6.8 Hz, 3H) ppm.

^{13}**C NMR** (100 MHz, $CDCl_3$): δ = 169.39, 155.66, 148.31, 140.33, 136.25, 135.33, 129.90, 128.25, 127.20, 124.98, 123.67, 123.04, 46.13, 32.06, 29.84, 29.79, 29.73, 29.65, 29.49, 29.48, 27.44, 26.99, 22.82, 14.32, 14.25 ppm.

HRMS (ESI$^+$): calcd for $C_{33}H_{51}N_2O^+$ $[M+H]^+$ 491.3996, found 491.3997.

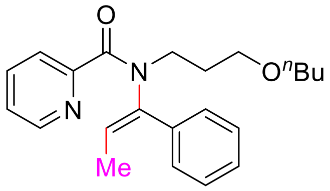

(*E*)-*N*-(3-butoxypropyl)-*N*-(1-phenylprop-1-en-1-yl)picolinamide (**4l**)

Purification via column chromatography on silica gel (ethyl acetate/petroleum ether = 1/3, v/v) afforded **4l** as yellow oil (19.0 mg, 54% yield, Z/E = 1:4).

^{1}**H NMR** (400 MHz, $CDCl_3$): δ = 8.46 (d, *J* = 4.5 Hz, 1H), 7.68–7.49 (m, 4H), 7.36 (t, *J* = 7.3 Hz, 2H), 7.31–7.28 (m, 1H), 7.20–7.12 (m, 1H), 5.26 (q, *J* = 7.2 Hz, 1H), 3.52 (t, *J* = 6.8 Hz, 2H), 3.43 (t, *J* = 6.8 Hz, 2H), 3.34 (t, *J* = 6.8 Hz, 2H), 2.08–1.74 (m, 4H), 1.54–1.38 (m, 5H), 1.34–1.20 (m, 4H), 0.88 (t, *J* = 7.3 Hz, 3H) ppm.

^{13}**C NMR** (100 MHz, $CDCl_3$): δ = 169.48, 155.41, 148.32, 140.28, 136.26, 135.15, 129.87, 128.26, 127.17, 125.15, 123.76, 123.01, 70.68, 68.54, 43.55, 31.91, 27.78, 19.43, 14.31, 14.05 ppm.

HRMS (ESI$^+$): calcd for $C_{22}H_{29}N_2O_2^+$ $[M+H]^+$ 353.2224, found 353.2221.

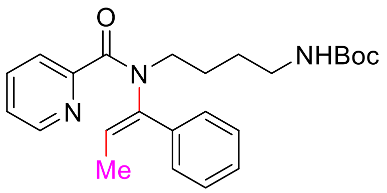

***tert*-Butyl (*E*)-(4-(*N*-(1-phenylprop-1-en-1-yl)picolinamido)butyl)carbamate** (**4m**)

Purification via column chromatography on silica gel (ethyl acetate/petroleum ether = 1/3, v/v) afforded **4m** as yellow oil (18.5 mg, 45% yield, Z/E = 1:4).

^{1}**H NMR** (400 MHz, $CDCl_3$): δ = 8.47 (d, *J* = 4.4 Hz, 1H), 7.69–7.48 (m, 4H), 7.37 (t, *J* = 7.3 Hz, 2H), 7.33–7.28 (m, 1H), 7.21–7.14 (m, 1H), 5.24 (q, *J* = 7.2 Hz, 1H), 4.63 (brs, 1H), 3.43 (t, *J* = 5.6 Hz, 2H), 3.10–3.08 (m, 2H), 1.66–1.54 (m, 2H), 1.45 (d, *J* = 7.2 Hz, 3H, overlap), 1.46–1.42 (m, 2H, overlap), 1.42 (s, 9H, overlap) ppm.

^{13}**C NMR** (100 MHz, $CDCl_3$): δ = 169.52, 156.08, 155.35, 148.35, 140.15, 136.30, 135.05, 129.85, 128.33, 127.16, 125.23, 123.81, 123.01, 45.55, 40.37, 30.30, 28.54, 27.33, 24.76, 14.31 ppm.

HRMS (ESI^+): calcd for $C_{24}H_{32}N_3O_3^+$ $[M+H]^+$ 410.2438, found 410.2438.

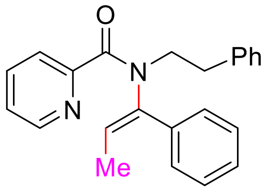

(*E*)-*N*-phenethyl-*N*-(1-phenylprop-1-en-1-yl)picolinamide (**4n**)

Purification via column chromatography on silica gel (ethyl acetate/petroleum ether = 1/5, v/v) afforded **4n** as yellow oil (23.4 mg, 68% yield, Z/E = 1:4).

^{1}**H NMR** (400 MHz, $CDCl_3$): δ = 8.49 (d, *J* = 4.7 Hz, 1H), 7.70–7.54 (m, 4H), 7.39 (t, *J* = 7.3 Hz, 2H), 7.35–7.30 (m, 1H), 7.28–7.13 (m, 6H), 5.20 (q, *J* = 7.2 Hz, 1H), 3.66 (t, *J* = 7.2 Hz, 2H), 2.94 (t, *J* = 7.6 Hz, 2H), 1.45 (d, *J* = 7.3 Hz, 3H) ppm.

^{13}**C NMR** (100 MHz, $CDCl_3$): δ = 169.40, 155.25, 148.34, 140.25, 139.17, 136.32, 135.16, 129.88, 128.99, 128.44, 128.33, 127.21, 126.29, 125.22, 123.86, 123.10, 47.82, 33.77, 14.33 ppm.

HRMS (ESI^+): calcd for $C_{23}H_{23}N_2O^+$ $[M+H]^+$ 343.1805, found 343.1802.

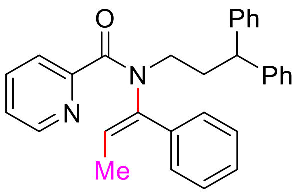

(*E*)-*N*-(3,3-diphenylpropyl)-*N*-(1-phenylprop-1-en-1-yl)picolinamide (**4o**)

Purification via column chromatography on silica gel (ethyl acetate/petroleum ether = 1/5, v/v) afforded **4o** as yellow oil (28.1 mg, 65% yield, Z/E = 1:4).

^{1}**H NMR** (400 MHz, $CDCl_3$): δ = 8.48 (dd, *J* = 4.8, 0.8 Hz, 1H), 7.65 (t, *J* = 7.6 Hz, 1H), 7.60–7.55 (m, 1H), 7.51 (d, *J* = 7.2 Hz, 2H), 7.38–7.32 (m, 3H), 7.27–7.09 (m, 11H), 5.21 (q, *J* = 7.2 Hz, 1H), 3.90 (t, *J* = 7.8 Hz, 1H), 3.42 (t, *J* = 7.6 Hz, 2H), 2.40 (q, *J* = 7.9 Hz, 2H), 1.45 (d, *J* = 7.1 Hz, 3H) ppm.

^{13}**C NMR** (100 MHz, $CDCl_3$): δ = 169.35, 155.31, 148.30, 144.33, 140.53, 136.28, 135.15, 129.96, 128.53, 128.25, 127.84, 127.34, 126.27, 124.84, 123.81, 123.07, 49.15, 45.63, 32.89, 14.28 ppm.

HRMS (ESI^+): calcd for $C_{30}H_{29}N_2O^+$ $[M+H]^+$ 433.2274, found 433.2273.

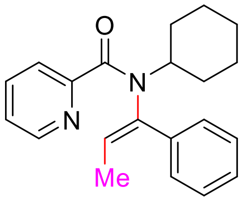

(*E*)-*N*-cyclohexyl-*N*-(1-phenylprop-1-en-1-yl)picolinamide (**4p**)

Purification via column chromatography on silica gel (ethyl acetate/petroleum ether = 1/5, v/v) afforded **4p** as yellow oil (17.5 mg, 54% yield, Z/E = 1:4).

^{1}**H NMR** (400 MHz, $CDCl_3$): δ = 8.42 (d, *J* = 4.4 Hz, 1H), 7.65–7.56 (m, 3H), 7.50 (d, *J* = 7.8 Hz, 1H), 7.38–7.25 (m, 3H), 7.15–7.09 (m, 1H), 5.32 (q, *J* = 7.2 Hz, 1H), 4.19 (t, *J* = 10.1 Hz, 1H), 1.90–1.66 (m, 6H), 1.47 (d, *J* = 7.3 Hz, 3H), 1.31–1.24 (m, 2H), 0.97–0.91 (m, 2H) ppm.

^{13}**C NMR** (100 MHz, $CDCl_3$): δ = 169.54, 156.43, 148.09, 139.17, 136.93, 136.14, 130.32, 127.97, 127.80, 127.73, 123.34, 122.78, 56.92, 30.88, 26.23, 25.57, 14.54 ppm.

HRMS (ESI^+): calcd for $C_{21}H_{25}N_2O^+$ $[M+H]^+$ 321.1961, found 321.1959.

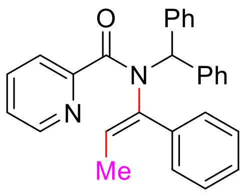

(*E*)-*N*-benzhydryl-*N*-(1-phenylprop-1-en-1-yl)picolinamide (**4q**)

Purification via column chromatography on silica gel (ethyl acetate/petroleum ether = 1/5, v/v) afforded **4q** as yellow oil (22.4 mg, 55% yield, Z/E = 1:8).

^{1}**H NMR** (400 MHz, $CDCl_3$): δ = 8.54–8.48 (m, 1H), 7.58 (td, *J* = 7.7, 1.7 Hz, 1H), 7.49 (d, *J* = 7.8 Hz, 1H), 7.45 (d, *J* = 7.0 Hz, 2H), 7.40 (d, *J* = 7.4 Hz, 4H), 7.35 (d, *J* = 4.4 Hz, 1H), 7.31 (t, *J* = 7.3 Hz, 4H), 7.27–7.20 (m, 4H), 7.17 (ddd, *J* = 7.5, 4.8, 1.2 Hz, 1H), 6.20 (brs, 1H), 5.42–5.26 (m, 1H), 1.38 (d, *J* = 7.2 Hz, 3H) ppm.

^{13}**C NMR** (100 MHz, $CDCl_3$): δ = 169.69, 155.73, 148.00, 139.45, 136.29, 130.16, 129.36, 128.79, 128.12, 128.02, 127.95, 127.61, 127.18, 125.62, 123.93, 123.72, 66.10, 14.40 ppm.

HRMS (ESI^+): calcd for $C_{28}H_{25}N_2O^+$ $[M+H]^+$ 405.1961, found 405.1960.

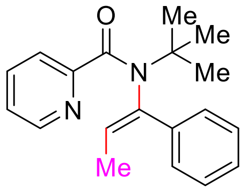

(*E*)-*N*-(*tert*-butyl)-*N*-(1-phenylprop-1-en-1-yl)picolinamide (**4r**)

Purification via column chromatography on silica gel (ethyl acetate/petroleum ether = 1/5, v/v) afforded **4r** as pale yellow oil (4.7 mg, 16% yield, Z/E < 1:20).

^{1}**H NMR** (400 MHz, $CDCl_3$): δ = 8.37 (d, *J* = 4.8 Hz, 1H), 7.62–7.53 (m, 3H), 7.40–7.31 (m, 3H), 7.28–7.24 (m, 1H), 7.07 (ddd, *J* = 7.6, 4.9, 1.1 Hz, 1H), 5.51 (q, *J* = 7.4 Hz, 1H), 1.53 (d, *J* = 7.4 Hz, 3H), 1.42 (s, 9H) ppm.

^{13}**C NMR** (100 MHz, $CDCl_3$): δ = 170.72, 157.81, 148.11, 139.78, 138.22, 135.95, 130.47, 129.86, 127.88, 127.72, 122.79, 121.70, 59.60, 28.57, 14.86 ppm.

HRMS (ESI^+): calcd for $C_{19}H_{23}N_2O^+$ $[M+H]^+$ 295.1805, found 295.1803.

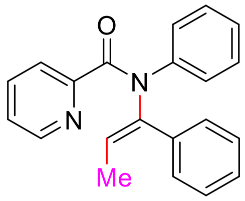

(*E*)-*N*-phenyl-*N*-(1-phenylprop-1-en-1-yl)picolinamide (**4s**)

Purification via column chromatography on silica gel (ethyl acetate/petroleum ether = 1/4, v/v) afforded **4s** as yellow oil (21.4 mg, 68% yield, Z/E = 1:8).

^{1}**H NMR** (400 MHz, acetone-d_6): δ = 8.47 (m, 1H), 7.84–7.82 (m, 1H), 7.78–7.58 (m, 3H), 7.53–7.12 (m, 8H), 7.07 (t, J = 7.2 Hz, 1H), 5.60 (m, 1H), 1.58 (m, 3H) ppm.

^{13}**C NMR** (100 MHz, acetone-d_6): δ = 169.81, 156.58, 148.95, 141.78, 138.48, 137.42, 136.40, 130.82, 129.22, 128.61, 128.37, 127.32, 126.51, 125.05, 124.45, 14.55 ppm.

HRMS (ESI$^+$): calcd for $C_{21}H_{19}N_2O^+$ [M+H]$^+$ 315.1492, found 315.1489.

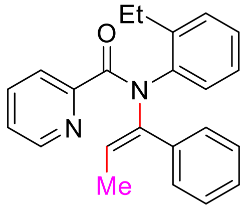

(*E*)-*N*-(2-ethylphenyl)-*N*-(1-phenylprop-1-en-1-yl)picolinamide (**4t**)

Purification via column chromatography on silica gel (ethyl acetate/petroleum ether = 1/6, v/v) afforded **4t** as pale yellow oil (22.4 mg, 65% yield, Z/E = 1:6).

^{1}**H NMR** (400 MHz, CD_3OD): δ = 8.58 (d, J = 3.0 Hz, 1H), 7.84 (t, J = 7.5 Hz, 1H), 7.68 (d, J = 7.7 Hz, 1H), 7.43–7.45 (m, 3H), 7.30–7.13 (m, 7H), 5.49 (q, J = 6.8 Hz, 1H), 2.66 (q, 7.5 Hz, 2H), 1.41 (d, J = 7.0 Hz, 3H), 1.13 (t, J = 7.4 Hz, 3H) ppm.

^{13}**C NMR** (100 MHz, CD_3OD): δ = 165.79, 149.53, 142.96, 142.58, 138.20, 137.82, 136.60, 131.03, 130.55, 129.87, 129.19, 129.09, 128.93, 128.43, 127.52, 126.41, 125.91, 124.78, 24.38, 14.28, 14.17 ppm.

HRMS (ESI$^+$): calcd for $C_{23}H_{23}N_2O^+$ [M+H]$^+$ 343.1805, found 343.1802.

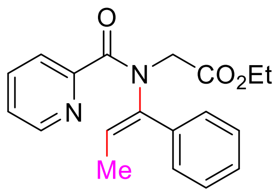

Ethyl (*E*)-*N*-(1-phenylprop-1-en-1-yl)-*N*-picolinoylglycinate (5a)

Purification via column chromatography on silica gel (ethyl acetate/petroleum ether = 1/5, v/v) afforded **5a** as yellow oil (22.7 mg, 70% yield, Z/E = 1:10).

^{1}**H NMR** (400 MHz, $CDCl_3$): δ = 8.47 (d, *J* = 4.8 Hz, 1H), 7.71–7.62 (m, 2H), 7.61–7.57 (m, 2H), 7.39–7.33 (m, 2H), 7.33–7.28 (m, 1H), 7.22–7.19 (m, 1H), 5.50 (q, *J* = 7.3 Hz, 1H), 4.20 (q, *J* = 7.1 Hz, 2H), 4.13 (brs, 2H), 1.50 (d, *J* = 7.3 Hz, 3H), 1.27 (t, *J* = 7.1 Hz, 3H) ppm.

^{13}**C NMR** (100 MHz, $CDCl_3$): δ = 169.84, 168.96, 154.23, 148.28, 140.52, 136.43, 134.90, 130.10, 128.42, 128.32, 125.31, 124.24, 123.67, 61.21, 48.77, 14.43, 14.29 ppm.

HRMS (ESI^+): calcd for $C_{19}H_{21}N_2O_3^+$ $[M+H]^+$ 325.1547, found 325.1544.

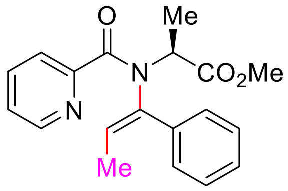

Methyl (*E*)-N-(1-phenylprop-1-en-1-yl)-*N*-picolinoyl-*L*-alaninate (5b)

Purification via column chromatography on silica gel (ethyl acetate/petroleum ether = 1/5, v/v) afforded **5b** as yellow oil (22.1 mg, 68% yield, Z/E = 1:10).

^{1}**H NMR** (400 MHz, $CDCl_3$): δ = 8.47 (d, *J* = 4.7 Hz, 1H), 7.71–7.58 (m, 4H), 7.39–7.35 (m, 2H), 7.34–7.27 (m, 1H), 7.21–7.16 (m, 1H), 5.39 (q, *J* = 7.2 Hz, 1H), 4.13 (q, *J* = 7.0 Hz, 1H), 3.71 (s, 3H), 1.54–1.46 (m, 6H) ppm.

^{13}**C NMR** (100 MHz, $CDCl_3$): δ = 171.68, 169.03, 154.59, 148.31, 140.65, 136.38, 135.11, 130.41, 128.44, 128.16, 125.80, 124.15, 123.39, 56.34, 52.30, 14.47, 14.39 ppm.

HRMS (ESI^+): calcd for $C_{19}H_{21}N_2O_3^+$ $[M+H]^+$ 325.1547, found 325.1546.

HPLC: (IC, *n*-hexane/*i*-PrOH = 70/30, flow rate: 1.0 mL/min, λ = 254 nm), t_{major} = 28.006 min, > 99% e.e. (racemic **5b**, t_1 = 26.165 min, t_2 = 28.392 min).

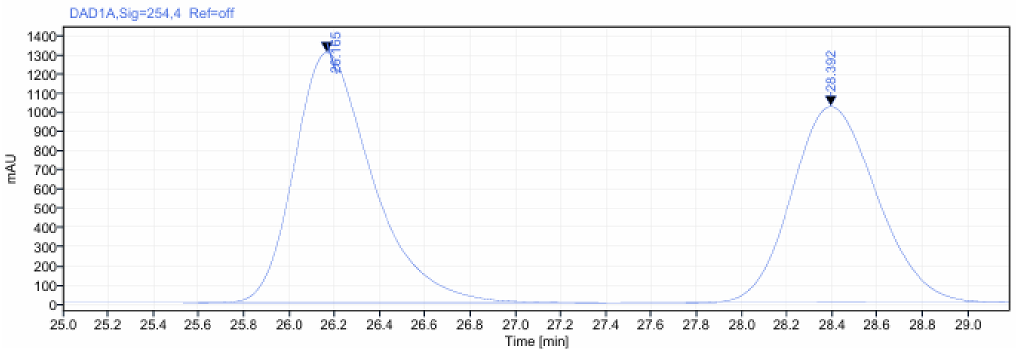

Signal: DAD1A,Sig=254,4 Ref=off

RT [min]	Type	Width [min]	Area	Height	Area%	Name
26.165	BB	1.80	31256.01	1304.68	53.99	
28.392	BB	1.41	26636.57	1022.30	46.01	
		**Sum**	**57892.58**			

HPLC of racemic **5b**

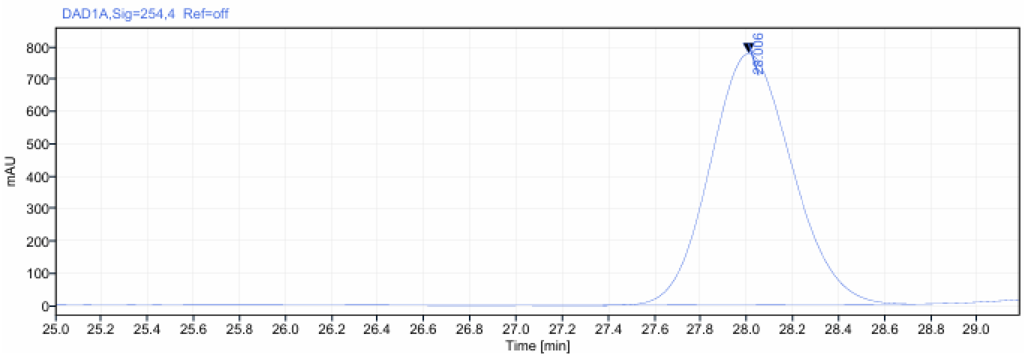

Signal: DAD1A,Sig=254,4 Ref=off

RT [min]	Type	Width [min]	Area	Height	Area%	Name
28.006	BB	1.36	19175.88	775.18	100.00	
		**Sum**	**19175.88**			

HPLC of chiral **5b**

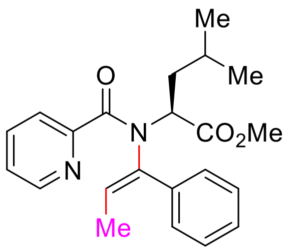

Methyl (*E*)-*N*-(1-phenylprop-1-en-1-yl)-*N*-picolinoyl-*L*-leucinate (5c)

Purification via column chromatography on silica gel (ethyl acetate/petroleum ether = 1/5, v/v) afforded **5c** as yellow oil (25.0 mg, 68% yield, Z/E = 1:13).

^{1}**H NMR** (400 MHz, $CDCl_3$): *δ* = 8.52–8.46 (m, 1H), 7.72–7.64 (m, 4H), 7.40–7.30 (m, 3H), 7.22–7.19 (m, 1H), 5.33 (q, *J* = 7.2 Hz, 1H), 4.20 (m, 1H), 3.66 (s, 3H), 2.14–2.04 (m, 1H), 1.73–1.62 (m, 2H), 1.43 (d, *J* = 7.2 Hz, 3H), 0.79 (d, *J* = 6.2 Hz, 3H), 0.72 (d, *J* = 6.2 Hz, 3H) ppm.

^{13}**C NMR** (100 MHz, $CDCl_3$): *δ* = 171.52, 169.39, 154.76, 148.29, 140.67, 136.41, 134.86, 130.71, 128.53, 128.12, 126.22, 124.10, 123.42, 57.96, 52.18, 38.61, 25.28, 22.48, 22.39, 14.44 ppm.

HRMS (ESI^+): calcd for $C_{22}H_{27}N_2O_3^+$ $[M+H]^+$ 367.2016, found 367.2012.

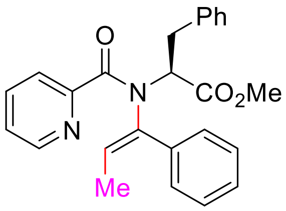

Methyl (*E*)-*N*-(1-phenylprop-1-en-1-yl)-*N*-picolinoyl-*L*-phenylalaninate (**5d**)

Purification via column chromatography on silica gel (ethyl acetate/petroleum ether = 1/5, v/v) afforded **5d** as yellow oil (29.9 mg, 75% yield, Z/E = 1:15).

^{1}**H NMR** (400 MHz, $CDCl_3$): *δ* = 8.48 (ddd, *J* = 4.8, 1.5, 1.0 Hz, 1H), 7.68 (dt, *J* = 7.6, 1.6 Hz, 1H), 7.63 (td, *J* = 7.6, 1.2 Hz, 1H), 7.54–7.51 (m, 2H), 7.37–7.29 (m, 3H), 7.24–7.15 (m, 4H), 7.10 (d, *J* = 6.9 Hz, 2H), 4.92 (q, *J* = 7.1 Hz, 1H), 4.28 (t, *J* = 7.1 Hz, 1H), 3.69 (s, 3H), 3.54 (dd, *J* = 14.1, 6.7 Hz, 1H), 3.34 (dd, *J* = 14.1, 7.7 Hz, 1H), 1.27 (d, *J* = 7.3 Hz, 3H) ppm.

^{13}**C NMR** (100 MHz, $CDCl_3$): *δ* = 170.64, 169.44, 154.57, 148.43, 140.50, 138.56, 136.37, 134.44, 130.61, 129.68, 128.40, 128.35, 128.13, 126.49, 126.15, 124.14, 123.28, 61.58, 52.35, 35.67, 14.37 ppm.

HRMS (ESI^+): calcd for $C_{26}H_{27}N_2O_3^+$ $[M+H]^+$ 415.2016, found 415.2012.

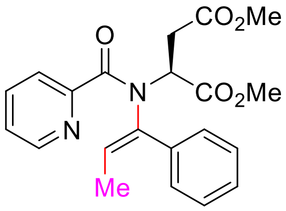

Dimethyl (*E*)-*N*-(1-phenylprop-1-en-1-yl)-*N*-picolinoyl-*L*-aspartate (**5e**)

Purification via column chromatography on silica gel (ethyl acetate/petroleum ether = 1/3, v/v) afforded **5e** as a pale yellow solid (30.8 mg, 80% yield, Z/E = 1:9). M.p.: 96–98 °C.

^{1}**H NMR** (400 MHz, $CDCl_3$): *δ* = 8.49 (d, *J* = 4.7 Hz, 1H), 7.73–7.61 (m, 4H), 7.39 (t, *J* = 7.6 Hz, 2H),

7.36–7.30 (m, 1H), 7.24–7.19 (m, 1H), 5.29 (q, J = 7.2 Hz, 1H), 4.57–4.48 (m, 1H), 3.70 (s, 3H), 3.63 (s, 3H), 3.41 (dd, J = 16.5, 8.0 Hz, 1H), 2.72 (dd, J = 16.5, 5.1 Hz, 1H), 1.47 (d, J = 7.3 Hz, 3H) ppm.

^{13}**C NMR** (100 MHz, $CDCl_3$): δ = 171.66, 170.09, 169.24, 153.97, 148.40, 140.82, 136.48, 134.37, 130.54, 128.70, 128.26, 125.71, 124.42, 123.52, 57.16, 52.65, 51.97, 34.32, 14.46 ppm.

HRMS (ESI$^+$): calcd for $C_{21}H_{23}N_2O_5^+$ [M+H]$^+$ 383.1601, found 383.1597.

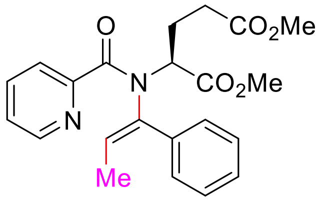

Dimethyl (*E*)-*N*-(1-phenylprop-1-en-1-yl)-*N*-picolinoyl-*L*-glutamate (**5f**)

Purification via column chromatography on silica gel (ethyl acetate/petroleum ether = 1/4, v/v) afforded **5f** as yellow oil (28.9 mg, 73% yield, Z/E = 1:12).

^{1}**H NMR** (400 MHz, $CDCl_3$): δ = 8.52–8.44 (m, 1H), 7.73–7.62 (m, 4H), 7.37 (t, J = 7.4 Hz, 2H), 7.34–7.28 (m, 1H), 7.24–7.17 (m, 1H), 5.35 (q, J = 7.2 Hz, 1H), 4.13 (t, J = 6.3 Hz, 1H), 3.67 (s, 3H), 3.57 (s, 3H), 2.50–2.41 (m, 3H), 2.30–2.16 (m, 1H), 1.41 (d, J = 7.2 Hz, 3H) ppm.

^{13}**C NMR** (100 MHz, $CDCl_3$): δ = 173.36, 170.63, 169.47, 154.44, 148.28, 140.79, 136.47, 134.63, 130.68, 128.58, 128.15, 126.31, 124.25, 123.50, 58.92, 52.25, 51.61, 31.03, 24.50, 14.42 ppm.

HRMS (ESI$^+$): calcd for $C_{22}H_{25}N_2O_5^+$ [M+H]$^+$ 397.1758, found 397.1756.

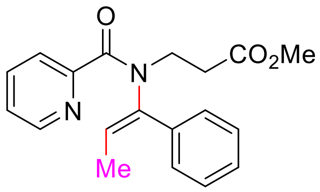

Methyl (*E*)-3-(*N*-(1-phenylprop-1-en-1-yl)picolinamido)propanoate (**5g**)

Purification via column chromatography on silica gel (ethyl acetate/petroleum ether = 1/5, v/v) afforded **5g** as yellow oil (22.8 mg, 70% yield, Z/E = 1:5).

^{1}**H NMR** (400 MHz, $CDCl_3$): δ = 8.47 (d, J = 4.5 Hz, 1H), 7.69–7.61 (m, 1H), 7.61–7.54 (m, 3H), 7.38 (t, J = 7.3 Hz, 2H), 7.35–7.29 (m, 1H), 7.22–7.16 (m, 1H), 5.24 (q, J = 7.3 Hz, 1H), 3.73 (t, J = 7.0 Hz, 2H), 3.64 (s, 3H), 2.66 (t, J = 7.5 Hz, 2H), 1.46 (d, J = 7.3 Hz, 3H) ppm.

^{13}**C NMR** (100 MHz, $CDCl_3$): δ = 172.21, 169.51, 154.81, 148.34, 140.06, 136.37, 134.83, 129.92, 128.47,

127.20, 125.57, 124.02, 123.20, 51.79, 42.28, 32.18, 14.38 ppm.

HRMS (ESI$^+$): calcd for $C_{19}H_{21}N_2O_3^+$ [M+H]$^+$ 325.1547, found 325.1545.

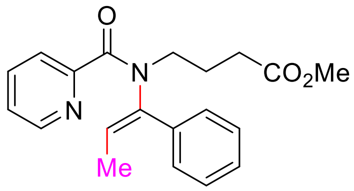

Methyl (*E*)-4-(*N*-(1-phenylprop-1-en-1-yl)picolinamido)butanoate (**5h**)

Purification via column chromatography on silica gel (ethyl acetate/petroleum ether = 1/5, v/v) afforded **5h** as yellow oil (24.5 mg, 72% yield, Z/E = 1:5).

^{1}**H NMR** (400 MHz, $CDCl_3$): δ = 8.47 (d, J = 4.7 Hz, 1H), 7.69–7.52 (m, 4H), 7.40–7.34 (m, 2H), 7.34–7.28 (m, 1H), 7.22–7.15 (m, 1H), 5.25 (q, J = 7.2 Hz, 1H), 3.63 (s, 3H), 3.47 (m, 2H), 2.34 (t, J = 7.7 Hz, 2H), 1.96–1.86 (m, 2H), 1.45 (d, J = 7.3 Hz, 3H) ppm.

^{13}**C NMR** (100 MHz, $CDCl_3$): δ = 173.66, 169.58, 155.22, 148.32, 140.05, 136.33, 134.97, 129.90, 128.33, 127.24, 125.39, 123.87, 123.12, 51.68, 45.06, 31.55, 22.86, 14.33 ppm.

HRMS (ESI$^+$): calcd for $C_{20}H_{23}N_2O_3^+$ [M+H]$^+$ 339.1703, found 339.1700.

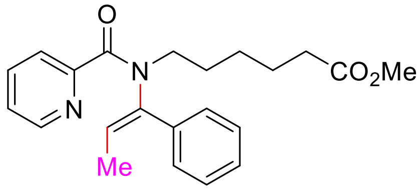

Methyl (*E*)-6-(*N*-(1-phenylprop-1-en-1-yl)picolinamido)hexanoate (**5i**)

Purification via column chromatography on silica gel (ethyl acetate/petroleum ether = 1/5, v/v) afforded **5i** as yellow oil (24.9 mg, 68% yield, Z/E = 1:5).

^{1}**H NMR** (400 MHz, $CDCl_3$): δ = 8.46 (d, J = 4.4 Hz, 1H), 7.67–7.48 (m, 4H), 7.40–7.33 (m, 2H), 7.32–7.28 (m, 1H), 7.20–7.14 (m, 1H), 5.24 (q, J = 7.2 Hz, 1H), 3.64 (s, 3H), 3.42 (m, 2H), 2.27 (t, J = 7.5 Hz, 2H), 1.67–1.52 (m, 4H), 1.45 (d, J = 7.3 Hz, 3H), 1.37–1.27 (m, 2H) ppm.

^{13}**C NMR** (100 MHz, $CDCl_3$): δ = 174.19, 169.42, 155.48, 148.30, 140.24, 136.26, 135.18, 129.85, 128.28, 127.16, 125.10, 123.73, 123.02, 51.54, 45.78, 34.03, 27.08, 26.46, 24.76, 14.29 ppm.

HRMS (ESI$^+$): calcd for $C_{22}H_{27}N_2O_3^+$ [M+H]$^+$ 367.2016, found 367.2014.

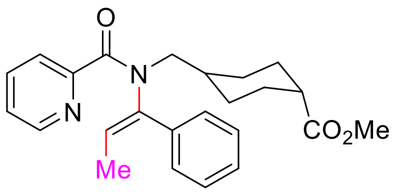

Methyl (*E*)-4-((*N*-(1-phenylprop-1-en-1-yl)picolinamido)methyl)cyclohexane-1-carboxylate (**5j**)

Purification via column chromatography on silica gel (ethyl acetate/petroleum ether = 1/5, v/v) afforded **5j** as yellow oil (24.8 mg, 63% yield, Z/E = 1:5).

^{1}**H NMR** (400 MHz, $CDCl_3$): δ = 8.48 (d, *J* = 4.6 Hz, 1H), 7.69–7.49 (m, 4H), 7.38 (t, *J* = 7.2 Hz, 2H), 7.34–7.28 (m, 1H), 7.21–7.13 (m, 1H), 5.25 (q, *J* = 7.2 Hz, 1H), 3.64 (s, 3H), 3.31 (brs, 2H), 2.22 (t, *J* = 12.2 Hz, 1H), 2.02–1.87 (m, 3H), 1.76 (d, *J* = 12.6 Hz, 2H), 1.70–1.50 (m, 2H), 1.42 (d, *J* = 7.1 Hz, 3H), 1.07–0.98 (m, 2H) ppm.

^{13}**C NMR** (100 MHz, $CDCl_3$): δ = 176.63, 169.91, 155.61, 148.37, 140.42, 136.28, 134.84, 129.82, 128.34, 127.38, 125.37, 123.67, 122.87, 51.61, 50.91, 43.22, 36.03, 29.85, 28.65, 14.28 ppm.

HRMS (ESI$^+$): calcd for $C_{24}H_{29}N_2O_3^+$ [M+H]$^+$ 393.2173, found 393.2169.

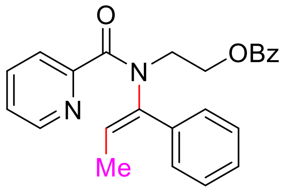

(*E*)-2-(*N*-(1-phenylprop-1-en-1-yl)picolinamido)ethyl benzoate (**5k**)

Purification via column chromatography on silica gel (ethyl acetate/petroleum ether = 1/4, v/v) afforded **5k** as yellow oil (23.9 mg, 62% yield, Z/E = 1:6).

^{1}**H NMR** (400 MHz, $CDCl_3$): δ = 8.47 (d, *J* = 4.7 Hz, 1H), 8.04 (d, *J* = 7.3 Hz, 2H), 7.70–7.63 (m, 1H), 7.60–7.51 (m, 4H), 7.44 (t, *J* = 7.7 Hz, 2H), 7.39–7.29 (m, 3H), 7.22–7.16 (m, 1H), 5.29 (q, *J* = 7.3 Hz, 1H), 4.55 (t, *J* = 5.7 Hz, 2H), 3.86 (brs, 2H), 1.37 (d, *J* = 7.3 Hz, 3H) ppm.

^{13}**C NMR** (100 MHz, $CDCl_3$): δ = 169.88, 166.49, 154.94, 148.34, 140.20, 136.38, 134.79, 133.10, 130.20, 129.92, 129.83, 128.46, 128.41, 127.30, 125.58, 123.99, 123.24, 62.36, 44.64, 14.28 ppm.

HRMS (ESI$^+$): calcd for $C_{24}H_{23}N_2O_3^+$ [M+H]$^+$ 387.1703, found 387.1699.

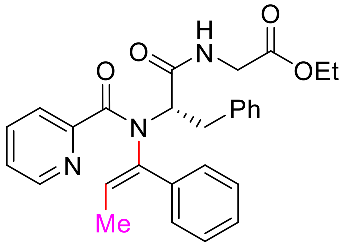

Ethyl (*E*)-(*E*)-*N*-(1-phenylprop-1-en-1-yl)-*N*-picolinoyl-*L*-phenylalanylglycinate (**6a**)

Purification via column chromatography on silica gel (MeOH/DCM = 1/5, v/v) afforded **6a** as yellow oil (21.7 mg, 46% yield, Z/E = 1:4).

^{1}**H NMR** (400 MHz, CD_3OD): δ = 8.44 (d, *J* = 3.2 Hz, 1H), 7.78 (t, *J* = 7.2 Hz, 1H), 7.55 (d, *J* = 7.7 Hz, 1H), 7.38–7.11 (m, 11H), 5.19 (q, *J* = 6.4 Hz, 1H), 4.70 (t, *J* = 7.3 Hz, 1H), 4.17–4.19 (m, 3H), 3.89 (brs, 2H), 3.48 – 3.33 (m, 2H), 1.30 (d, *J* = 5.4 Hz, 3H), 1.25 (t, *J* = 7.1 Hz, 3H) ppm.

^{13}**C NMR** (100 MHz, CD_3OD): δ = 172.18, 172.15, 171.08, 155.96, 149.41, 140.92, 139.04, 138.04, 131.28, 130.56, 130.43, 129.50, 129.34, 129.09, 128.53, 127.73, 125.63, 124.19, 64.37, 62.32, 42.27, 35.96, 30.76, 14.47 ppm.

HRMS (ESI$^+$): calcd for $C_{28}H_{30}N_3O_4^+$ $[M+H]^+$ 472.2231, found 472.2230.

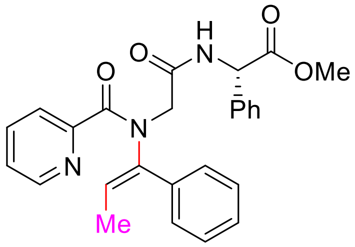

Methyl (*S,E*)-2-phenyl-2-(2-(*N*-(1-phenylprop-1-en-1-yl)picolinamido)acetamido)acetate (**6b**)

Purification via column chromatography on silica gel (MeOH/DCM = 1/5, v/v) afforded **6b** as yellow oil (27.1 mg, 61% yield, Z/E = 1:4).

^{1}**H NMR** (400 MHz, CD_3OD): δ = 8.44 (d, *J* = 4.6 Hz, 1H), 7.79 (t, *J* = 7.6 Hz, 1H), 7.60 (d, *J* = 7.4 Hz, 1H), 7.48–7.42 (m, 2H), 7.41–7.23 (m, 9H), 5.61 (brs, 1H), 5.51 (s, 1H), 4.26 (d, *J* = 3.4 Hz, 2H), 3.70 (s, 3H), 1.47 (m, 3H) ppm.

^{13}**C NMR** (100 MHz, CD_3OD): δ = 172.39, 171.64, 169.96, 155.37, 149.35, 141.65, 138.08, 137.44, 136.13, 130.79, 129.92, 129.59, 129.42, 129.32, 128.66, 128.04, 126.69, 125.79, 124.64, 58.11, 53.07, 14.35 ppm.

HRMS (ESI+): calcd for $C_{26}H_{26}N_3O_4^+$ [M+H]+ 444.1918, found 444.1917.

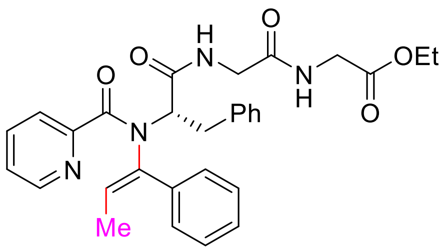

Ethyl (*E*)-(*E*)-*N*-(1-phenylprop-1-en-1-yl)-*N*-picolinoyl-*L*-phenylalanylglycylglycinate (**6c**)

Purification via column chromatography on silica gel (MeOH/DCM = 1/5, v/v) afforded **6c** as yellow oil (33.9 mg, 64% yield, Z/E = 1:5).

^{1}**H NMR** (400 MHz, CD_3OD): δ = 8.45 (d, J = 2.9 Hz, 1H), 7.78 (t, J = 4.2 Hz, 1H), 7.56 (d, J = 7.5 Hz, 1H), 7.47–7.03 (m, 11H), 5.17 (q, J = 6.5 Hz, 1H), 4.64 (t, J = 7.1 Hz, 1H), 4.22–4.09 (m, 2H), 4.00–3.75 (m, 4H), 3.37 (d, J = 7.4 Hz, 2H), 1.40–1.13 (m, 6H) ppm.

^{13}**C NMR** (100 MHz, CD_3OD): δ = 172.35, 172.08, 171.92, 171.17, 159.81, 155.81, 149.45, 140.74, 138.97, 138.03, 137.59, 131.26, 130.60, 129.50, 129.19, 127.77, 125.66, 124.22, 123.37, 64.05, 62.33, 43.62, 41.96, 36.03, 14.70, 14.45 ppm.

HRMS (ESI+): calcd for $C_{30}H_{33}N_4O_5^+$ [M+H]+ 529.2445, found 529.2444.

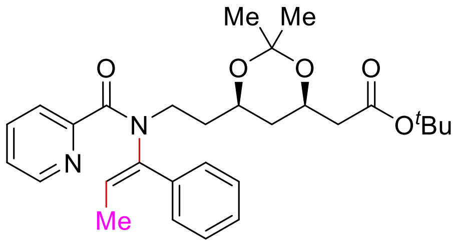

***tert*-Butyl 2-((4*R*,6*R*)-2,2-dimethyl-6-(2-(*N*-((*E*)-1-phenylprop-1-en-1-yl)picolinamido)ethyl)-1,3-dioxan-4-yl)acetate** (**6d**)

Purification via column chromatography on silica gel (ethyl acetate/petroleum ether = 1/5, v/v) afforded **6d** as yellow oil (29.2 mg, 59% yield, Z/E = 1:5).

^{1}**H NMR** (400 MHz, $CDCl_3$): δ = 8.47 (d, J = 4.4 Hz, 1H), 7.64 (td, J = 7.7, 1.6 Hz, 1H), 7.61–7.52 (m, 3H), 7.39–7.27 (m, 3H), 7.20–7.14 (m, 1H), 5.25 (q, J = 7.2 Hz, 1H), 4.27–4.17 (m, 1H), 3.93–3.87 (m, 1H), 3.62–3.40 (m, 2H), 2.39 (dd, J = 15.1, 7.2 Hz, 1H), 2.27 (dd, J = 15.1, 5.9 Hz, 1H), 2.05–2.03 (m, 1H),

1.79–1.75 (m, 2H), 1.60–1.50 (m, 1H), 1.45 (d, *J* = 7.2 Hz, 3H, overlap), 1.43 (s, 9H, overlap), 1.40 (s, 3H, overlap), 1.31 (s, 3H) ppm.

^{13}**C NMR** (100 MHz, $CDCl_3$): δ = 170.40, 169.43, 155.36, 148.33, 140.22, 136.31, 135.19, 129.88, 128.49, 128.28, 125.34, 123.80, 123.02, 98.78, 80.69, 67.12, 66.26, 42.85, 42.60, 36.47, 34.16, 30.15, 28.22, 19.82, 14.34 ppm.

HRMS (ESI$^+$): calcd for $C_{29}H_{39}N_2O_5^+$ $[M+H]^+$ 495.2853, found 495.2851.

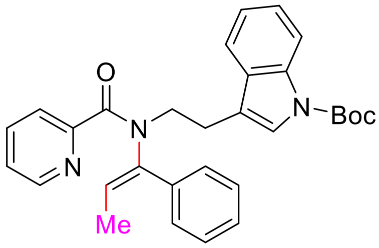

***tert*-Butyl (*E*)-3-(2-(*N*-(1-phenylprop-1-en-1-yl)picolinamido)ethyl)-1*H*-indole-1-carboxylate** (**6e**)

Purification via column chromatography on silica gel (ethyl acetate/petroleum ether = 1/4, v/v) afforded **6e** as yellow oil (34.0 mg, 70% yield, Z/E = 1:4).

^{1}**H NMR** (400 MHz, $CDCl_3$): δ = 8.50 (d, *J* = 4.4 Hz, 1H), 8.09 (d, *J* = 7.4 Hz, 1H), 7.73–7.56 (m, 4H), 7.43–7.32 (m, 5H), 7.31–7.25 (m, 1H), 7.24–7.15 (m, 2H), 5.24 (q, *J* = 7.2 Hz, 1H), 3.74 (t, *J* = 7.2 Hz, 2H), 3.04 (t, *J* = 7.6 Hz, 2H), 1.65 (s, 9H), 1.46 (d, *J* = 7.2 Hz, 3H) ppm.

^{13}**C NMR** (100 MHz, $CDCl_3$): δ = 169.56, 155.24, 149.84, 148.37, 140.53, 136.35, 135.13, 130.65, 130.01, 128.56, 128.37, 127.34, 125.06, 124.37, 123.94, 123.25, 123.18, 122.49, 119.26, 117.90, 115.24, 83.46, 46.44, 28.35, 23.22, 14.34 ppm.

HRMS (ESI$^+$): calcd for $C_{30}H_{32}N_3O_3^+$ $[M+H]^+$ 482.2438, found 482.2435.

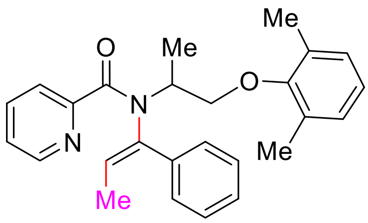

(*E*)-*N*-(1-(2,6-dimethylphenoxy)propan-2-yl)-*N*-(1-phenylprop-1-en-1-yl)picolinamide (**6f**)

Purification via column chromatography on silica gel (ethyl acetate/petroleum ether = 1/5, v/v) afforded **6f** as yellow oil (24.5 mg, 61% yield, Z/E = 1:10).

^{1}**H NMR** (400 MHz, $CDCl_3$): δ = 8.45 (d, *J* = 4.6 Hz, 1H), 7.66–7.63 (m, 3H), 7.55 (d, *J* = 7.8 Hz, 1H),

7.38–7.31 (m, 3H), 7.17 (dd, J = 6.7, 5.1 Hz, 1H), 6.97 (d, J = 7.4 Hz, 2H), 6.89 (dd, J = 8.0, 7.2 Hz, 1H), 5.39 (q, J = 7.2 Hz, 1H), 4.42–4.33 (m, 1H), 4.13 (dd, J = 8.8, 5.5 Hz, 1H), 3.84 (brs, 1H), 2.20 (s, 6H), 1.48 (d, J = 7.3 Hz, 3H), 1.44 (d, J = 6.9 Hz, 3H) ppm.

^{13}**C NMR** (100 MHz, $CDCl_3$): δ = 169.86, 155.91, 155.73, 148.21, 140.46, 136.31, 136.15, 131.05, 130.39, 128.84, 128.28, 128.12, 126.38, 123.82, 123.71, 123.00, 73.37, 54.76, 16.39, 15.50, 14.50 ppm.

HRMS (ESI$^+$): calcd for $C_{26}H_{29}N_2O_2^+$ [M+H]$^+$ 401.2224, found 401.2220.

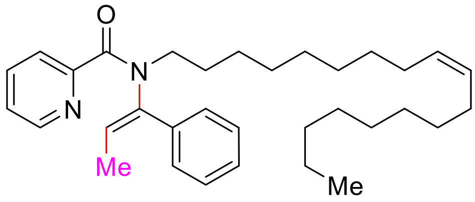

***N*-((*Z*)-octadec-9-en-1-yl)-*N*-((*E*)-1-phenylprop-1-en-1-yl)picolinamide** (**6g**)

Purification via column chromatography on silica gel (ethyl acetate/petroleum ether = 1/6, v/v) afforded **6g** as yellow oil (19.6 mg, 40% yield, Z/E = 1:4).

^{1}**H NMR** (400 MHz, $CDCl_3$): δ = 8.47 (d, J = 4.6 Hz, 1H), 7.68–7.49 (m, 4H), 7.37 (t, J = 7.3 Hz, 2H), 7.34–7.28 (m, 1H), 7.21–7.14 (m, 1H), 5.40–5.30 (m, 2H), 5.25 (q, J = 7.2 Hz, 1H), 3.43 (m, 2H), 2.01–1.95 (m, 4H), 1.61–1.54 (m, 2H), 1.46 (d, J = 7.3 Hz, 3H), 1.33–1.25 (m, 22H), 0.87 (t, J = 6.8 Hz, 3H) ppm.

^{13}**C NMR** (100 MHz, $CDCl_3$): δ = 169.40, 155.64, 148.32, 140.31, 136.26, 135.31, 130.05, 129.89, 128.26, 127.20, 125.01, 123.69, 123.03, 122.53, 46.11, 32.75, 32.04, 29.92, 29.89, 29.84, 29.80, 29.66, 29.56, 29.46, 29.39, 27.43, 27.36, 26.98, 22.82, 14.34, 14.26 ppm.

HRMS (ESI$^+$): calcd for $C_{33}H_{49}N_2O^+$ [M+H]$^+$ 489.3839, found 489.3839.

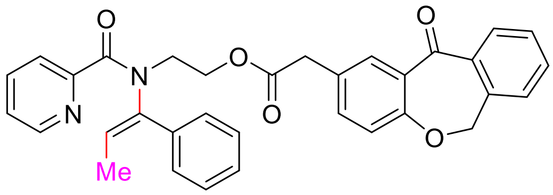

(*E*)-2-(*N*-(1-phenylprop-1-en-1-yl)picolinamido)ethyl

2-(11-oxo-6,11-dihydrodibenzo[*b*,*e*]oxepin-2-yl)acetate (**6h**)

Purification via column chromatography on silica gel (ethyl acetate/petroleum ether = 1/2, v/v) afforded **6h** as yellow oil (27.5 mg, 51% yield, Z/E = 1:4).

^{1}**H NMR** (400 MHz, CDCl$_3$): δ = 8.46 (d, J = 4.4 Hz, 1H), 8.10 (s, 1H), 7.87 (d, J = 7.7 Hz, 1H), 7.64 (t, J = 7.6 Hz, 1H), 7.58–7.50 (m, 4H), 7.46 (t, J = 7.6 Hz, 2H), 7.38–7.35 (m, 3H), 7.32–7.29 (m, 1H), 7.22–7.14 (m, 1H), 7.02 (d, J = 8.4 Hz, 1H), 5.25 (q, J = 7.1 Hz, 1H), 5.17 (s, 2H), 4.31 (t, J = 5.6 Hz, 2H), 3.74 (brs, 2H), 3.63 (s, 2H), 1.43 (d, J = 7.3 Hz, 3H) ppm.

^{13}**C NMR** (100 MHz, CDCl$_3$): δ = 190.86, 171.37, 169.84, 160.59, 154.88, 148.28, 140.57, 140.20, 136.64, 136.35, 135.69, 134.86, 132.87, 132.59, 129.93, 129.60, 129.36, 128.57, 128.40, 127.91, 127.27, 125.43, 125.23, 123.98, 123.32, 121.20, 73.75, 62.12, 44.73, 40.26, 14.35 ppm.

HRMS (ESI$^+$): calcd for $C_{33}H_{29}N_2O_5^+$ [M+H]$^+$ 533.2071, found 533.2068.

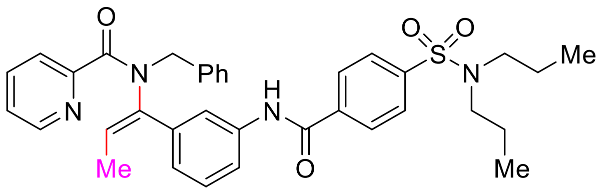

(*E*)-*N*-benzyl-*N*-(1-(3-(4-(*N*,*N*-dipropylsulfamoyl)benzamido)phenyl)prop-1-en-1-yl)picolinamide (**6i**)

Purification via column chromatography on silica gel (ethyl acetate/petroleum ether = 1/2, v/v) afforded **6i** as yellow oil (25.7 mg, 42% yield, Z/E = 1:4).

^{1}**H NMR** (400 MHz, CDCl$_3$): δ = 9.21 (brs, 1H), 8.43 (d, J = 4.5 Hz, 1H), 8.08 (d, J = 8.3 Hz, 2H), 8.04 (d, J = 8.2 Hz, 1H), 7.88–7.82 (m, 2H), 7.80 (brs, 1H), 7.59 (t, J = 7.6 Hz, 1H), 7.47 (d, J = 7.6 Hz, 1H), 7.37 (t, J = 7.9 Hz, 1H), 7.31–7.20 (m, 5H), 7.17–7.13 (m, 2H), 5.06 (q, J = 7.2 Hz, 1H), 4.60 (brs, 2H), 3.10–3.06 (m, 4H), 1.61–1.47 (m, 4H), 1.39 (d, J = 7.3 Hz, 3H), 0.86 (t, J = 7.4 Hz, 6H) ppm.

^{13}**C NMR** (100 MHz, CDCl$_3$): δ = 169.70, 164.98, 154.74, 148.54, 142.98, 139.02, 138.71, 137.15, 136.39, 135.53, 130.17, 129.62, 128.98, 128.94, 128.47, 128.40, 127.49, 127.30, 126.57, 124.06, 122.95, 121.14, 120.62, 50.10, 49.72, 22.07, 14.37, 11.31 ppm.

HRMS (ESI$^+$): calcd for $C_{35}H_{39}N_4O_4S^+$ [M+H]$^+$ 611.2687, found 611.2682.

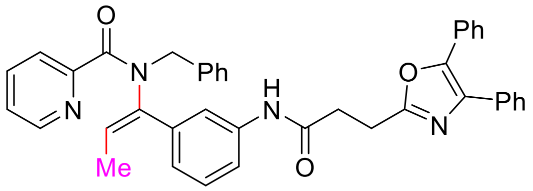

(*E*)-*N*-benzyl-*N*-(1-(3-(3-(4,5-diphenyloxazol-2-yl)propanamido)phenyl)prop-1-en-1-yl)picolinamide (**6j**)

Purification via column chromatography on silica gel (ethyl acetate/petroleum ether = 1/3, v/v) afforded **6j** as yellow oil (29.9 mg, 48% yield, Z/E = 1:7).

[1]**H NMR** (400 MHz, $CDCl_3$): δ = 8.62 (brs, 1H), 8.43 (d, J = 4.6 Hz, 1H), 7.67 (d, J = 7.7 Hz, 1H), 7.65 – 7.53 (m, 6H), 7.38–7.11 (m, 15H), 5.05 (q, J = 7.2 Hz, 1H), 4.63 (brs, 2H), 3.30 (t, J = 6.9 Hz, 2H), 2.98 (t, J = 6.9 Hz, 2H), 1.30 (d, J = 8.0 Hz, 3H) ppm.

[13]**C NMR** (100 MHz, $CDCl_3$): δ = 170.05, 169.67, 162.62, 155.05, 148.41, 145.76, 139.17, 138.43, 137.28, 136.36, 135.71, 134.96, 132.32, 130.24, 129.04, 128.96, 128.79, 128.75, 128.71, 128.39, 128.34, 127.97, 127.55, 127.34, 126.57, 125.71, 123.89, 123.09, 120.95, 119.89, 49.70, 34.13, 24.10, 14.23 ppm.

HRMS (ESI^+): calcd for $C_{40}H_{35}N_4O_3^+$ $[M+H]^+$ 619.2704, found 619.2702.

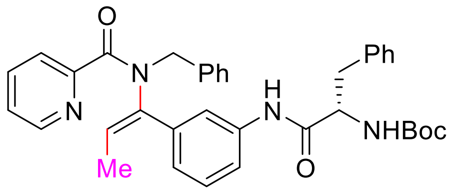

***tert*-Butyl (*S*,*E*)-(1-((3-(1-(*N*-benzylpicolinamido)prop-1-en-1-yl)phenyl)amino)-1-oxo-3-phenylpropan-2-yl)carbamate (6k)**

Purification via column chromatography on silica gel (ethyl acetate/petroleum ether = 1/3, v/v) afforded **6k** as yellow oil (26.8 mg, 45% yield, Z/E = 1:6).

[1]**H NMR** (400 MHz, $CDCl_3$): δ = 8.46 (d, J = 4.5 Hz, 1H), 8.11 (brs, 1H), 7.64 (t, J = 7.6 Hz, 1H), 7.57 (d, J = 7.6 Hz, 1H), 7.52 (d, J = 8.1 Hz, 1H), 7.45 (s, 1H), 7.38–7.06 (m, 13H), 5.27 (brs, 1H), 5.06 (q, J = 7.2 Hz, 1H), 4.75–4.42 (m, 3H), 3.21–3.10 (m, 2H), 1.41 (s, 9H), 1.36 (d, J = 7.3 Hz, 3H) ppm.

[13]**C NMR** (100 MHz, $CDCl_3$): δ = 169.84, 169.59, 156.10, 155.01, 148.39, 139.16, 137.59, 137.26, 136.76, 136.37, 135.78, 129.47, 129.08, 128.87, 128.39, 127.53, 127.36, 127.17, 126.71, 126.16, 123.94, 123.18, 121.41, 120.20, 56.68, 49.70, 44.61, 38.62, 28.39, 14.29 ppm.

HRMS (ESI^+): calcd for $C_{36}H_{39}N_4O_4^+$ $[M+H]^+$ 591.2966, found 591.2964.

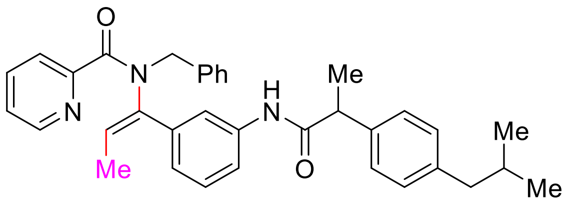

(*E*)-*N*-benzyl-*N*-(1-(3-(2-(4-isobutylphenyl)propanamido)phenyl)prop-1-en-1-yl)picolinamide (**6l**)

Purification via column chromatography on silica gel (ethyl acetate/petroleum ether = 1/4, v/v) afforded **6l** as yellow oil (25.6 mg, 48% yield, Z/E = 1:4).

^{1}**H NMR** (400 MHz, $CDCl_3$): δ = 8.42 (d, J = 4.6 Hz, 1H), 7.61 (d, J = 7.7 Hz, 2H), 7.54–7.49 (m, 1H), 7.44 (s, 1H), 7.37–7.20 (m, 9H), 7.19–7.12 (m, 3H), 7.09 (d, J = 7.9 Hz, 1H), 5.08 (q, J = 7.2 Hz, 1H), 4.62 (brs, 2H), 3.72 (q, J = 7.1 Hz, 1H), 2.47 (d, J = 7.2 Hz, 2H), 1.91–1.81 (m, 1H), 1.59 (d, J = 7.2 Hz, 3H), 1.35 (d, J = 7.3 Hz, 3H), 0.90 (d, J = 6.6 Hz, 6H) ppm.

^{13}**C NMR** (100 MHz, $CDCl_3$): δ = 172.81, 169.62, 155.08, 148.31, 141.17, 139.25, 138.27, 137.31, 136.40, 135.80, 129.94, 129.08, 128.82, 128.39, 127.55, 127.36, 126.58, 125.84, 124.48, 123.91, 123.21, 120.85, 119.82, 49.86, 47.86, 45.16, 31.88, 30.32, 22.51, 18.75, 14.29 ppm.

HRMS (ESI$^+$): calcd for $C_{35}H_{38}N_3O_2^+$ $[M+H]^+$ 532.2959, found 532.2957.

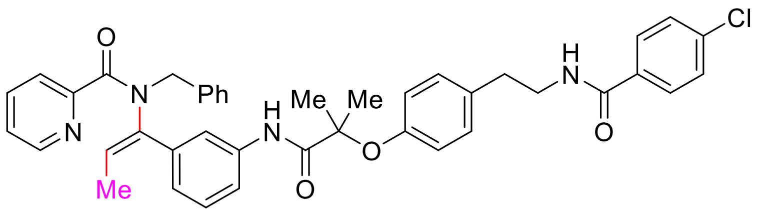

(*E*)-*N*-benzyl-*N*-(1-(3-(2-(4-(2-(4-chlorobenzamido)ethyl)phenoxy)-2-methylpropanamido)phenyl)prop-1-en-1-yl)picolinamide (**6m**)

Purification via column chromatography on silica gel (ethyl acetate/petroleum ether = 1/1, v/v) afforded **6m** as a pale yellow solid (35.3 mg, 51% yield, Z/E = 1:5). M.p.: 182–185 °C.

^{1}**H NMR** (400 MHz, $CDCl_3$): δ = 8.57 (s, 1H), 8.45 (d, J = 4.5 Hz, 1H), 7.68 (d, J = 8.2 Hz, 1H), 7.65–7.57 (m, 3H), 7.53 (d, J = 7.9 Hz, 1H), 7.51 (s, 1H), 7.42–7.09 (m, 12H), 6.97 (d, J = 7.8 Hz, 2H), 6.28 (t, J = 5.5 Hz, 1H), 5.14 (q, J = 7.1 Hz, 1H), 4.68 (brs, 2H), 3.67 (q, J = 6.8 Hz, 2H), 2.90 (t, J = 6.9 Hz, 2H), 1.59 (s, 6H), 1.41 (d, J = 7.3 Hz, 3H) ppm.

^{13}**C NMR** (100 MHz, $CDCl_3$): δ = 173.14, 169.61, 166.60, 155.01, 152.64, 148.30, 139.31, 137.70, 137.61, 137.30, 136.38, 136.16, 134.51, 133.03, 130.23, 129.83, 129.08, 128.86, 128.41, 127.54, 127.39,

126.46, 126.23, 123.95, 123.23, 122.14, 121.15, 119.92, 82.03, 50.05, 41.44, 35.00, 25.09, 14.33 ppm.

HRMS (ESI$^+$): calcd for $C_{41}H_{40}{}^{35}ClN_4O_4^+$ [M+H]$^+$ 687.2733, found 687.2731; $C_{41}H_{40}{}^{37}ClN_4O_4^+$ [M+H]$^+$ 689.2703, found 689.2720.

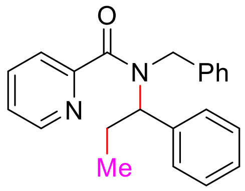

***N*-benzyl-*N*-(1-phenylpropyl)picolinamide** (**7a**)

The ratio of the two diastereoisomers is 1.2:1.

^{1}**H NMR** (400 MHz, $CDCl_3$, a mixture of two isomers): δ = 8.67 (d, *J* = 4.2 Hz, major isomer), 8.48 (d, *J* = 3.7 Hz, minor isomer), 7.82 (t, *J* = 7.6 Hz), 7.70 (d, *J* = 7.8 Hz), 7.54 (t, *J* = 7.3 Hz), 7.47–7.45 (m), 7.39–7.11 (m), 7.03–7.01 (m), 6.76 (d, *J* = 5.8 Hz), 5.86 (t, *J* = 7.6 Hz, C<u>H</u>CH_2CH_3, minor isomer), 5.22 (t, *J* = 7.4 Hz, C<u>H</u>CH_2CH_3, major isomer), 4.76 (d, *J* = 15.1 Hz, $C_6H_5C\underline{H}_2$, major isomer), 4.56 (d, *J* = 16.2 Hz, $C_6H_5C\underline{H}_2$, minor isomer), 4.44 (d, *J* = 16.2 Hz, $C_6H_5C\underline{H}_2$, minor isomer), 4.21 (d, *J* = 15.1 Hz, $C_6H_5C\underline{H}_2$, major isomer), 2.17–1.87 (m, $CHC\underline{H}_2CH_3$, major + minor isomer), 1.03 (t, *J* = 7.1 Hz, $CHCH_2C\underline{H}_3$, minor isomer), 0.75 (t, *J* = 7.2 Hz, $CHCH_2C\underline{H}_3$, major isomer) ppm.

^{13}**C NMR** (100 MHz, $CDCl_3$, a mixture of two isomers): δ = 170.30, 170.09, 155.53, 155.48, 148.55, 148.52, 148.08, 148.07, 139.35, 139.06, 138.66, 138.48, 137.12, 136.68, 129.01, 128.64, 128.56, 128.40, 128.19, 128.02, 127.96, 127.86, 127.84, 127.39, 126.82, 126.76, 124.31, 123.97, 123.93, 123.54, 62.74, 59.57, 48.58, 45.50, 24.70, 24.27, 11.56, 11.35 ppm.

HRMS (ESI$^+$): calcd for $C_{22}H_{23}N_2O^+$ [M+H]$^+$ 331.1805, found 331.1801.

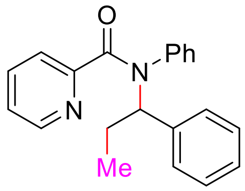

***N*-phenyl-*N*-(1-phenylpropyl)picolinamide** (**7b**)

^{1}**H NMR** (400 MHz, acetone-d_6): δ = 8.21 (brs, 1H), 7.63 (m, 1H), 7.42 (m, 1H), 7.34 – 7.23 (m, 4H), 7.11–7.06 (m, 4H), 6.73 (br, 2H), 6.08 (br, 1H), 2.18 – 1.89 (m, 2H), 1.10 (t, *J* = 7.1 Hz, 3H) ppm.

^{13}**C NMR** (100 MHz, acetone-d_6): δ = 169.68, 156.60, 148.93, 141.03, 140.05, 136.84, 131.61, 129.41,

129.00, 128.71, 128.33, 127.94, 124.09, 123.81, 60.69, 24.80, 11.75 ppm.

HRMS (ESI^+): calcd for $C_{21}H_{21}N_2O^+$ $[M+H]^+$ 317.1648, found 317.1646.

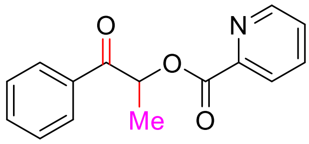

1-Oxo-1-phenylpropan-2-yl picolinate (**7c**)

^{1}**H NMR** (400 MHz, $CDCl_3$): δ = 8.81 (dd, J = 4.7, 0.7 Hz, 1H), 8.15 (dd, J = 7.8, 0.8 Hz, 1H), 8.00 (d, J = 8.3 Hz, 2H), 7.85 (tt, J = 8.0, 1.2 Hz, 1H), 7.63–7.55 (m, 1H), 7.54–7.43 (m, 3H), 6.26 (qd, J = 7.0, 0.8 Hz, 1H), 1.72 (dd, J = 7.0, 0.9 Hz, 3H) ppm.

^{13}**C NMR** (100 MHz, $CDCl_3$): δ = 196.31, 164.54, 150.04, 147.40, 137.31, 133.79, 130.16, 128.94, 128.66, 127.36, 125.68, 73.01, 17.41 ppm.

HRMS (ESI^+): calcd for $C_{15}H_{14}NO_3^+$ $[M+H]^+$ 256.0968, found 256.0965.

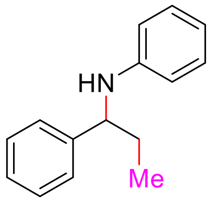

***N*-(1-phenylpropyl)aniline** (**7d**)[4]

^{1}**H NMR** (400 MHz, $CDCl_3$): δ = 7.35–7.29 (m, 4H), 7.23–7.20 (m, 1H), 7.07 (t, J = 7.6 Hz, 2H), 6.62 (t, J = 7.3 Hz, 1H), 6.51 (d, J = 7.9 Hz, 2H), 4.22 (t, J = 6.8 Hz, 1H), 4.07 (brs, 1H), 1.88–1.76 (m, 2H), 0.95 (t, J = 7.4 Hz, 3H) ppm.

^{13}**C NMR** (100 MHz, $CDCl_3$): δ = 147.63, 144.05, 129.21, 128.62, 127.00, 126.61, 117.23, 113.34, 59.84, 31.82, 10.98 ppm.

HRMS (ESI^+): calcd for $C_{15}H_{18}N^+$ $[M+H]^+$ 212.1434, found 212.1431.

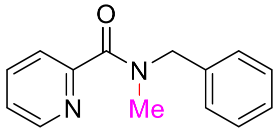

***N*-benzyl-*N*-methylpicolinamide (3a-4)**[5]

^{1}**H NMR** (400 MHz, $CDCl_3$, 1:1 mixture of rotamers): δ = 8.59 (d, J = 4.7 Hz, 1H), 8.56 (d, J = 4.8 Hz, 1H),

7.82 – 7.73 (m, 2H), 7.67 (dd, J = 8.2, 1.5 Hz, 2H), 7.39 – 7.22 (m, 11H), 4.77 (s, 2H), 4.67 (s, 2H), 3.02 (s, 3H), 2.96 (s, 3H).

^{13}C NMR (100 MHz, $CDCl_3$, 1:1 mixture of rotamers): δ = 169.42, 169.08, 154.57, 154.46, 148.42, 148.40, 137.18, 137.10, 136.84, 136.82, 128.77, 128.73, 128.27, 127.70, 127.56, 127.51, 124.53, 124.49, 123.78, 123.68, 77.48, 77.16, 76.84, 54.73, 51.28, 36.58, 33.45.

IX. References

1. K. Li, G. Tan, J. Huang, F. Song, J. You, Iron-Catalyzed Oxidative C–H/C–H Cross-Coupling: An Efficient Route to α-Quaternary α-Amino Acid Derivatives. *Angew. Chem. Int. Ed.* **2013**, *52*, 12942−12945.

2. Q. Li, S.-Y. Zhang, G. He, W. A. Nack, G. Chen, Palladium-Catalyzed Picolinamide-Directed Acetoxylation of Unactivated γ-C(sp^3)–H Bonds of Alkylamines. *Adv. Synth. Catal.* **2014**, *356*, 1544–1548.

3. M. Tan, K. Li, J. Yin, J. You, Manganese/cobalt-catalyzed oxidative C(sp^3)–H/C(sp^3)–H coupling: a route to α-tertiary β-arylethylamines. *Chem. Commun.* **2018**, *54*, 1221–1224.

4. K. Saito, K. Horiguchi, Y. Shibata, M. Yamanaka, T. Akiyama, Chiral phosphoric-acid-catalyzed transfer hydrogenation of ethylketimine derivatives by using benzothiazoline. *Chem.-Eur. J.* **2014**, *20*, 7616−7620.

5. H. Morimoto, R. Fujiwara, Y. Shimizu, K. Morisaki, T. Ohshima, Lanthanum(III) Triflate Catalyzed Direct Amidation of Esters. *Org. Lett.* **2014**, *16*, 2018−2021.

X. Copies of NMR spectra

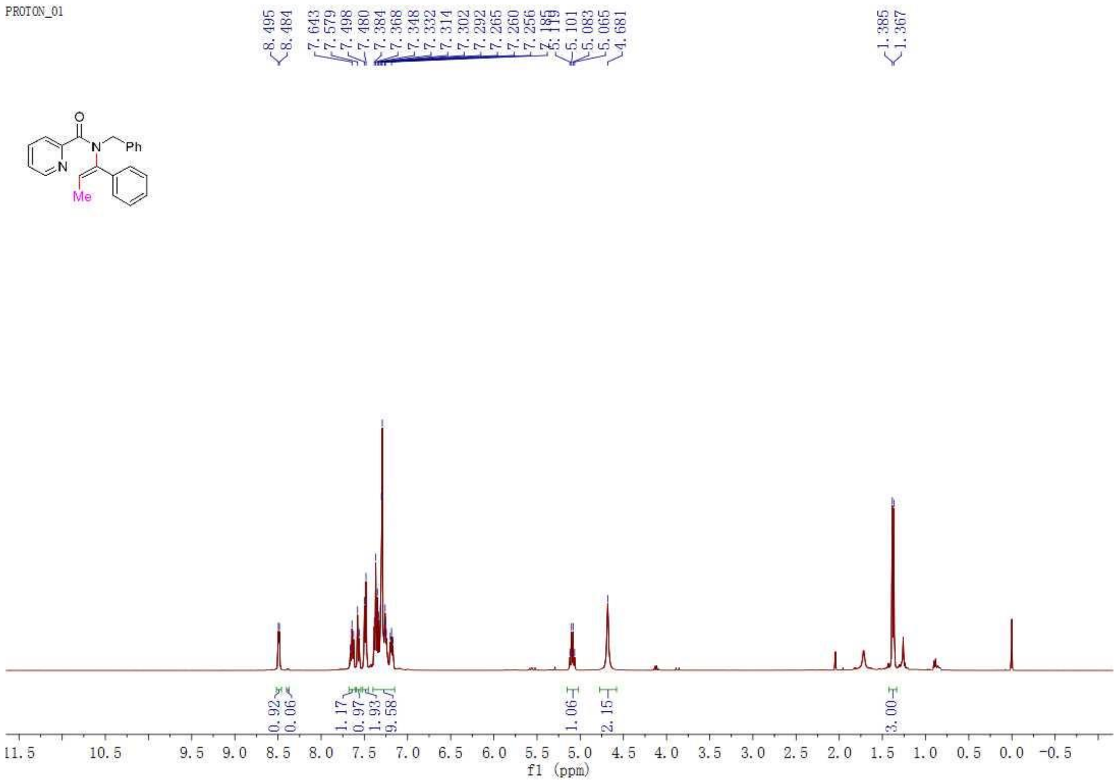

^{1}H NMR spectra for compound **3a** (400 MHz, $CDCl_3$)

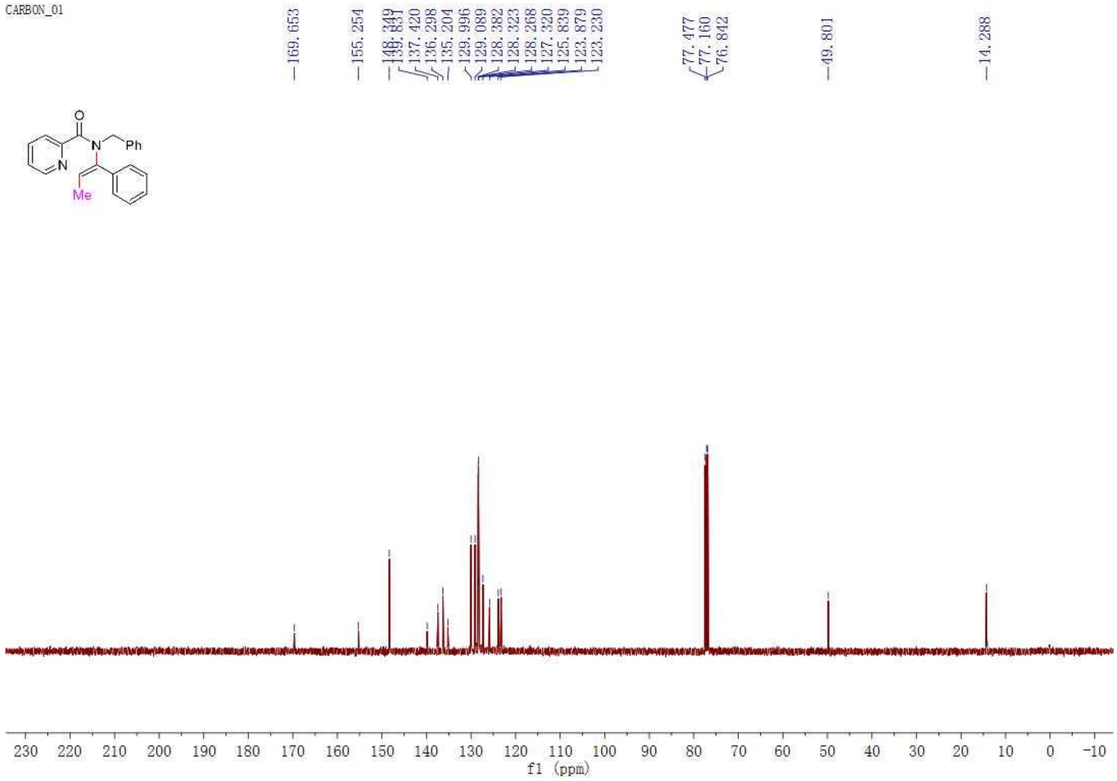

^{13}C NMR spectra for compound **3a** (100 MHz, $CDCl_3$)

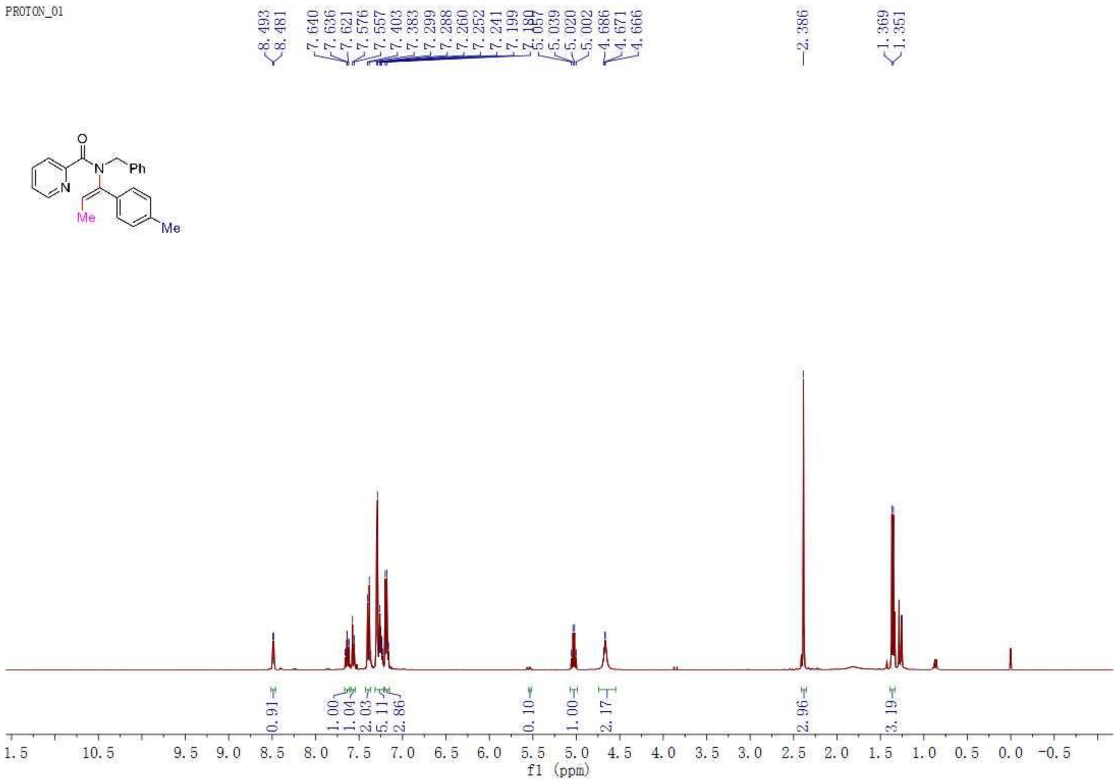

^{1}H NMR spectra for compound **3b** (400 MHz, $CDCl_3$)

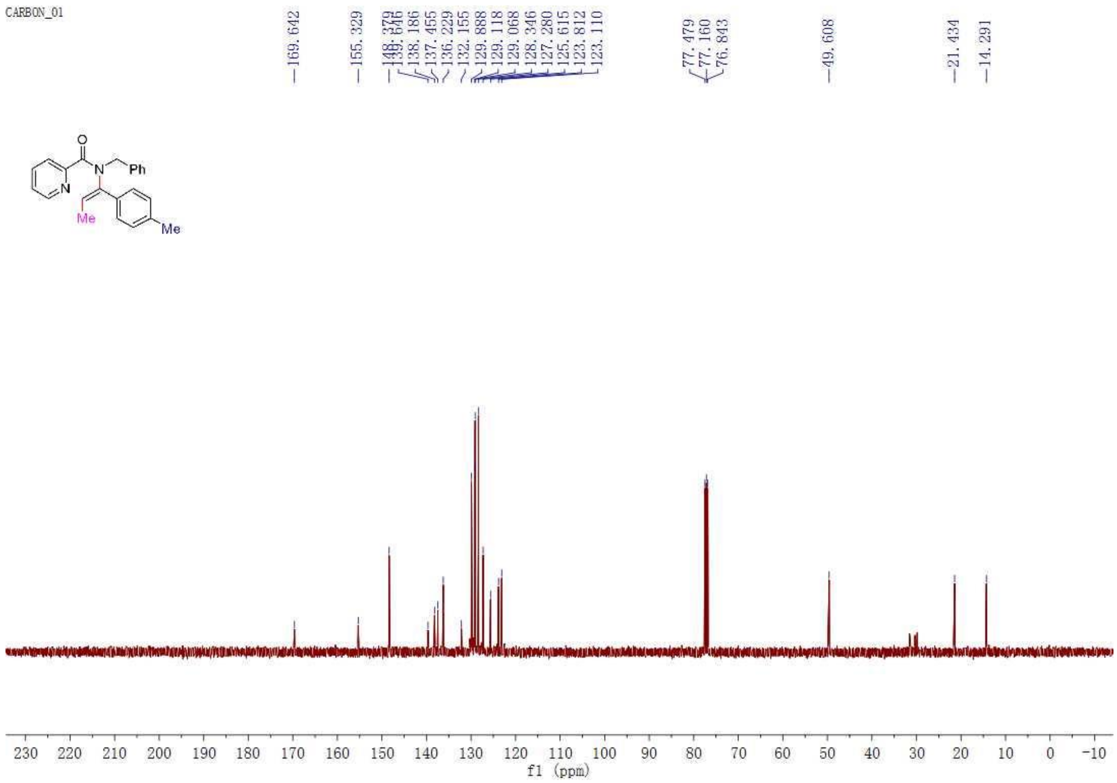

^{13}C NMR spectra for compound **3b** (100 MHz, $CDCl_3$)

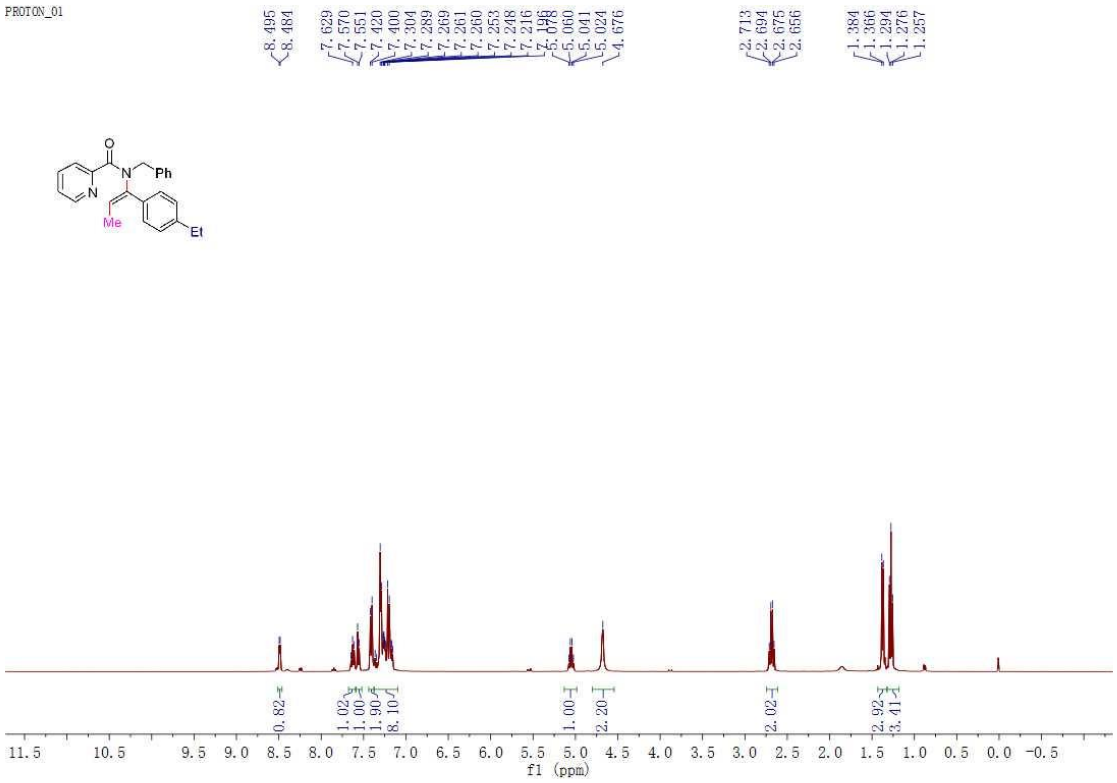

^{1}H NMR spectra for compound **3c** (400 MHz, $CDCl_3$)

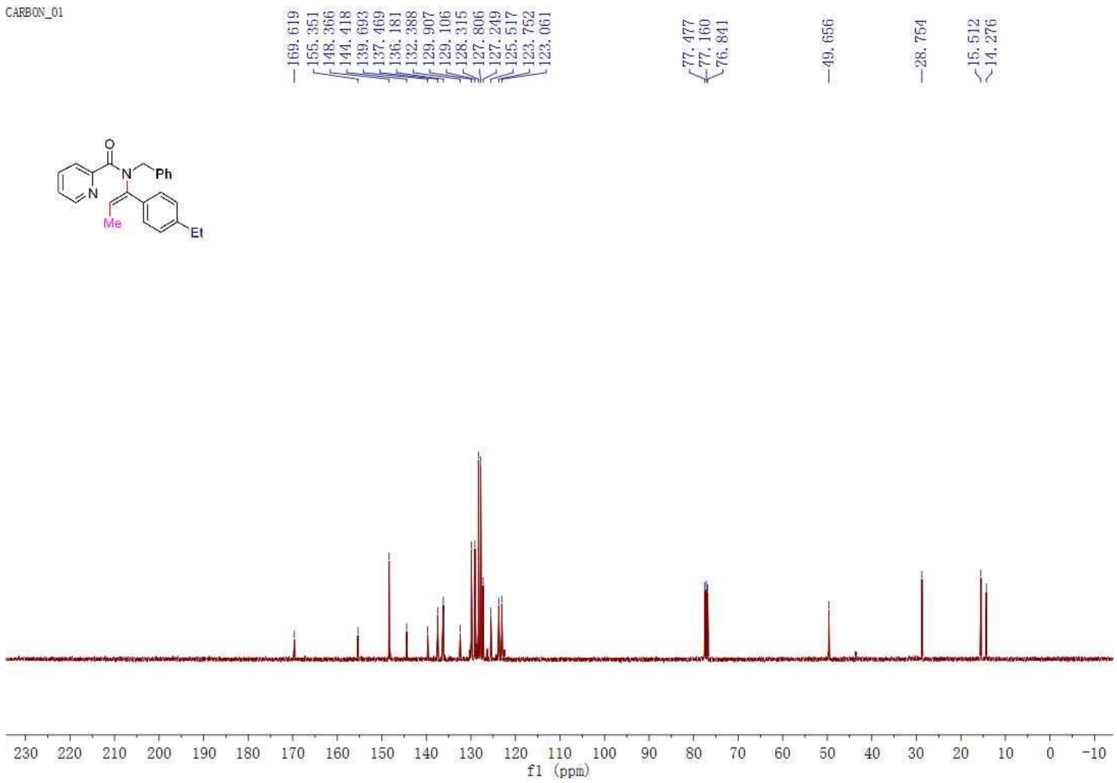

^{13}C NMR spectra for compound **3c** (100 MHz, $CDCl_3$)

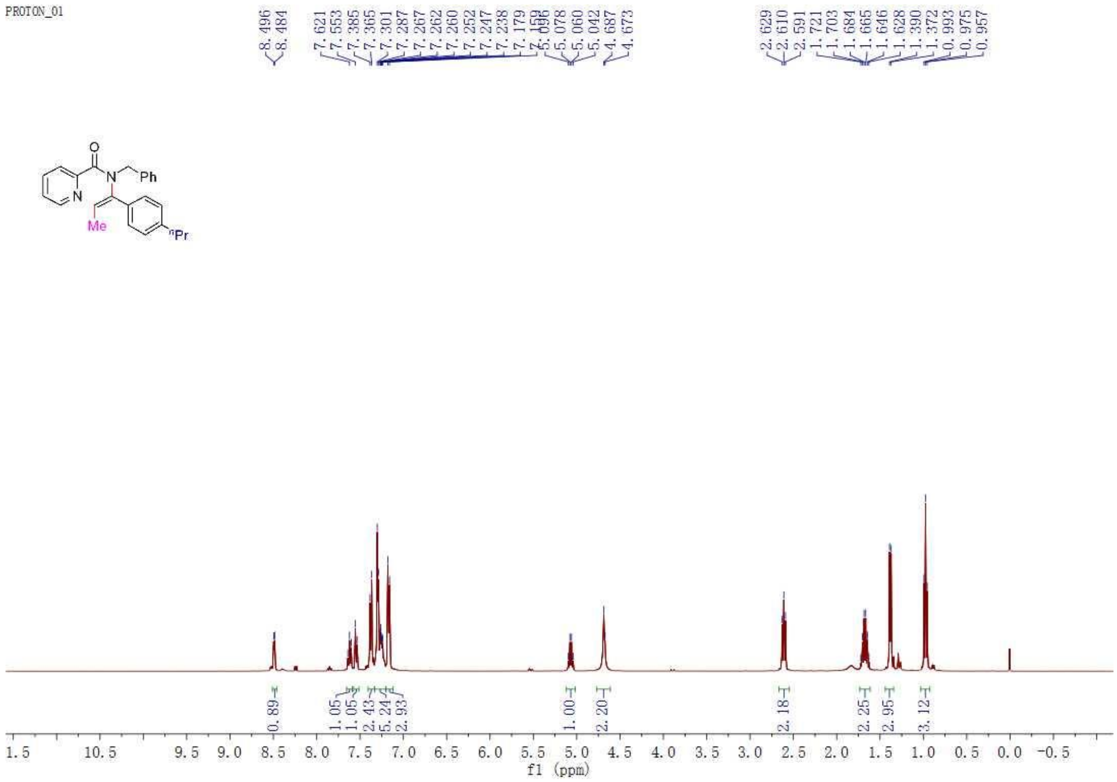

^{1}H NMR spectra for compound **3d** (400 MHz, $CDCl_3$)

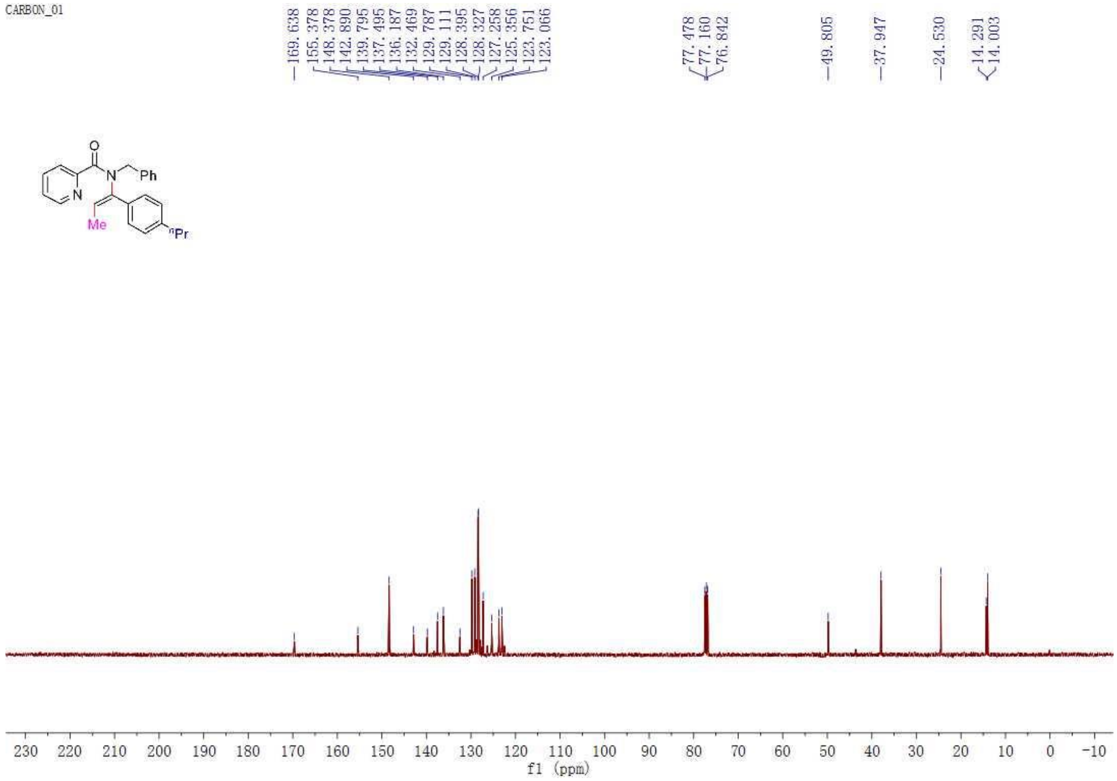

^{13}C NMR spectra for compound **3d** (100 MHz, $CDCl_3$)

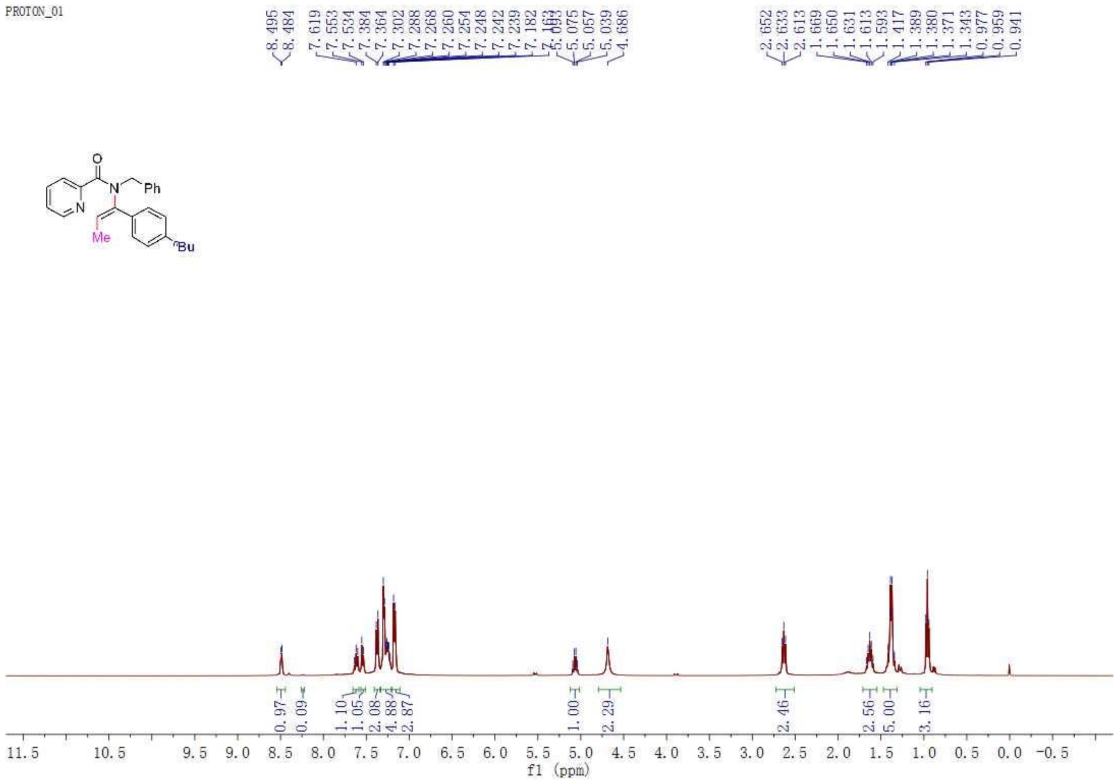

^{1}H NMR spectra for compound **3e** (400 MHz, $CDCl_3$)

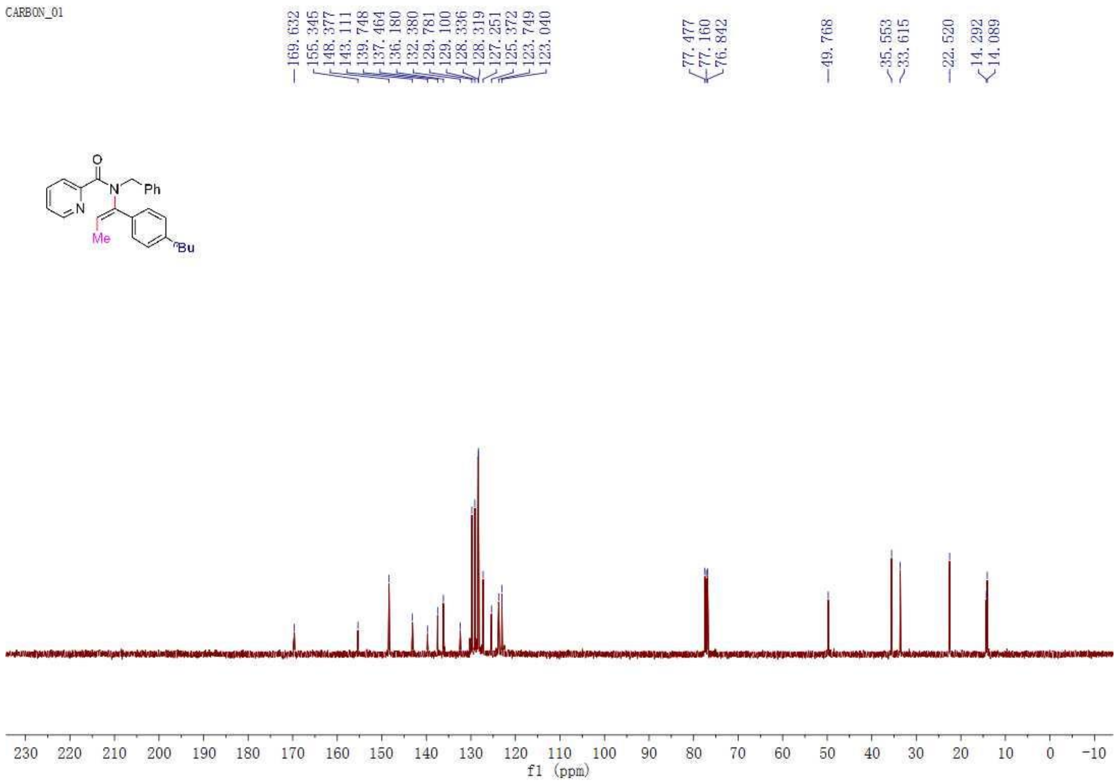

^{13}C NMR spectra for compound **3e** (100 MHz, $CDCl_3$)

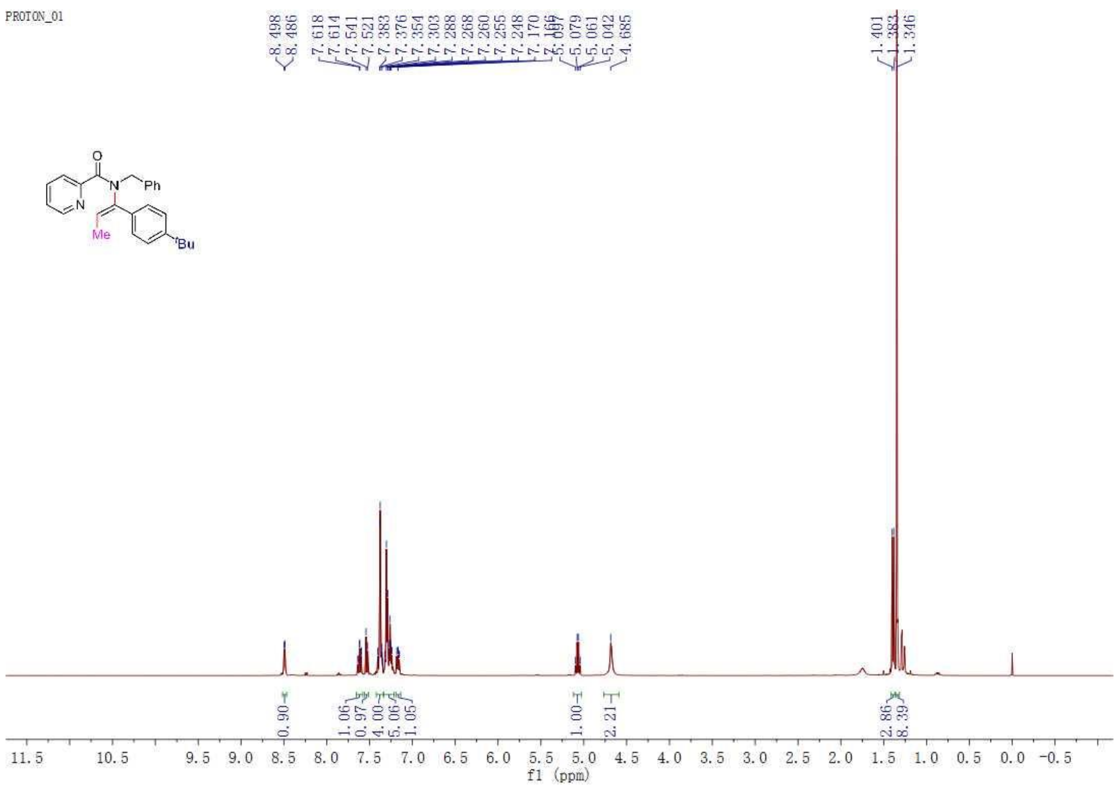

^{1}H NMR spectra for compound **3f** (400 MHz, $CDCl_3$)

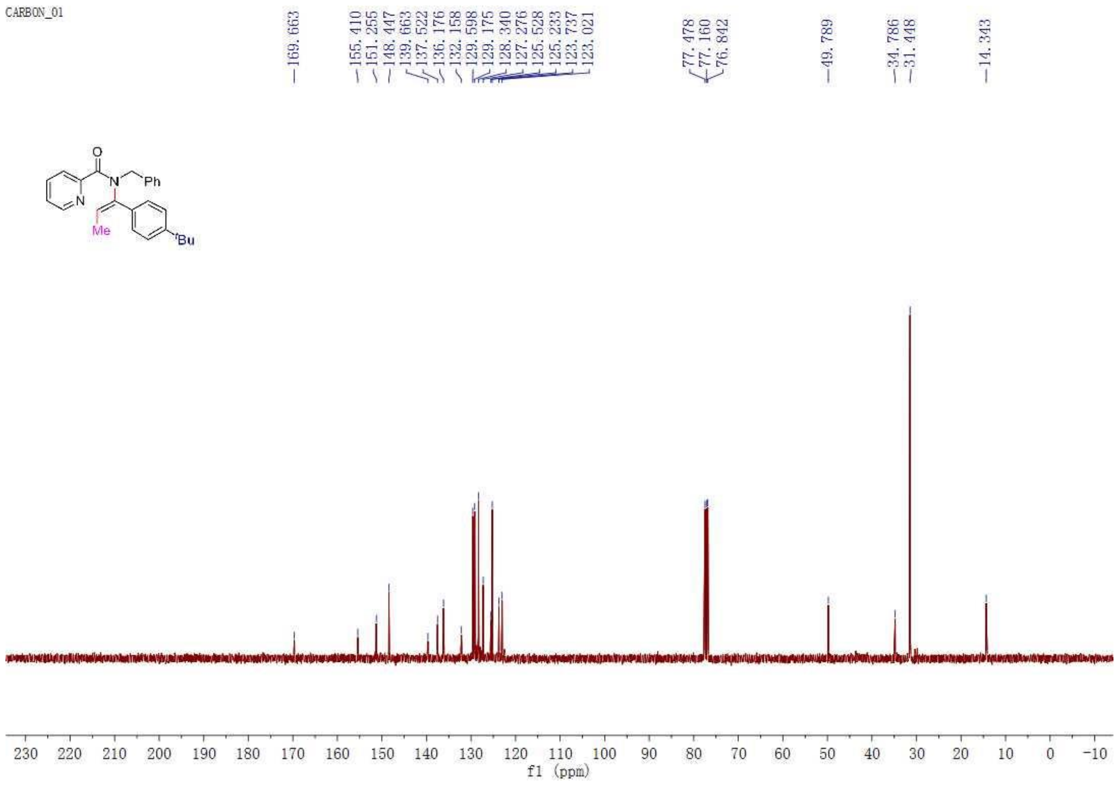

^{13}C NMR spectra for compound **3f** (100 MHz, $CDCl_3$)

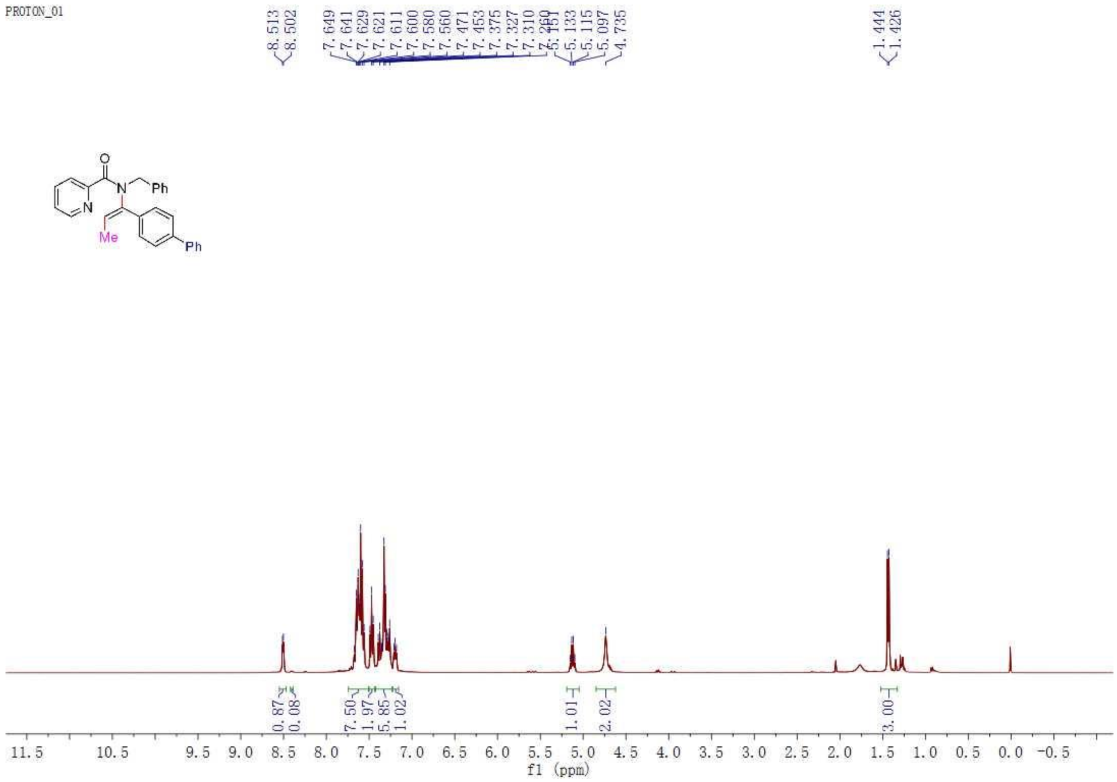

^{1}H NMR spectra for compound **3g** (400 MHz, $CDCl_3$)

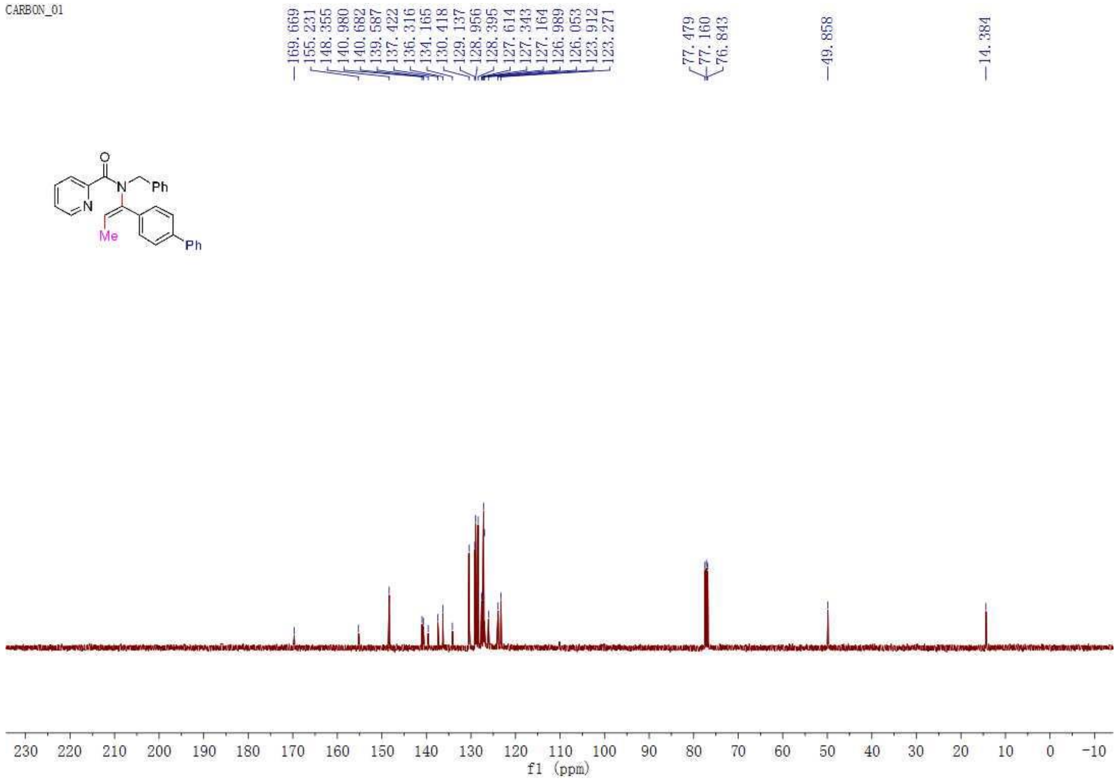

^{13}C NMR spectra for compound **3g** (100 MHz, $CDCl_3$)

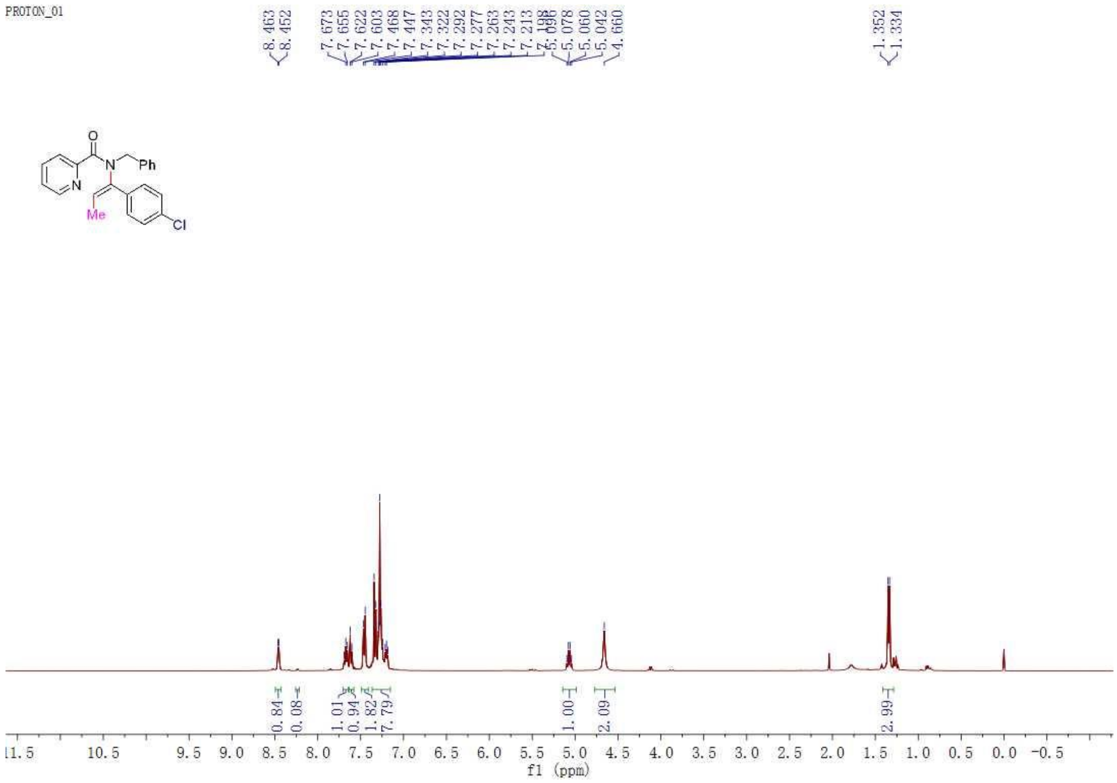

^{1}H NMR spectra for compound **3h** (400 MHz, $CDCl_3$)

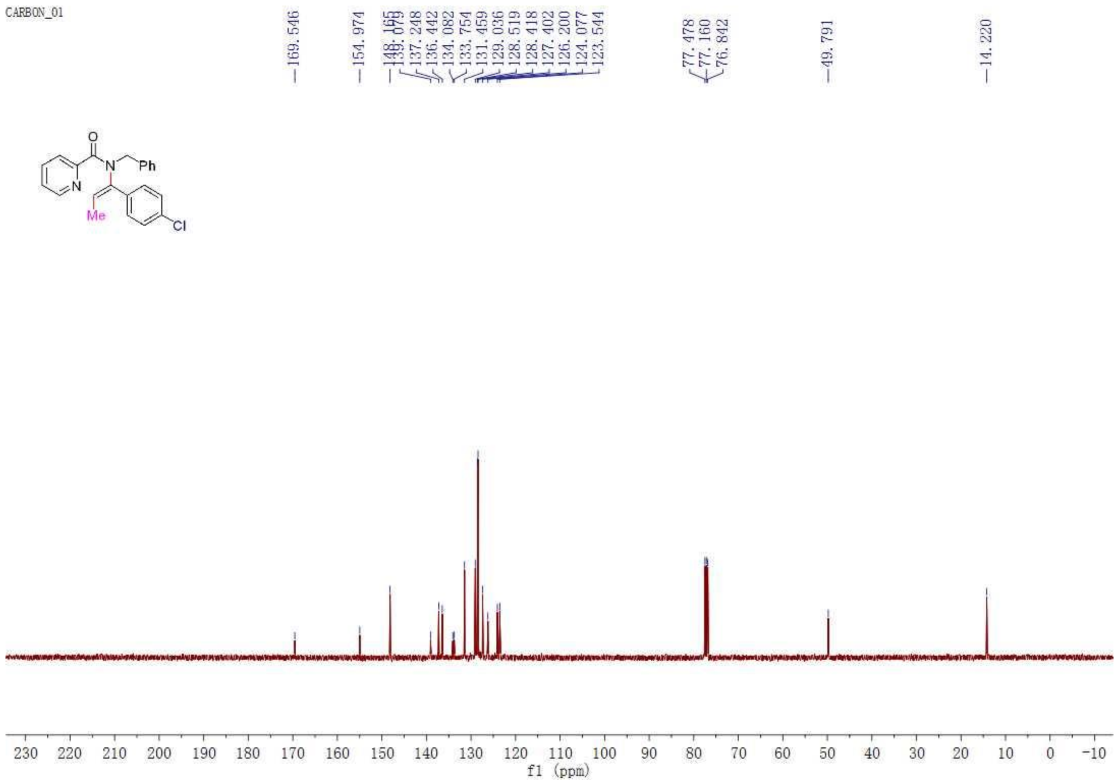

^{13}C NMR spectra for compound **3h** (100 MHz, $CDCl_3$)

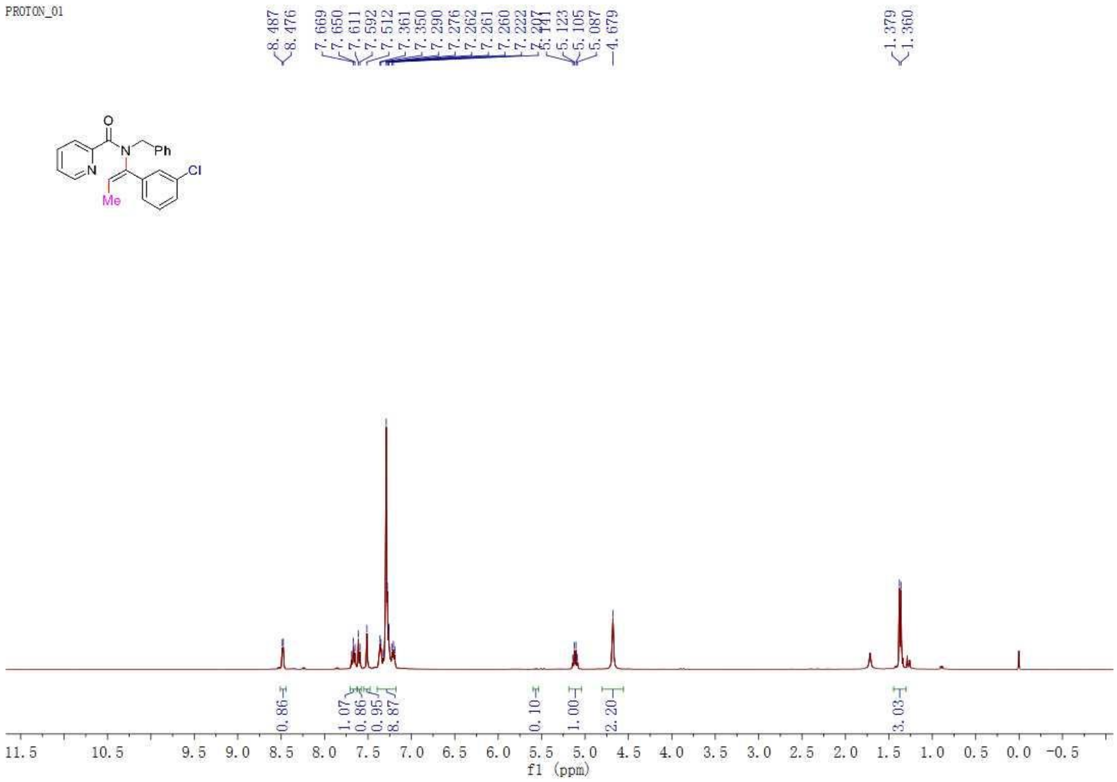

^{1}H NMR spectra for compound **3i** (400 MHz, $CDCl_3$)

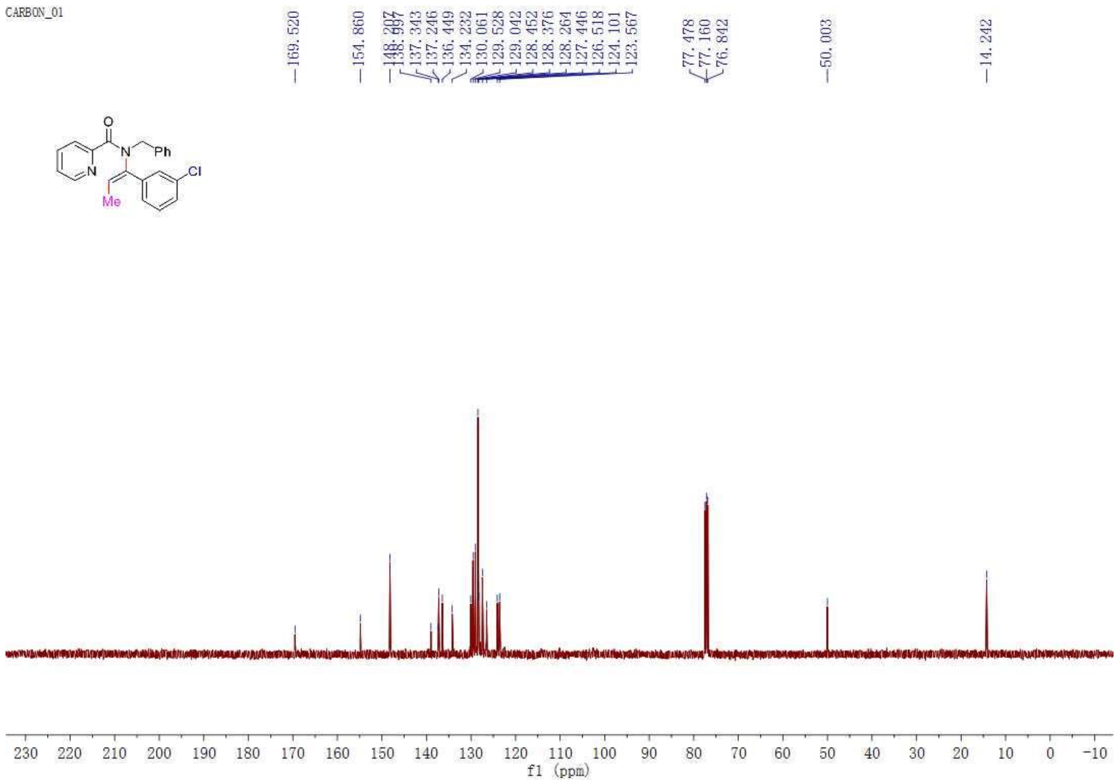

^{13}C NMR spectra for compound **3i** (100 MHz, $CDCl_3$)

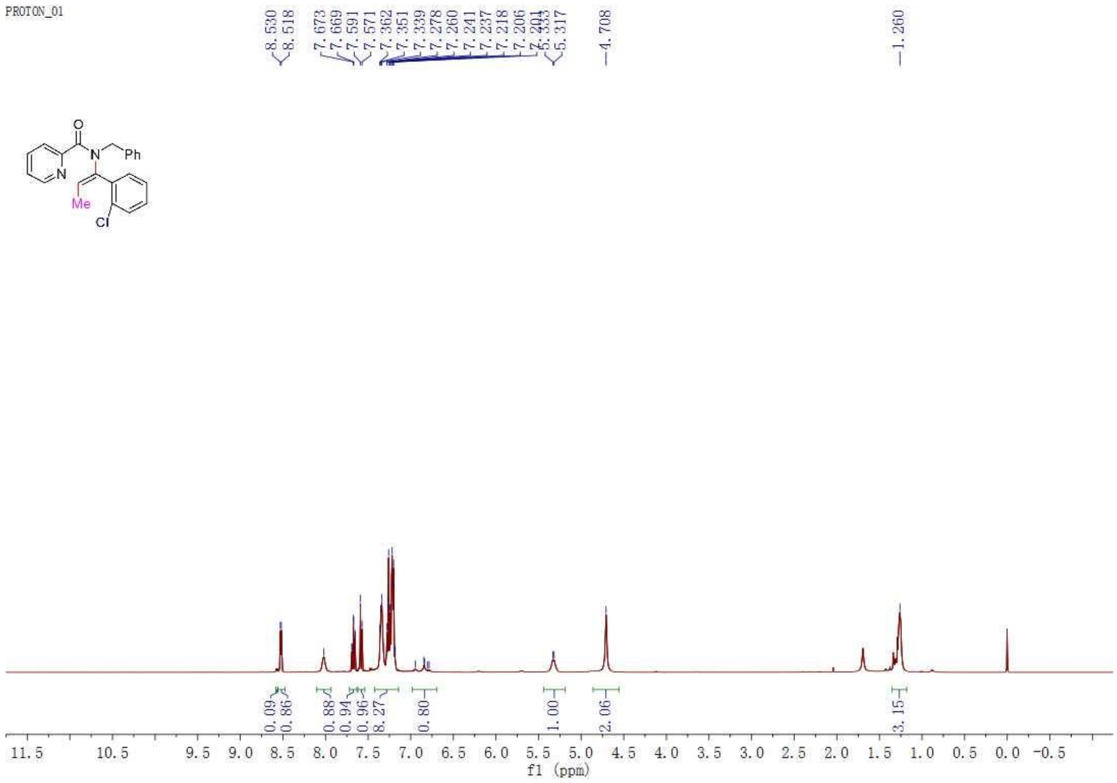

^{1}H NMR spectra for compound **3j** (400 MHz, $CDCl_3$)

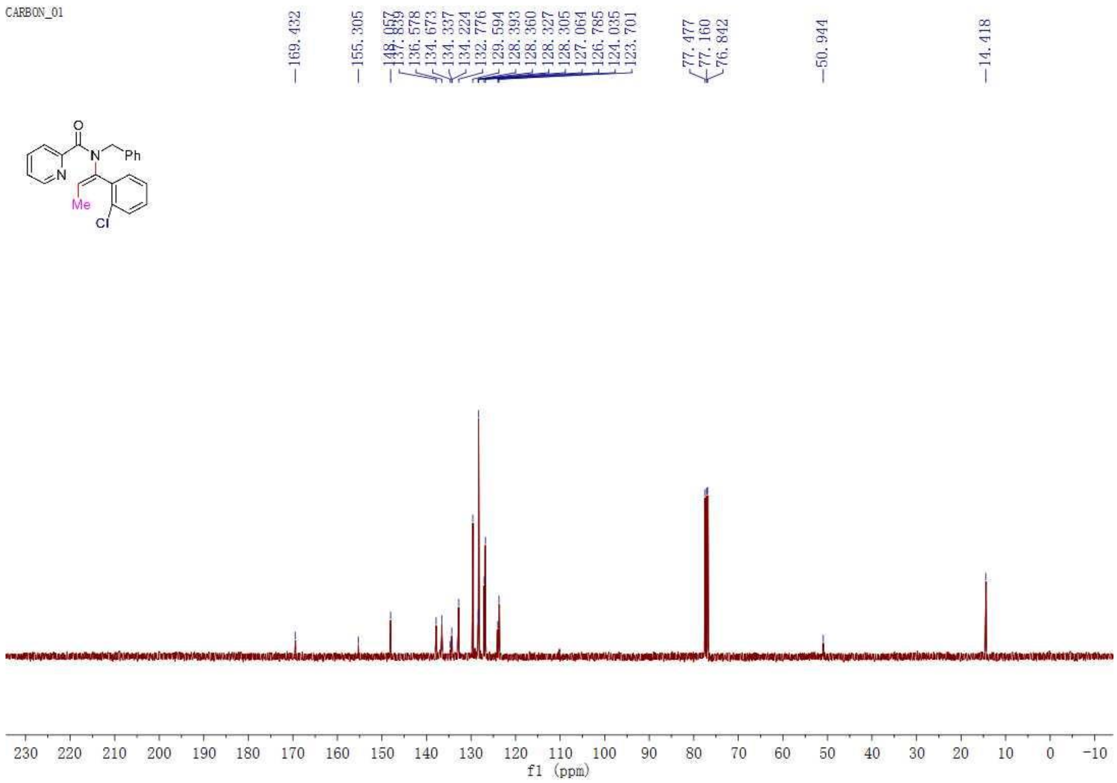

^{13}C NMR spectra for compound **3j** (100 MHz, $CDCl_3$)

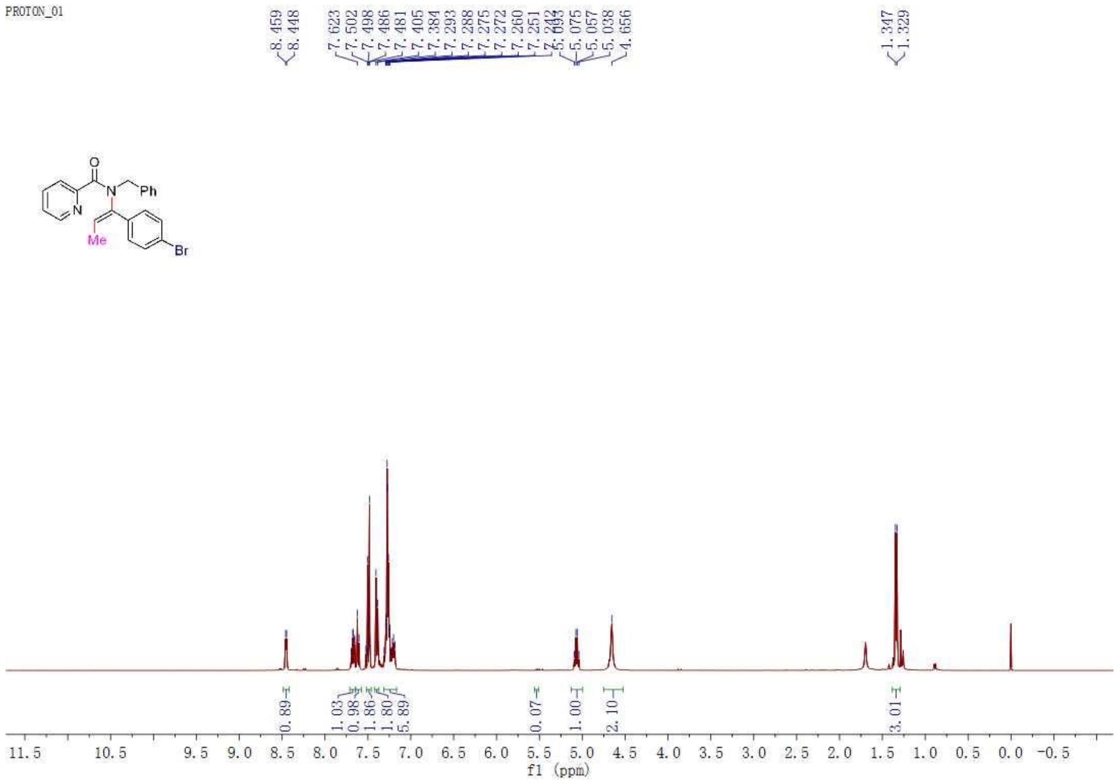

^{1}H NMR spectra for compound **3k** (400 MHz, $CDCl_3$)

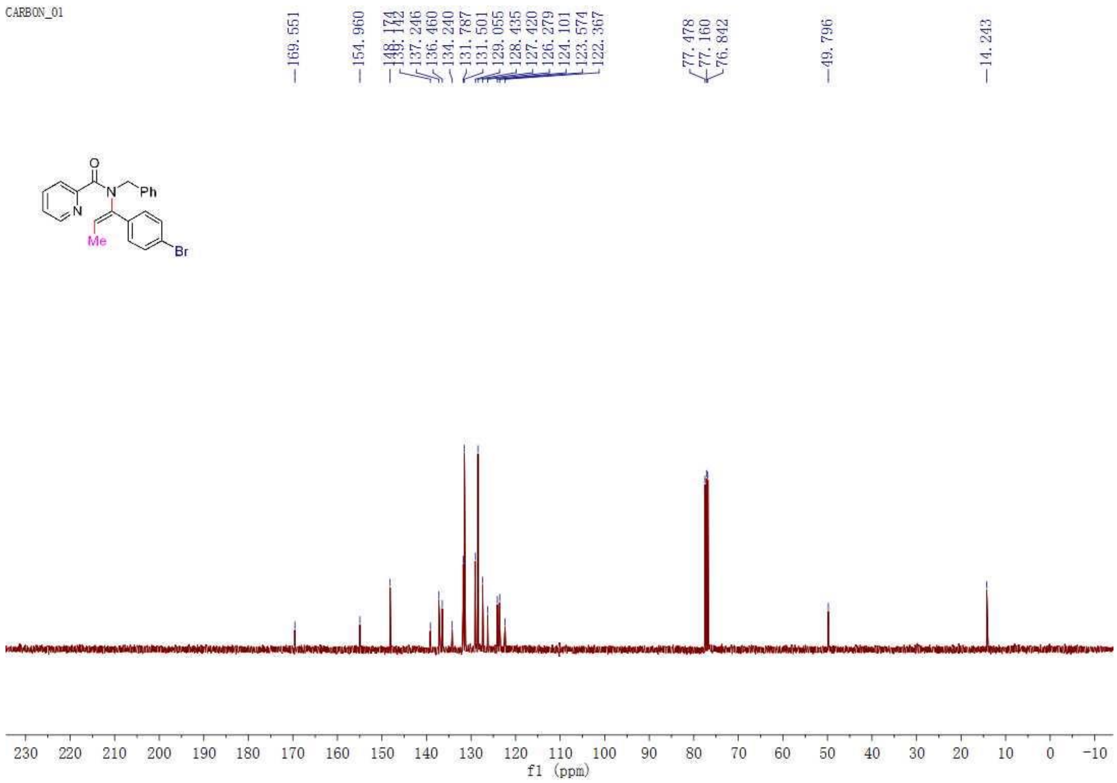

^{13}C NMR spectra for compound **3k** (100 MHz, $CDCl_3$)

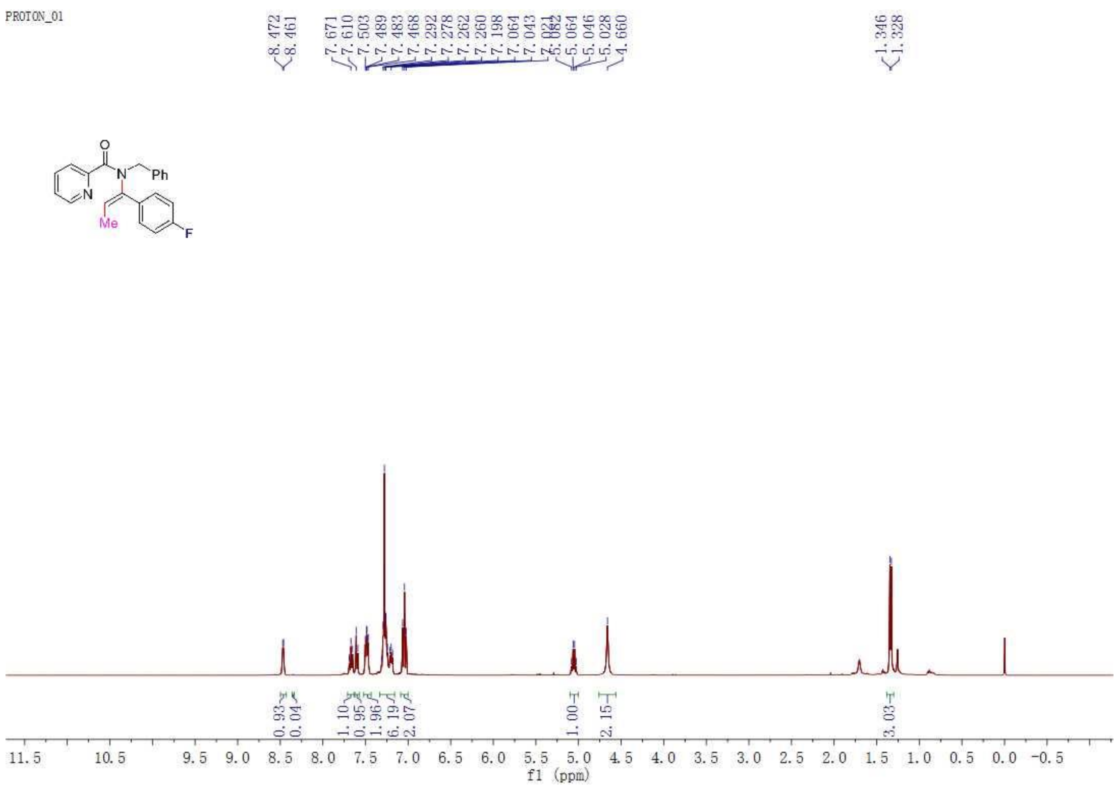

^{1}H NMR spectra for compound **3l** (400 MHz, $CDCl_3$)

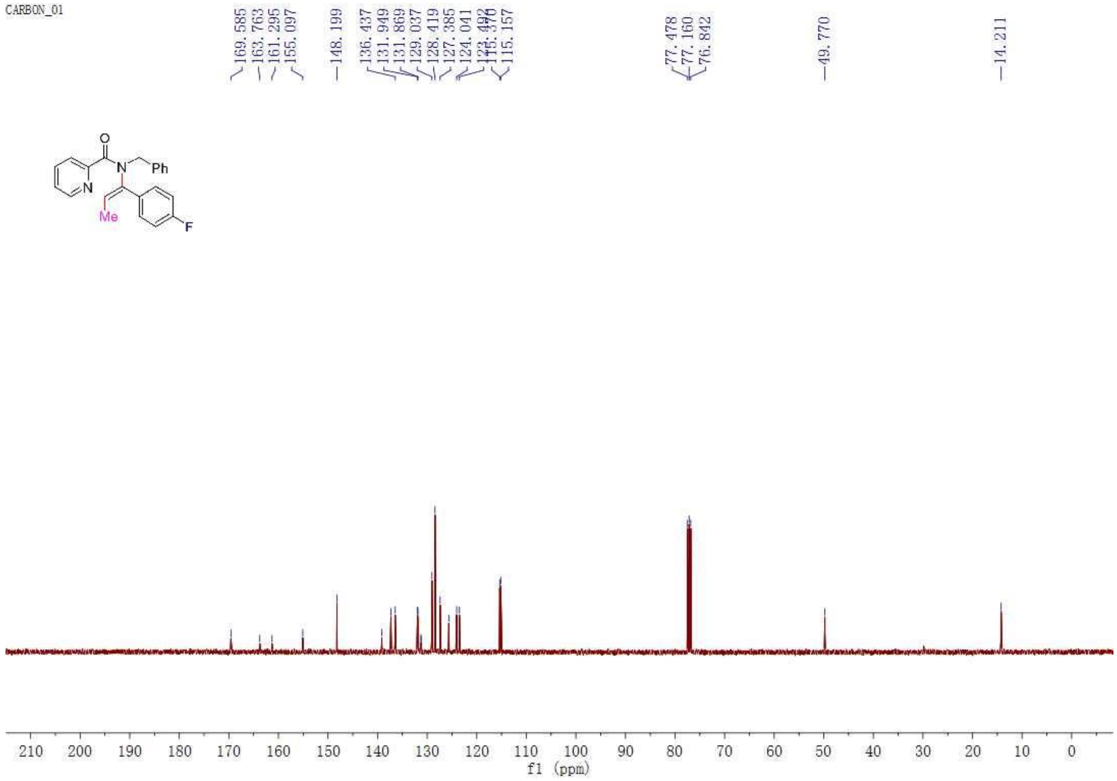

^{13}C NMR spectra for compound **3l** (100 MHz, $CDCl_3$)

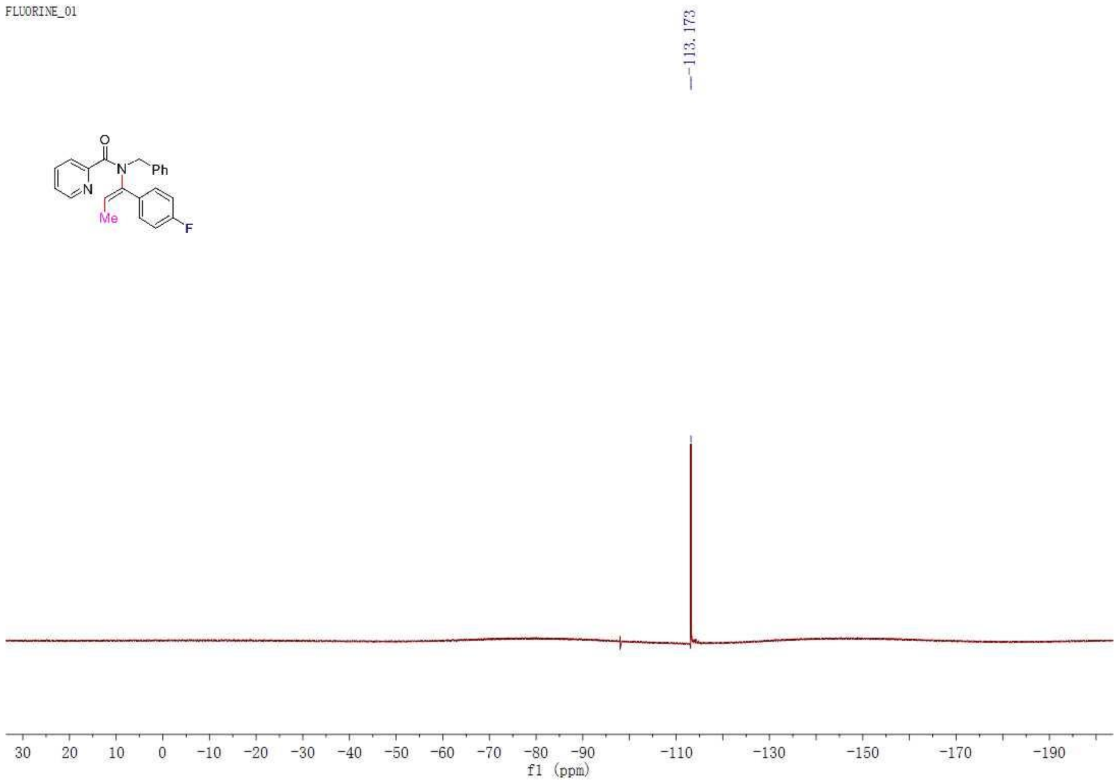

^{19}F NMR spectra for compound **3l** (376 MHz, $CDCl_3$)

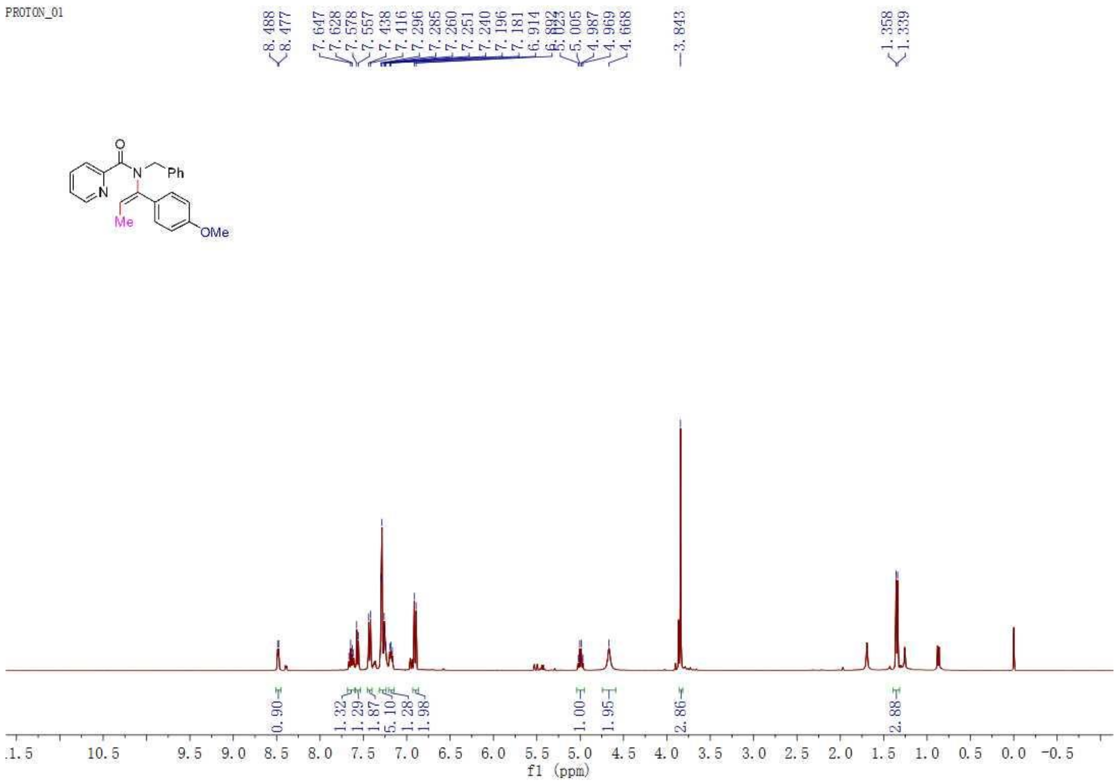

^{1}H NMR spectra for compound **3m** (400 MHz, $CDCl_3$)

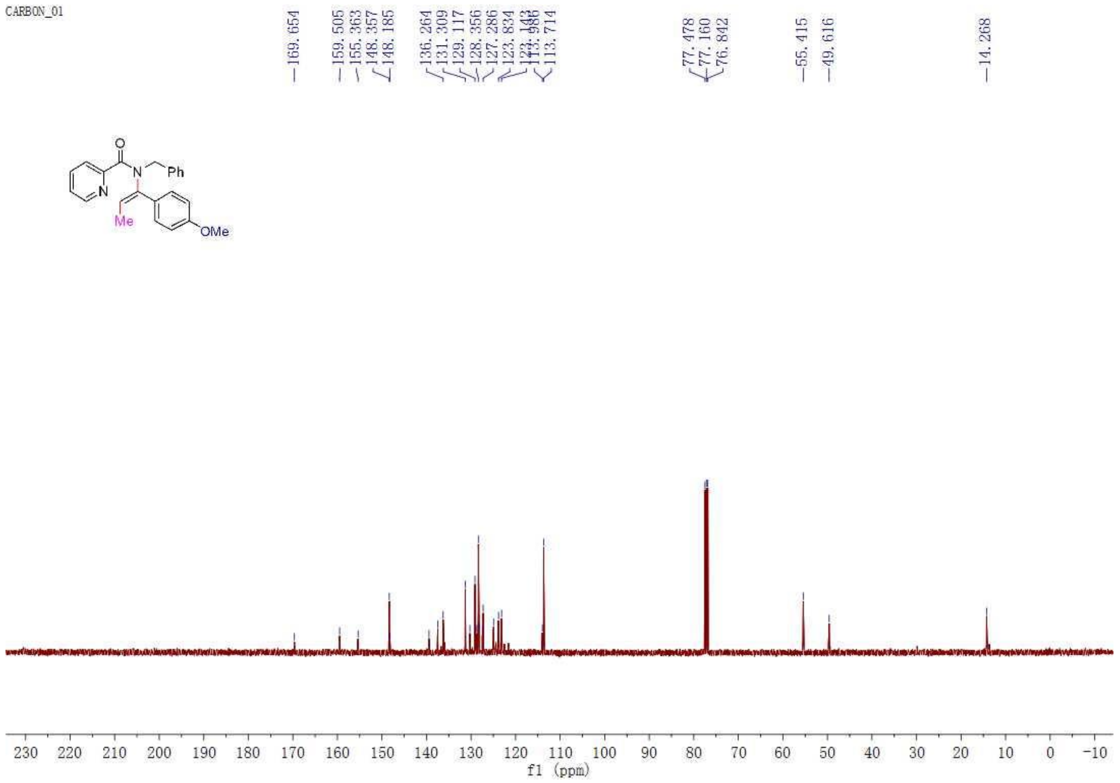

^{13}C NMR spectra for compound **3m** (100 MHz, $CDCl_3$)

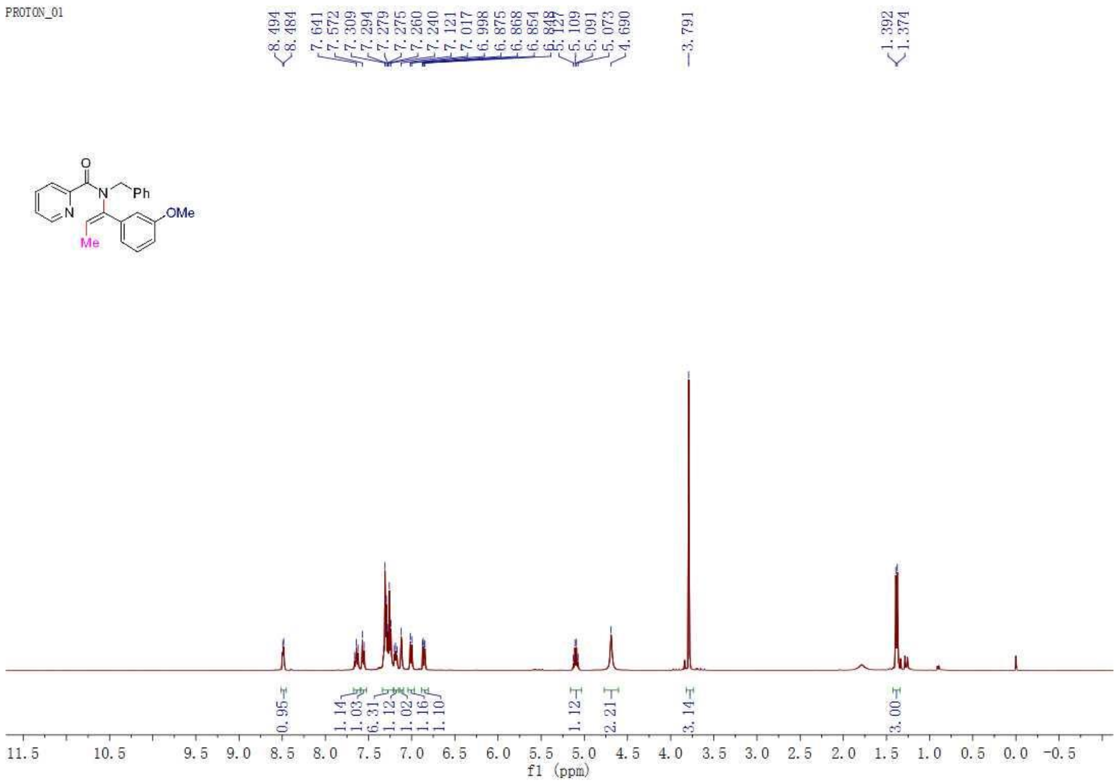

^{1}H NMR spectra for compound **3n** (400 MHz, $CDCl_3$)

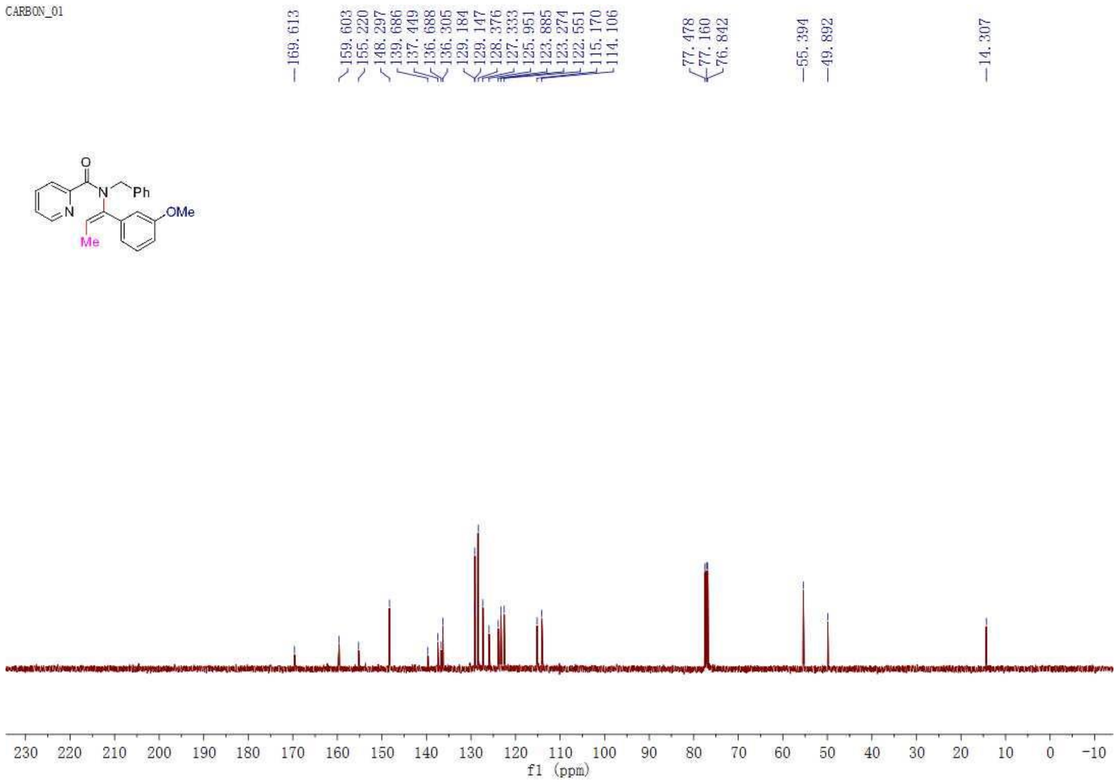

^{13}C NMR spectra for compound **3n** (100 MHz, $CDCl_3$)

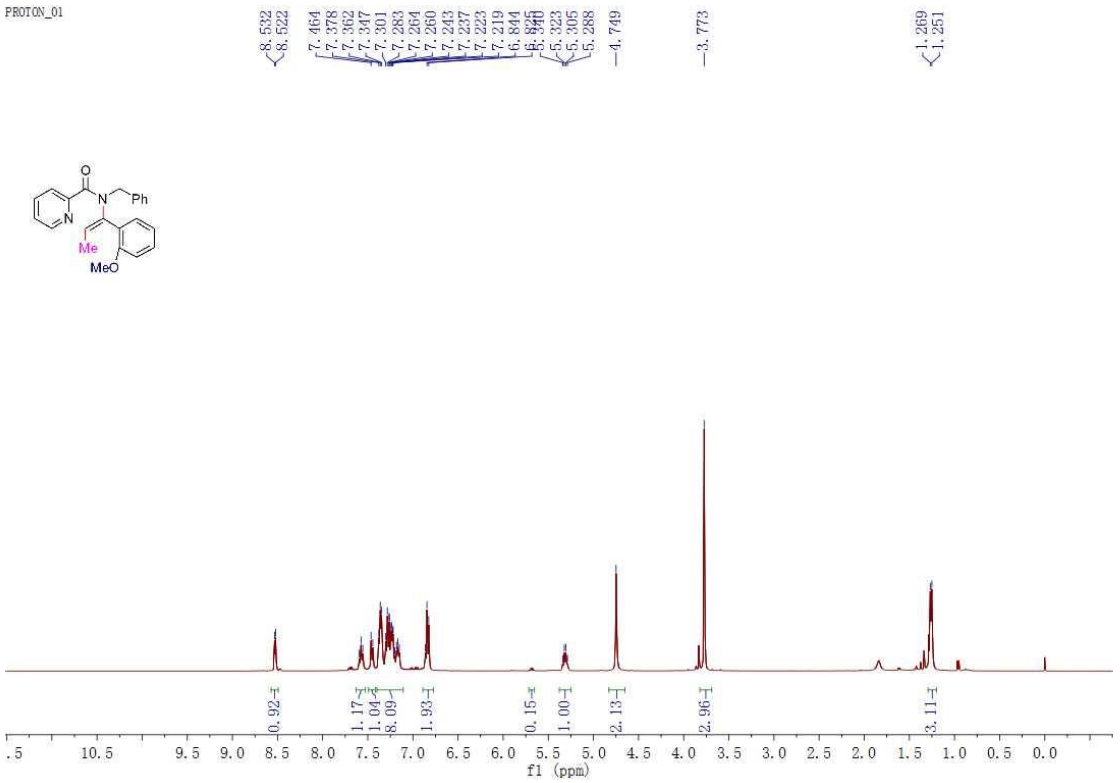

^{1}H NMR spectra for compound **3o** (400 MHz, $CDCl_3$)

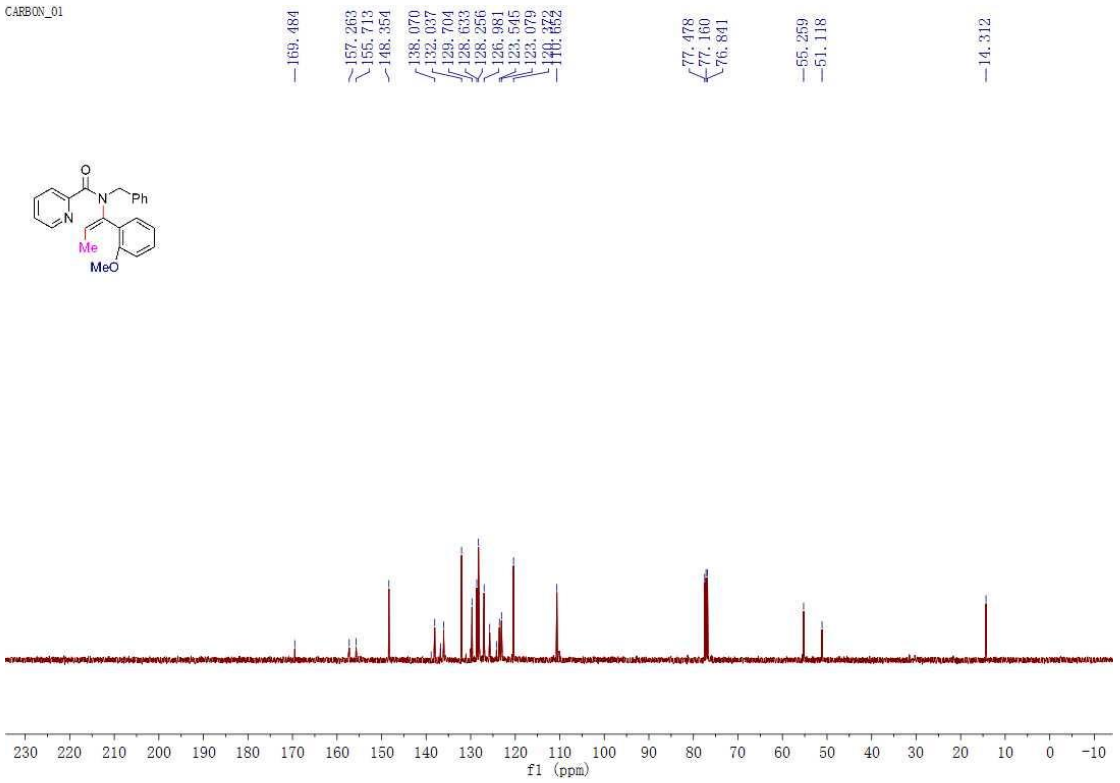

^{13}C NMR spectra for compound **3o** (100 MHz, $CDCl_3$)

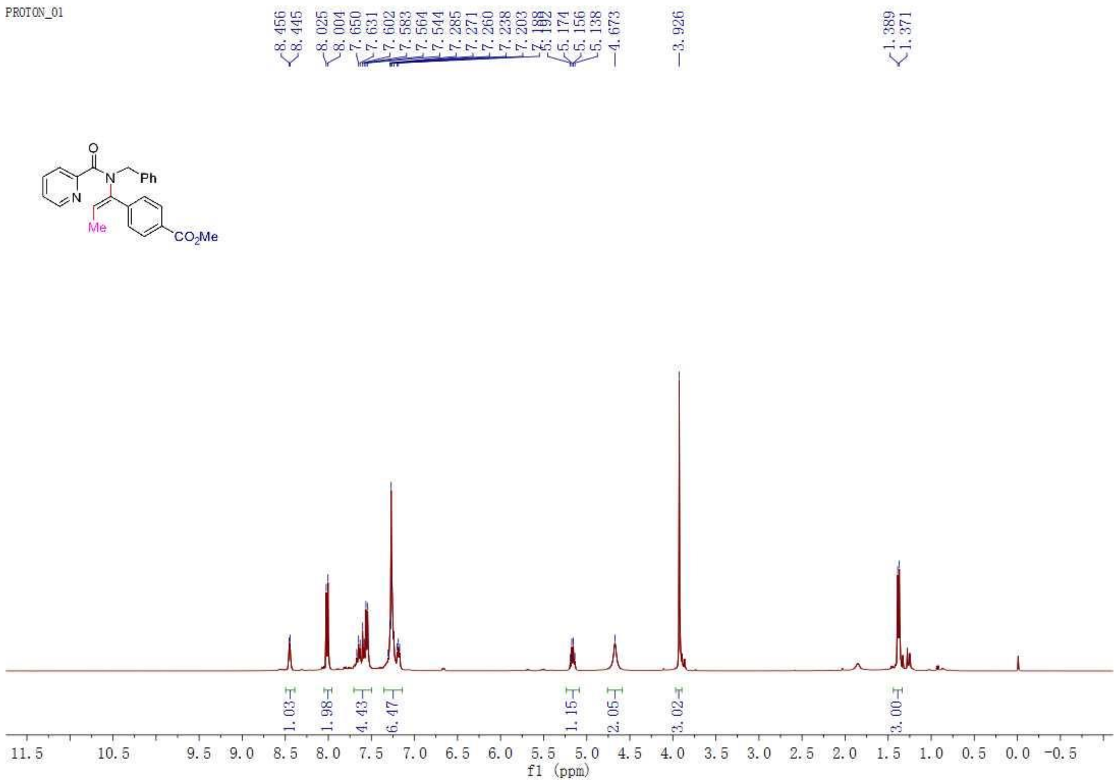

^{1}H NMR spectra for compound **3p** (400 MHz, $CDCl_3$)

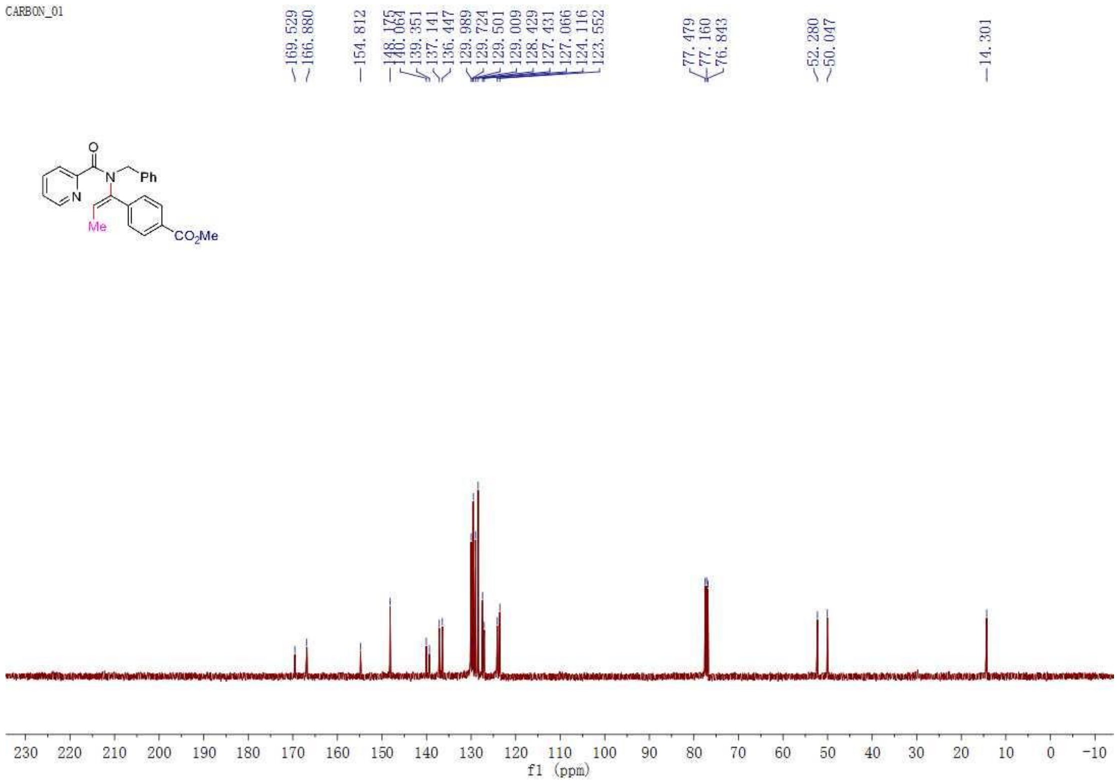

^{13}C NMR spectra for compound **3p** (100 MHz, $CDCl_3$)

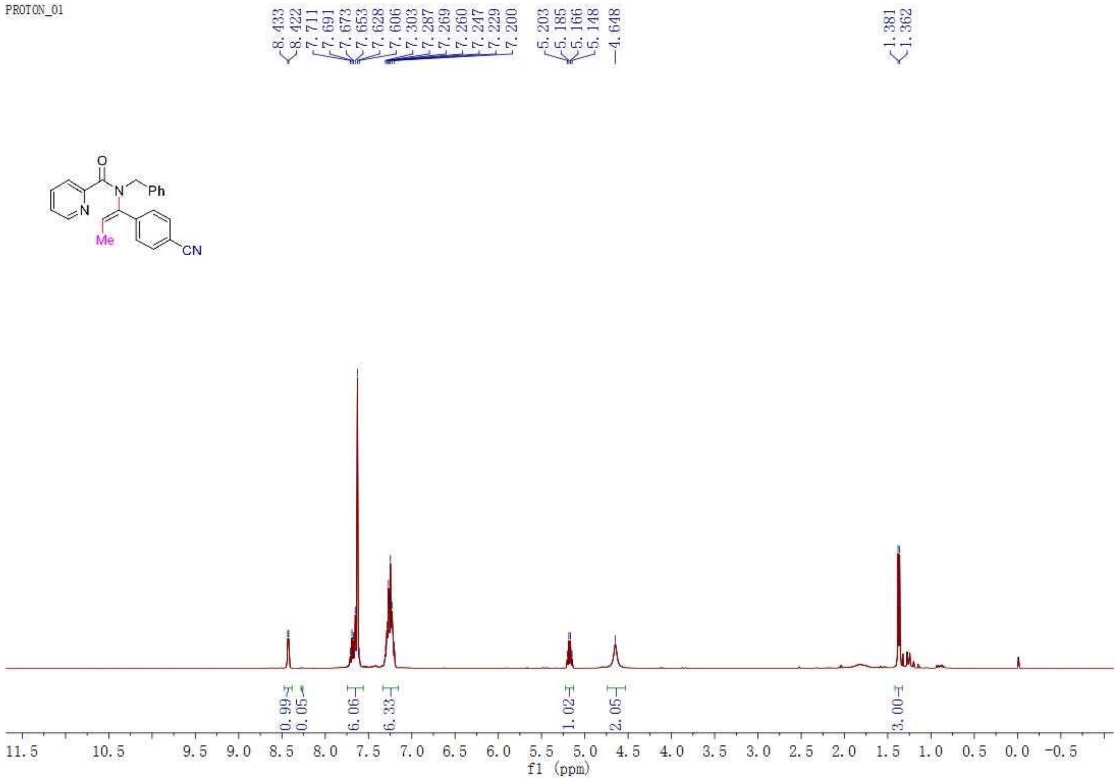

^{1}H NMR spectra for compound **3q** (400 MHz, $CDCl_3$)

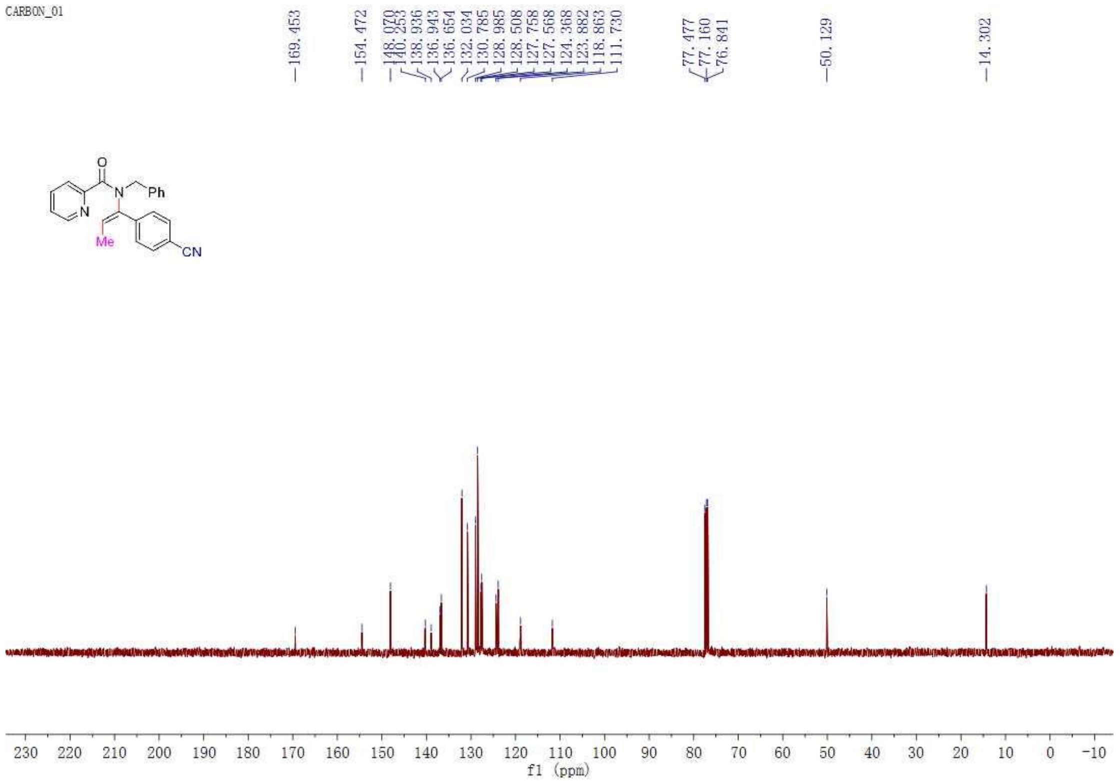

^{13}C NMR spectra for compound **3q** (100 MHz, $CDCl_3$)

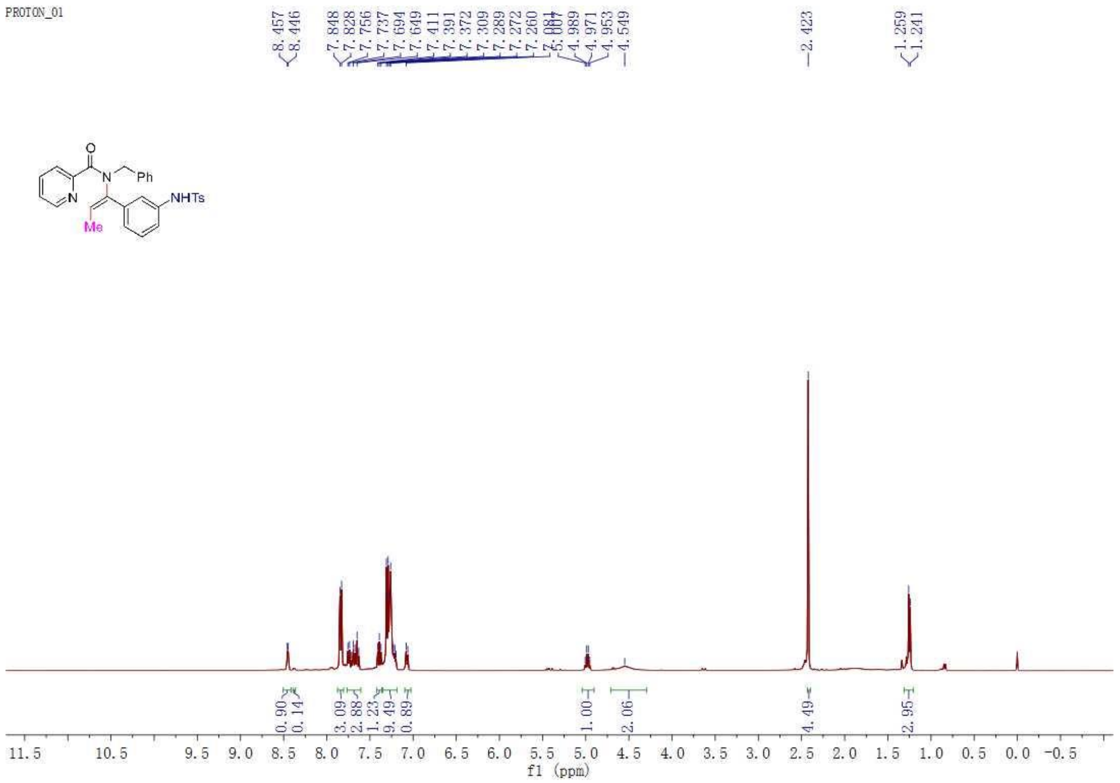

^{1}H NMR spectra for compound **3r** (400 MHz, $CDCl_3$)

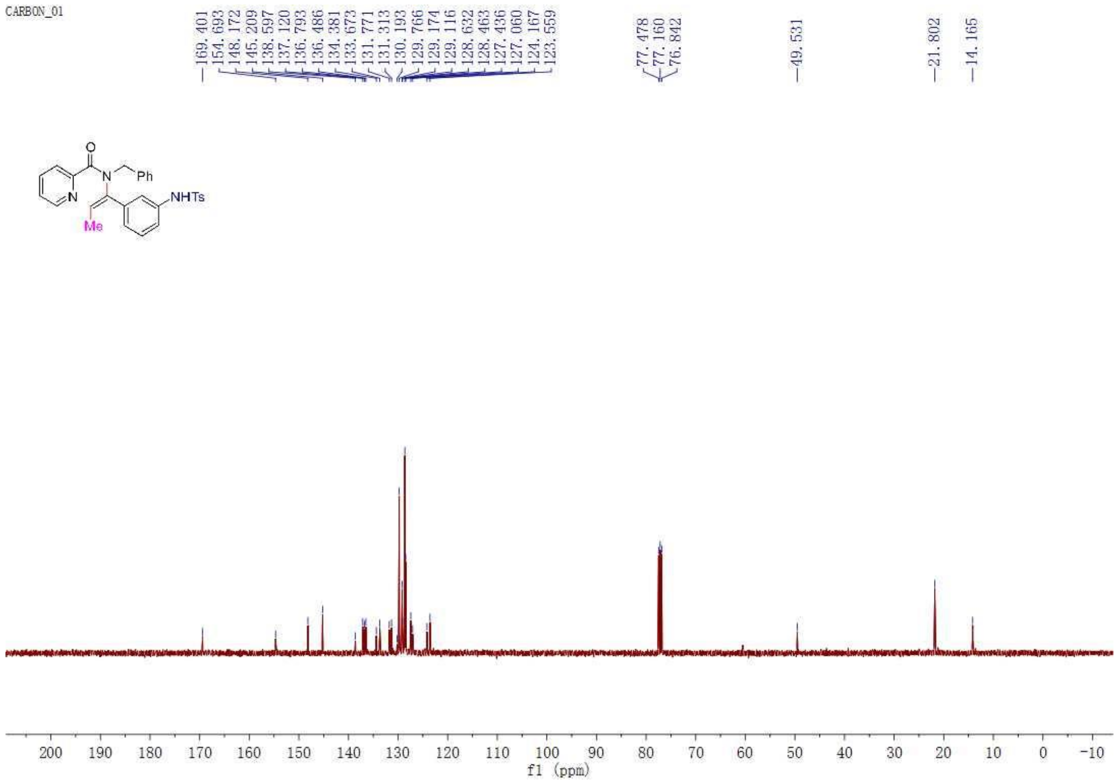

^{13}C NMR spectra for compound **3r** (100 MHz, $CDCl_3$)

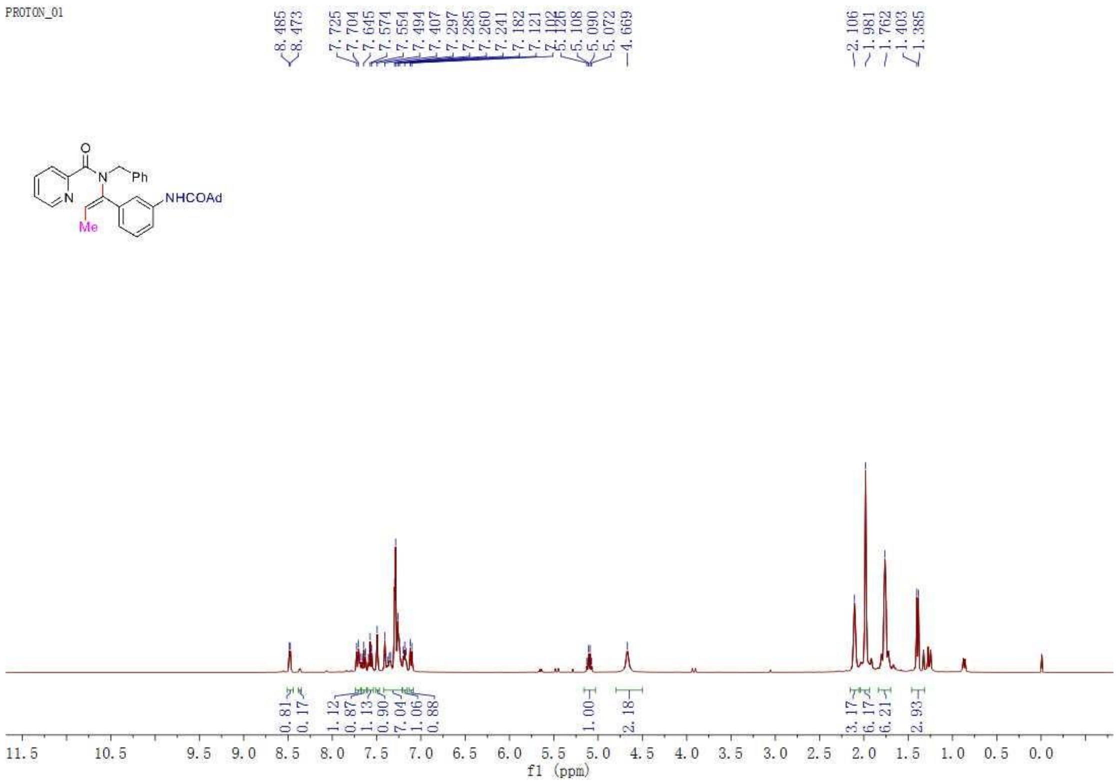

^{1}H NMR spectra for compound **3s** (400 MHz, $CDCl_3$)

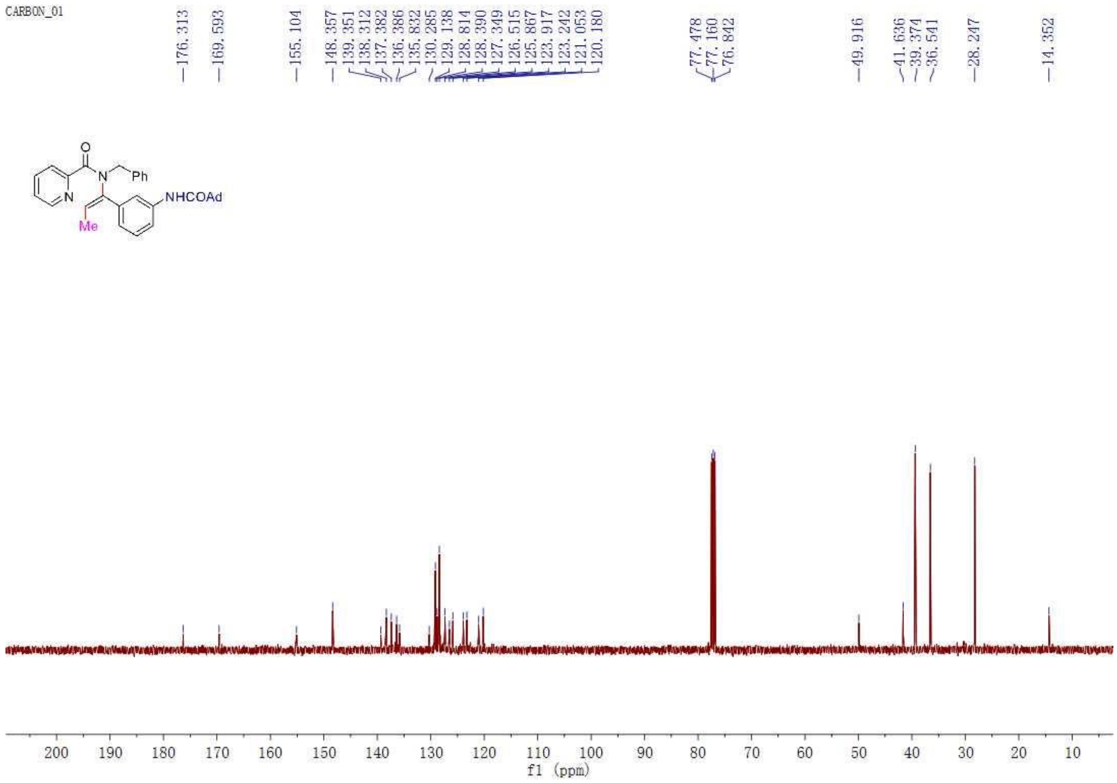

^{13}C NMR spectra for compound **3s** (100 MHz, $CDCl_3$)

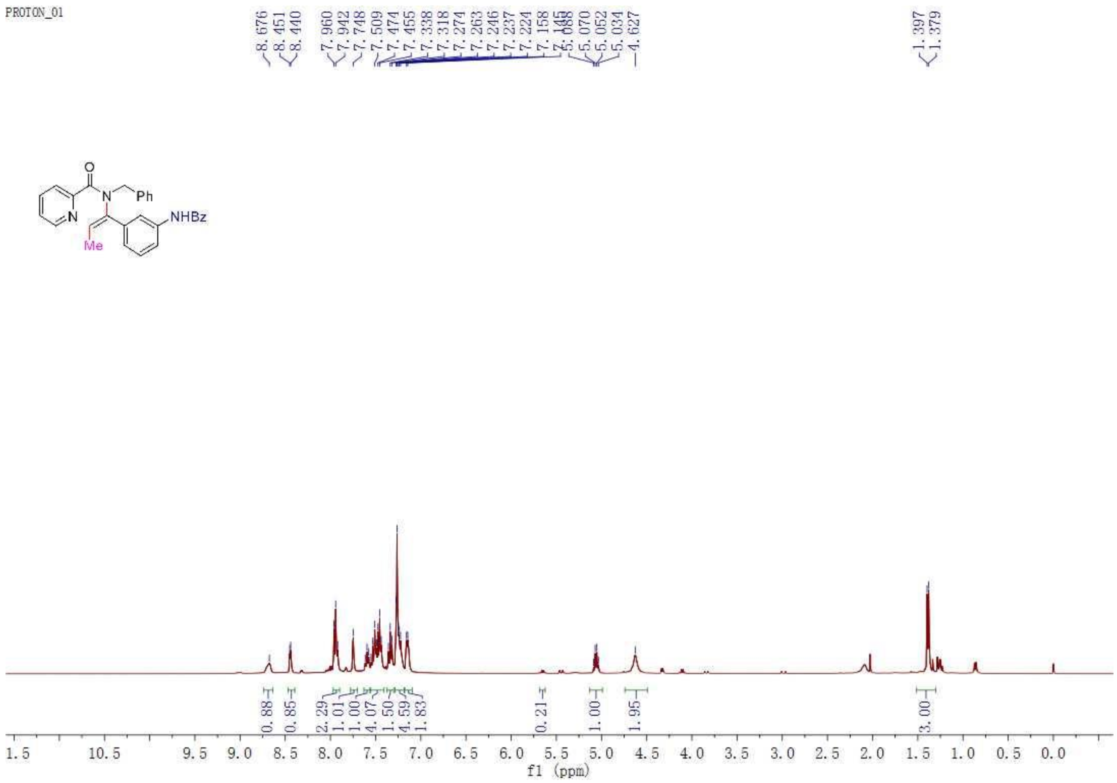

^{1}H NMR spectra for compound **3t** (400 MHz, $CDCl_3$)

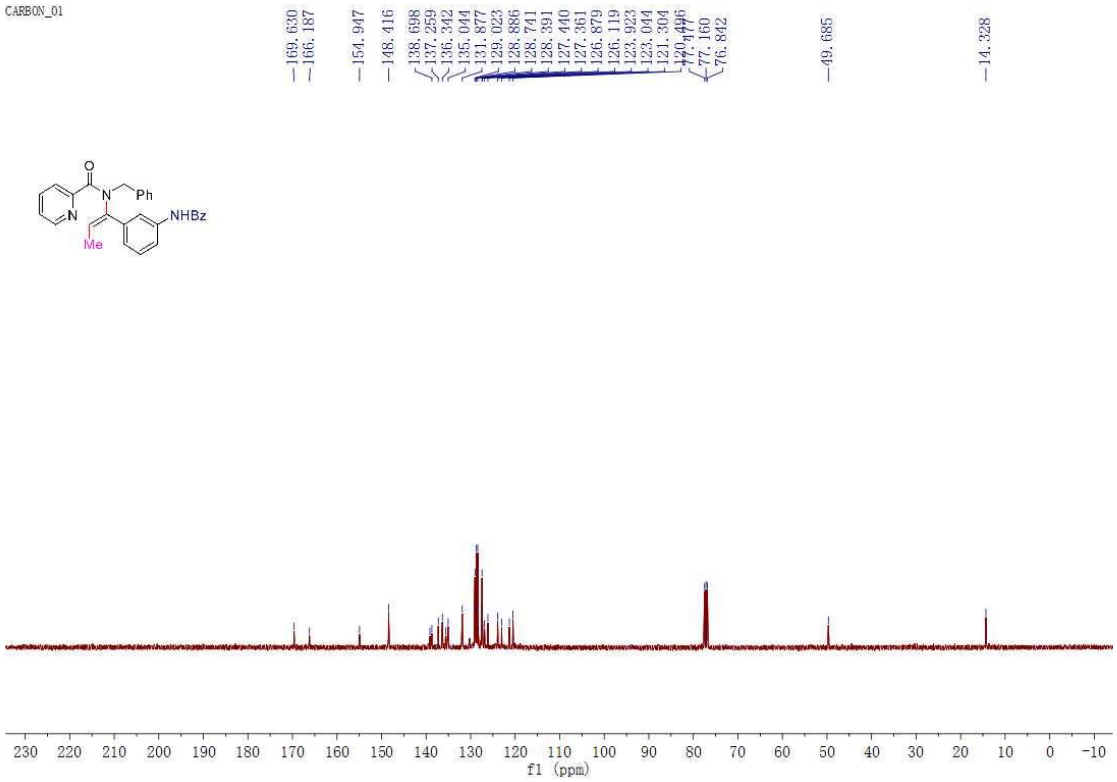

^{13}C NMR spectra for compound **3t** (100 MHz, $CDCl_3$)

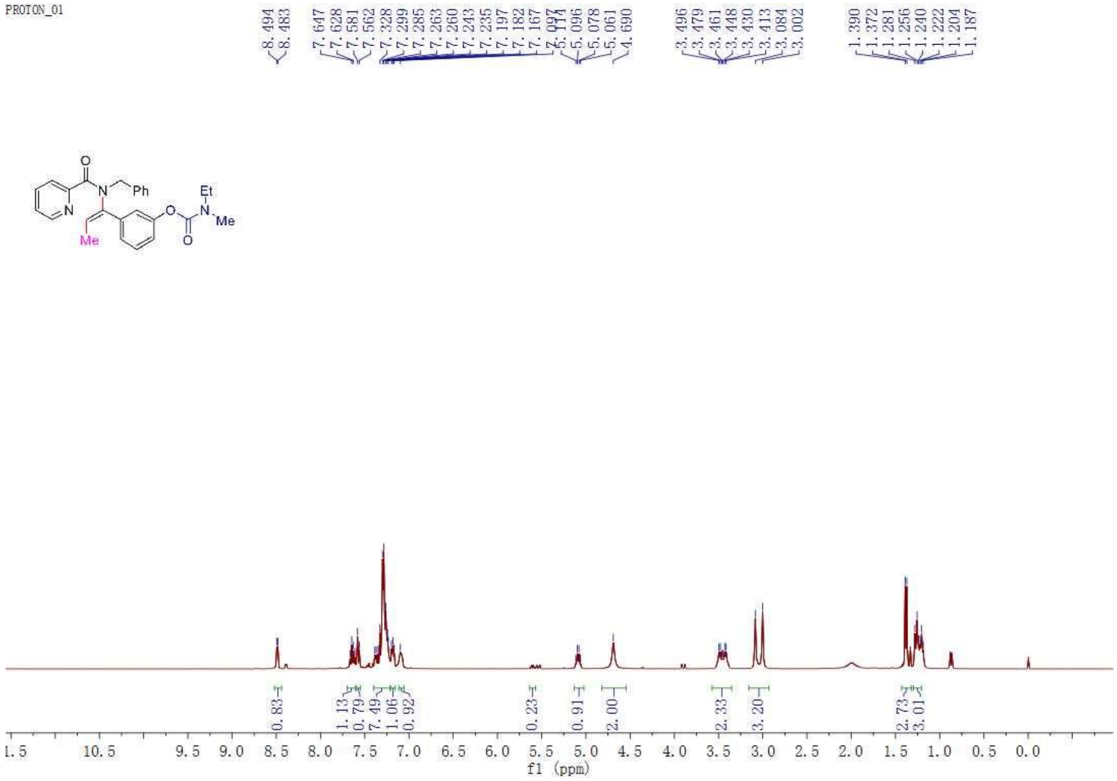

^{1}H NMR spectra for compound **3u** (400 MHz, $CDCl_3$)

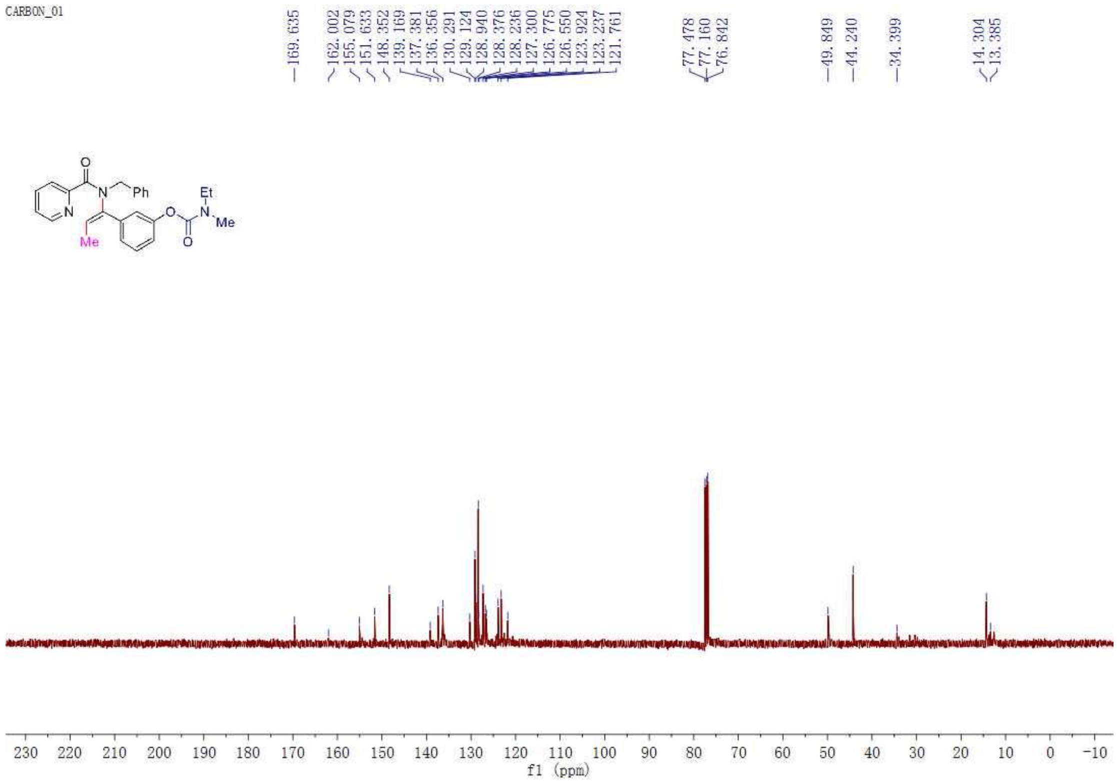

^{13}C NMR spectra for compound **3u** (100 MHz, $CDCl_3$)

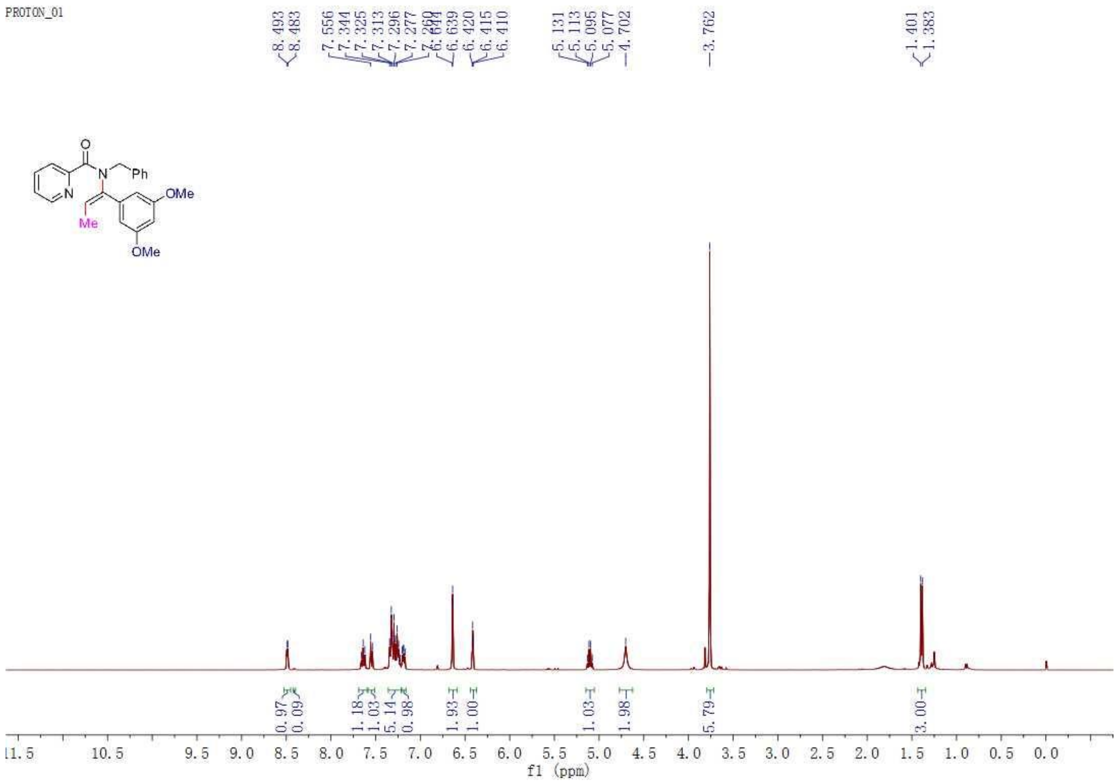

^{1}H NMR spectra for compound **3v** (400 MHz, $CDCl_3$)

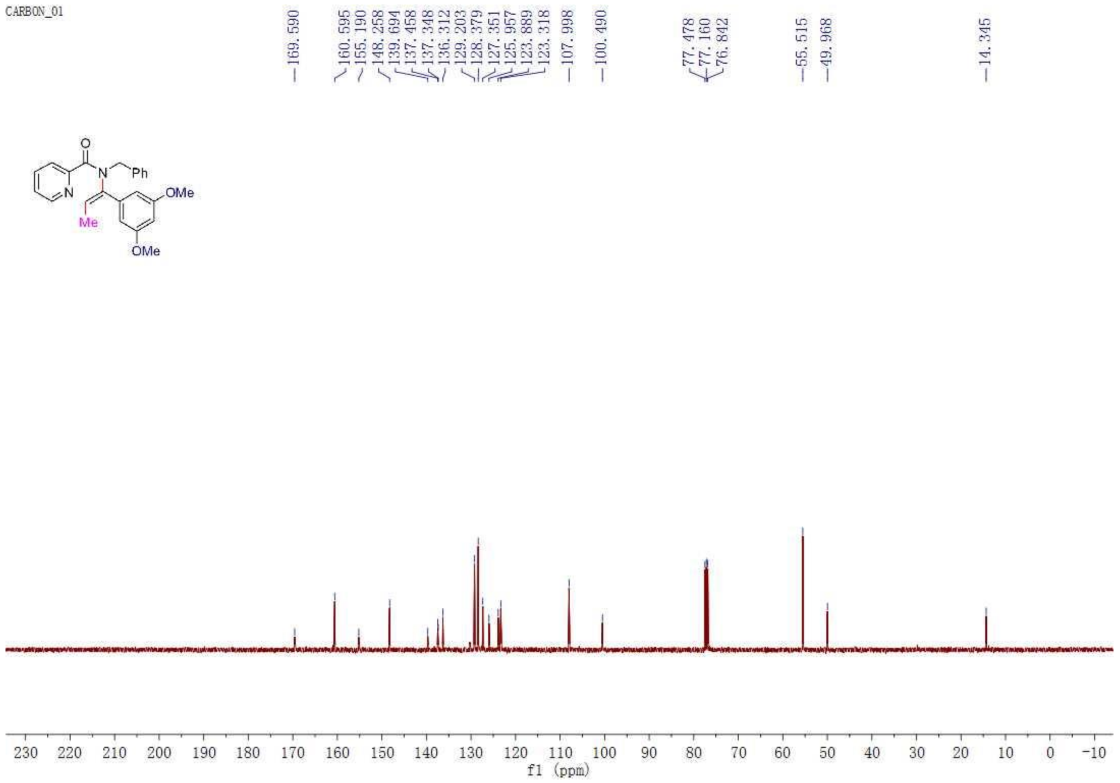

^{13}C NMR spectra for compound **3v** (100 MHz, $CDCl_3$)

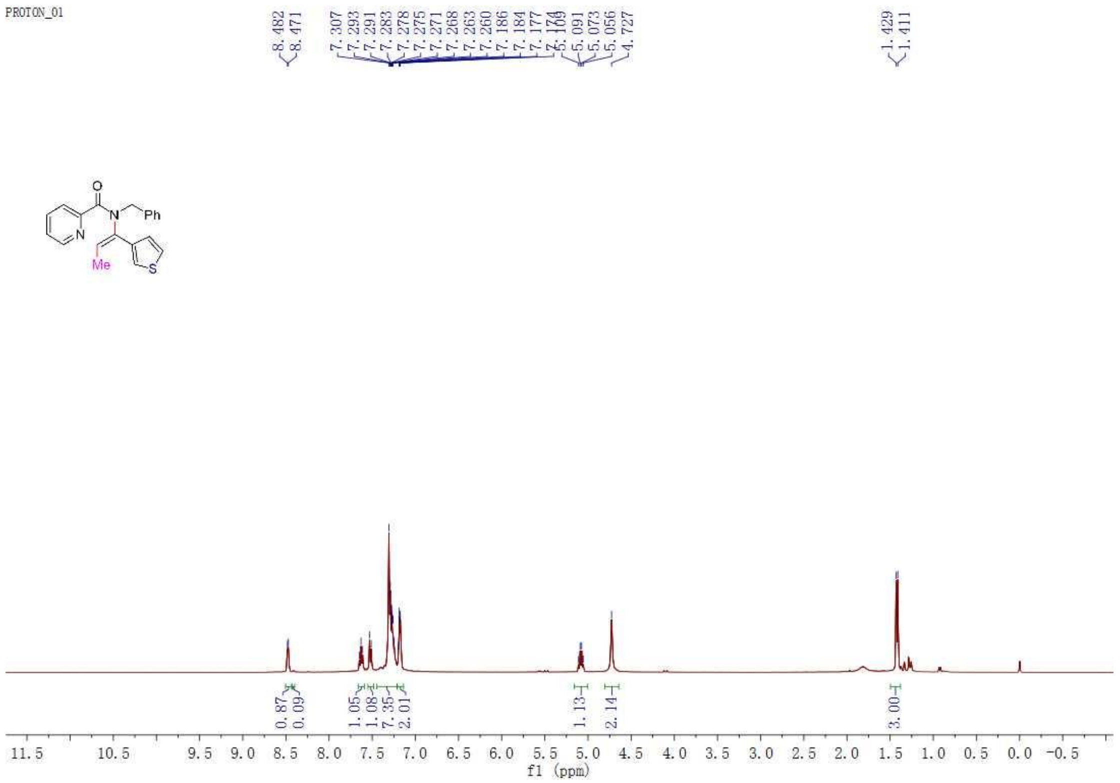

^{1}H NMR spectra for compound **3w** (400 MHz, $CDCl_3$)

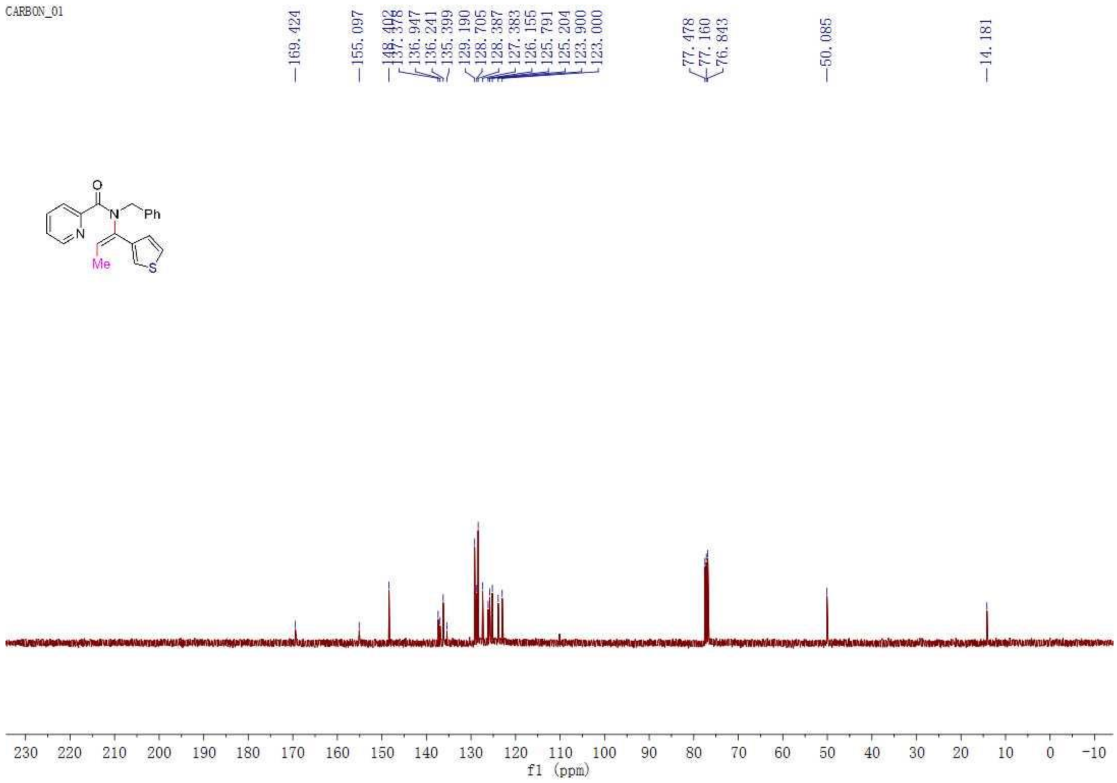

^{13}C NMR spectra for compound **3w** (100 MHz, $CDCl_3$)

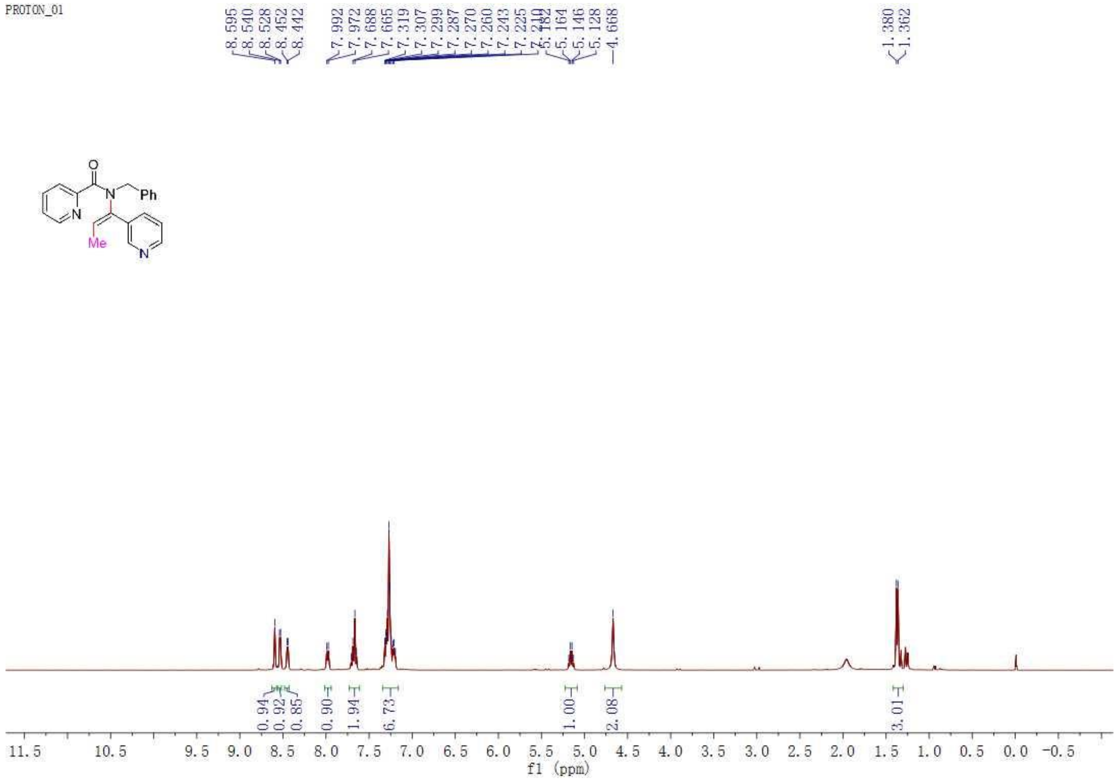

^{1}H NMR spectra for compound **3x** (400 MHz, $CDCl_3$)

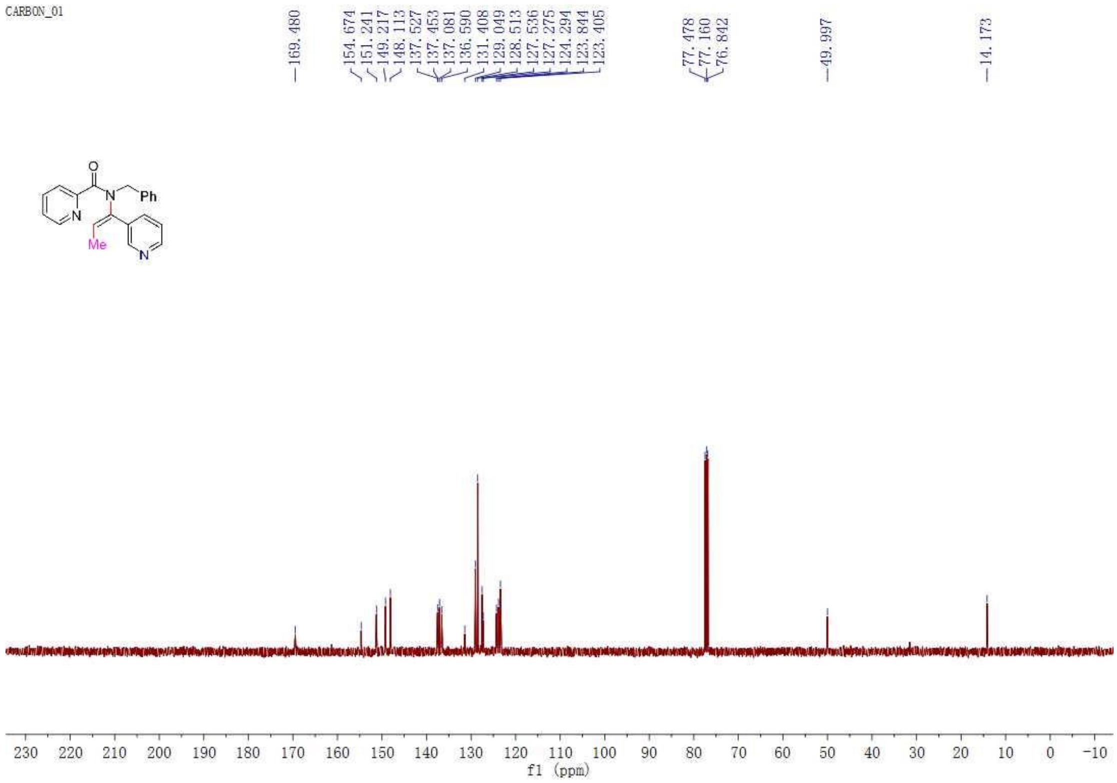

^{13}C NMR spectra for compound **3x** (100 MHz, $CDCl_3$)

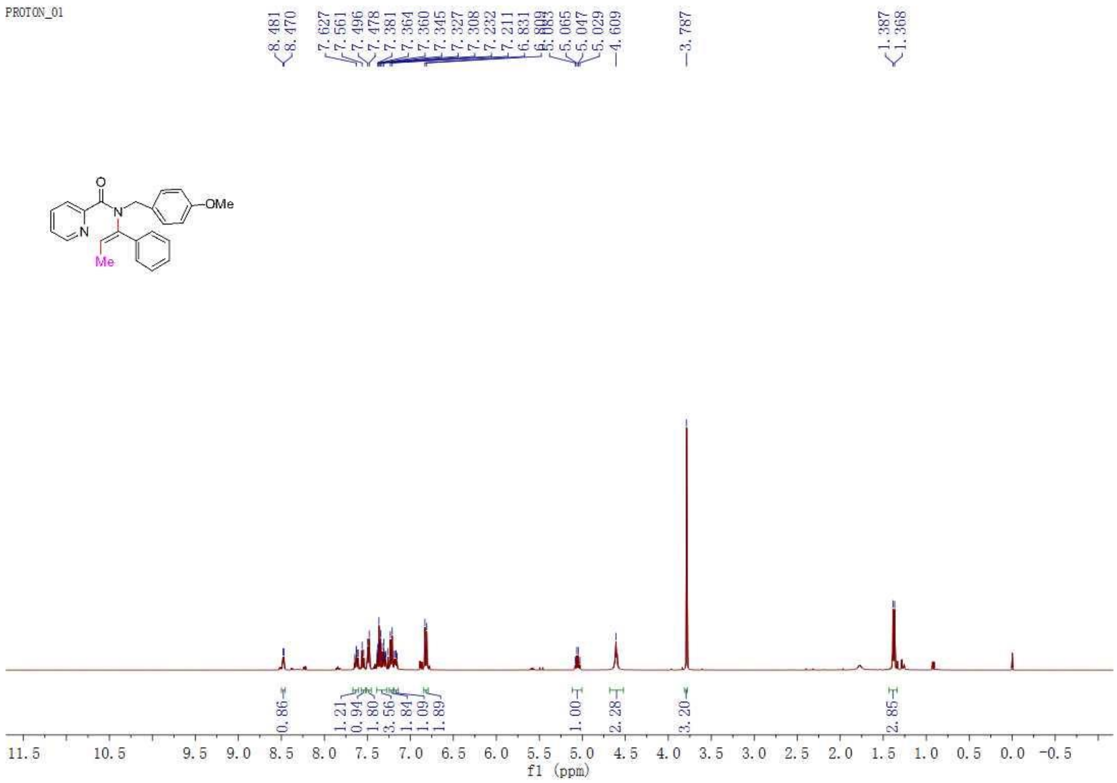

^{1}H NMR spectra for compound **4a** (400 MHz, $CDCl_3$)

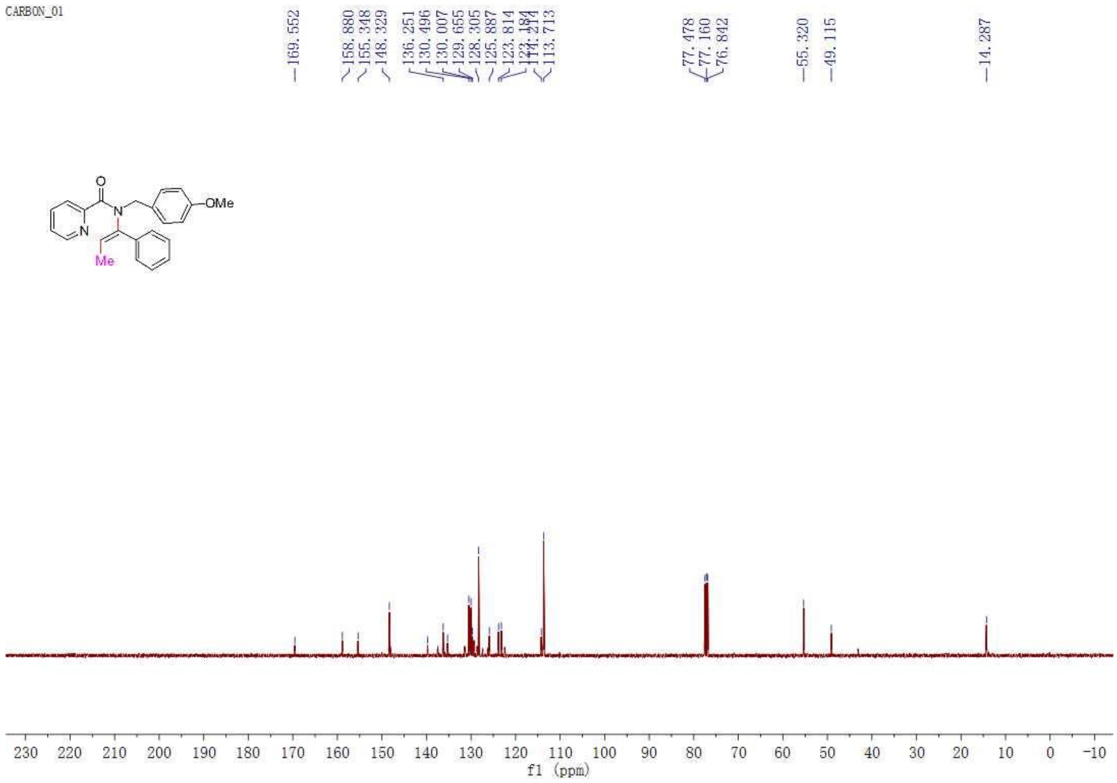

^{13}C NMR spectra for compound **4a** (100 MHz, $CDCl_3$)

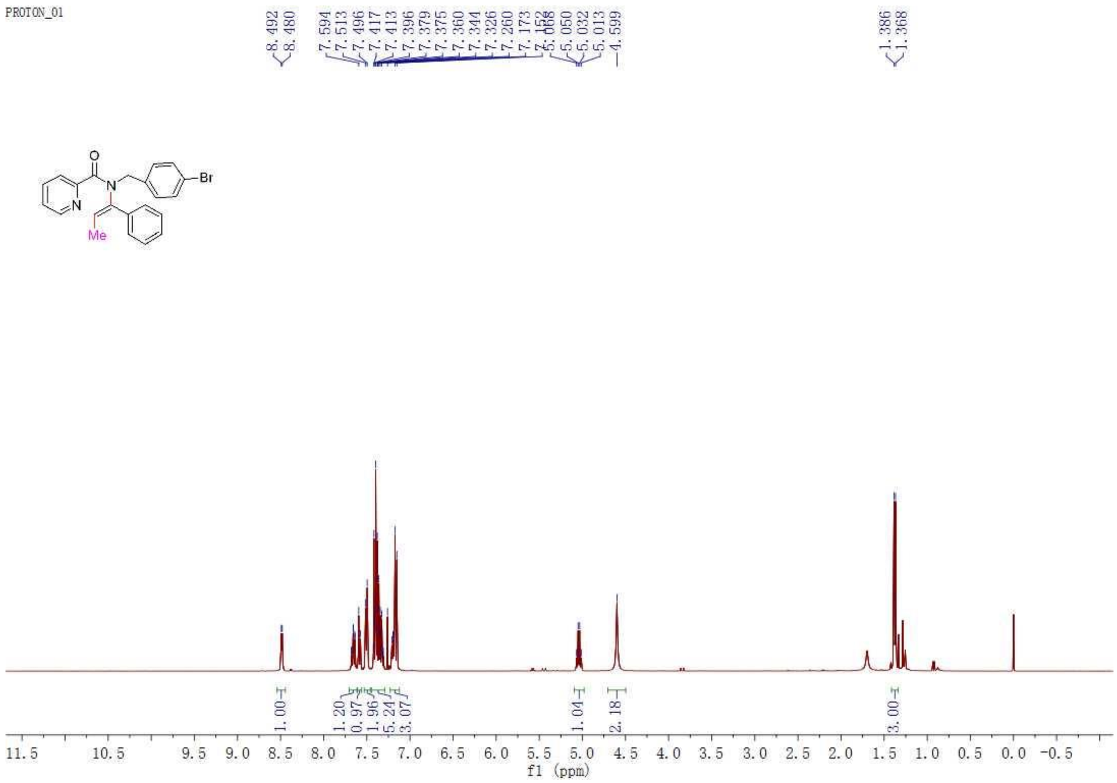

^{1}H NMR spectra for compound **4b** (400 MHz, $CDCl_3$)

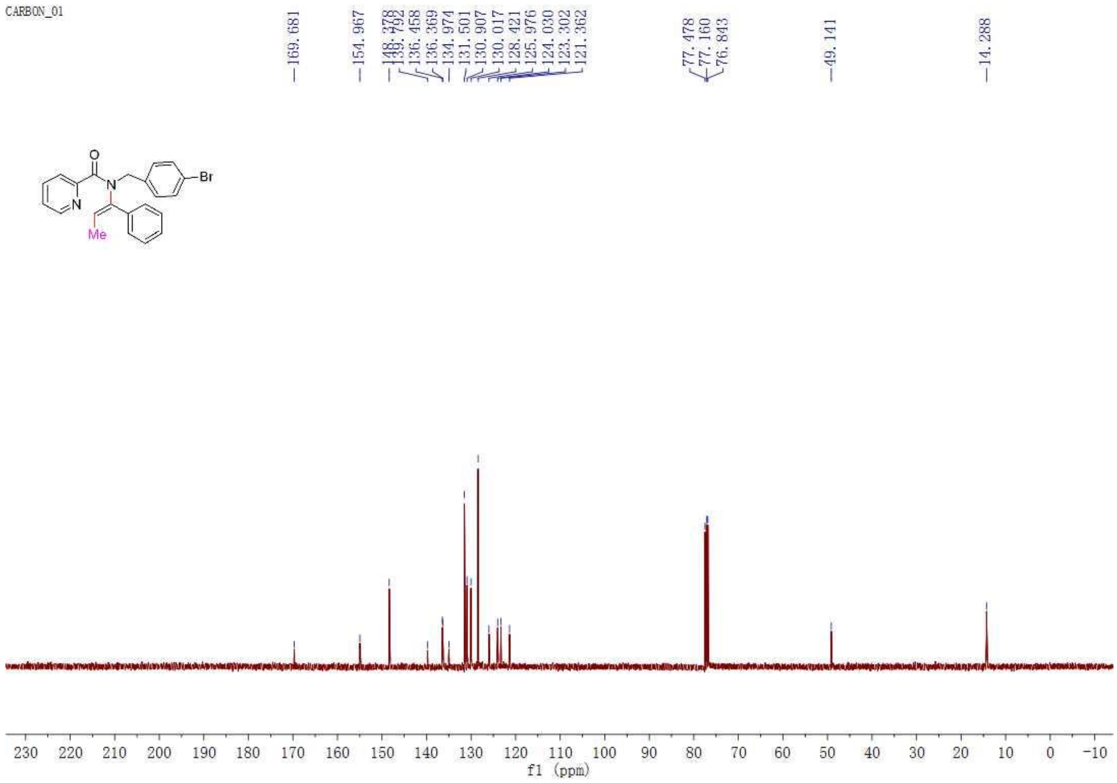

^{13}C NMR spectra for compound **4b** (100 MHz, $CDCl_3$)

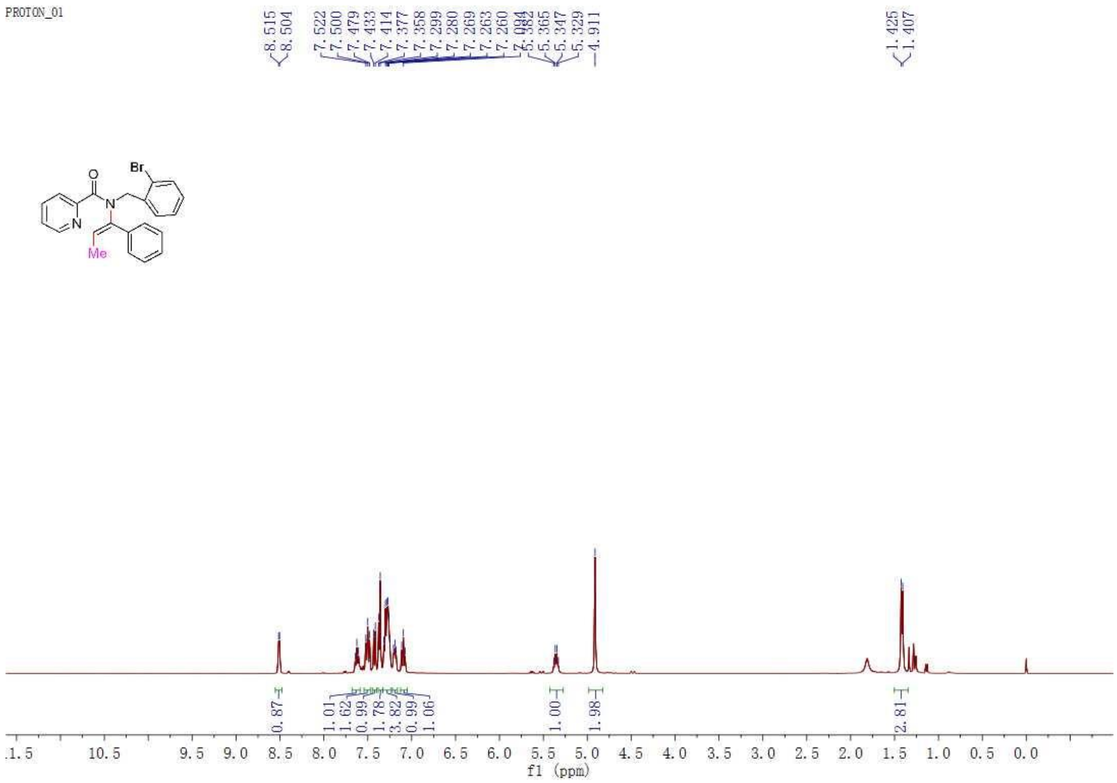

^{1}H NMR spectra for compound **4c** (400 MHz, $CDCl_3$)

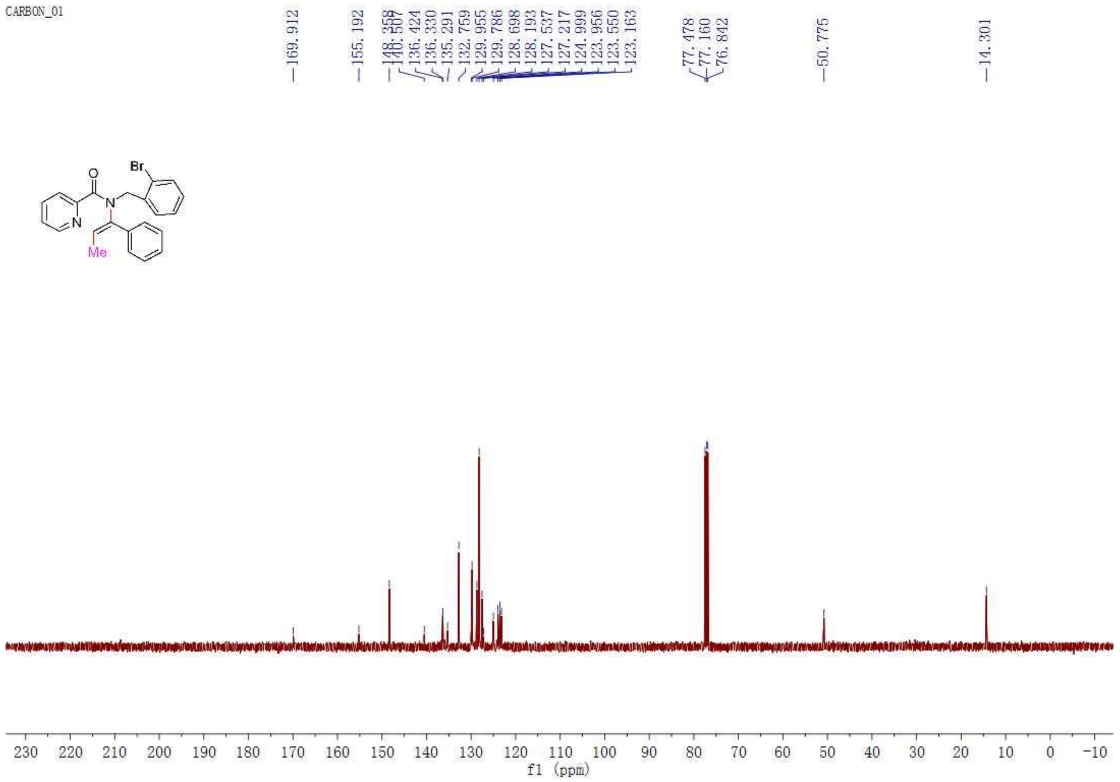

^{13}C NMR spectra for compound **4c** (100 MHz, $CDCl_3$)

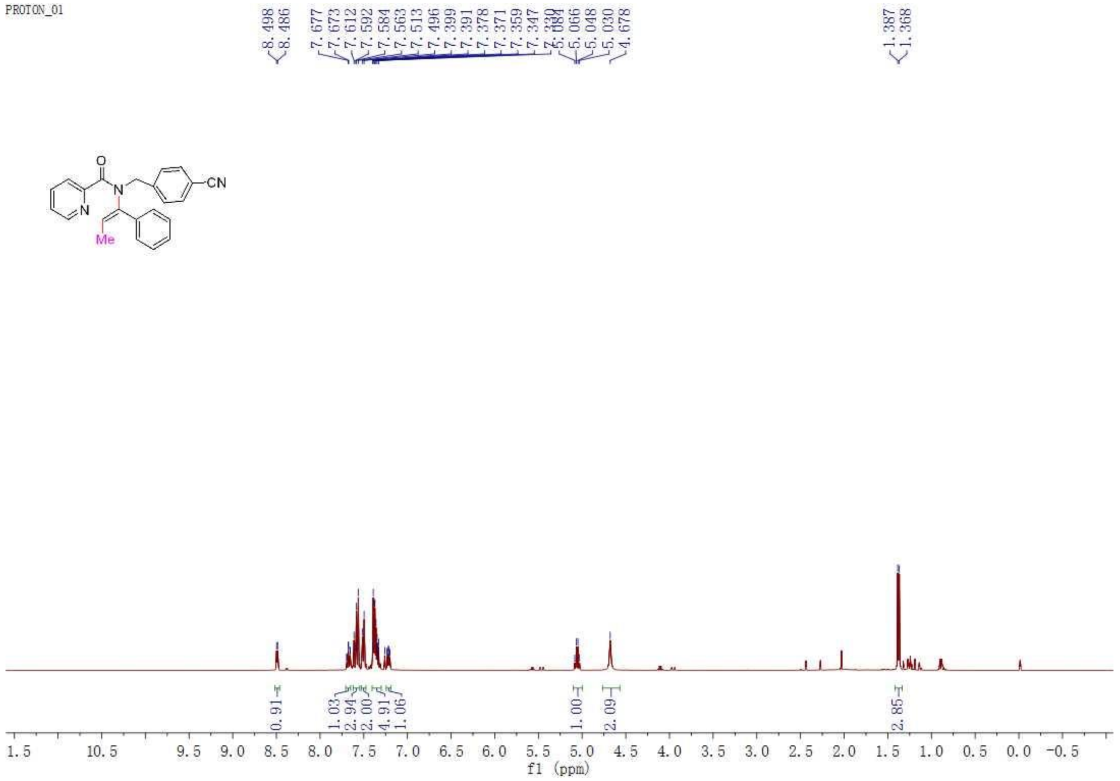

^{1}H NMR spectra for compound **4d** (400 MHz, $CDCl_3$)

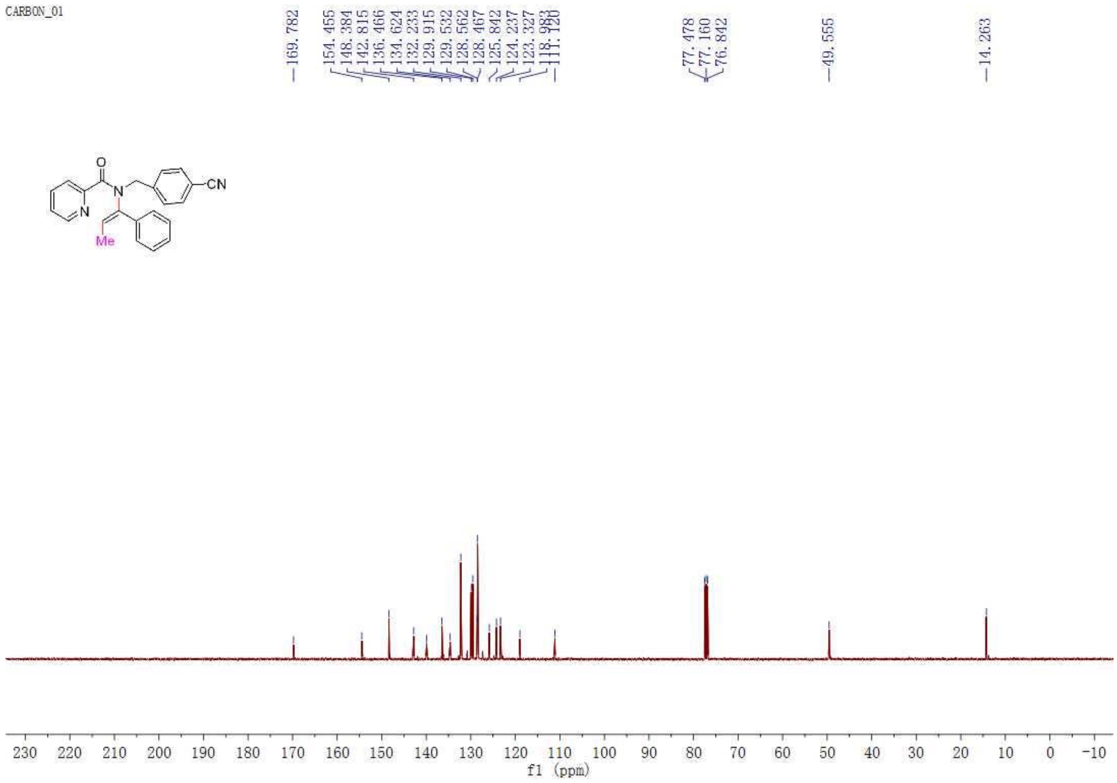

^{13}C NMR spectra for compound **4d** (100 MHz, $CDCl_3$)

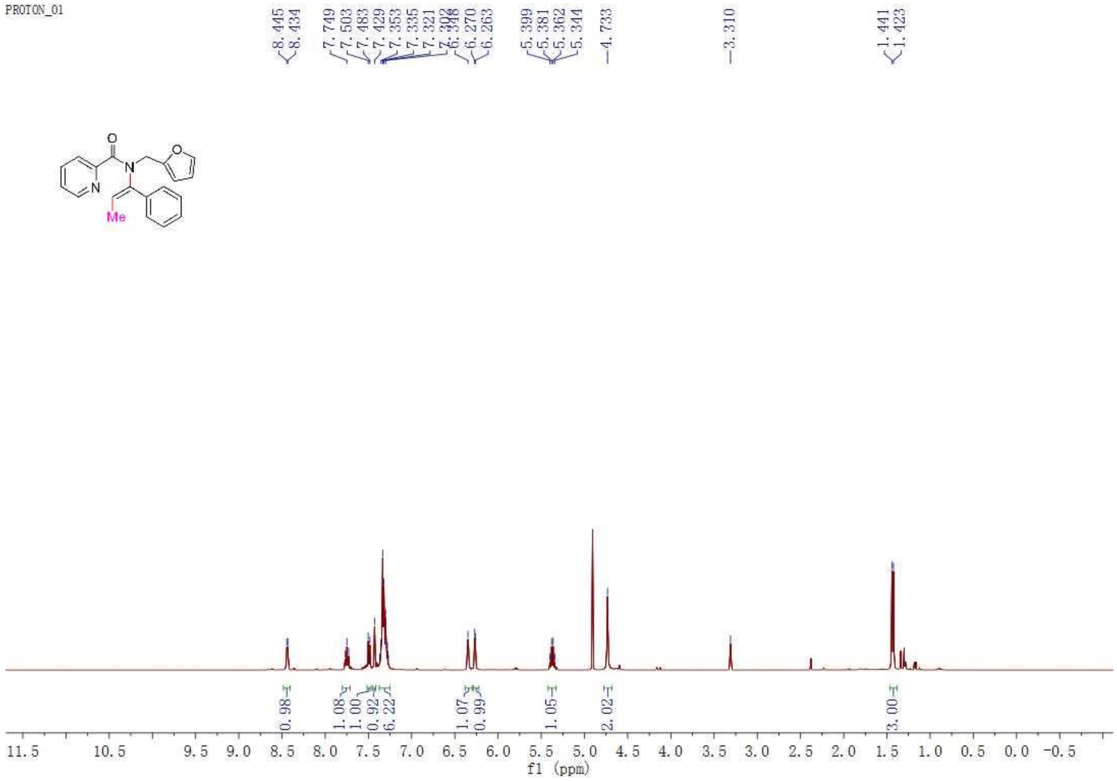

^{1}H NMR spectra for compound **4e** (400 MHz, CD_3OD)

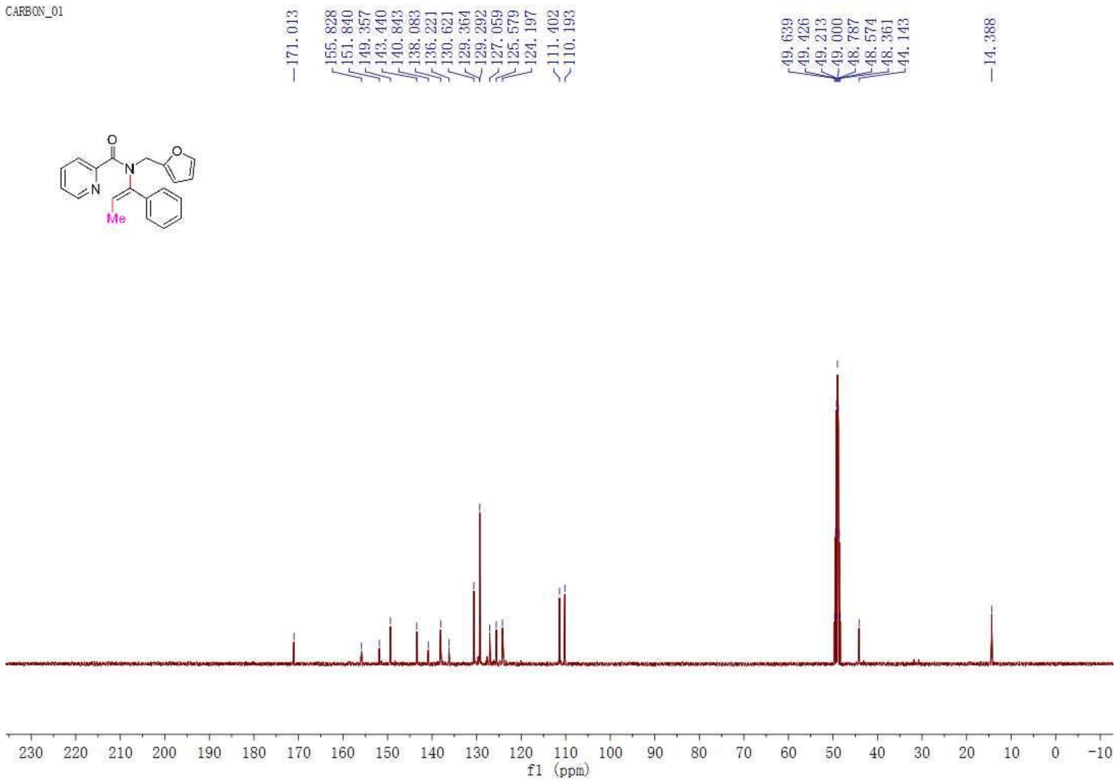

^{13}C NMR spectra for compound **4e** (100 MHz, CD_3OD)

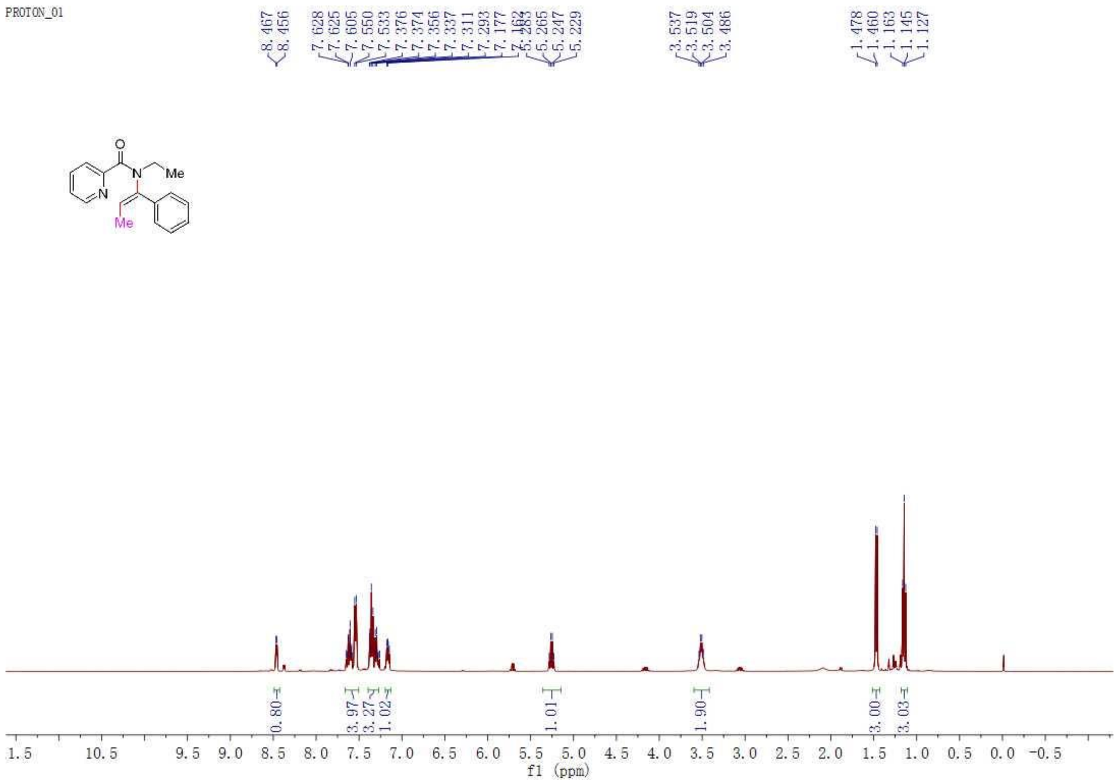

^{1}H NMR spectra for compound **4f** (400 MHz, $CDCl_3$)

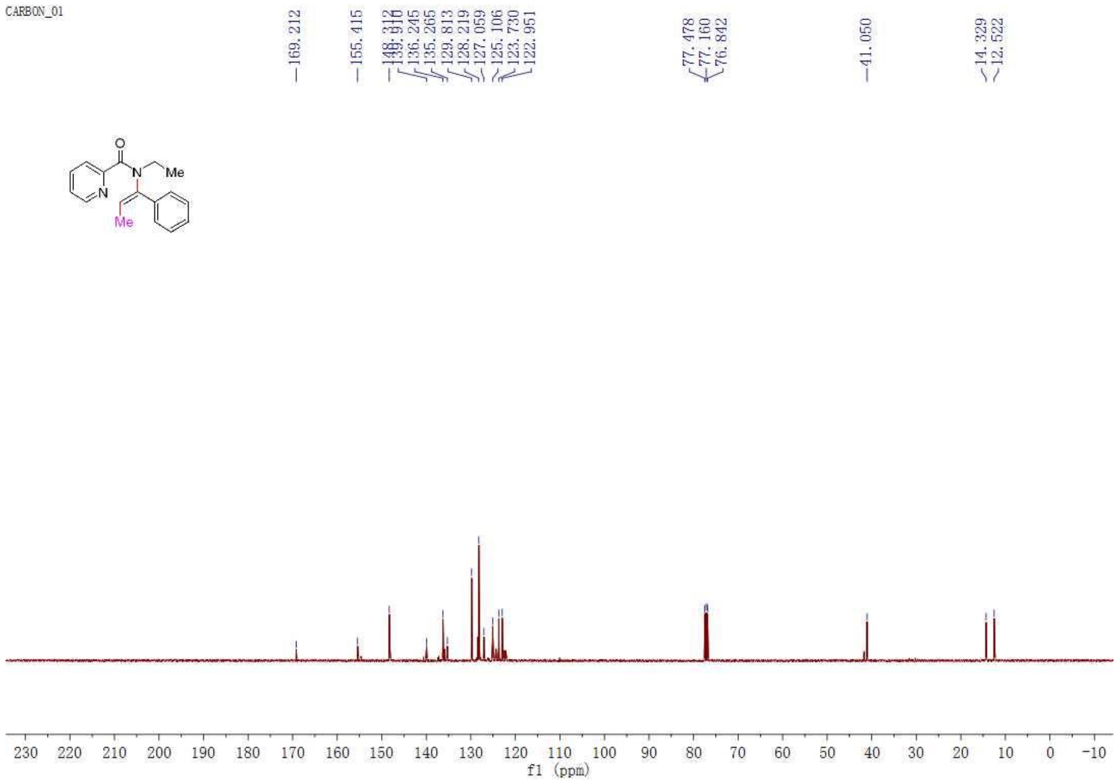

^{13}C NMR spectra for compound **4f** (100 MHz, $CDCl_3$)

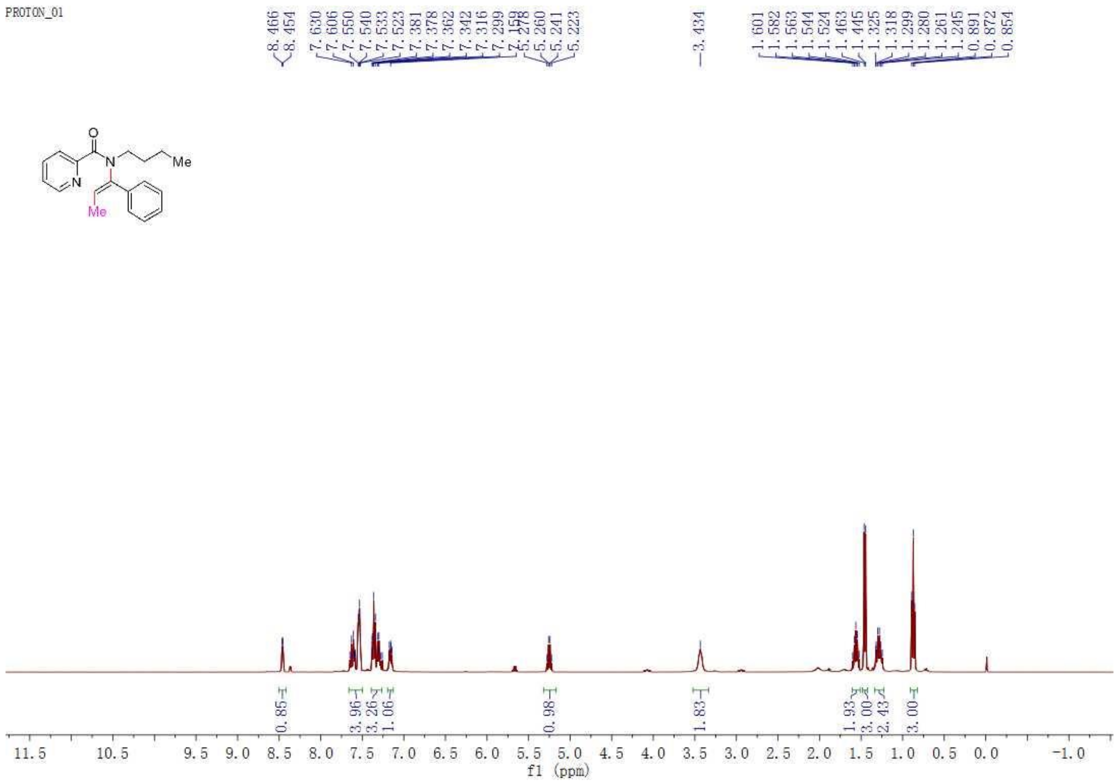

^{1}H NMR spectra for compound **4g** (400 MHz, $CDCl_3$)

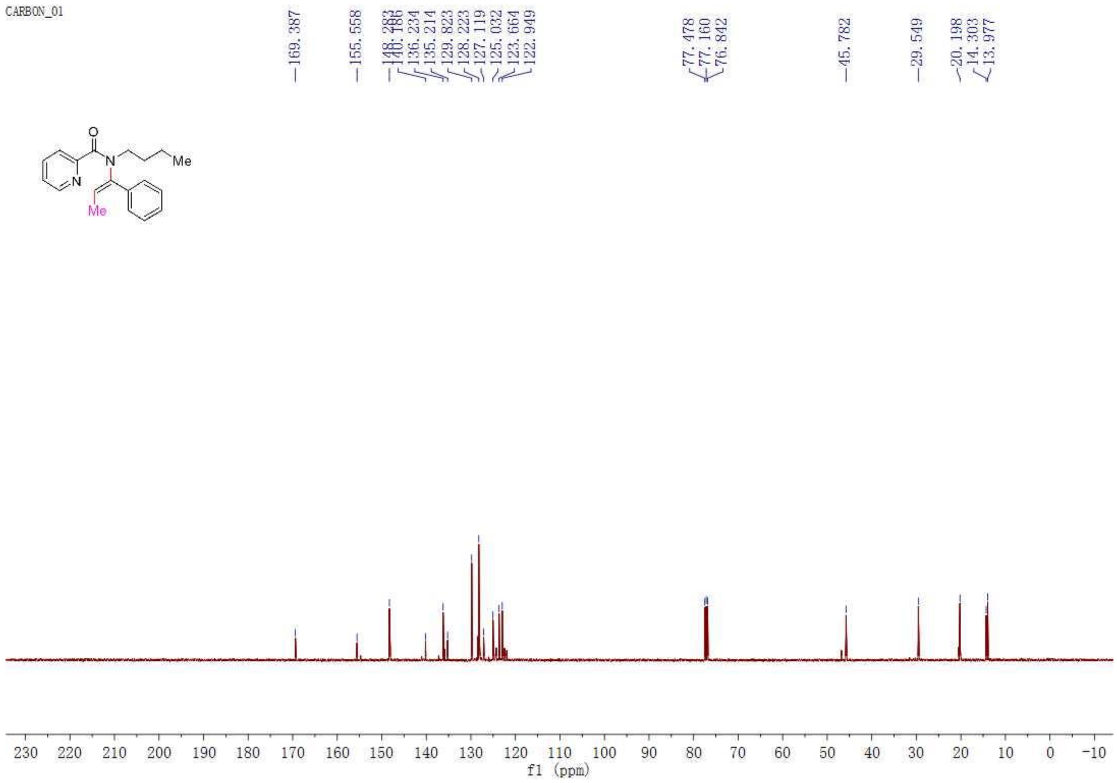

^{13}C NMR spectra for compound **4g** (100 MHz, $CDCl_3$)

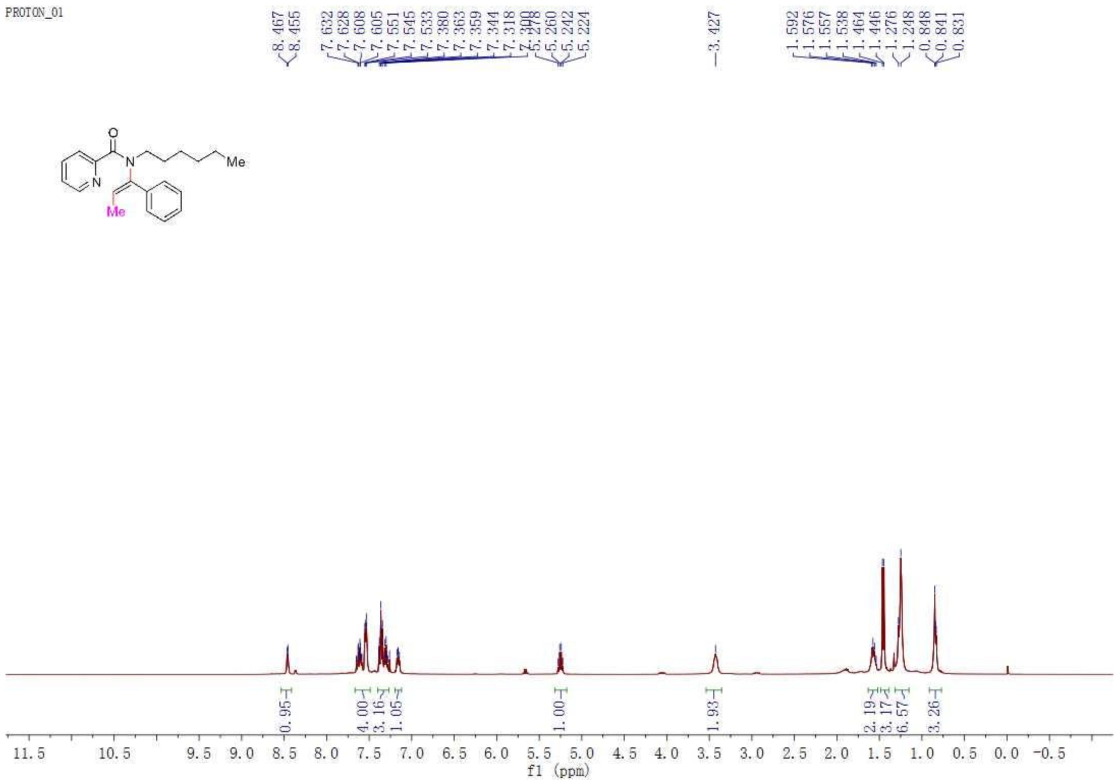

^{1}H NMR spectra for compound **4h** (400 MHz, $CDCl_3$)

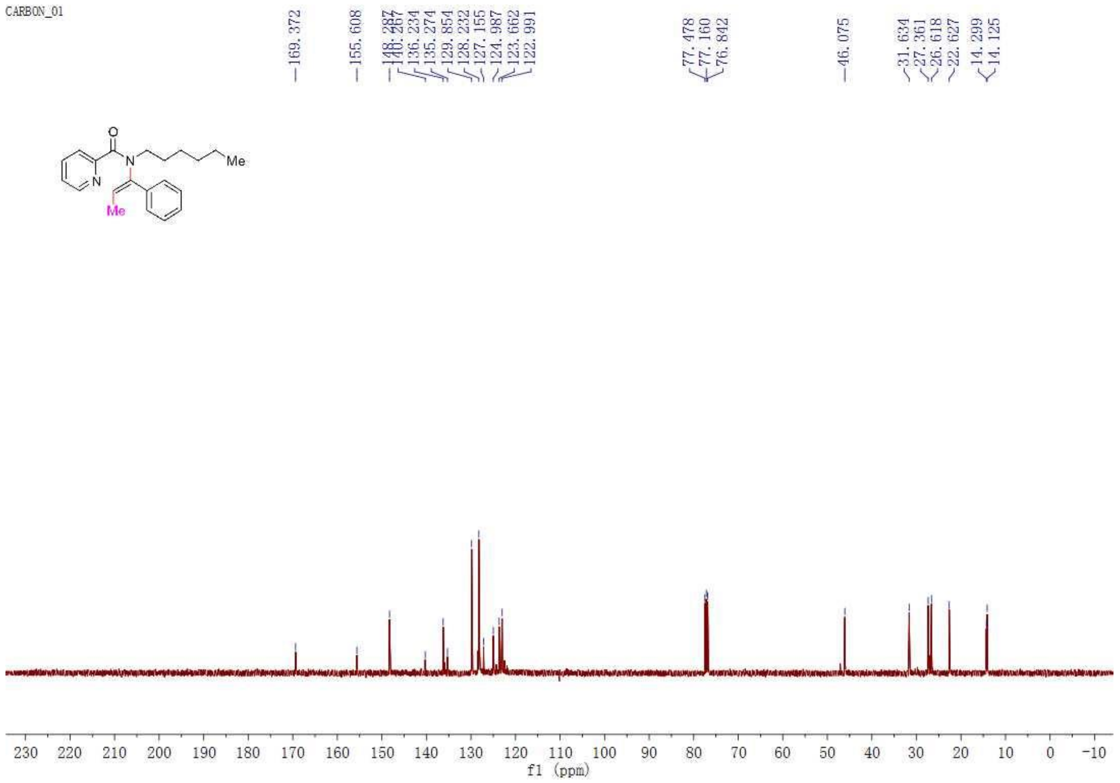

^{13}C NMR spectra for compound **4h** (100 MHz, $CDCl_3$)

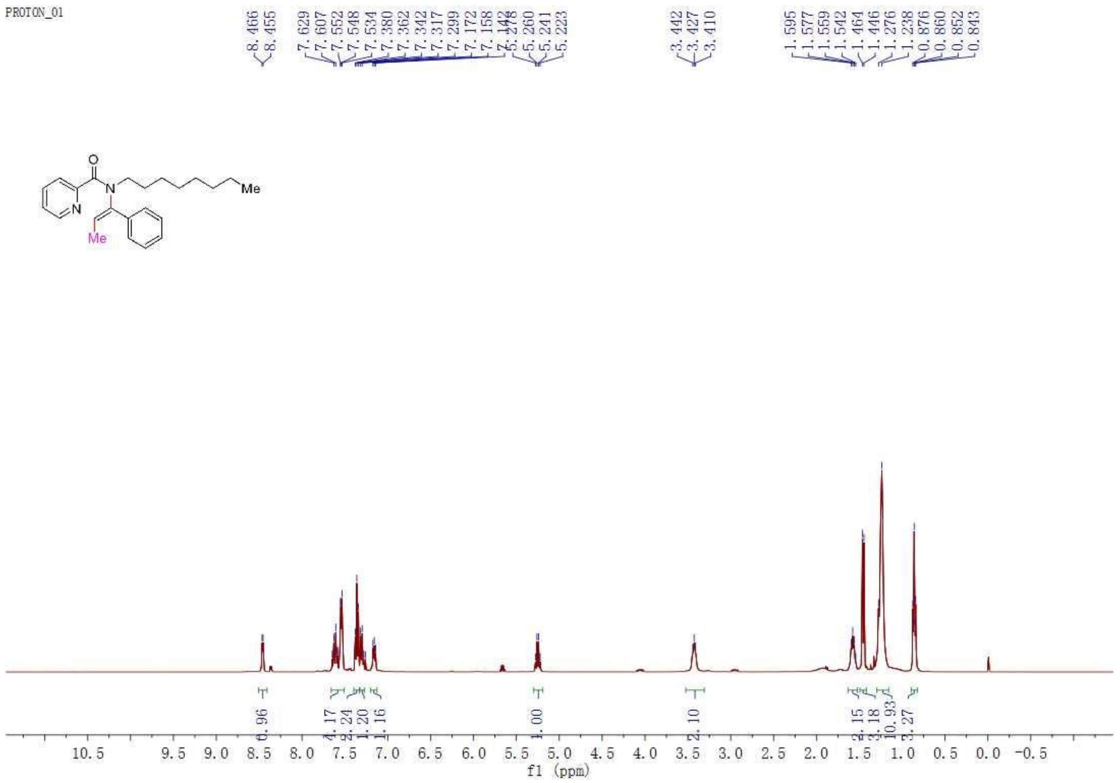

^{1}H NMR spectra for compound **4i** (400 MHz, $CDCl_3$)

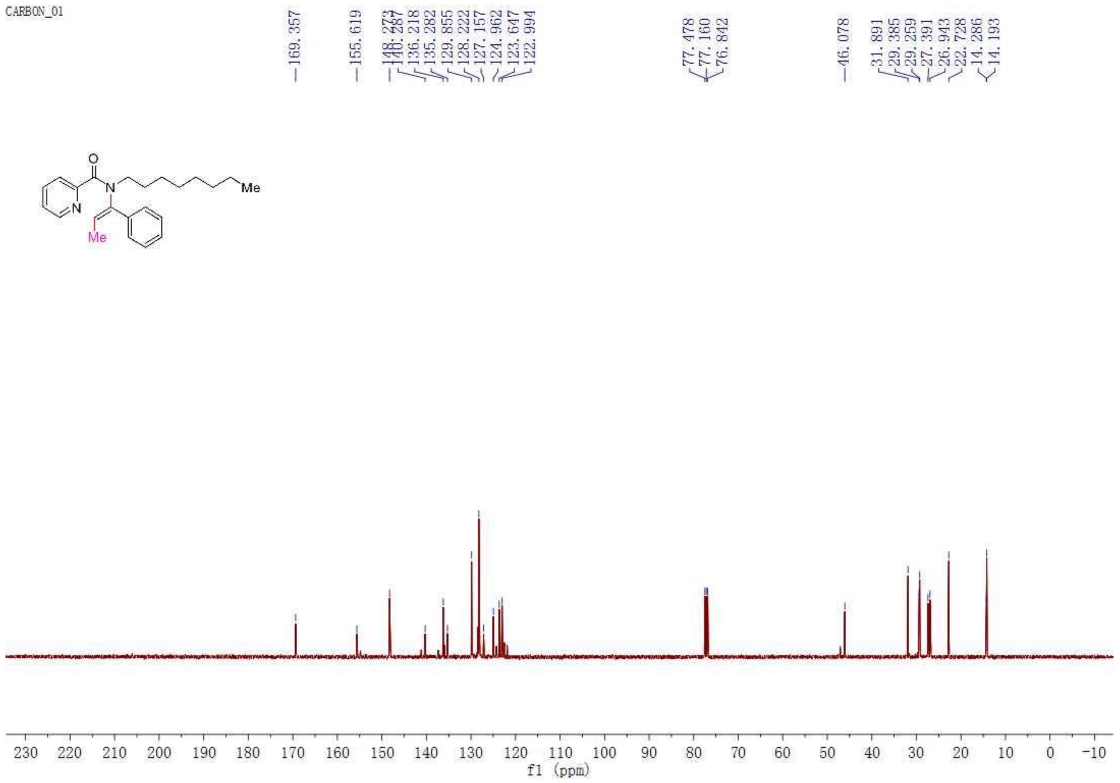

^{13}C NMR spectra for compound **4i** (100 MHz, $CDCl_3$)

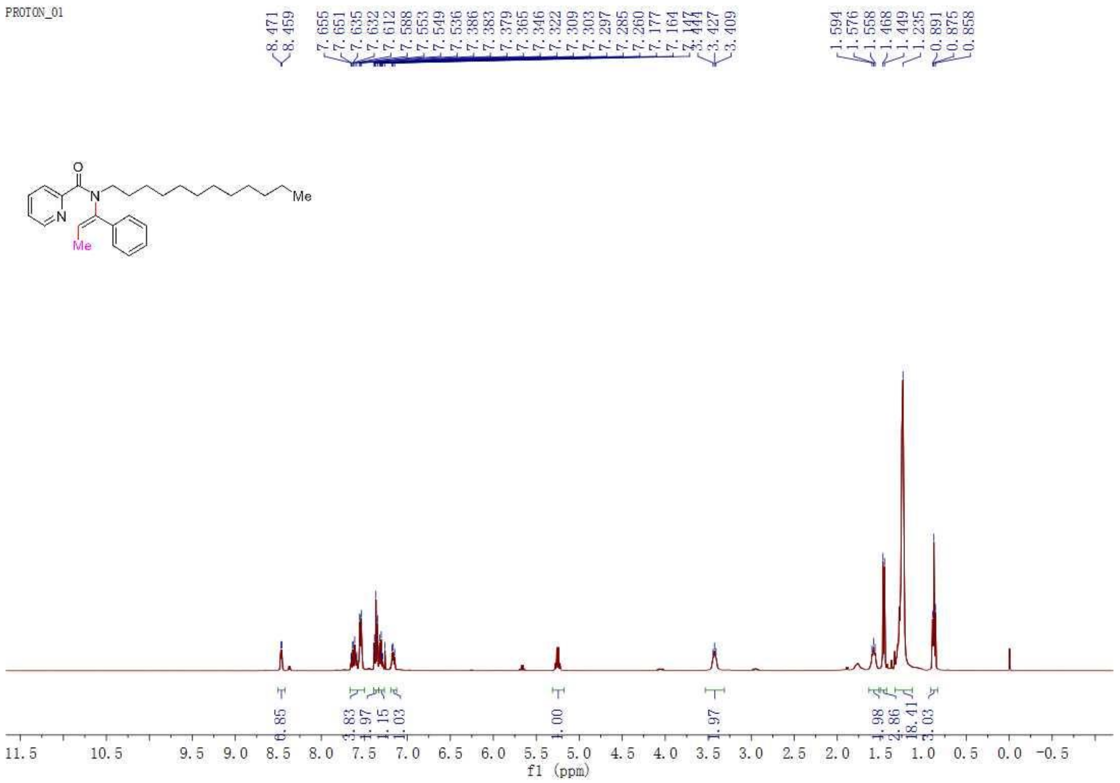

^{1}H NMR spectra for compound **4j** (400 MHz, $CDCl_3$)

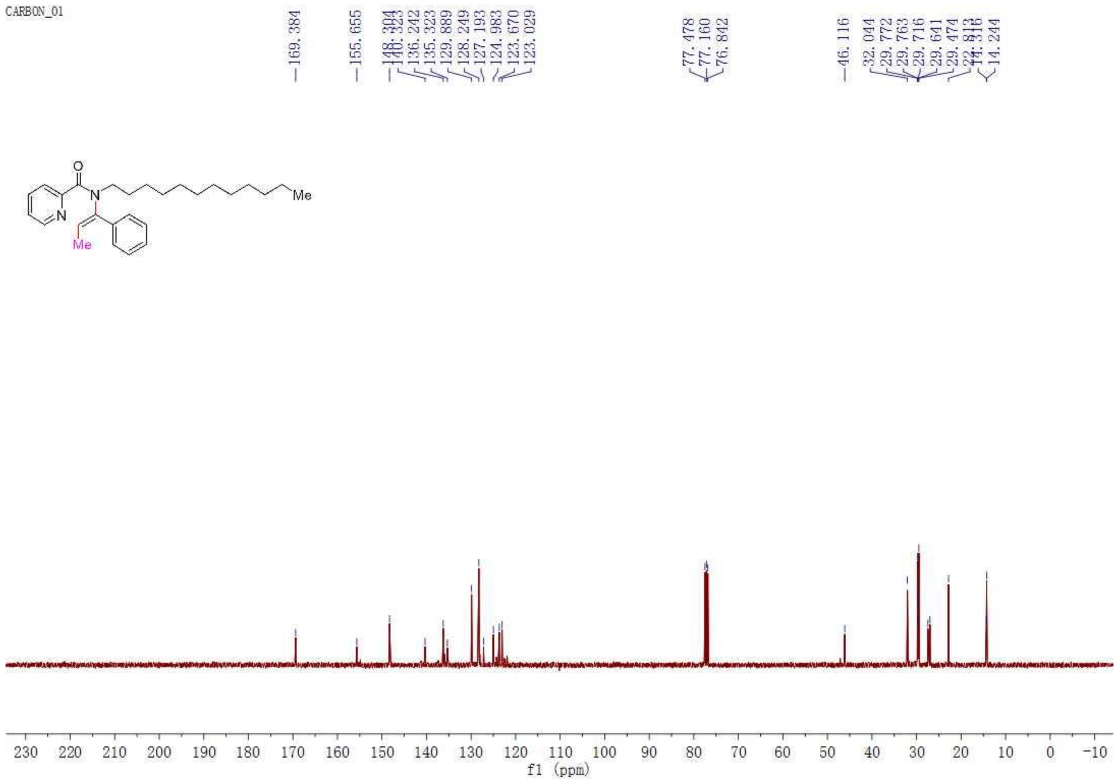

^{13}C NMR spectra for compound **4j** (100 MHz, $CDCl_3$)

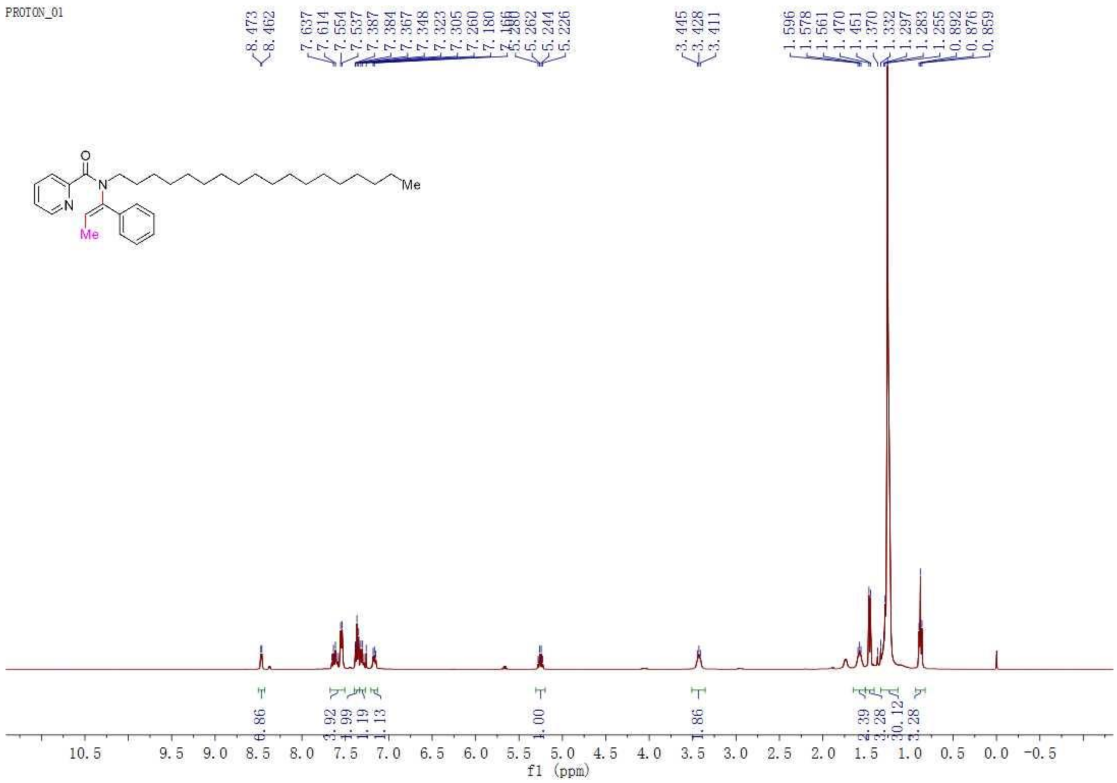

^{1}H NMR spectra for compound **4k** (400 MHz, $CDCl_3$)

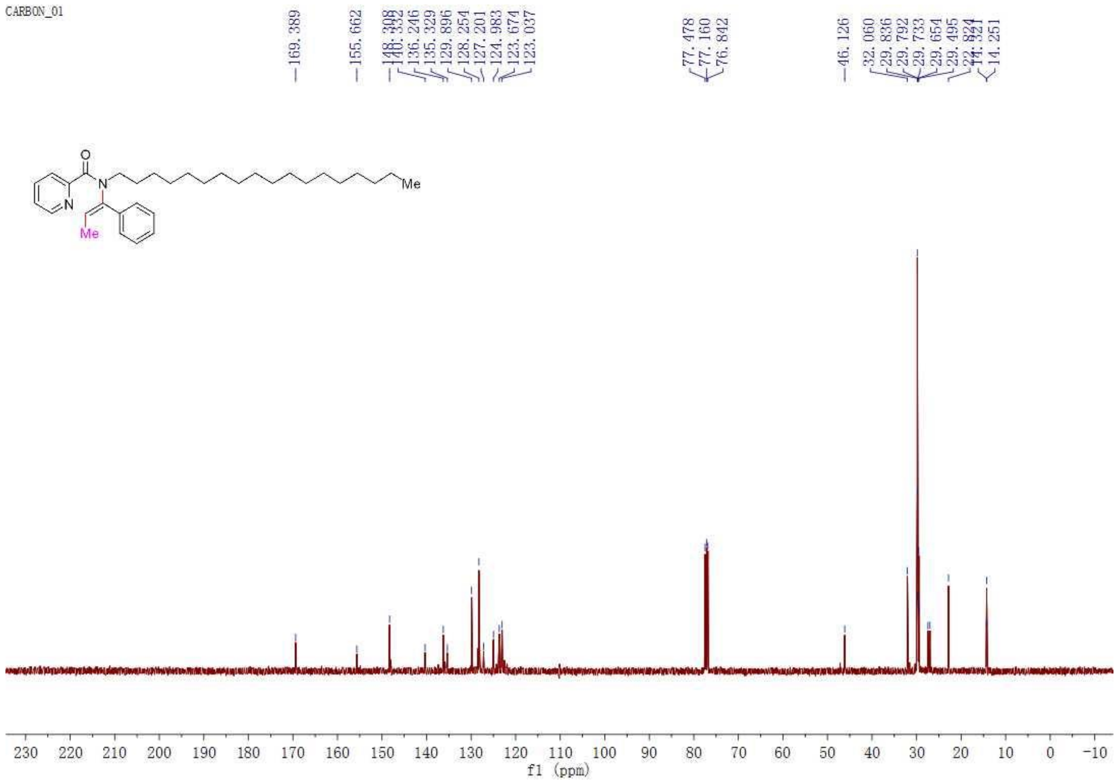

^{13}C NMR spectra for compound **4k** (100 MHz, $CDCl_3$)

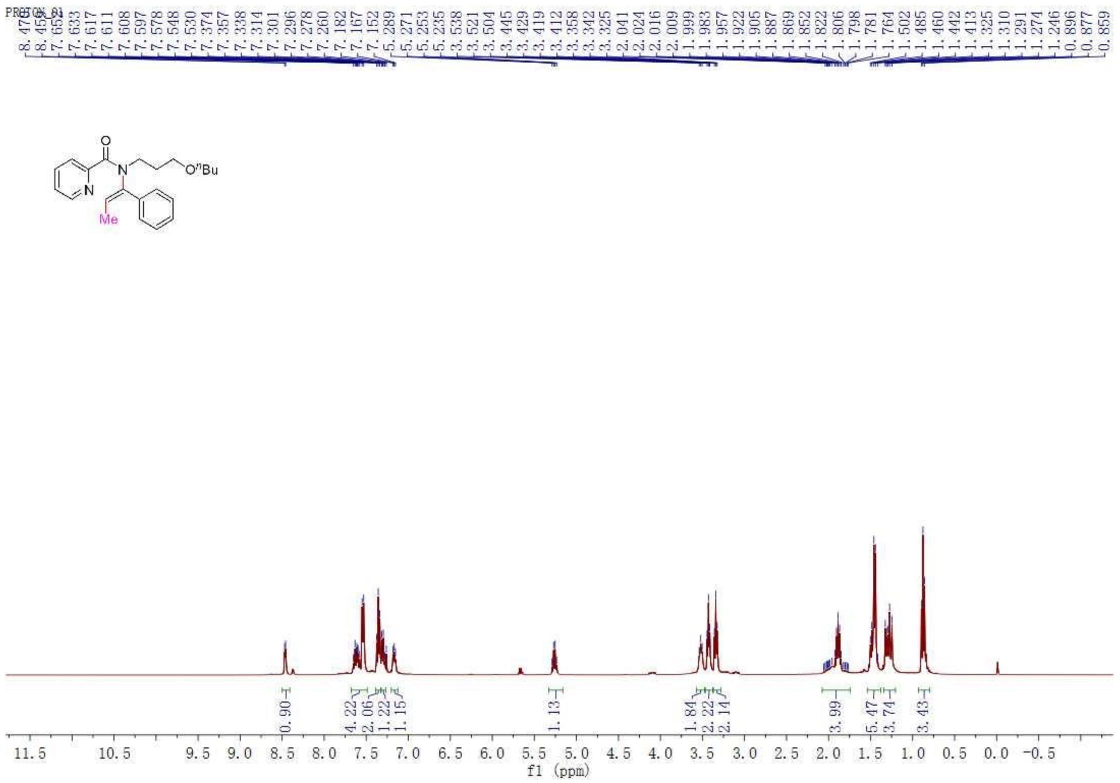

^{1}H NMR spectra for compound **4l** (400 MHz, $CDCl_3$)

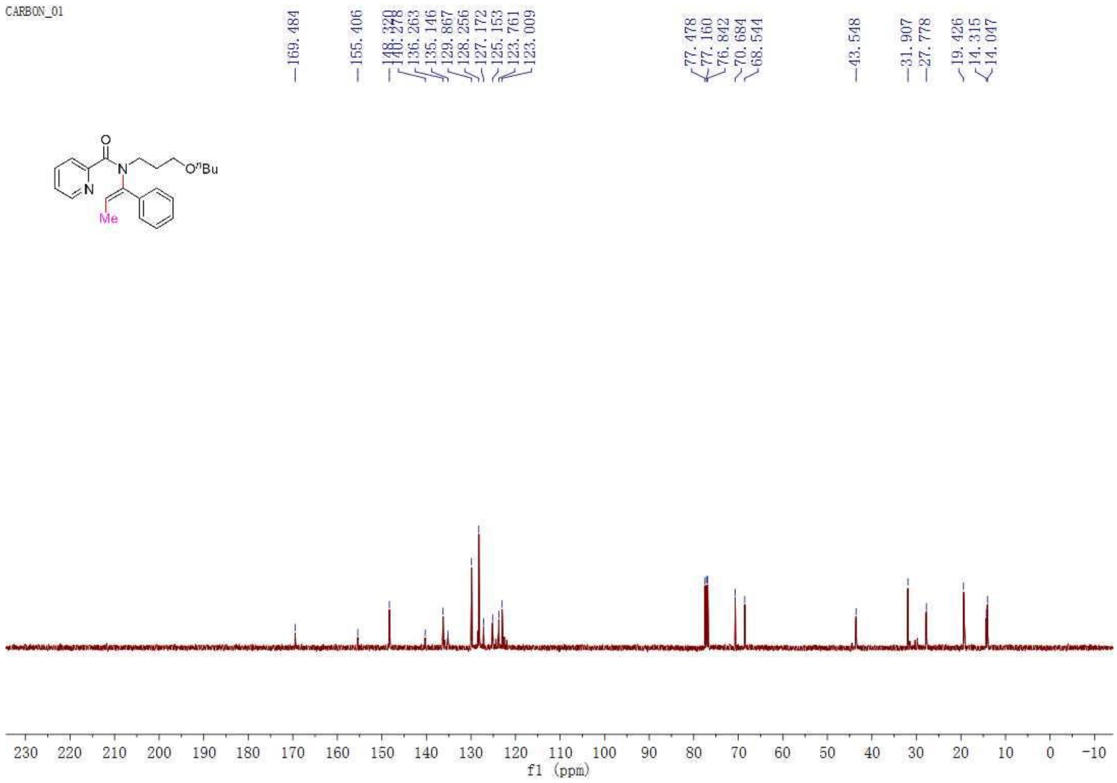

^{13}C NMR spectra for compound **4l** (100 MHz, $CDCl_3$)

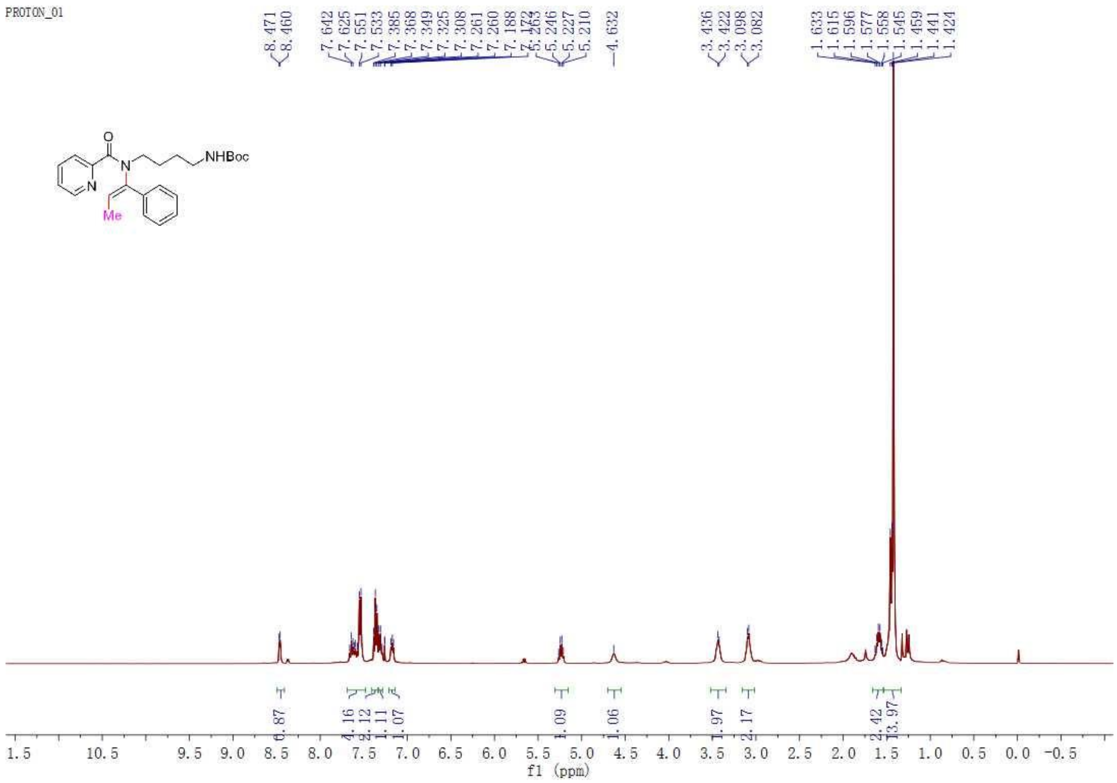

^{1}H NMR spectra for compound **4m** (400 MHz, $CDCl_3$)

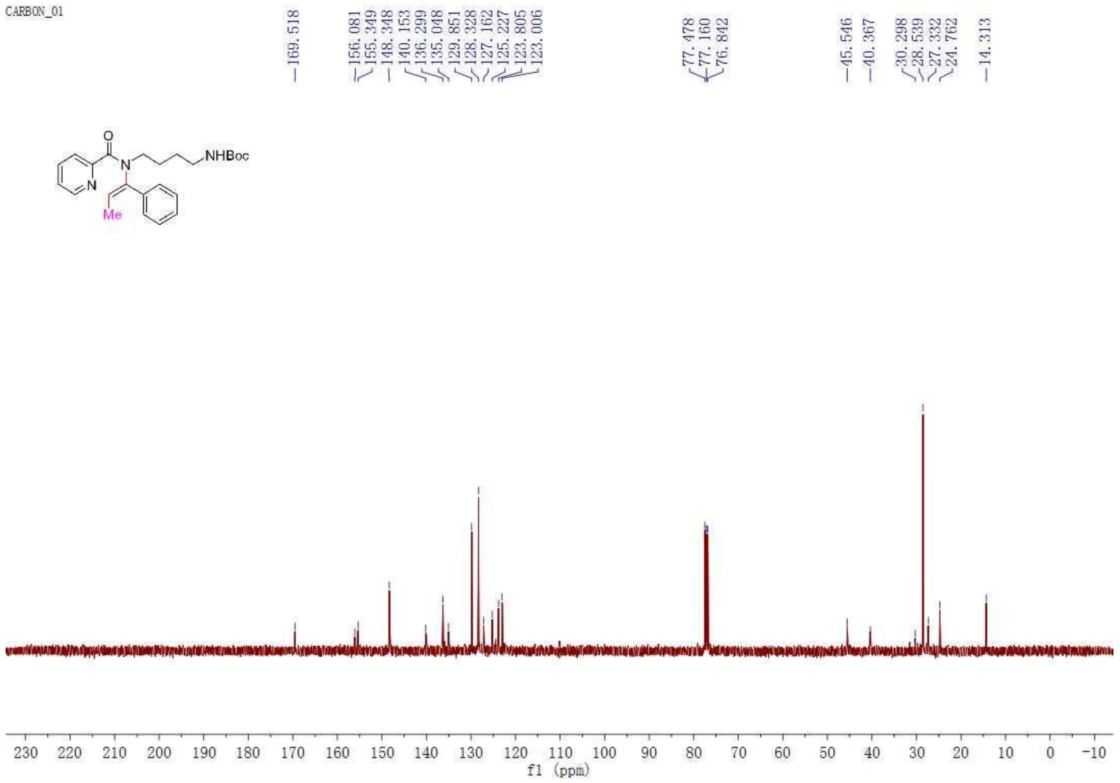

^{13}C NMR spectra for compound **4m** (100 MHz, $CDCl_3$)

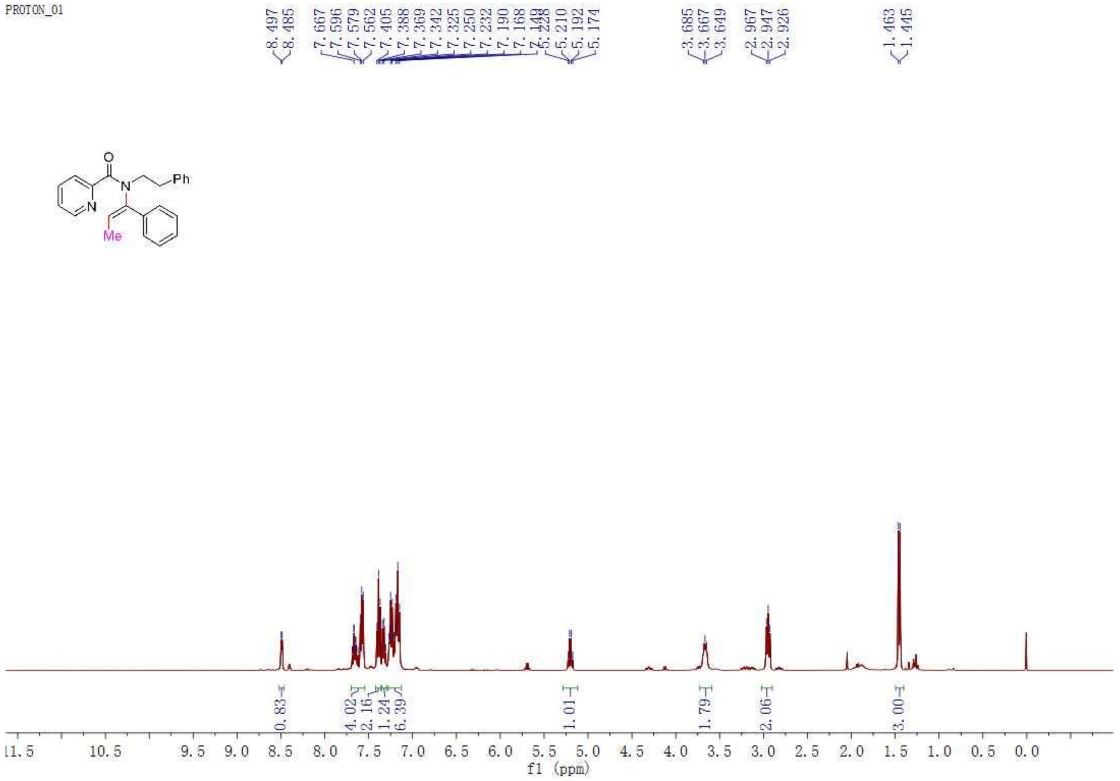

^{1}H NMR spectra for compound **4n** (400 MHz, $CDCl_3$)

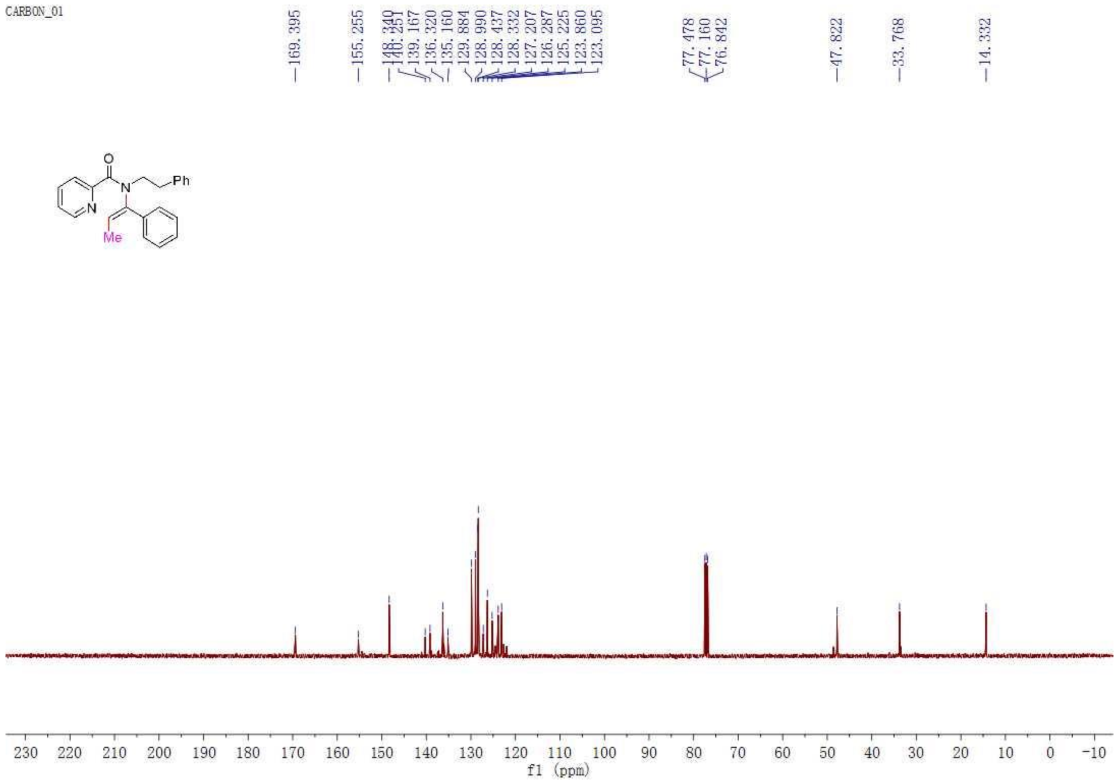

^{13}C NMR spectra for compound **4n** (100 MHz, $CDCl_3$)

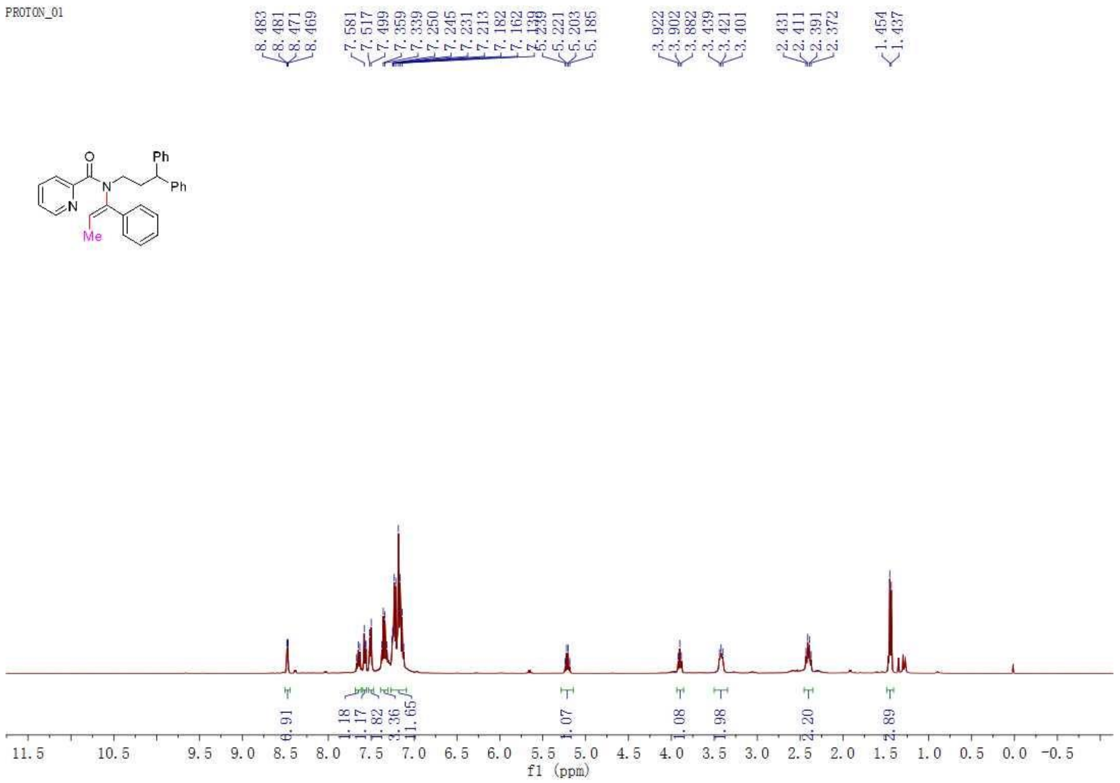

^{1}H NMR spectra for compound **4o** (400 MHz, $CDCl_3$)

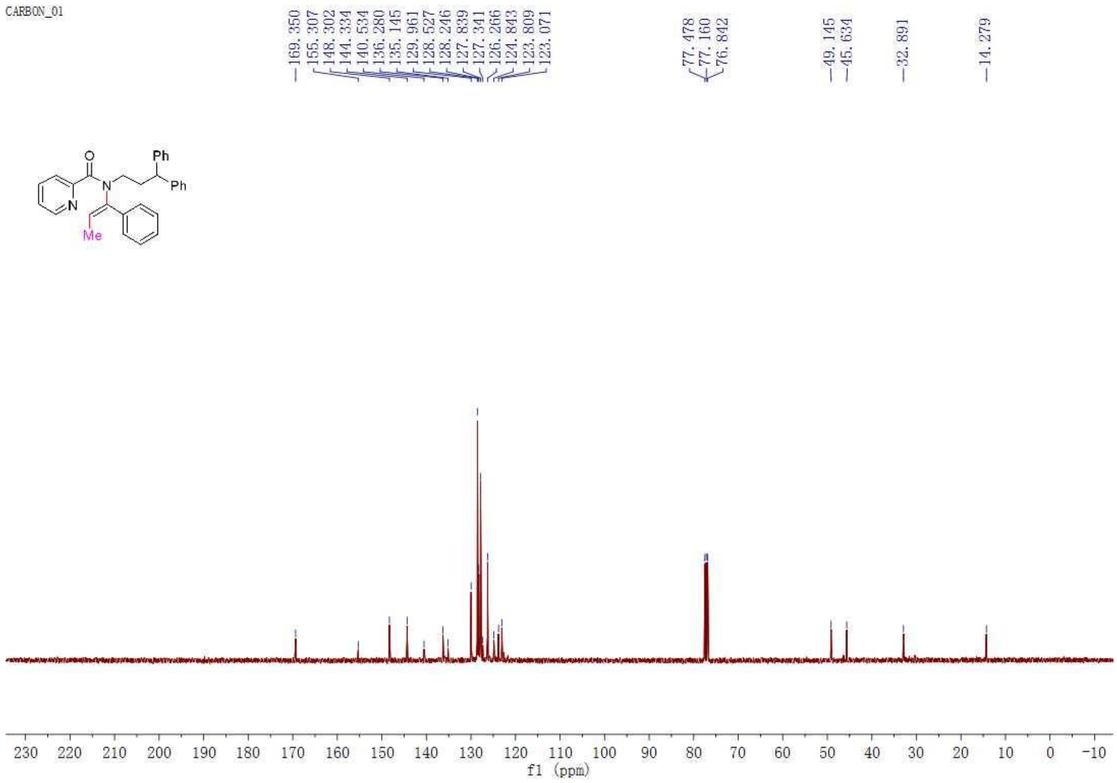

^{13}C NMR spectra for compound **4o** (100 MHz, $CDCl_3$)

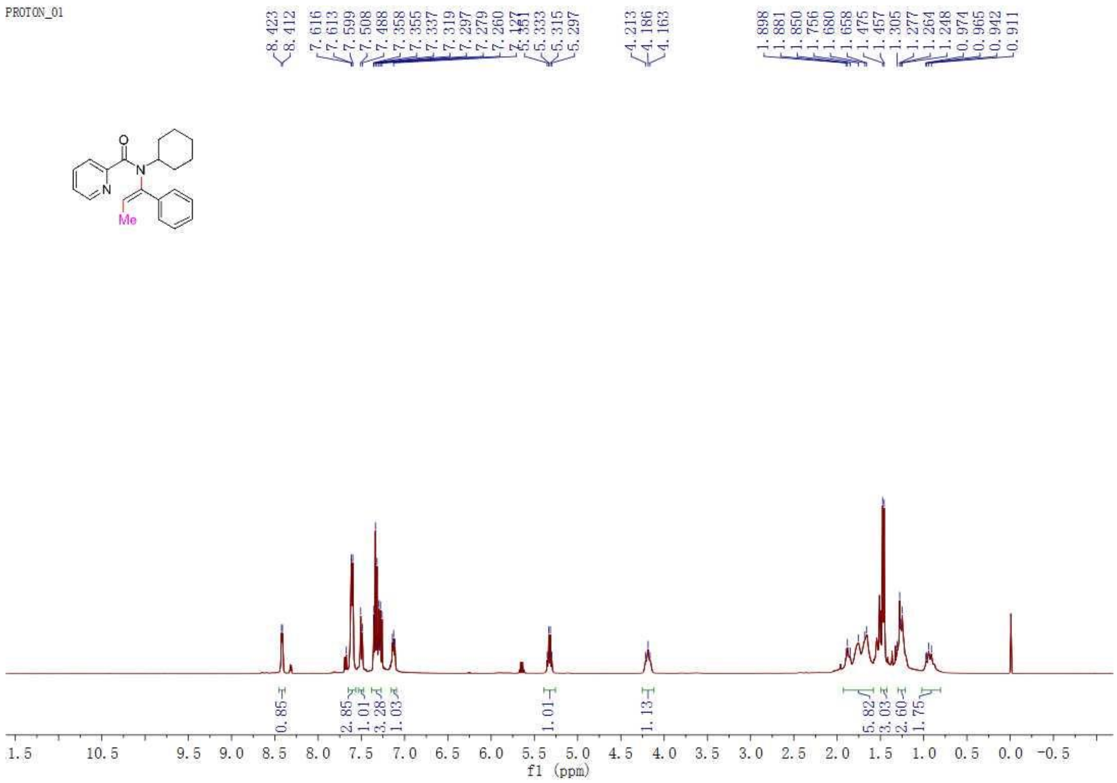

^{1}H NMR spectra for compound **4p** (400 MHz, $CDCl_3$)

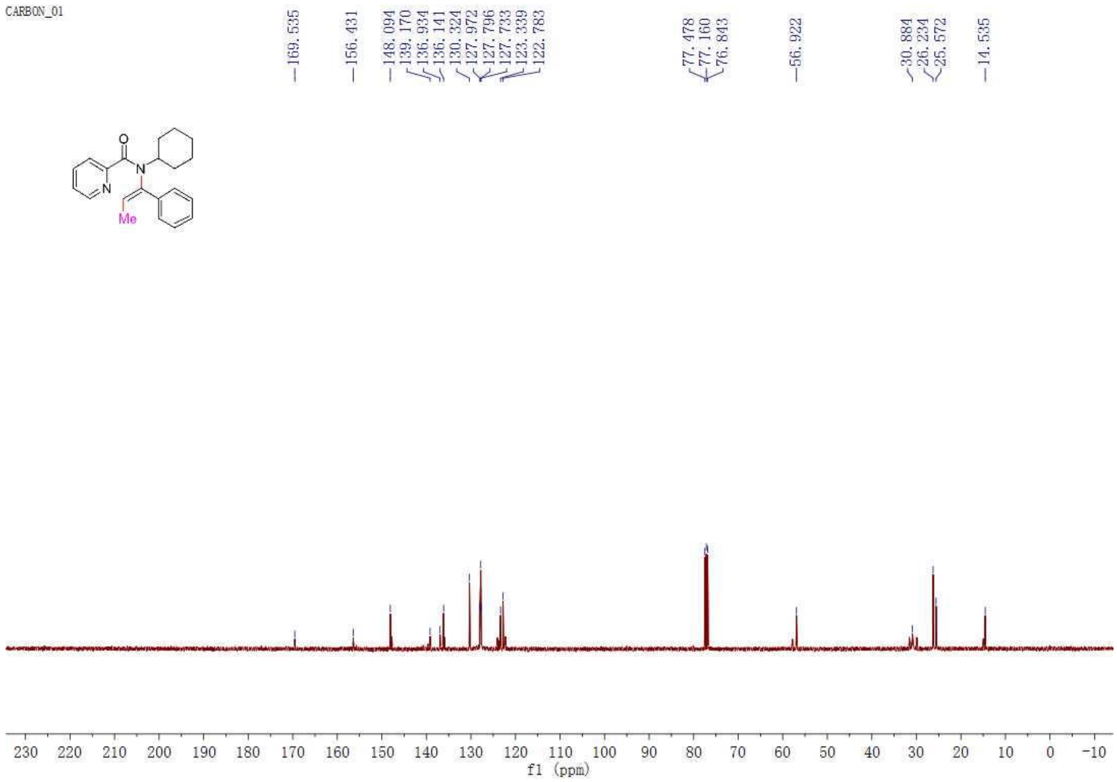

^{13}C NMR spectra for compound **4p** (100 MHz, $CDCl_3$)

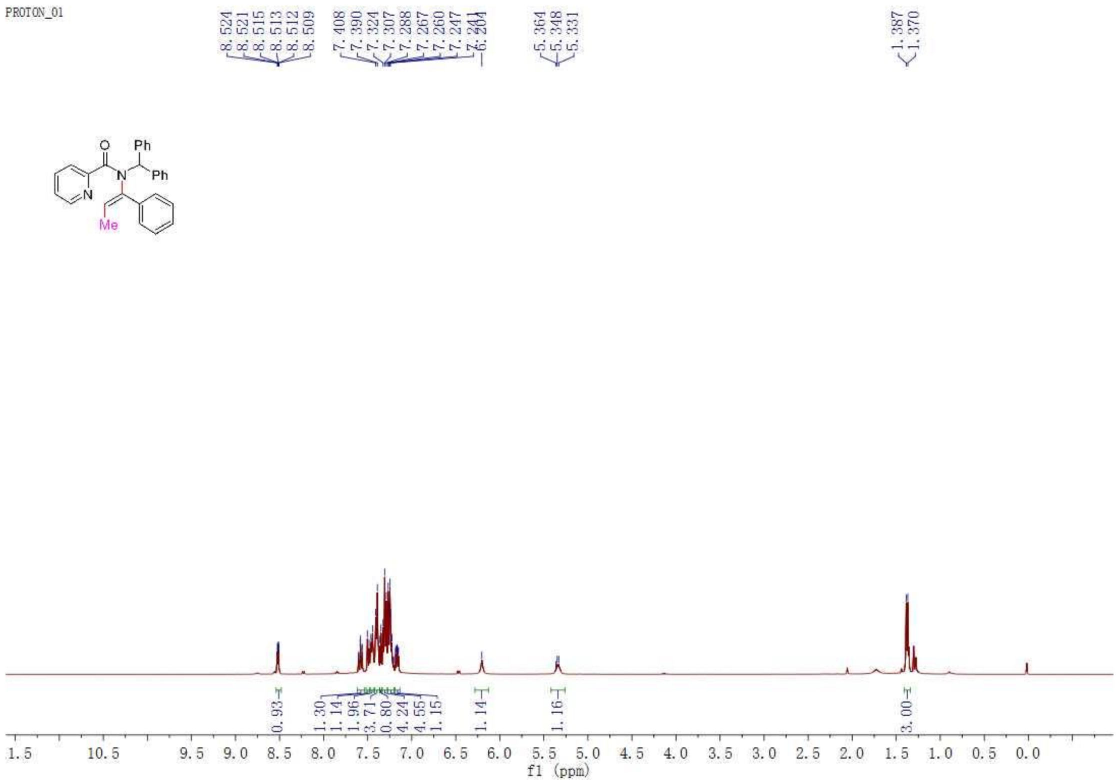

^{1}H NMR spectra for compound **4q** (400 MHz, $CDCl_3$)

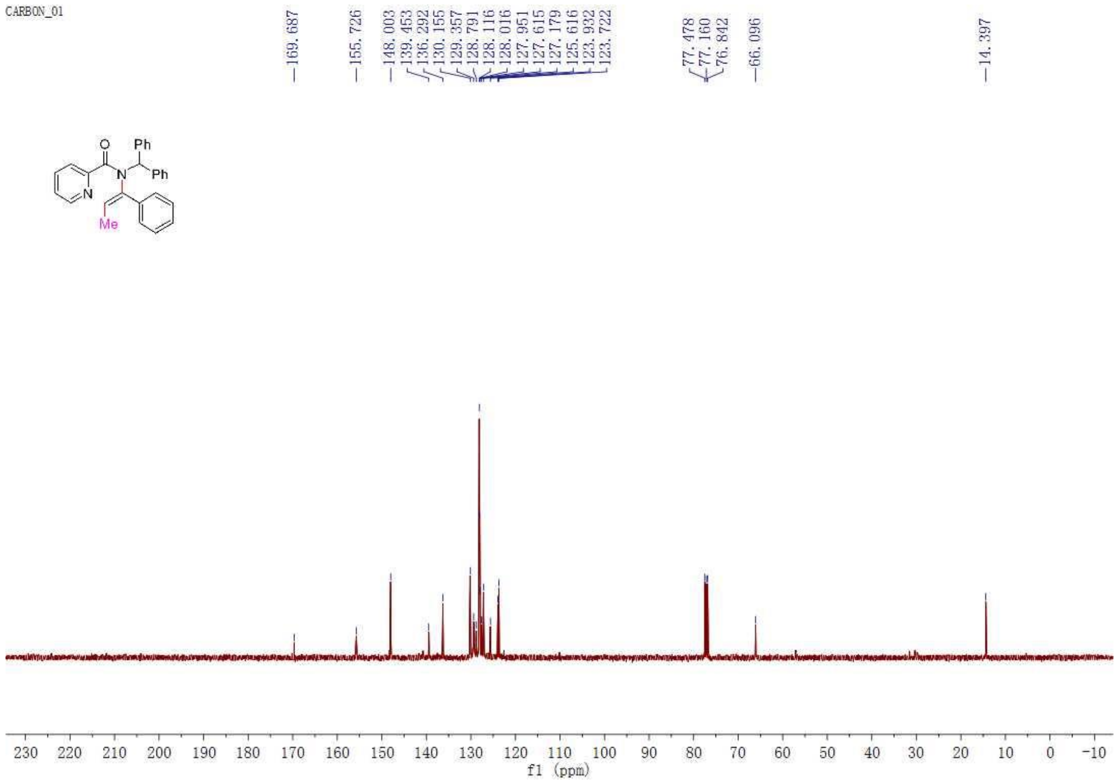

^{13}C NMR spectra for compound **4q** (100 MHz, $CDCl_3$)

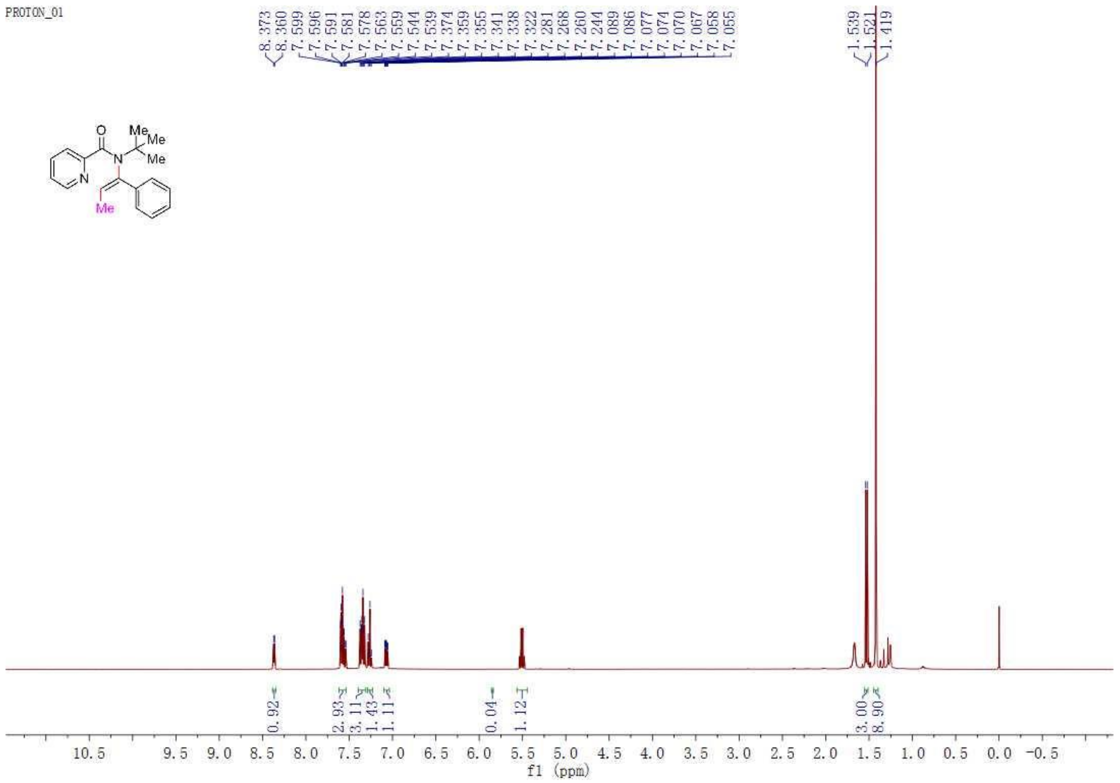

^{1}H NMR spectra for compound **4r** (400 MHz, $CDCl_3$)

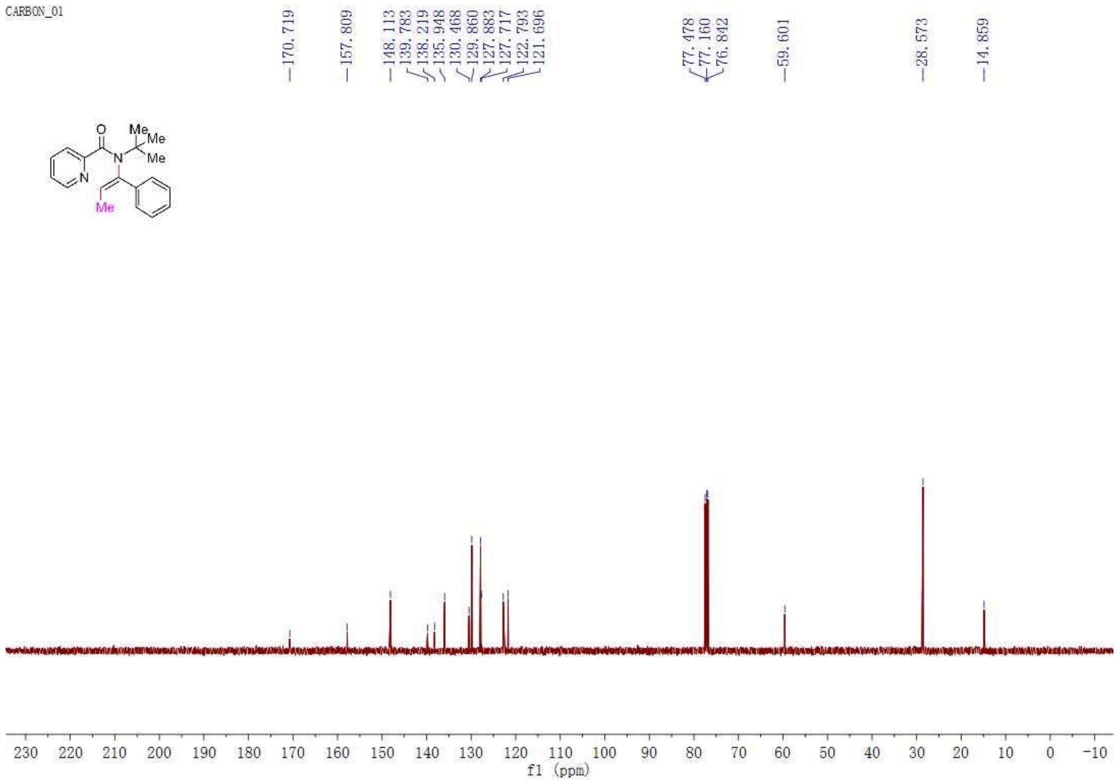

^{13}C NMR spectra for compound **4r** (100 MHz, $CDCl_3$)

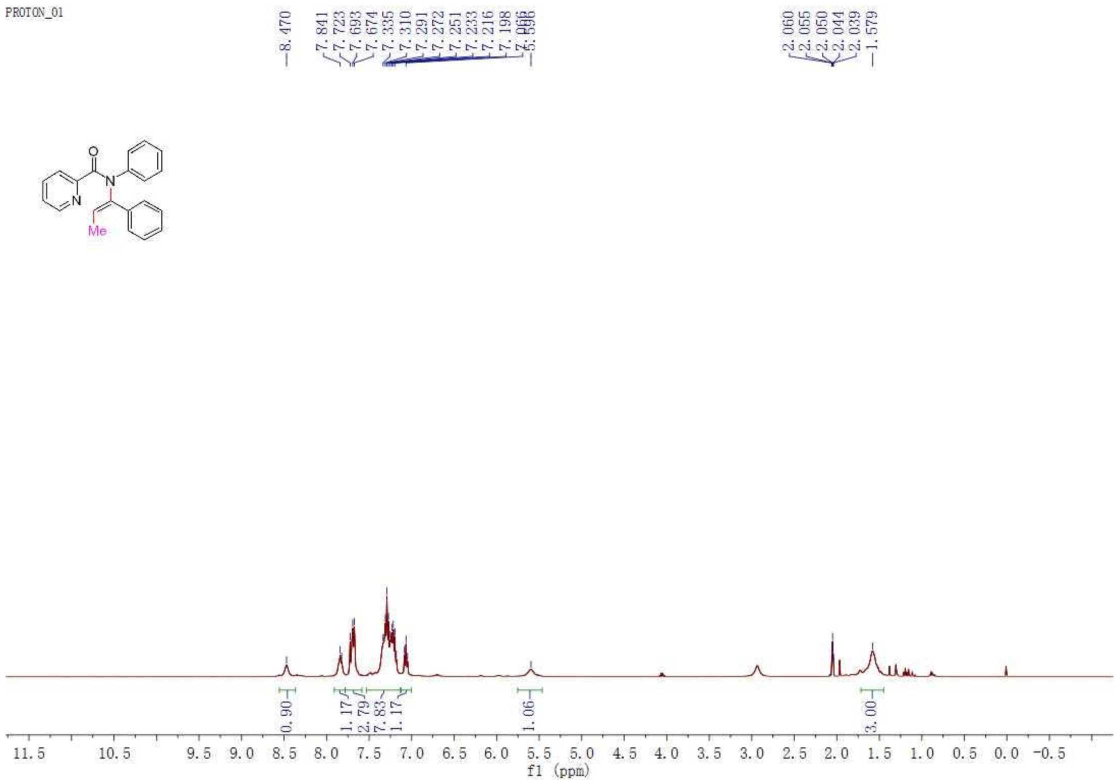

^{1}H NMR spectra for compound **4s** (400 MHz, Acetone-d_6)

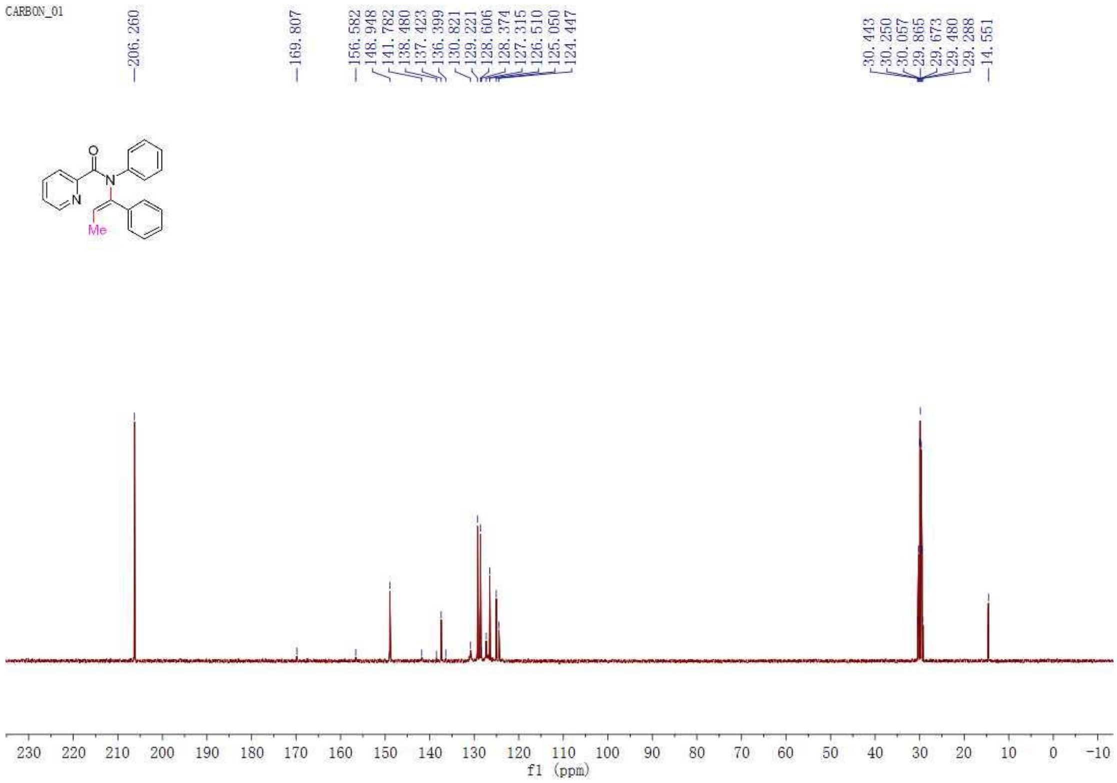

^{13}C NMR spectra for compound **4s** (100 MHz, Acetone-d_6)

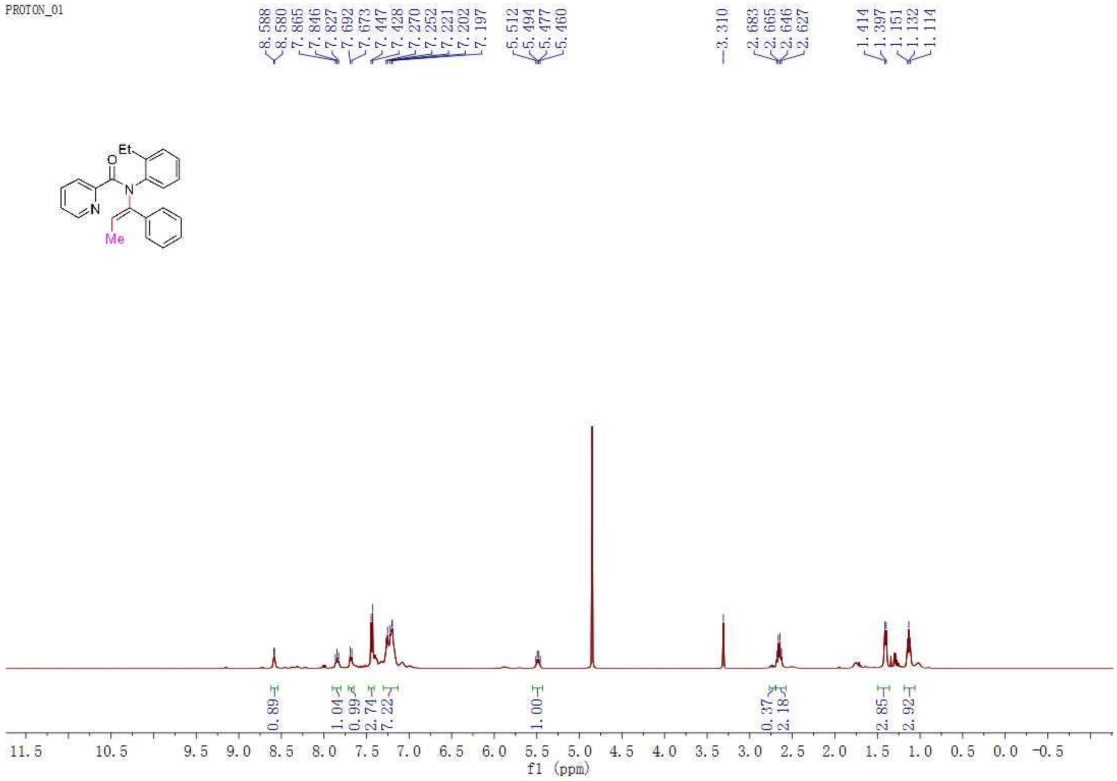

^{1}H NMR spectra for compound **4t** (400 MHz, CD_3OD)

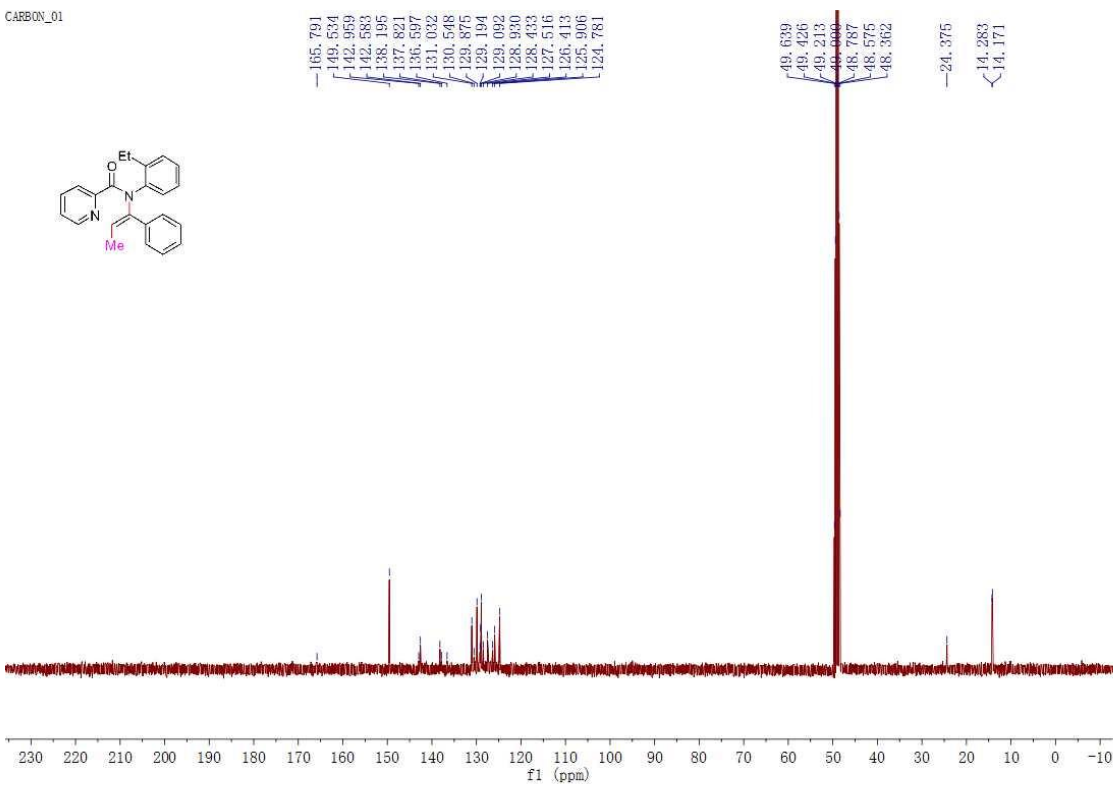

^{13}C NMR spectra for compound **4t** (100 MHz, CD_3OD)

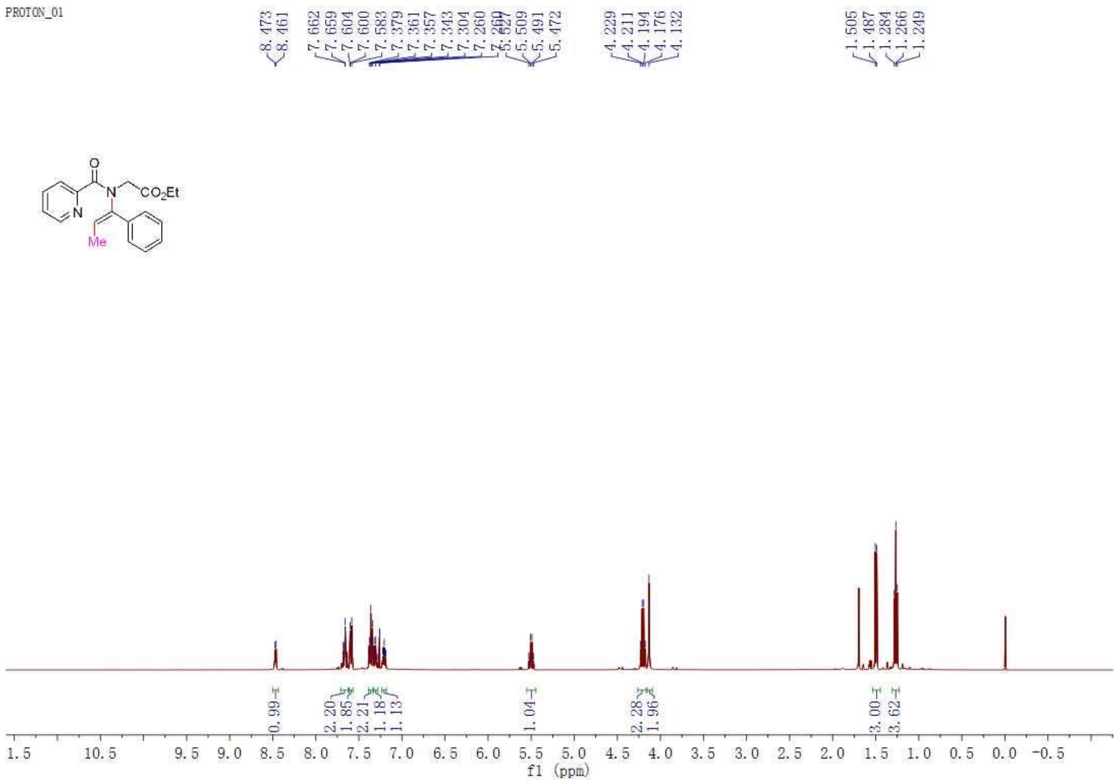

^{1}H NMR spectra for compound **5a** (400 MHz, $CDCl_3$)

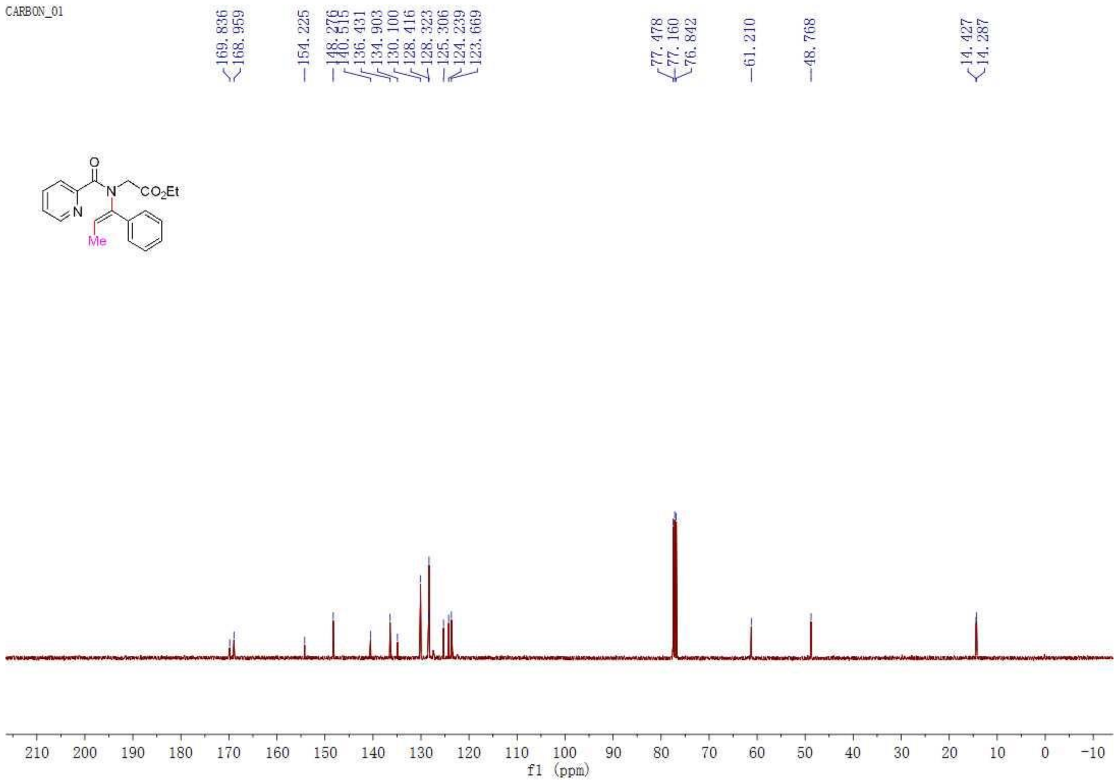

^{13}C NMR spectra for compound **5a** (100 MHz, $CDCl_3$)

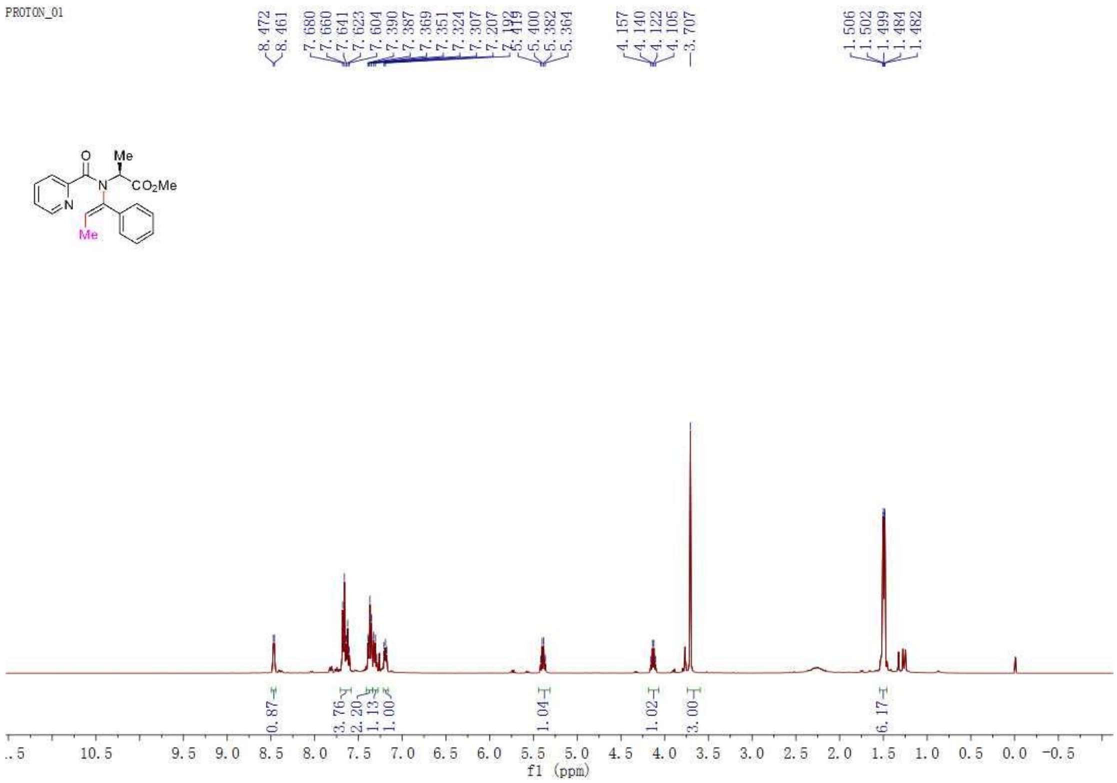

^{1}H NMR spectra for compound **5b** (400 MHz, $CDCl_3$)

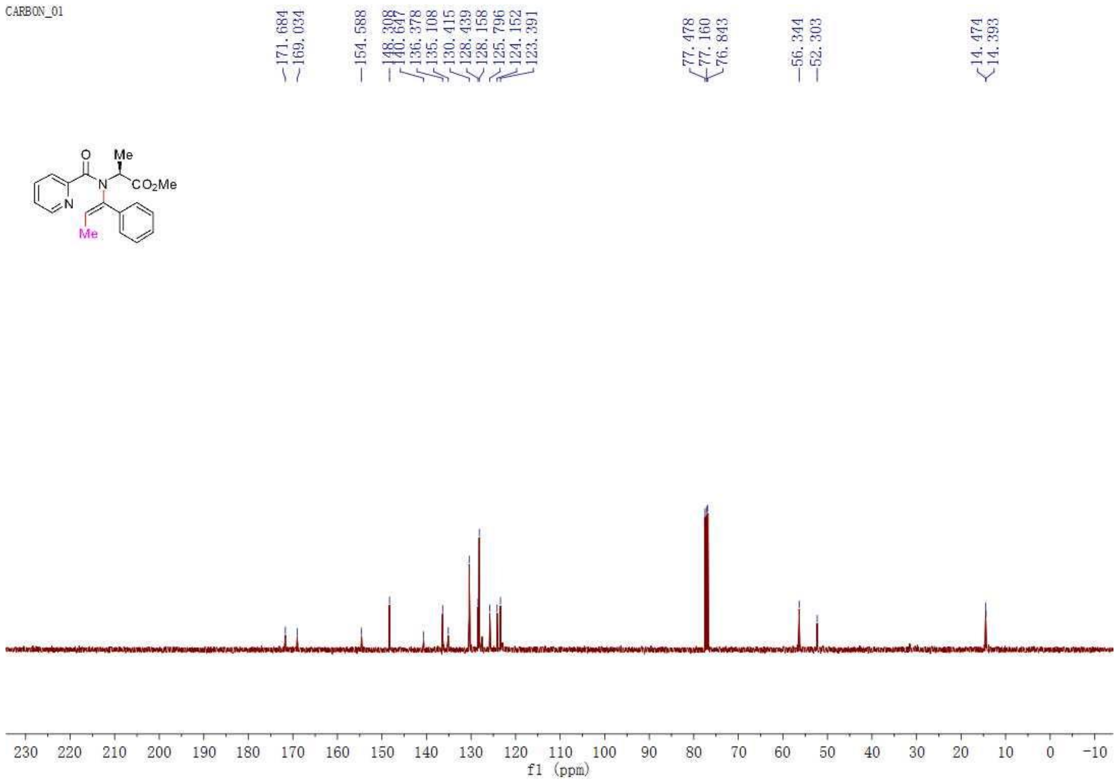

^{13}C NMR spectra for compound **5b** (100 MHz, $CDCl_3$)

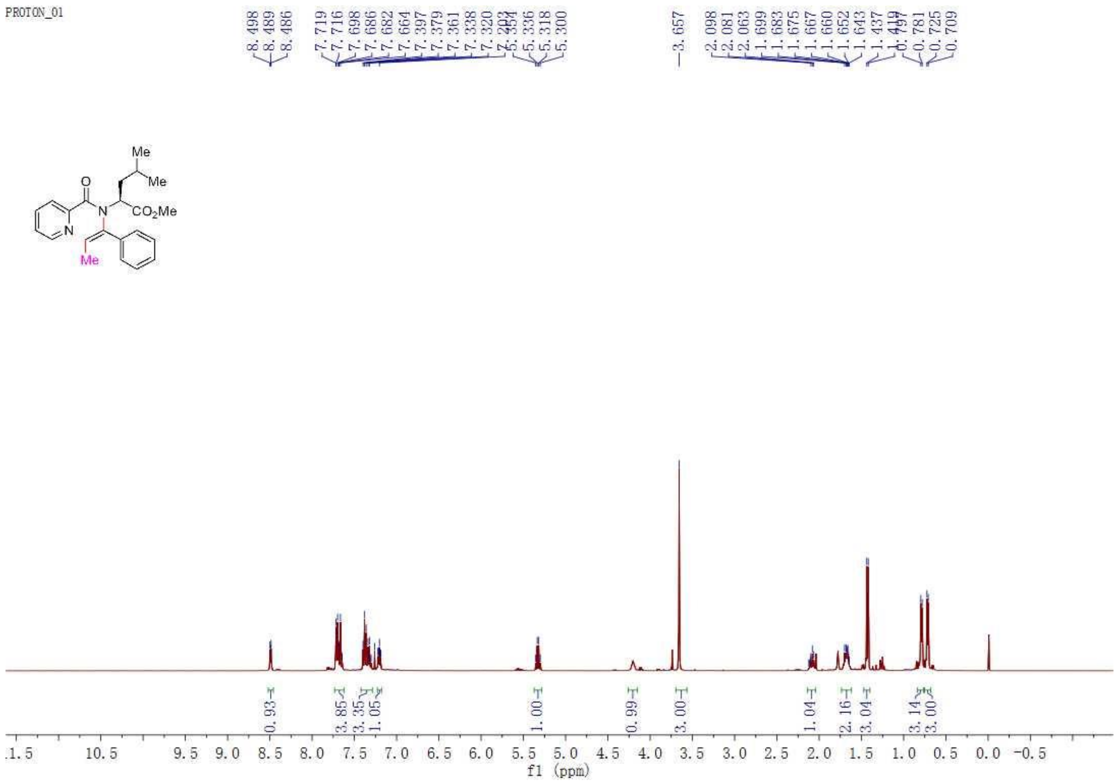

^{1}H NMR spectra for compound **5c** (400 MHz, $CDCl_3$)

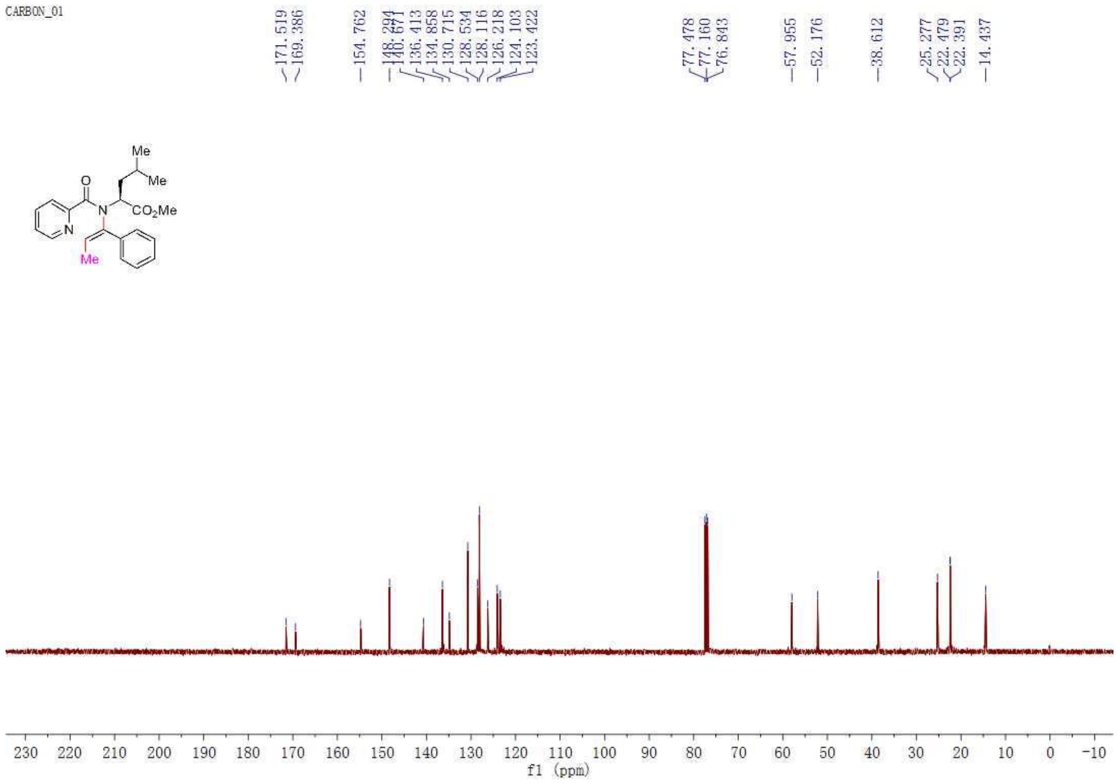

^{13}C NMR spectra for compound **5c** (100 MHz, $CDCl_3$)

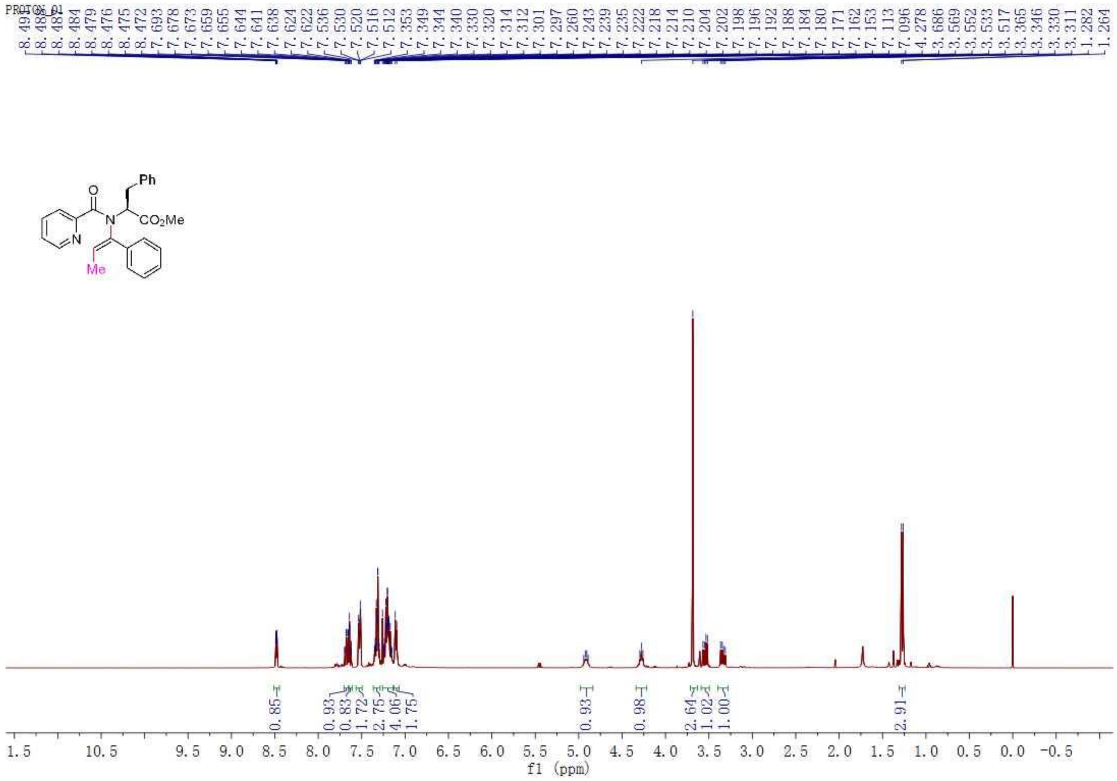

^{1}H NMR spectra for compound **5d** (400 MHz, $CDCl_3$)

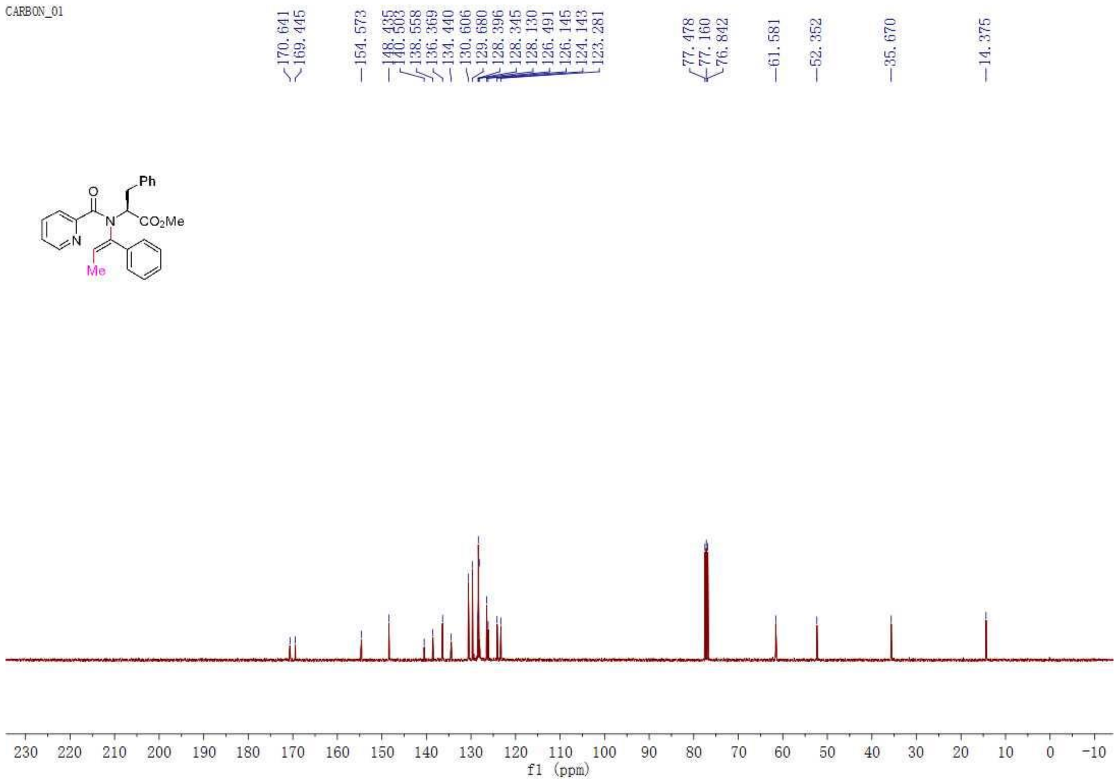

^{13}C NMR spectra for compound **5d** (100 MHz, $CDCl_3$)

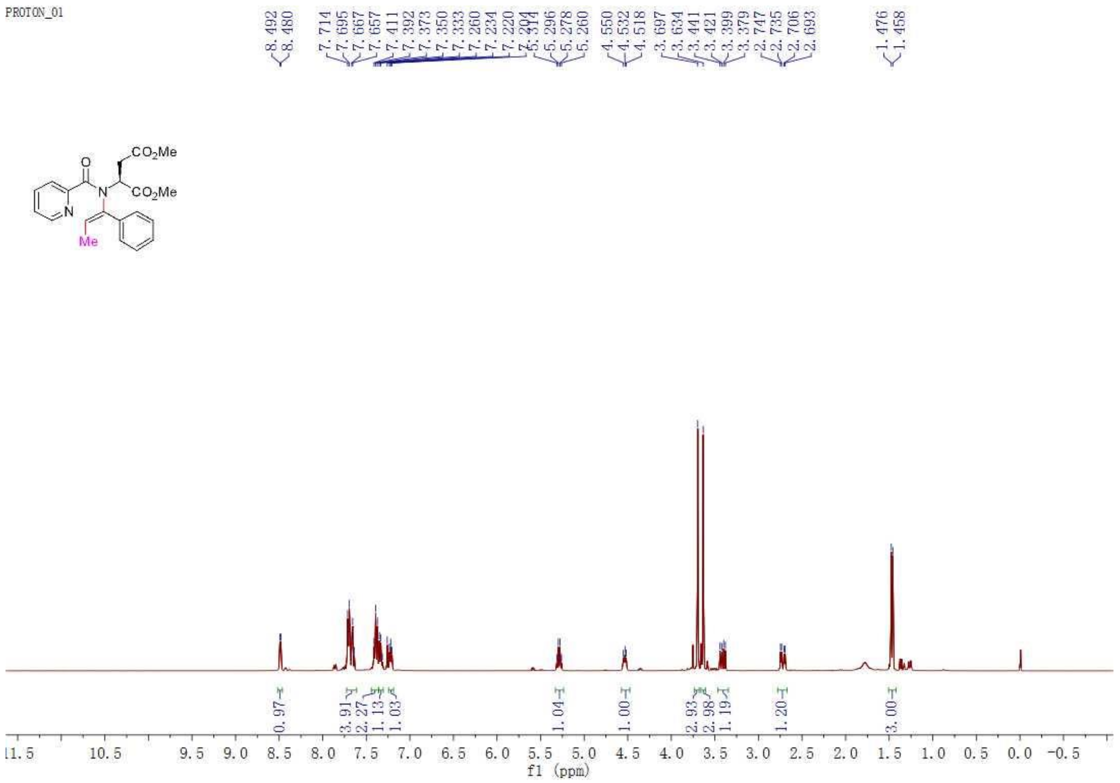

^{1}H NMR spectra for compound **5e** (400 MHz, $CDCl_3$)

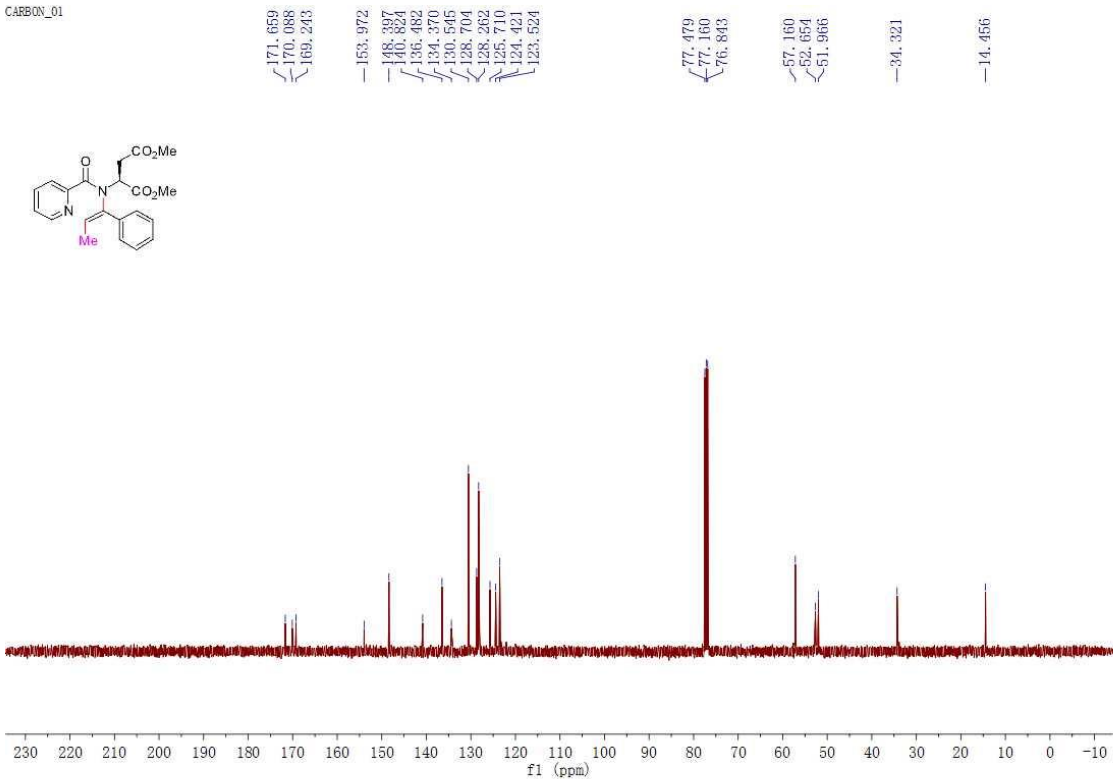

^{13}C NMR spectra for compound **5e** (100 MHz, $CDCl_3$)

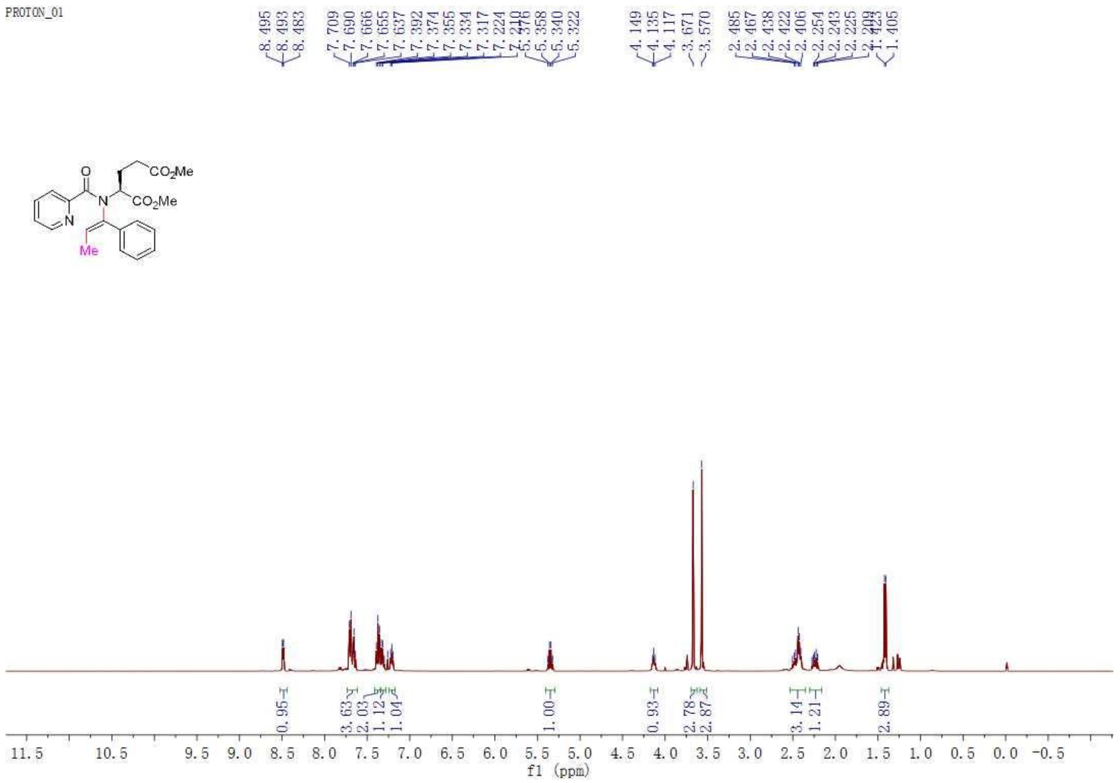

^{1}H NMR spectra for compound **5f** (400 MHz, $CDCl_3$)

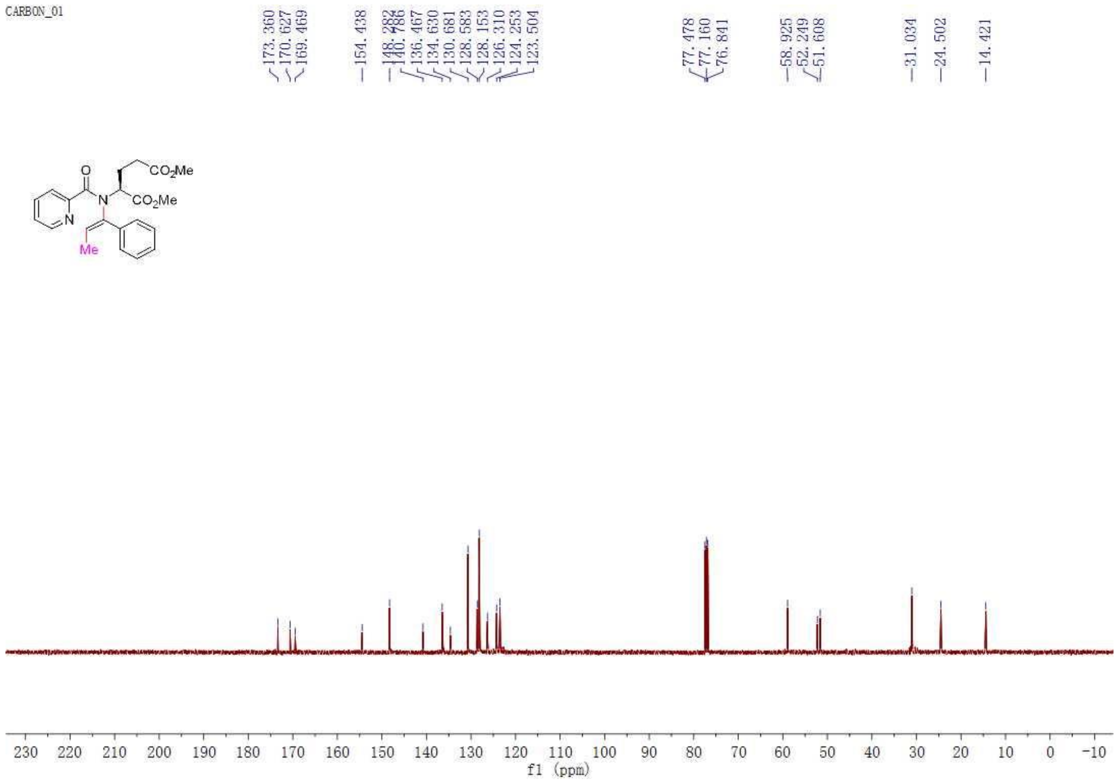

^{13}C NMR spectra for compound **5f** (100 MHz, $CDCl_3$)

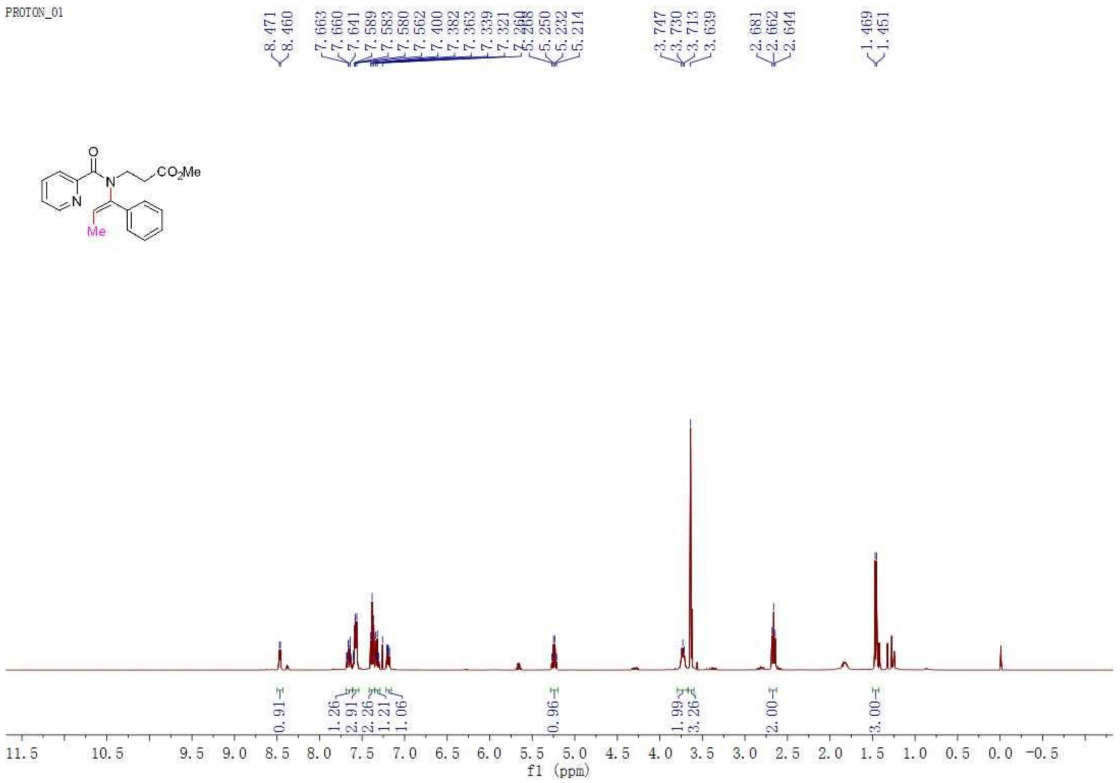

^{1}H NMR spectra for compound **5g** (400 MHz, $CDCl_3$)

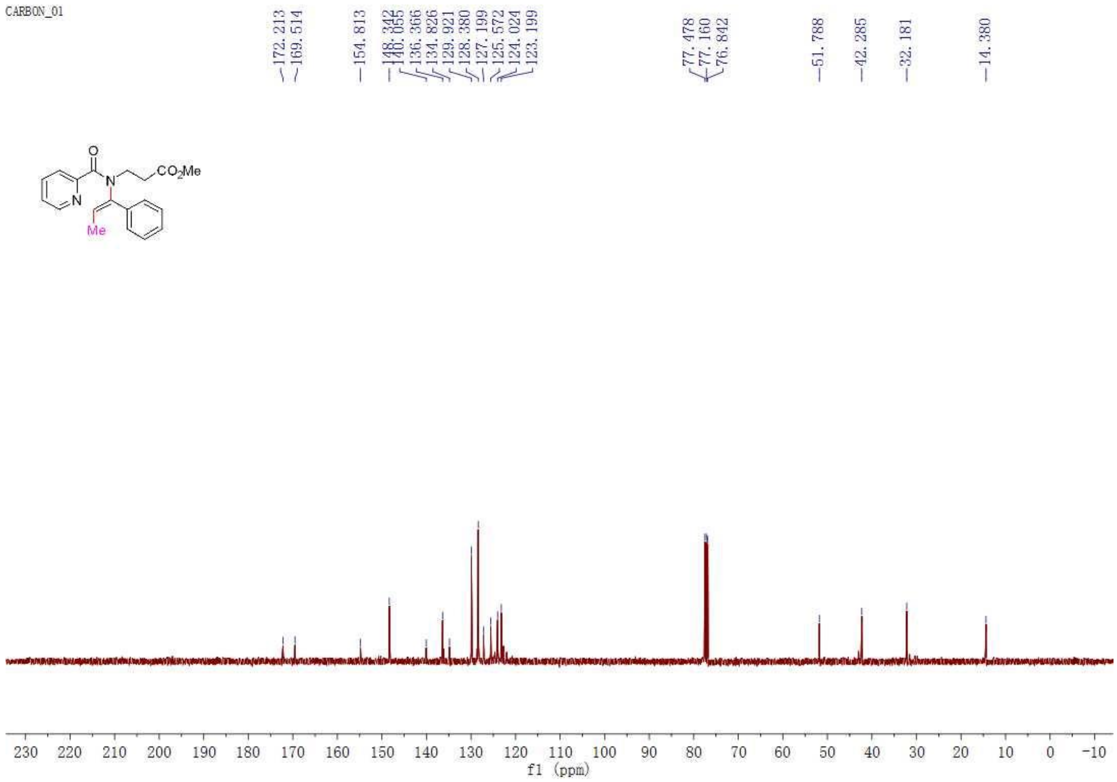

^{13}C NMR spectra for compound **5g** (100 MHz, $CDCl_3$)

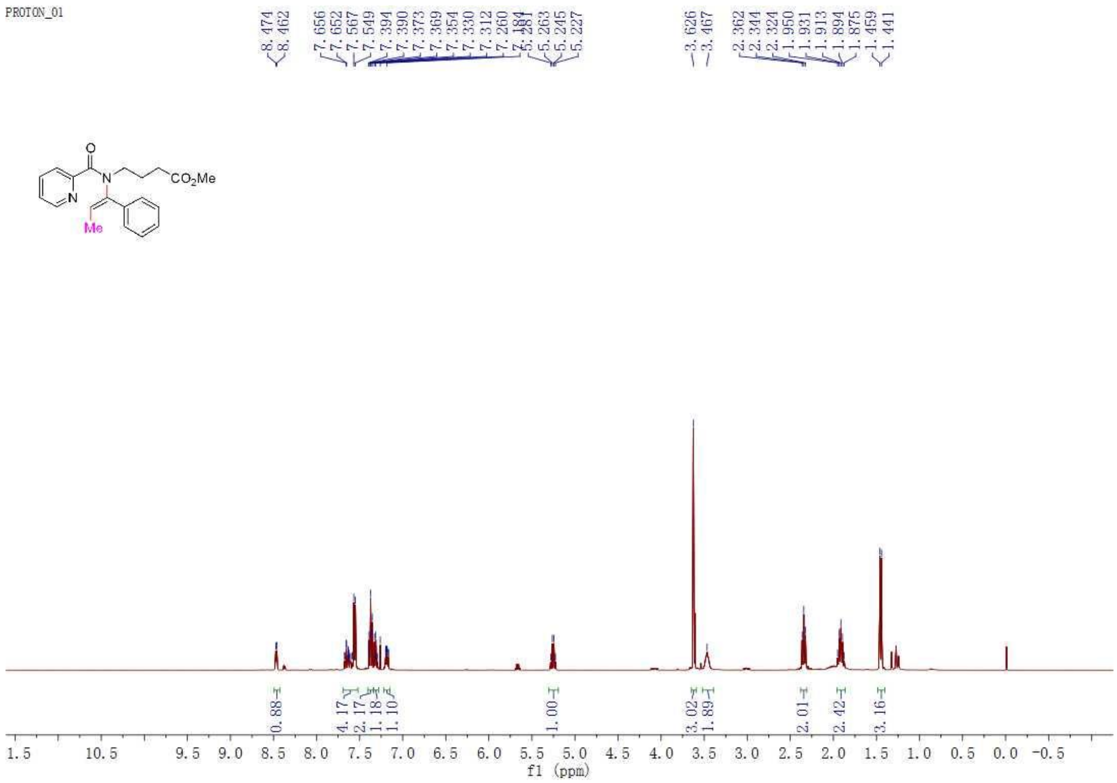

^{1}H NMR spectra for compound **5h** (400 MHz, $CDCl_3$)

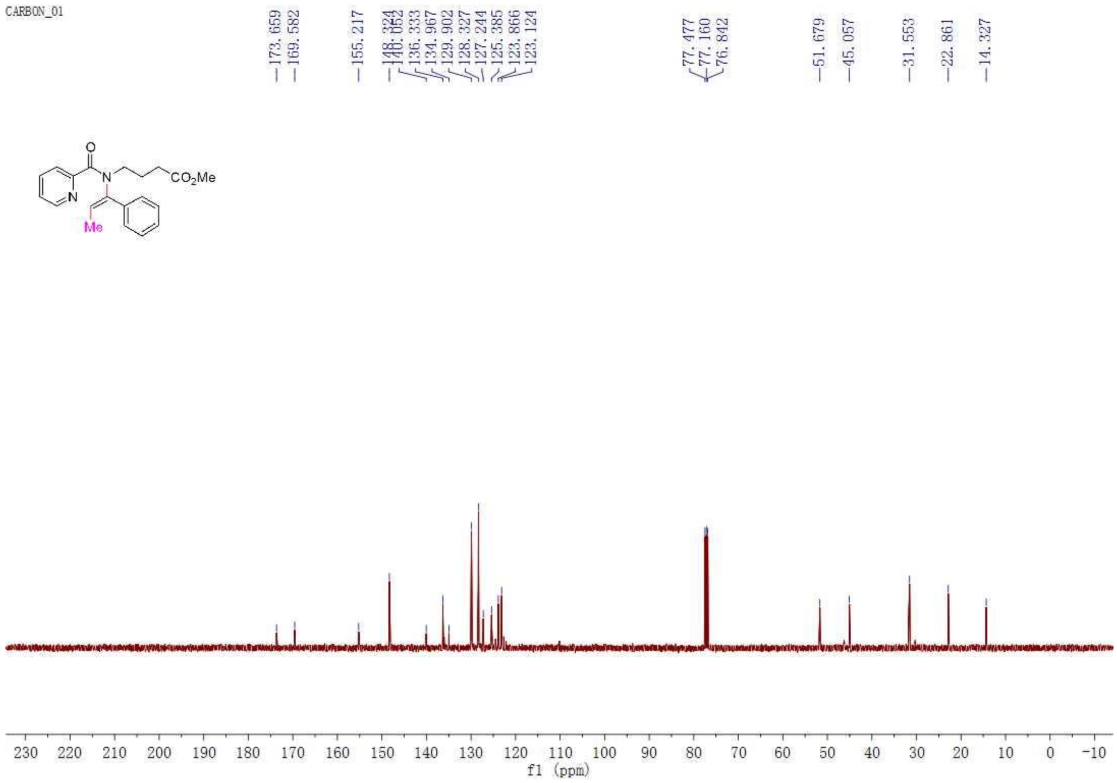

^{13}C NMR spectra for compound **5h** (100 MHz, $CDCl_3$)

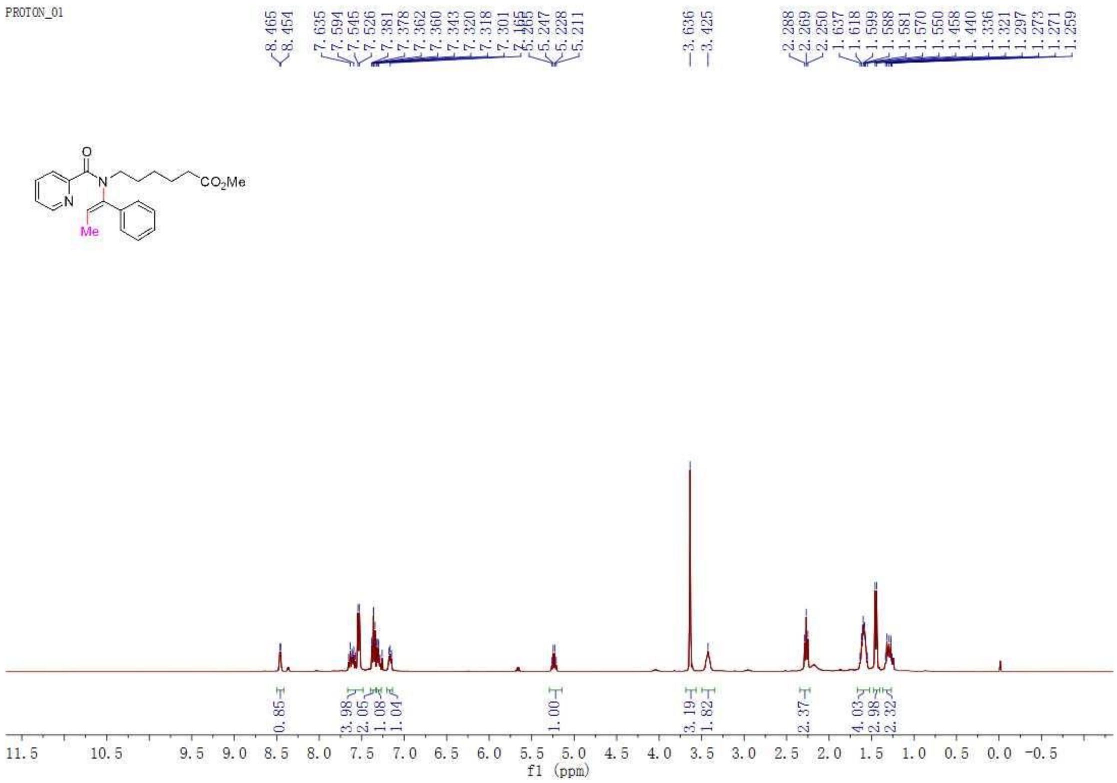

^{1}H NMR spectra for compound **5i** (400 MHz, $CDCl_3$)

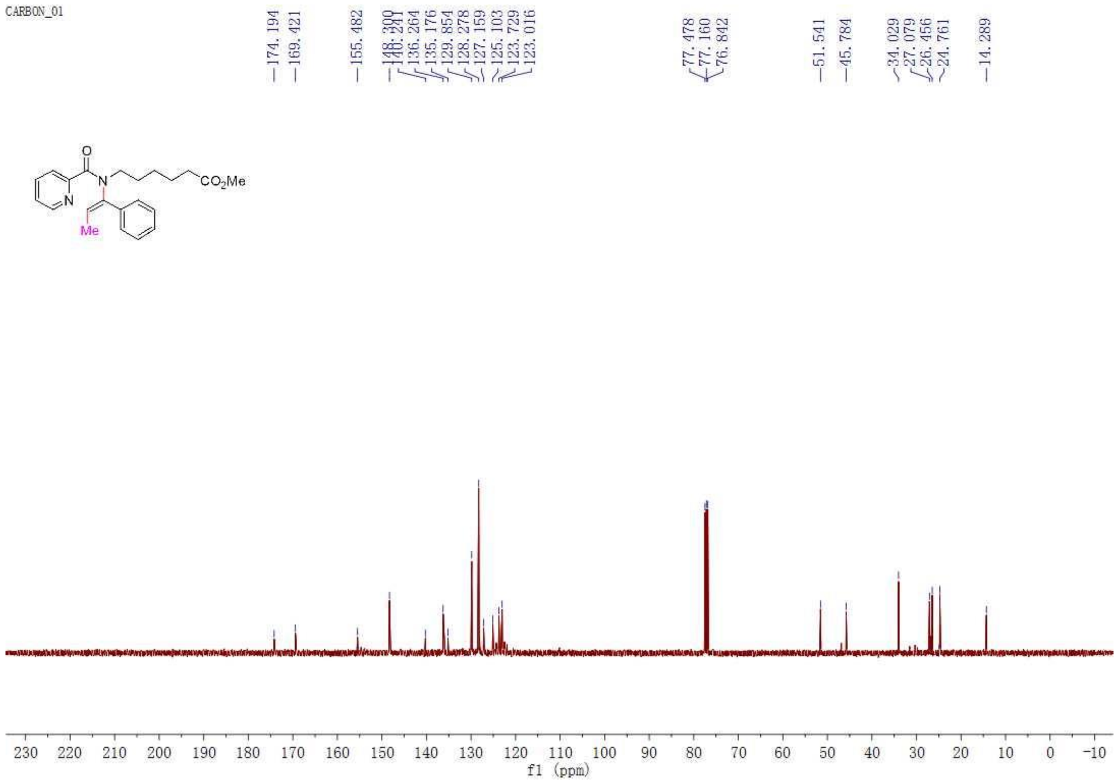

^{13}C NMR spectra for compound **5i** (100 MHz, $CDCl_3$)

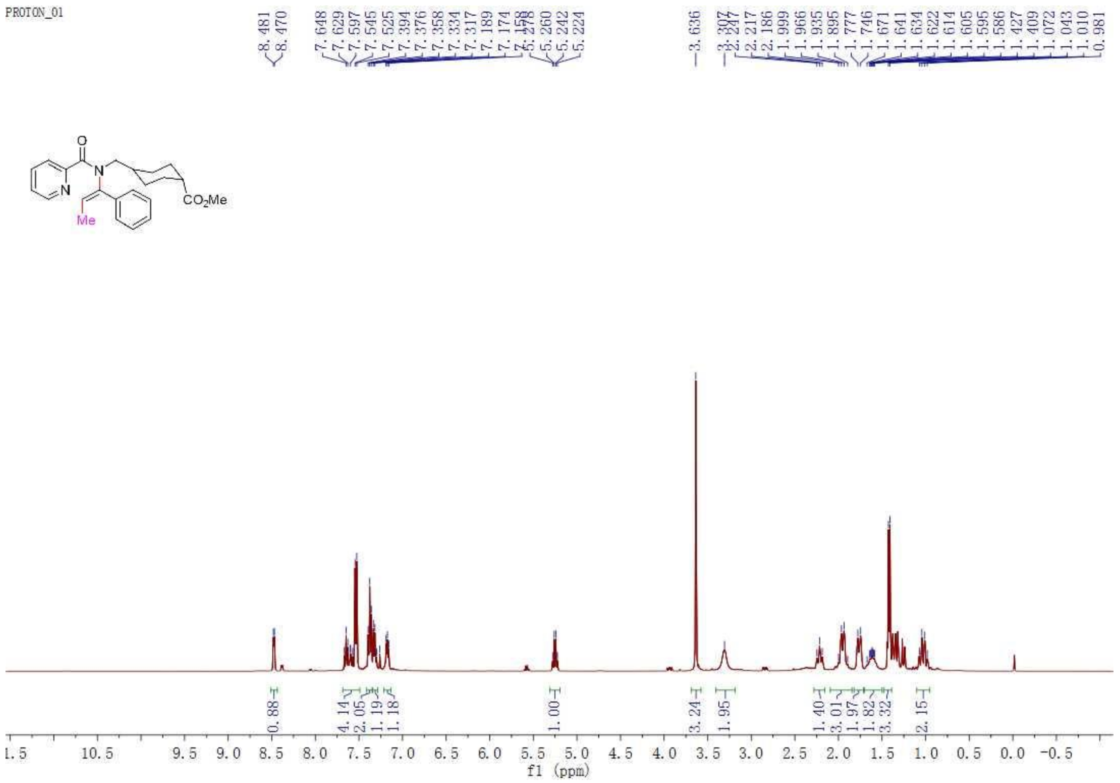

^{1}H NMR spectra for compound **5j** (400 MHz, $CDCl_3$)

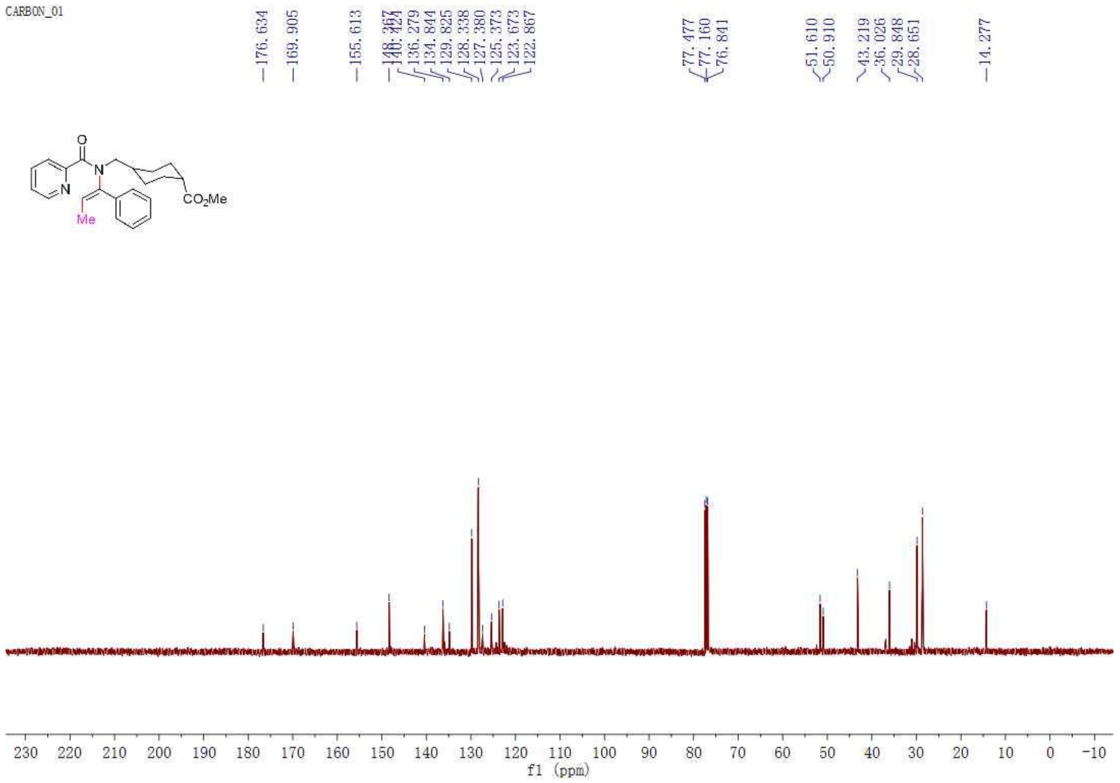

^{13}C NMR spectra for compound **5j** (100 MHz, $CDCl_3$)

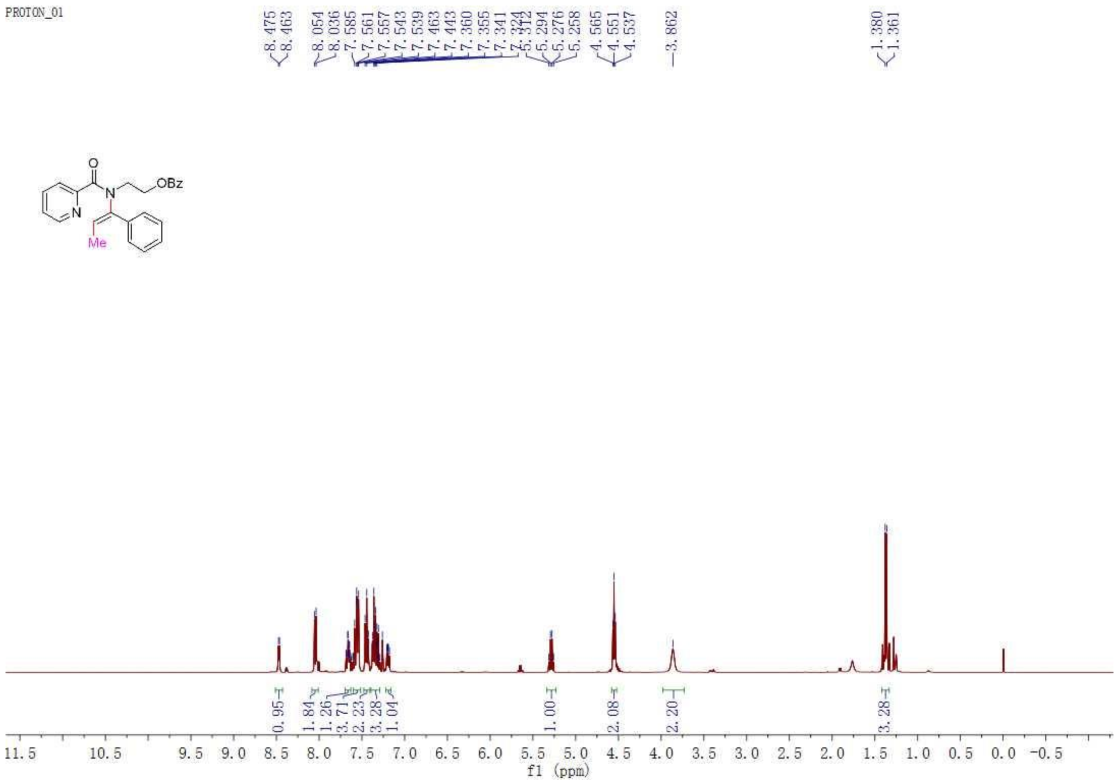

^{1}H NMR spectra for compound **5k** (400 MHz, $CDCl_3$)

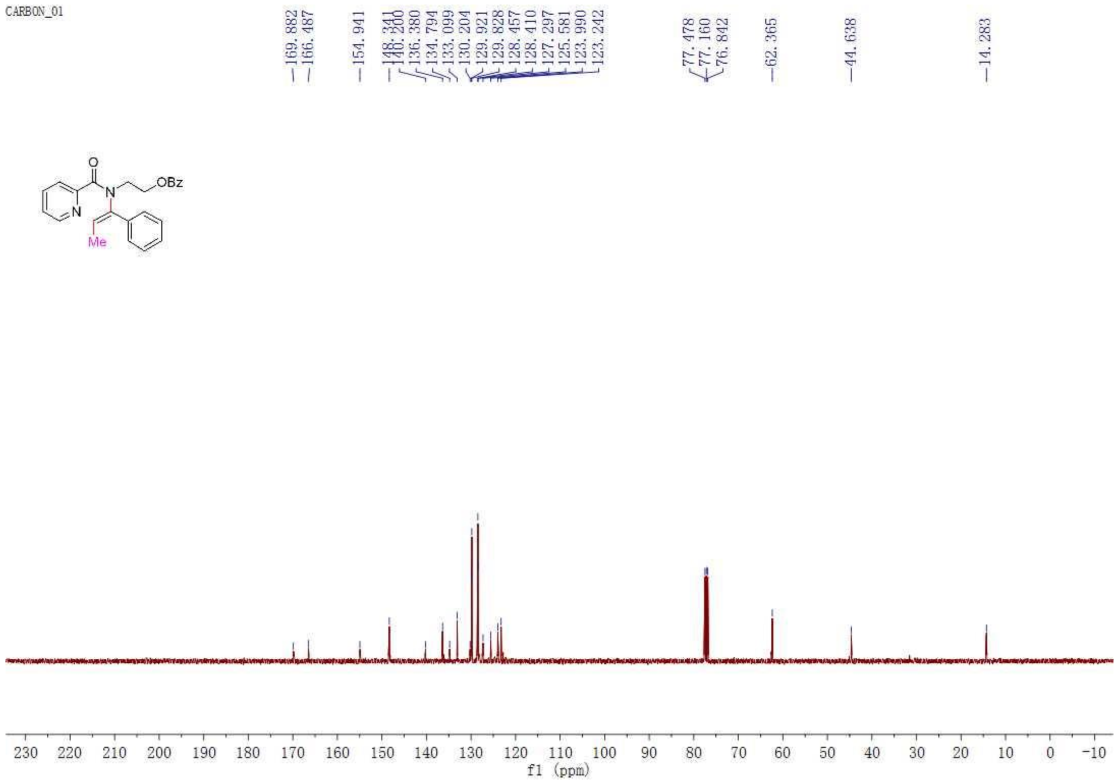

^{13}C NMR spectra for compound **5k** (100 MHz, $CDCl_3$)

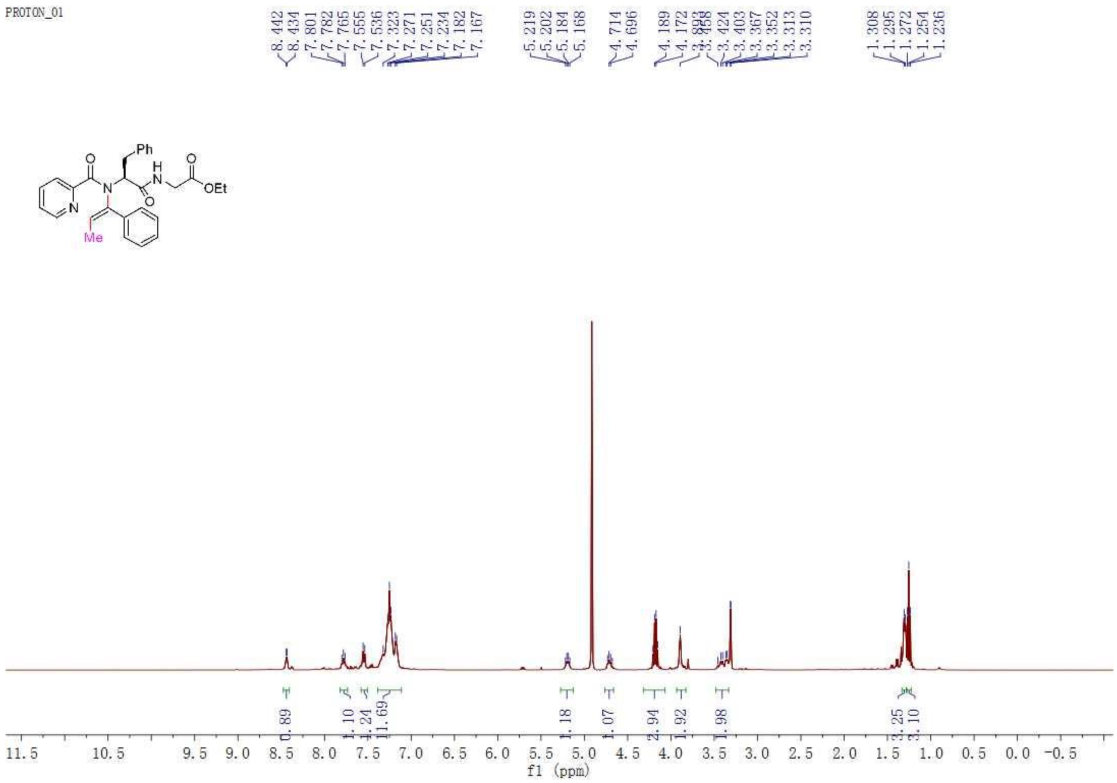

^{1}H NMR spectra for compound **6a** (400 MHz, CD_3OD)

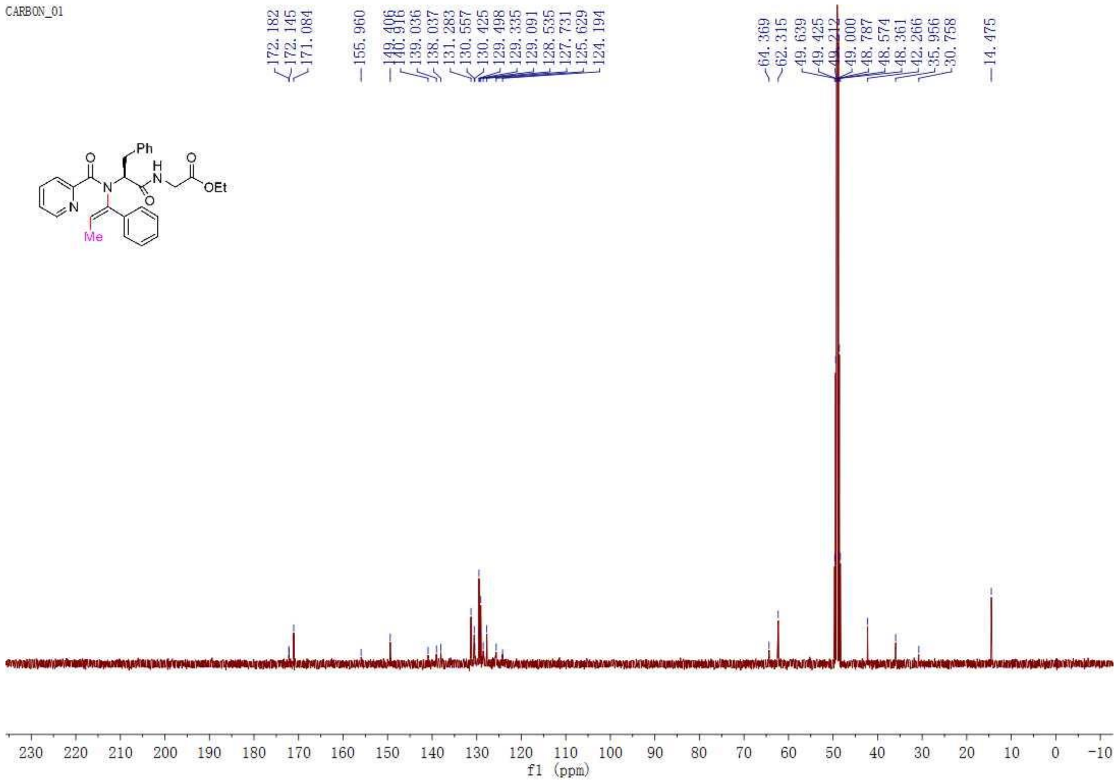

^{13}C NMR spectra for compound **6a** (100 MHz, CD_3OD)

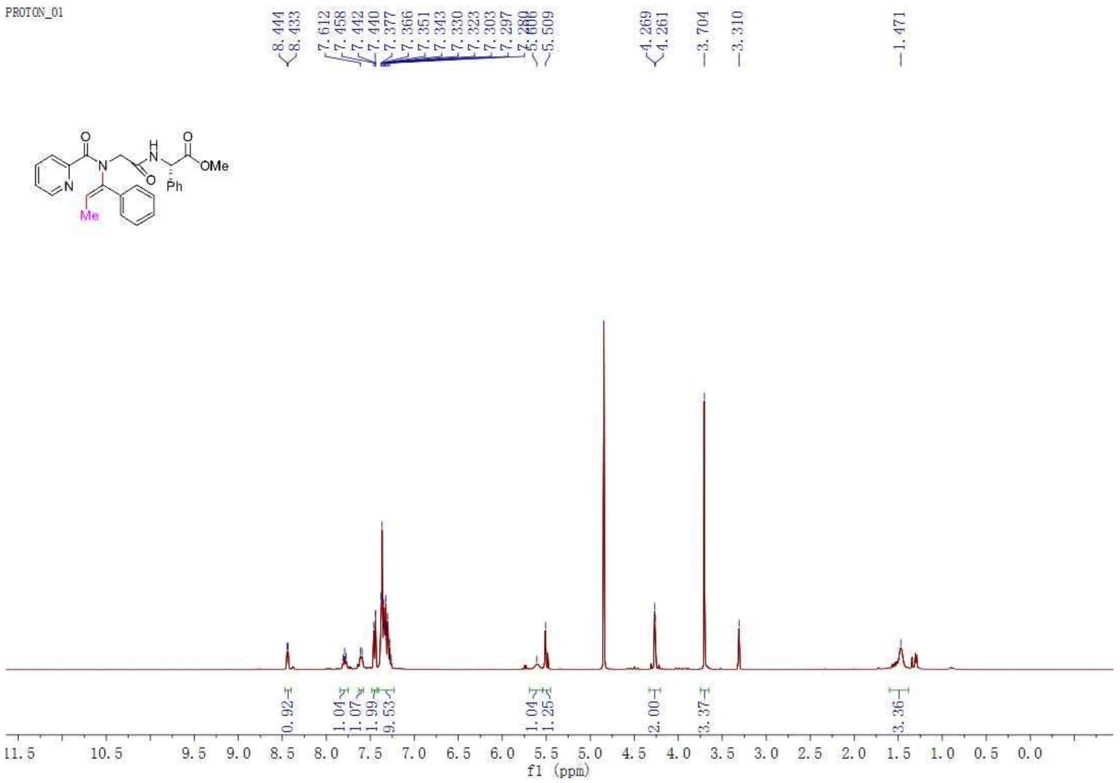

^{1}H NMR spectra for compound **6b** (400 MHz, CD_3OD)

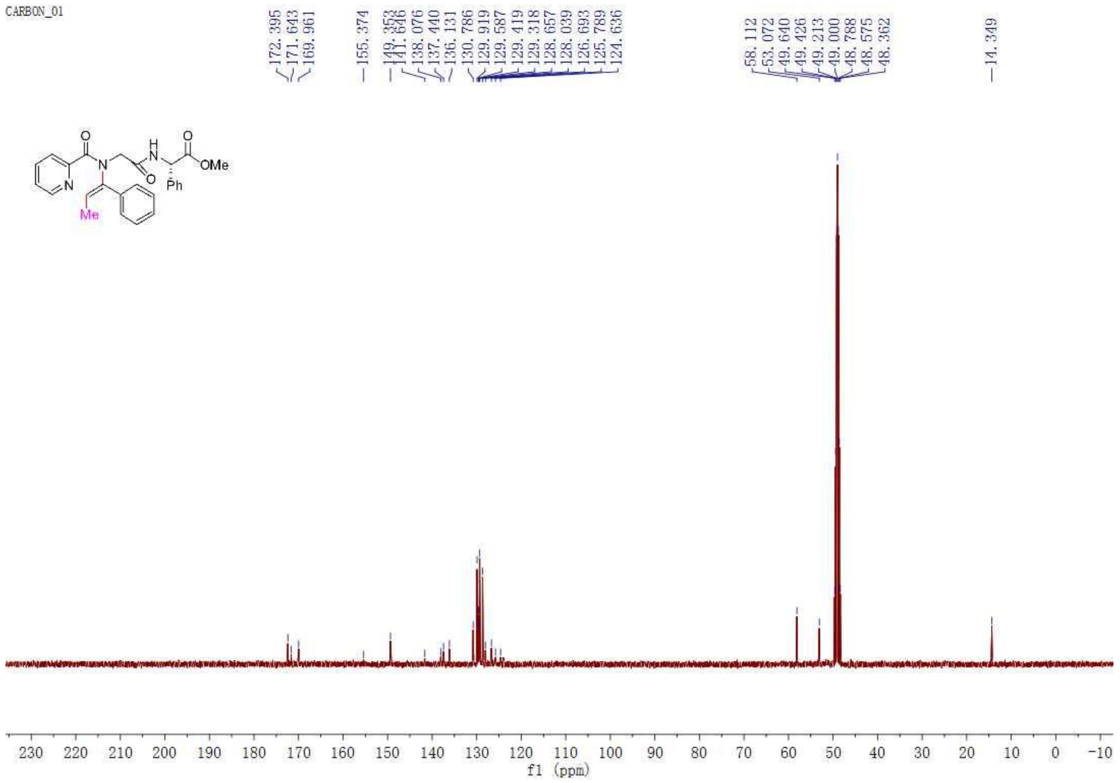

^{13}C NMR spectra for compound **6b** (100 MHz, CD_3OD)

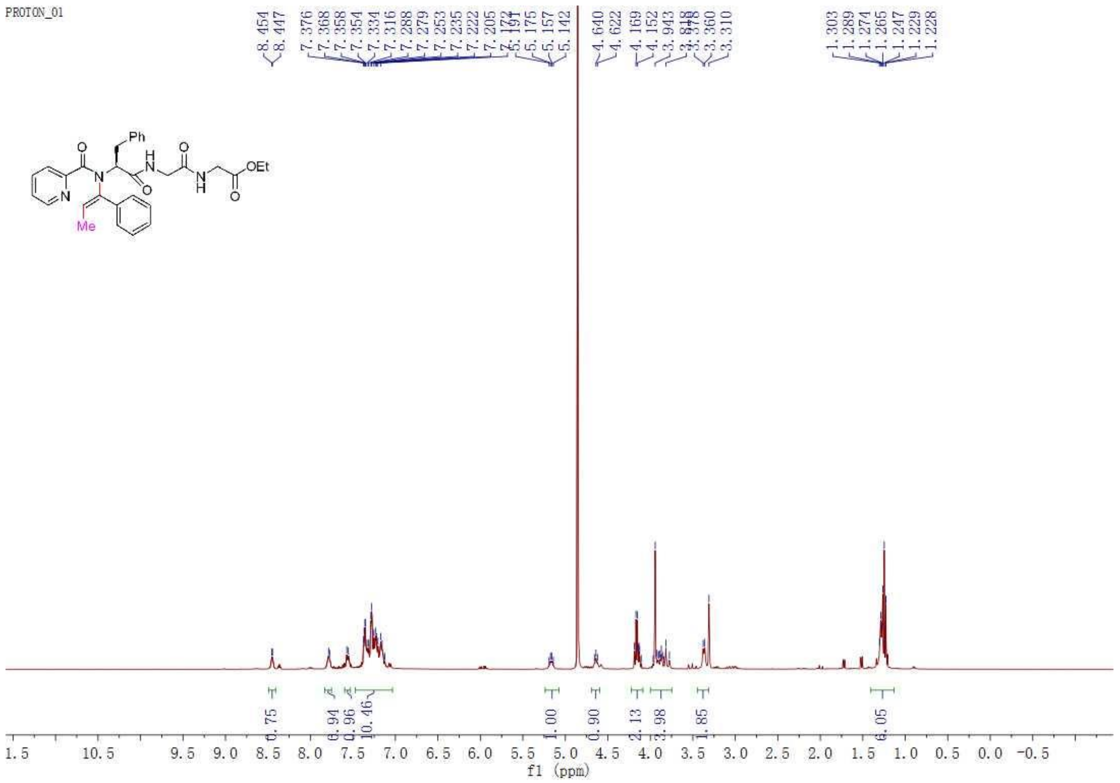

^{1}H NMR spectra for compound **6c** (400 MHz, CD_3OD)

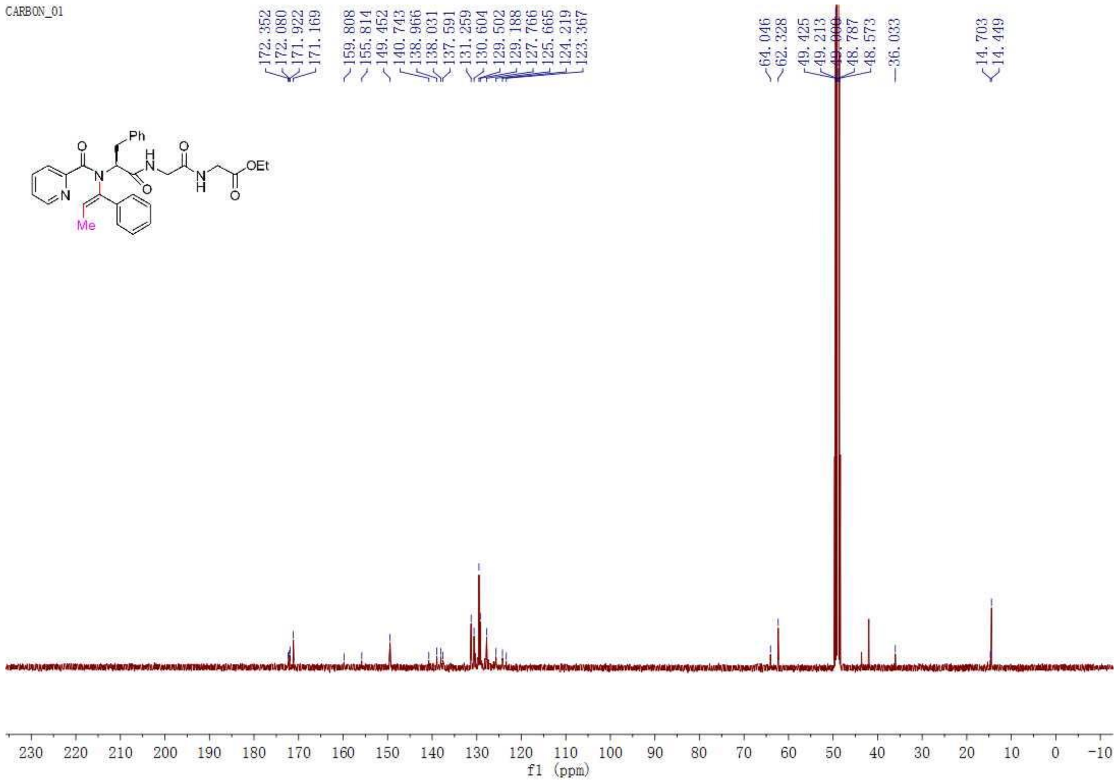

^{13}C NMR spectra for compound **6c** (100 MHz, CD_3OD)

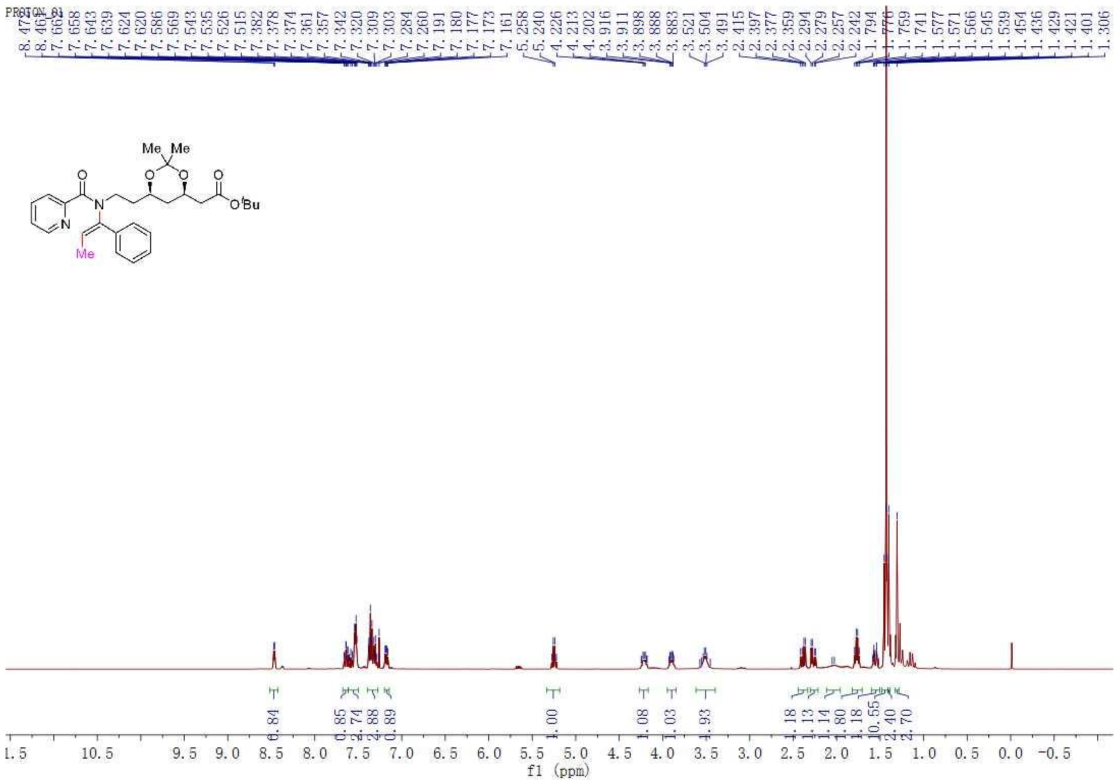

^{1}H NMR spectra for compound **6d** (400 MHz, $CDCl_3$)

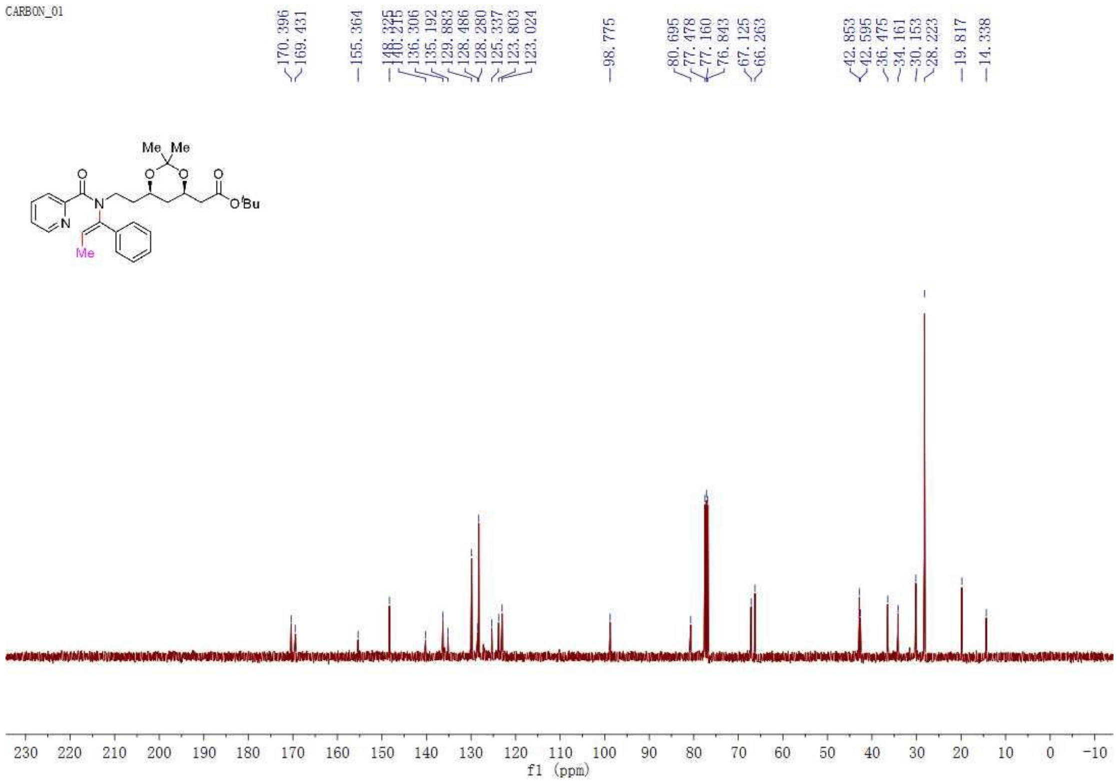

^{13}C NMR spectra for compound **6d** (100 MHz, $CDCl_3$)

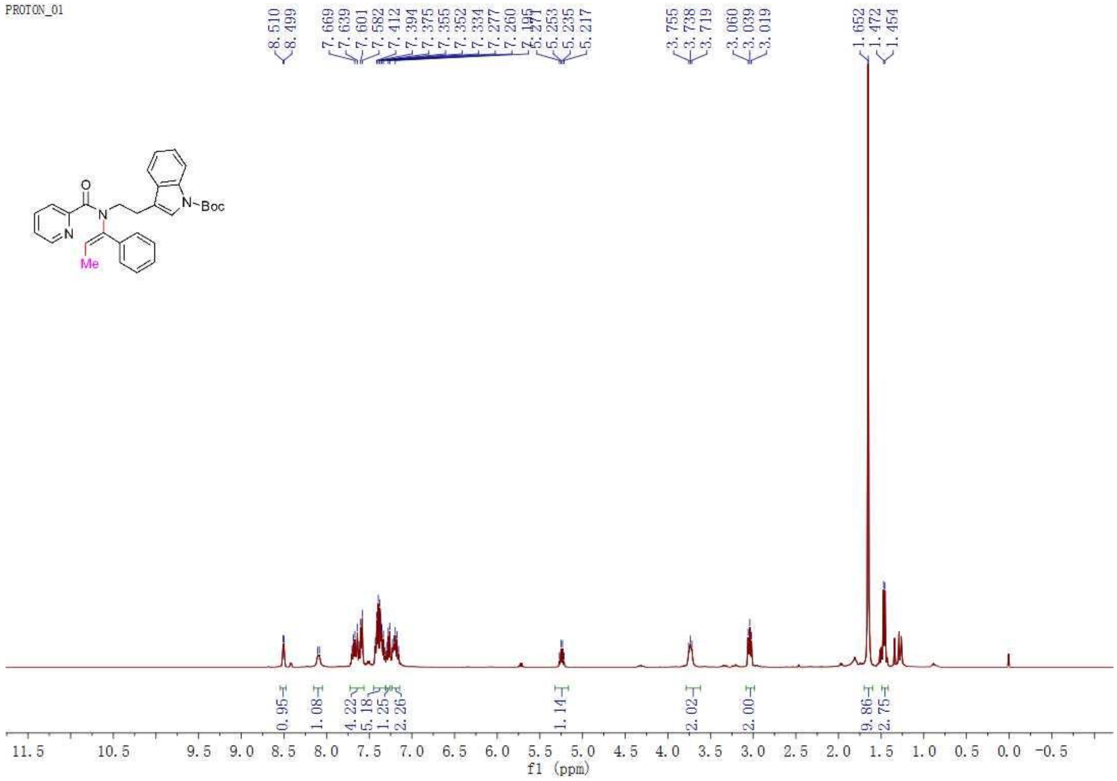

^{1}H NMR spectra for compound **6e** (400 MHz, $CDCl_3$)

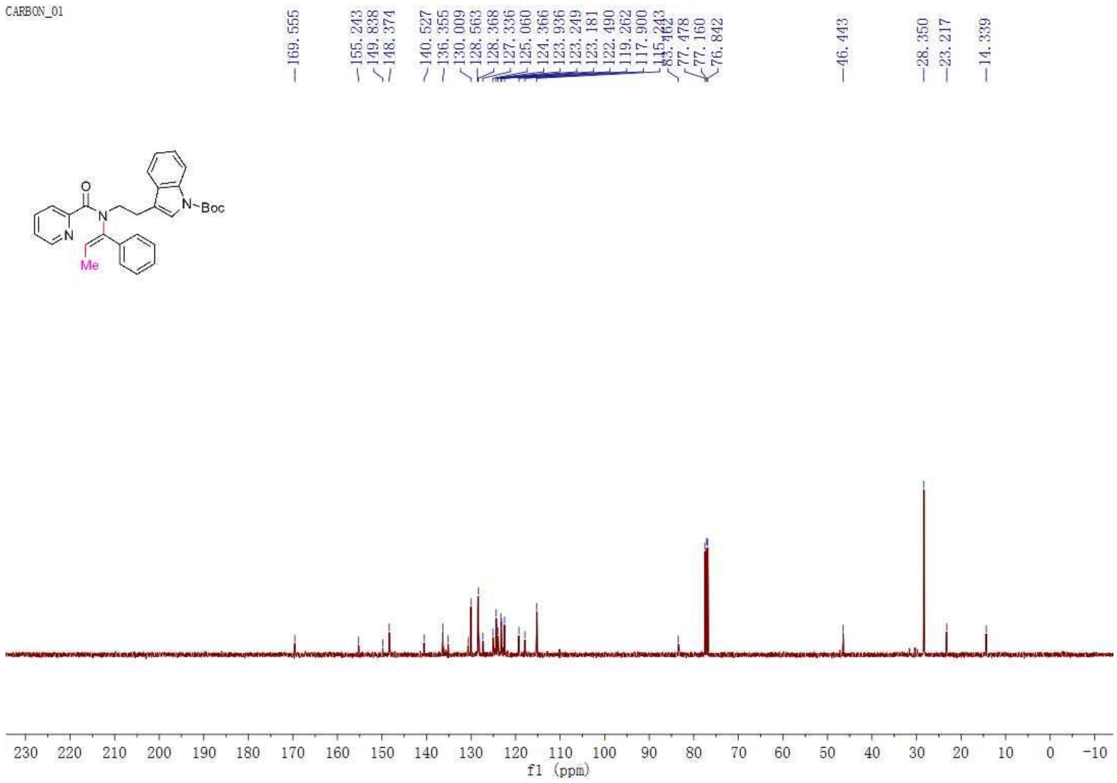

^{13}C NMR spectra for compound **6e** (100 MHz, $CDCl_3$)

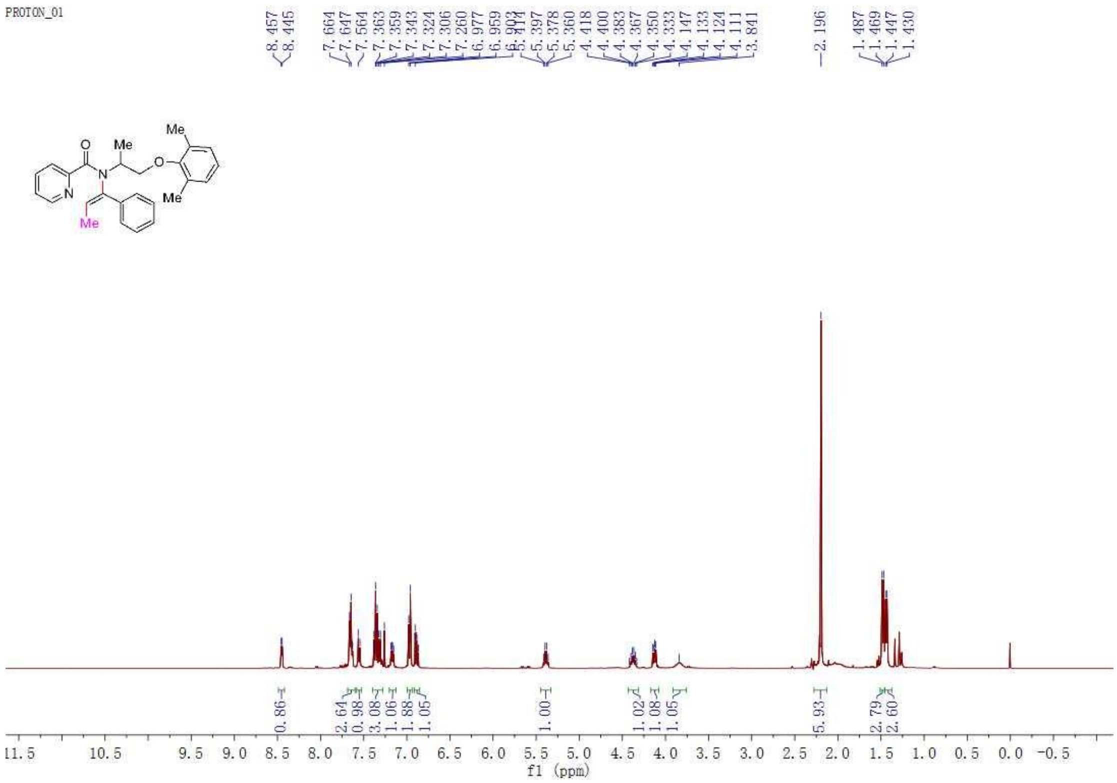

^{1}H NMR spectra for compound **6f** (400 MHz, $CDCl_3$)

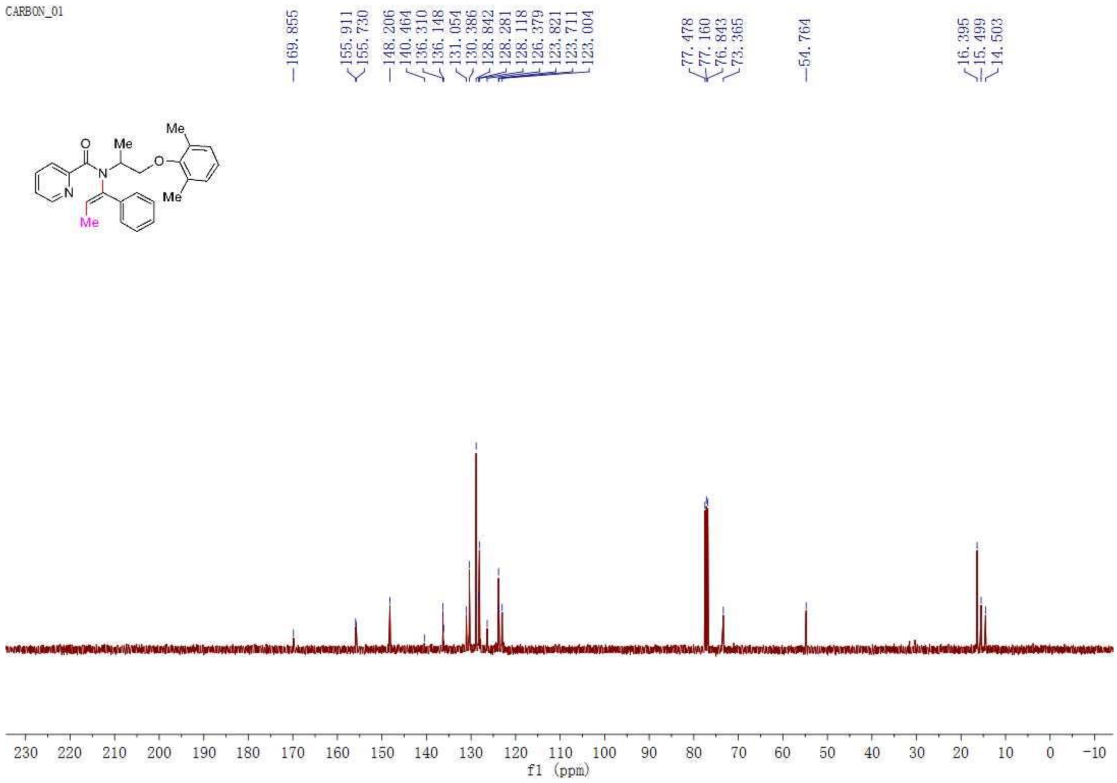

^{13}C NMR spectra for compound **6f** (100 MHz, $CDCl_3$)

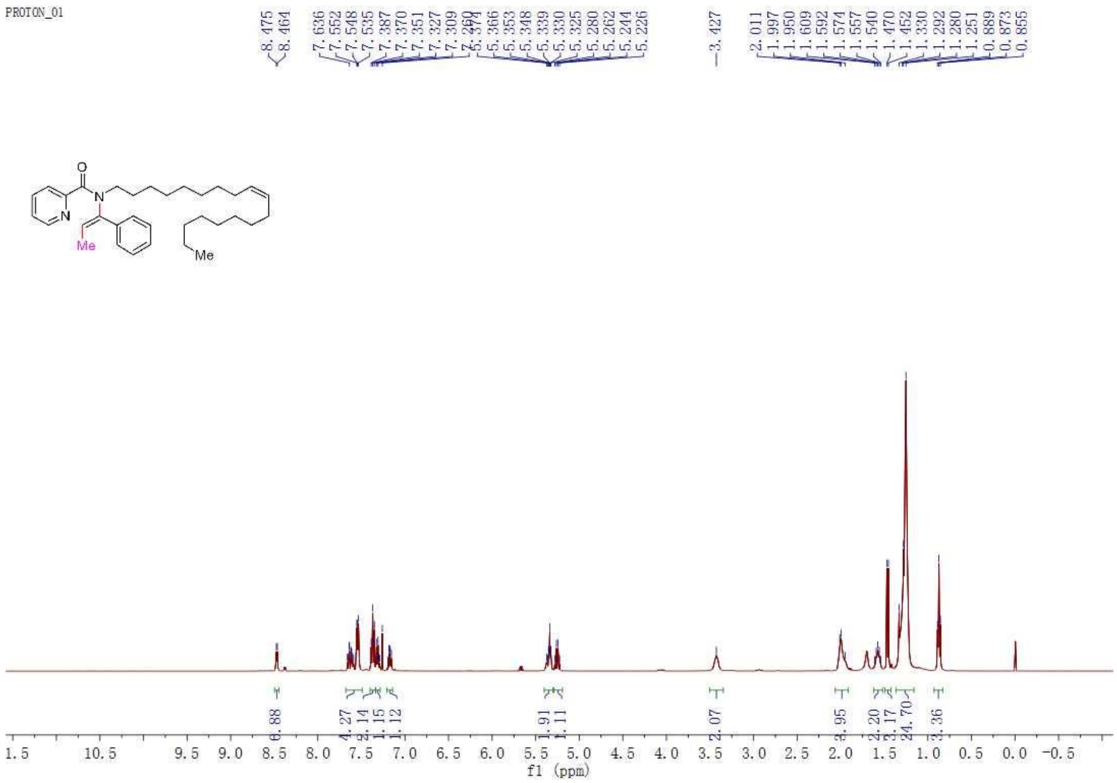

^{1}H NMR spectra for compound **6g** (400 MHz, $CDCl_3$)

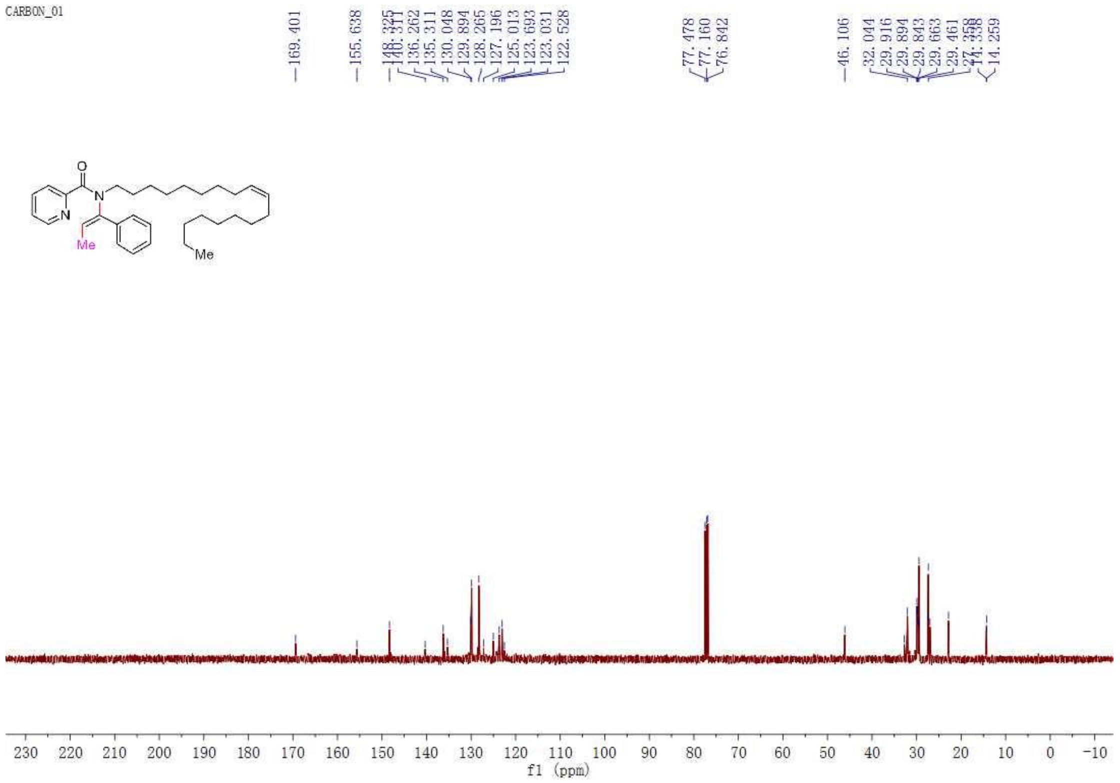

^{13}C NMR spectra for compound **6g** (100 MHz, $CDCl_3$)

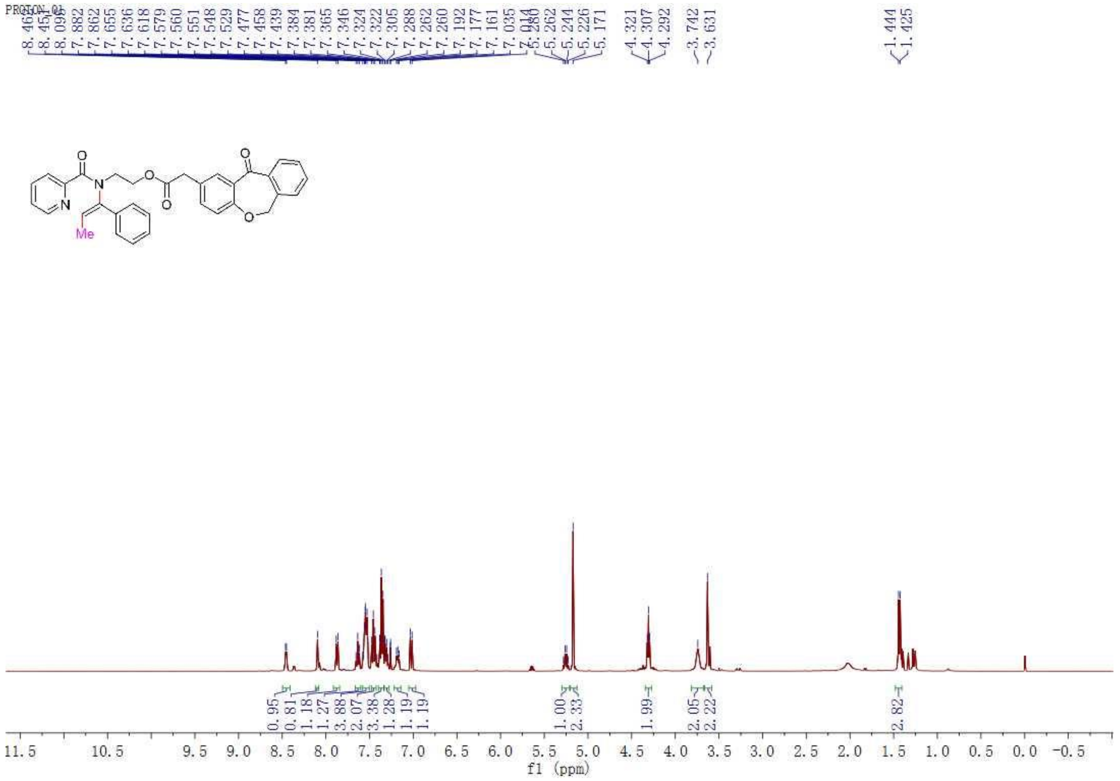

^{1}H NMR spectra for compound **6h** (400 MHz, $CDCl_3$)

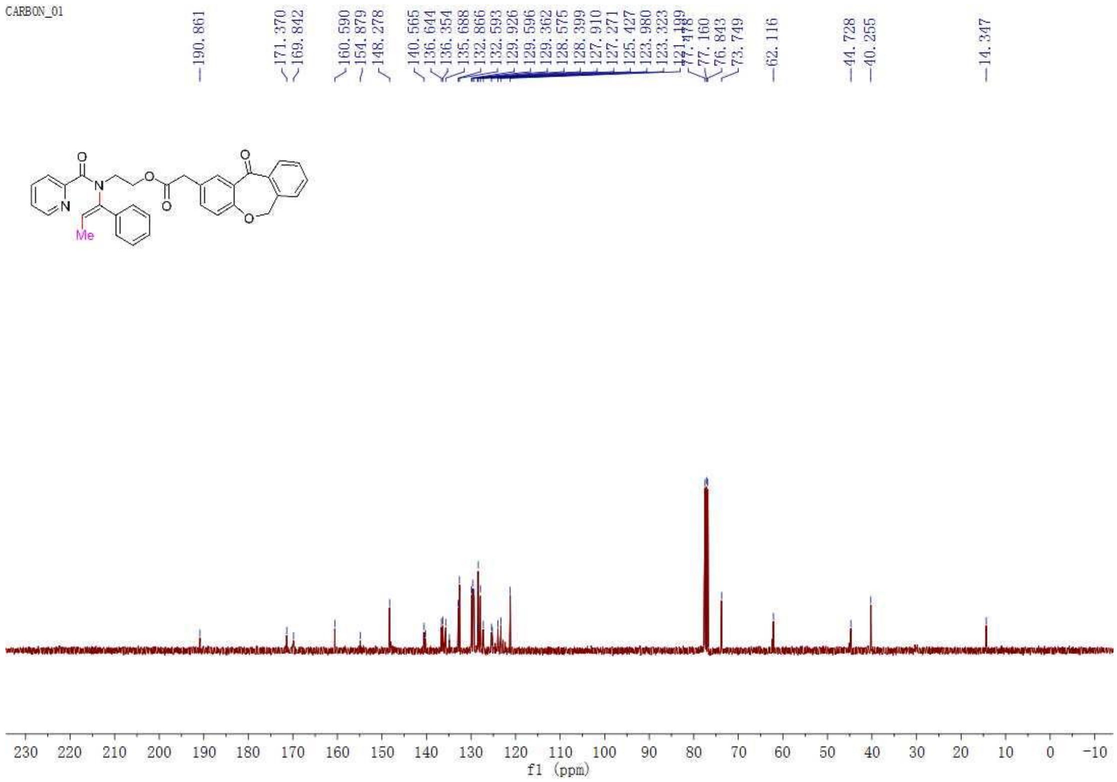

^{13}C NMR spectra for compound **6h** (100 MHz, $CDCl_3$)

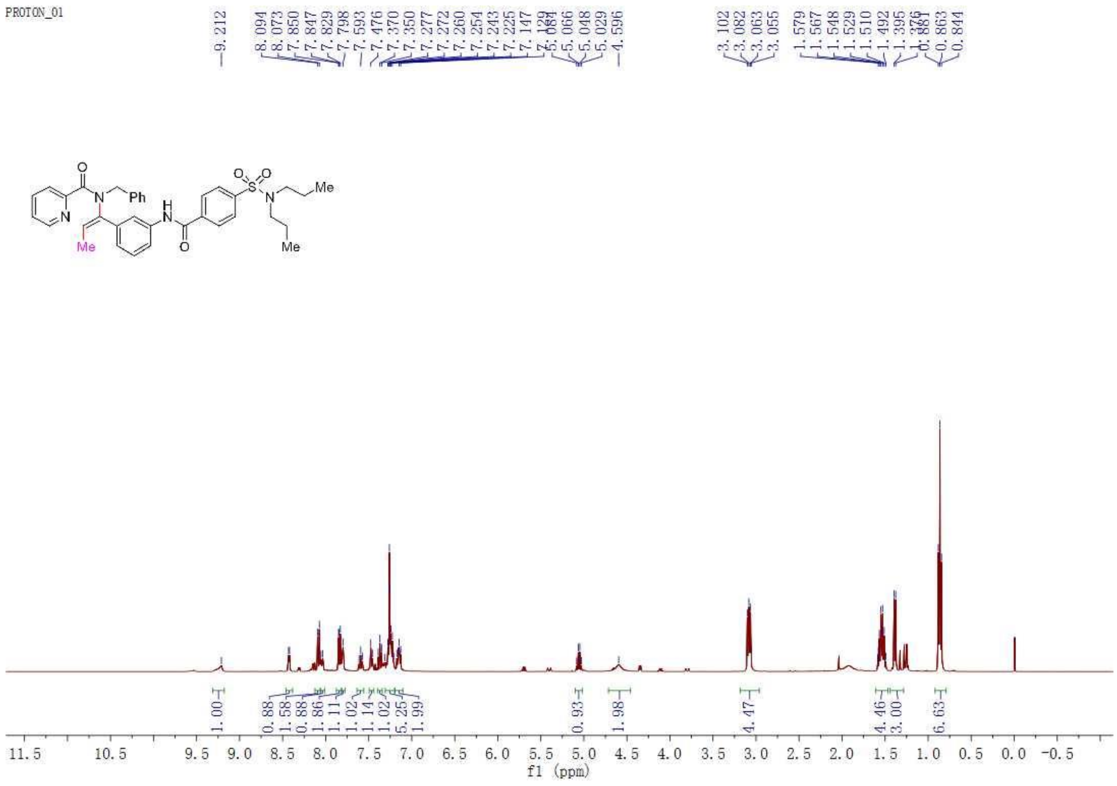

^{1}H NMR spectra for compound **6i** (400 MHz, $CDCl_3$)

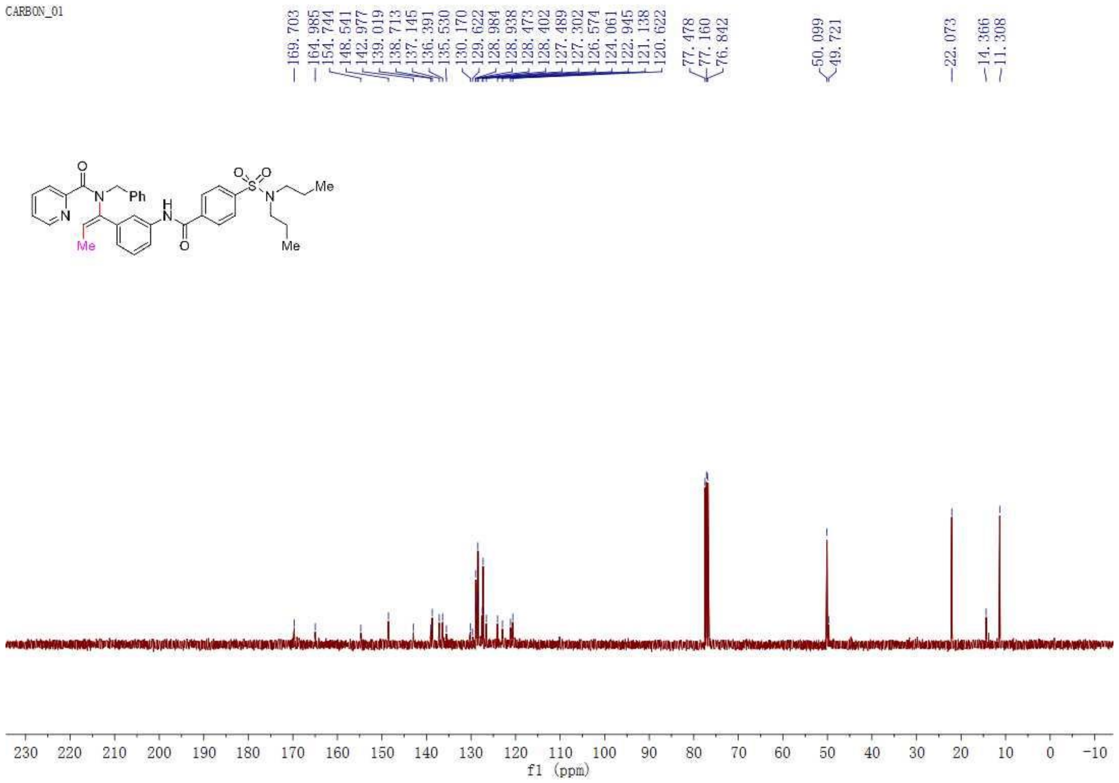

^{13}C NMR spectra for compound **6i** (100 MHz, $CDCl_3$)

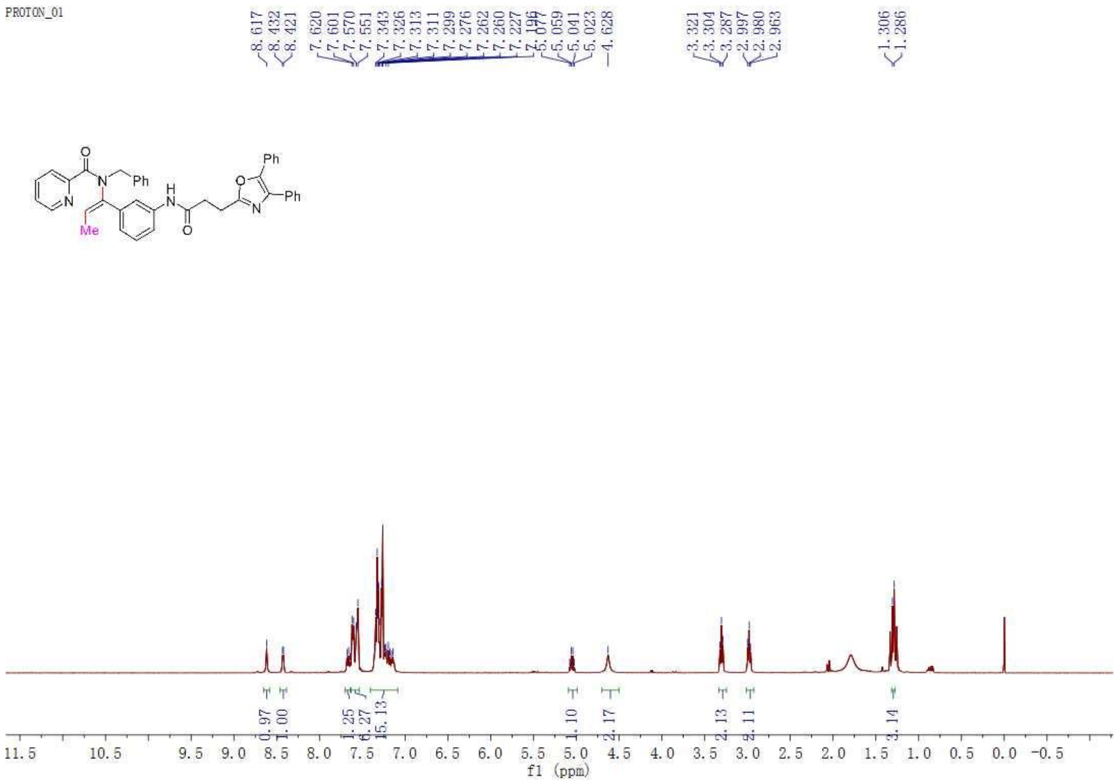

^{1}H NMR spectra for compound **6j** (400 MHz, $CDCl_3$)

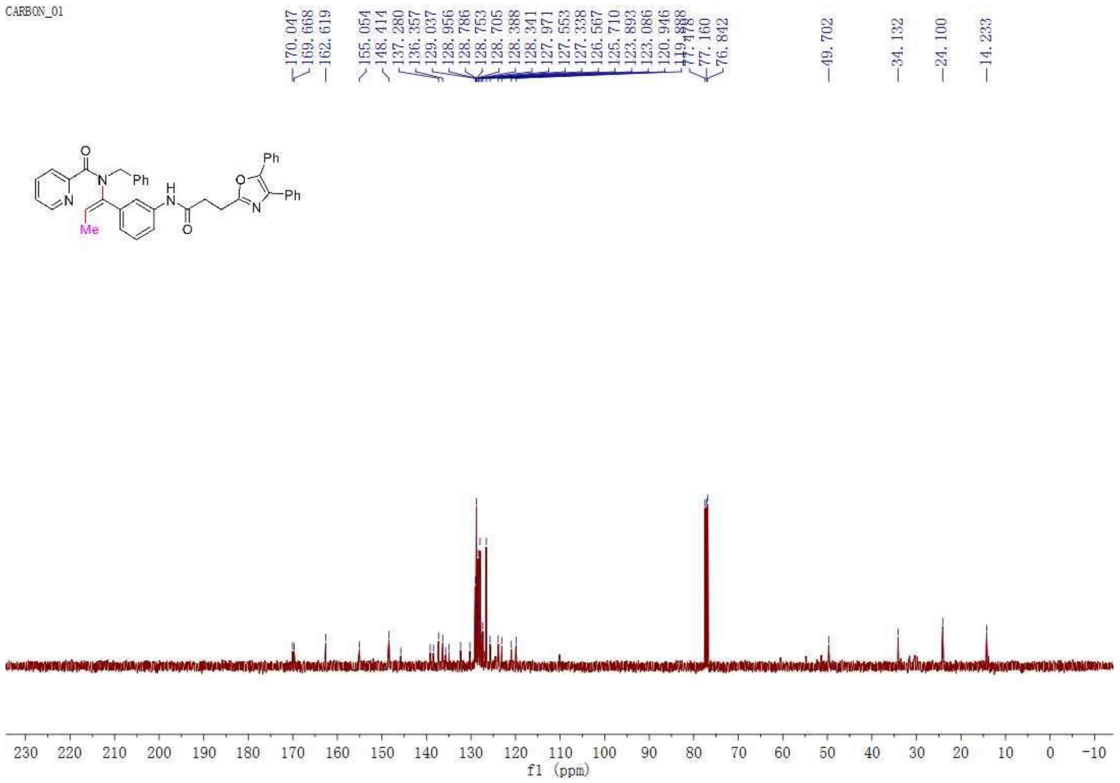

^{13}C NMR spectra for compound **6j** (100 MHz, $CDCl_3$)

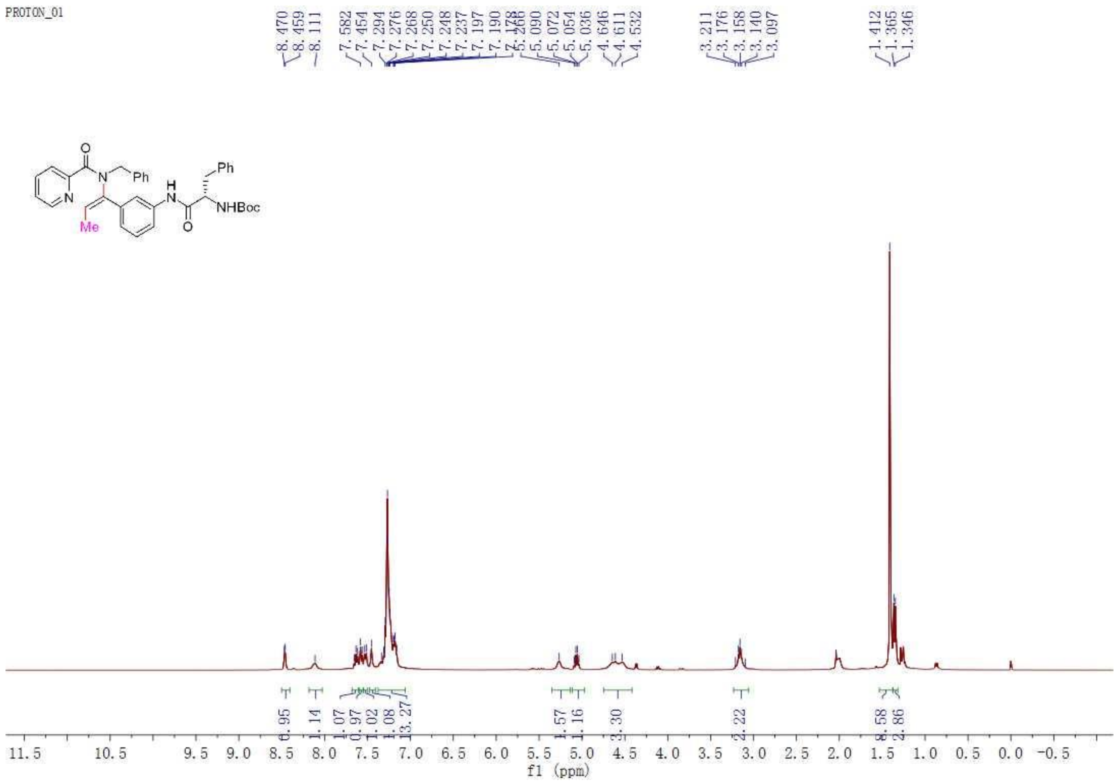

^{1}H NMR spectra for compound **6k** (400 MHz, $CDCl_3$)

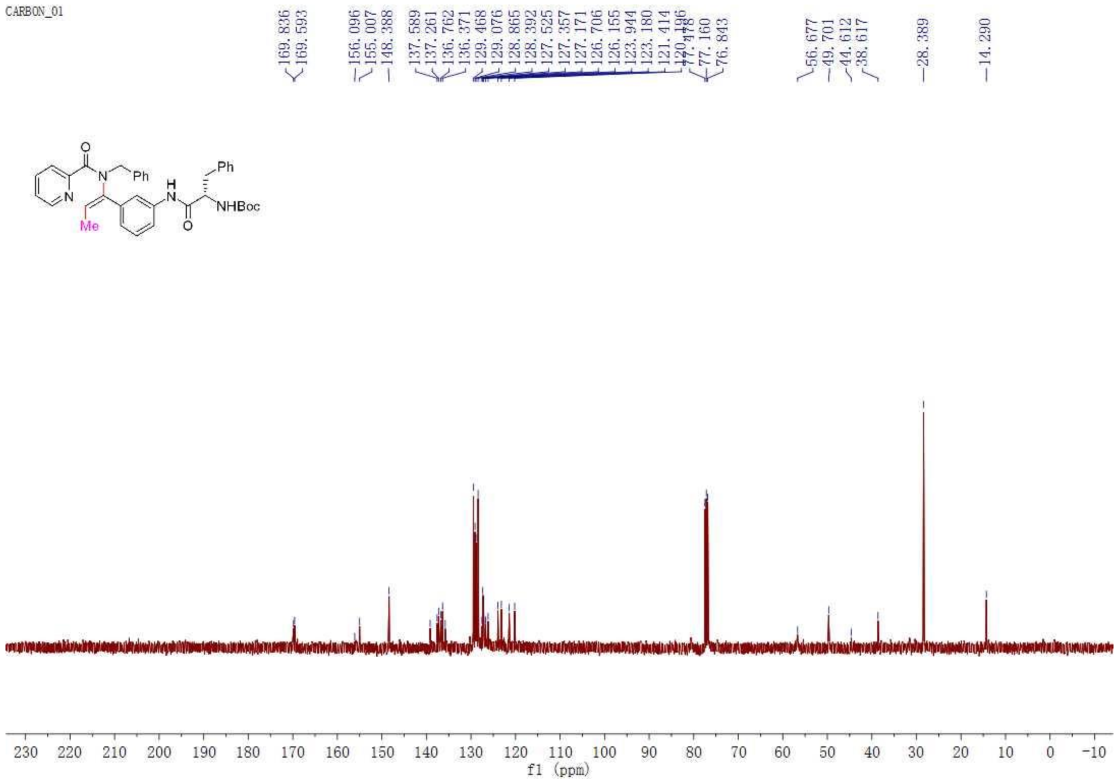

^{13}C NMR spectra for compound **6k** (100 MHz, $CDCl_3$)

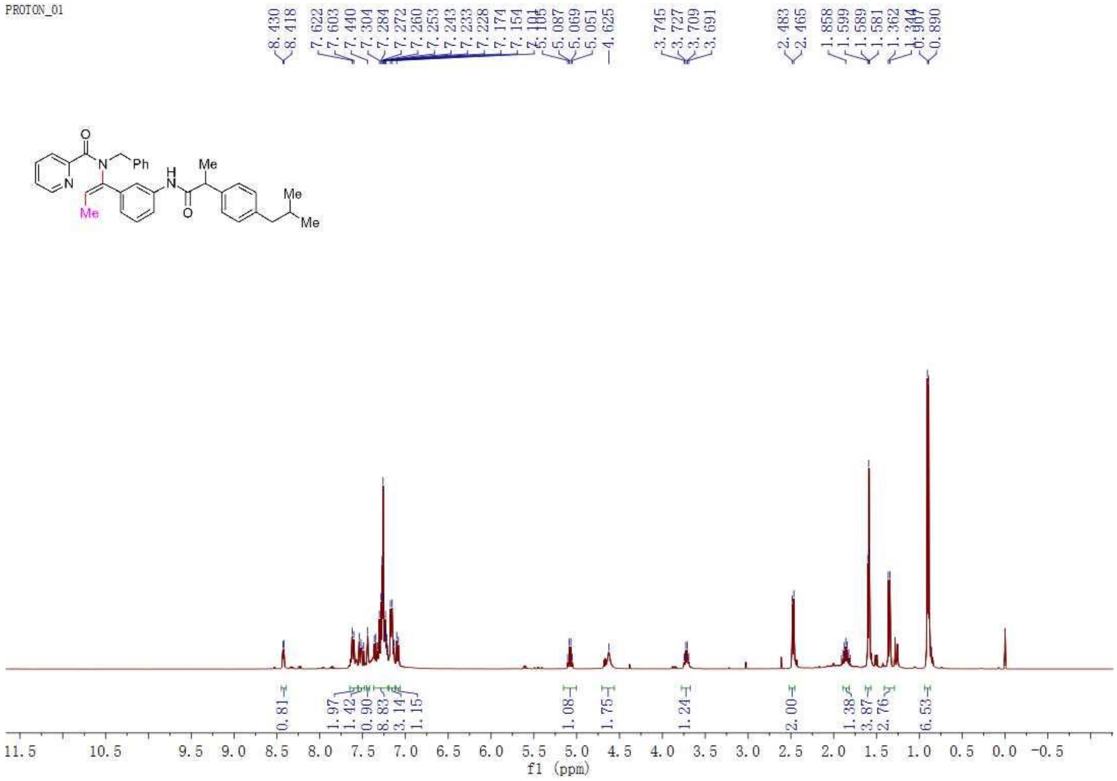

^{1}H NMR spectra for compound **6l** (400 MHz, $CDCl_3$)

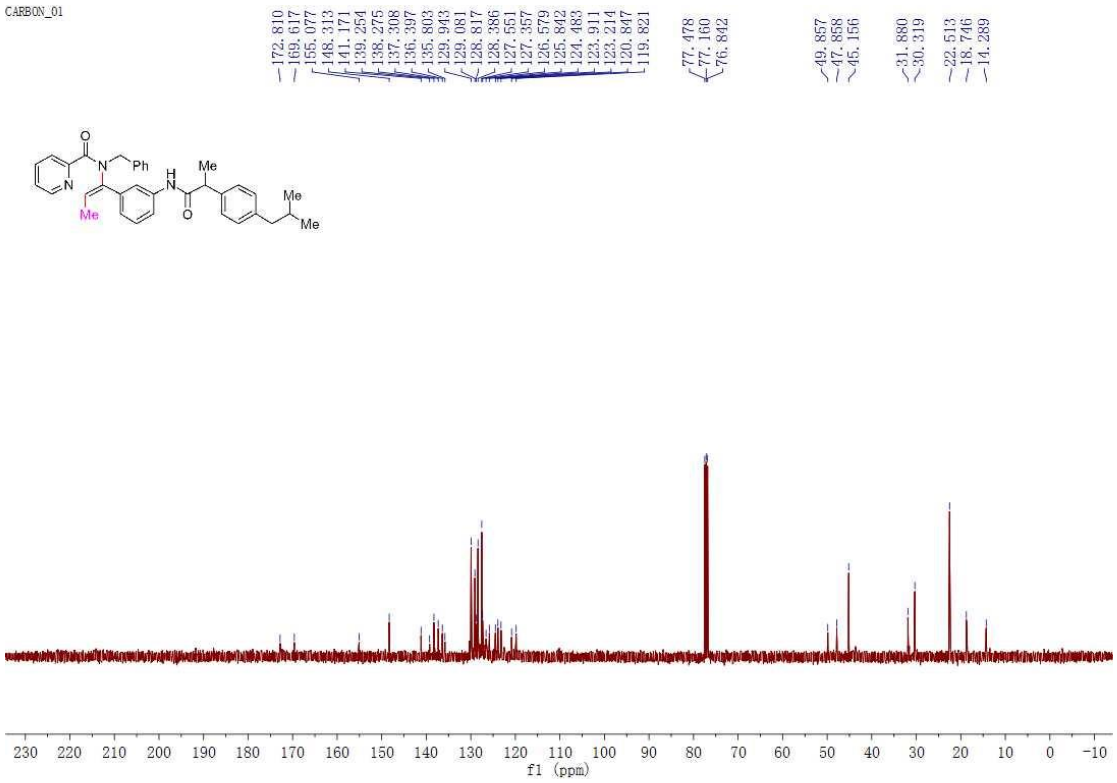

^{13}C NMR spectra for compound **6l** (100 MHz, $CDCl_3$)

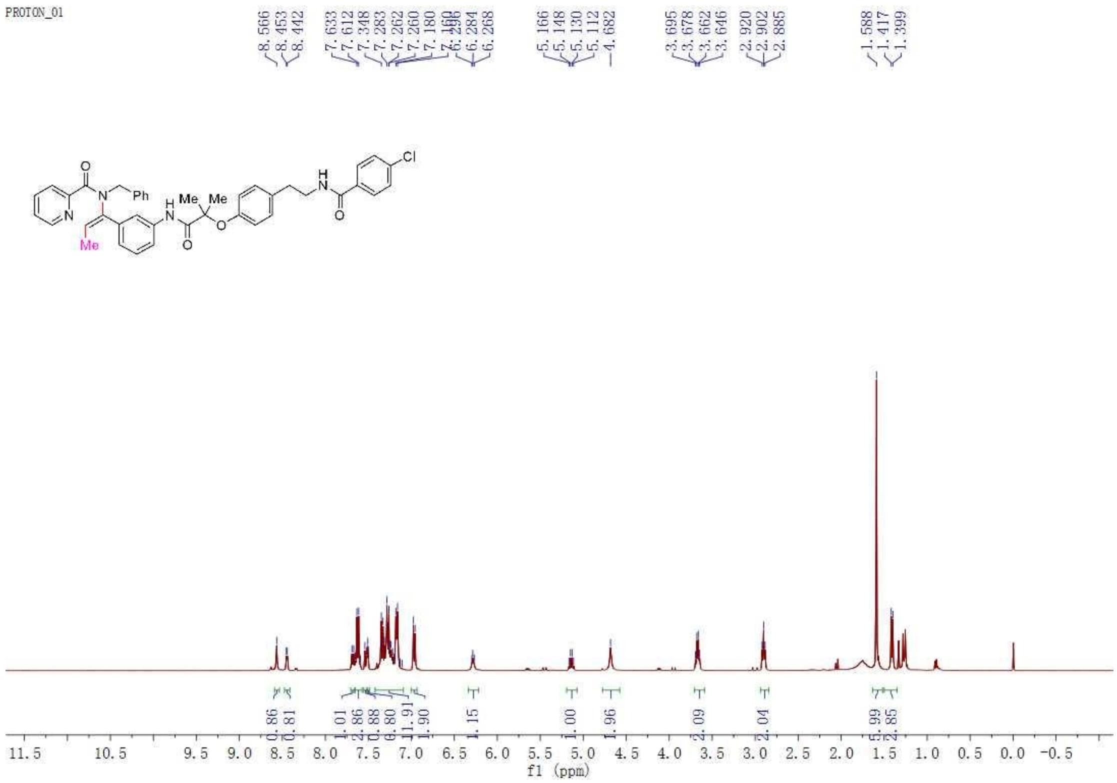

^{1}H NMR spectra for compound **6m** (400 MHz, $CDCl_3$)

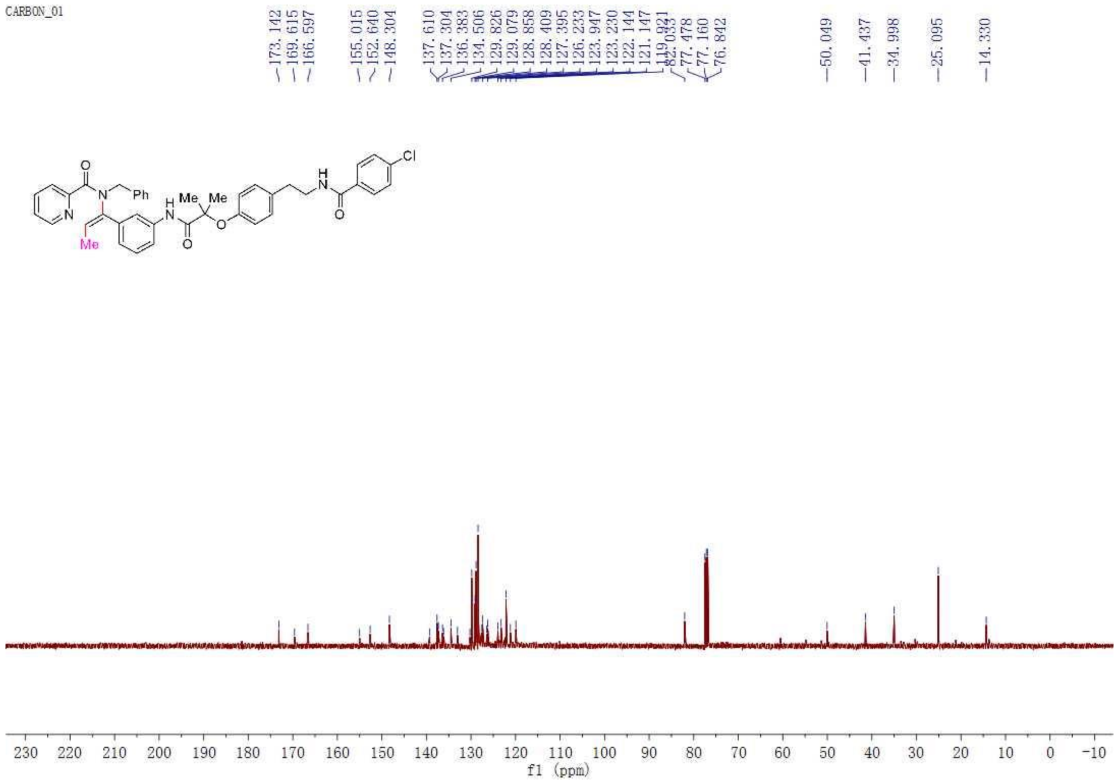

^{13}C NMR spectra for compound **6m** (100 MHz, $CDCl_3$)

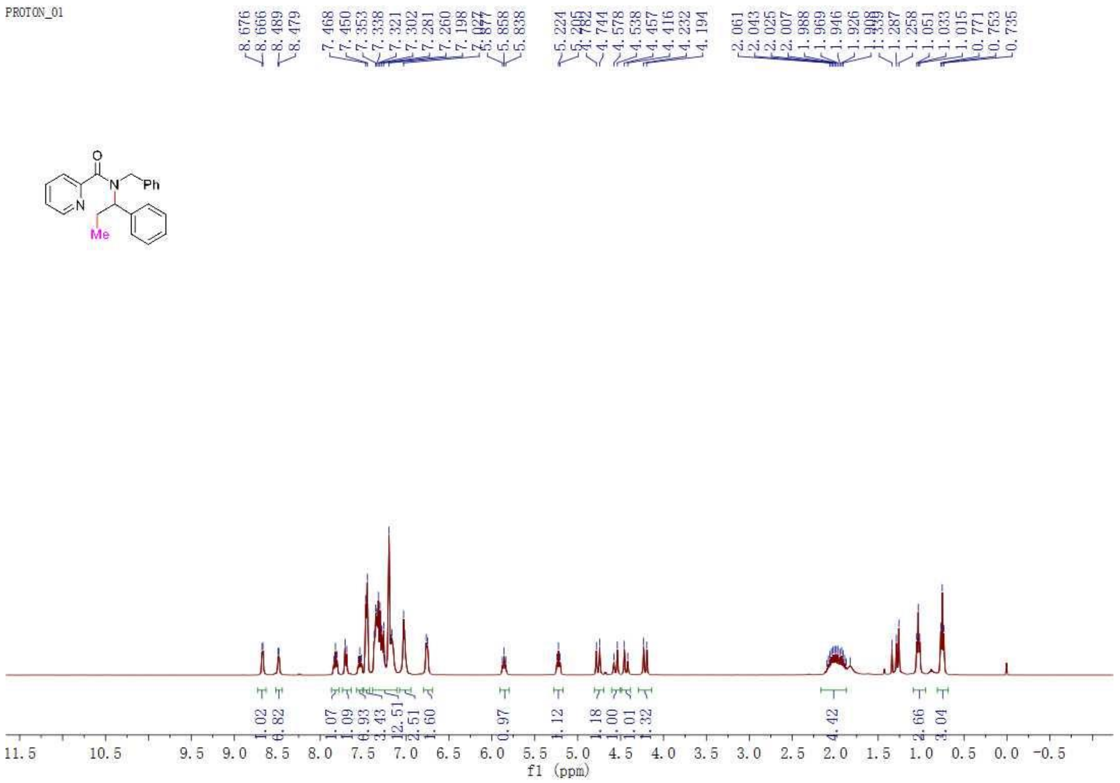

^{1}H NMR spectra for compound **7a** (400 MHz, $CDCl_3$)

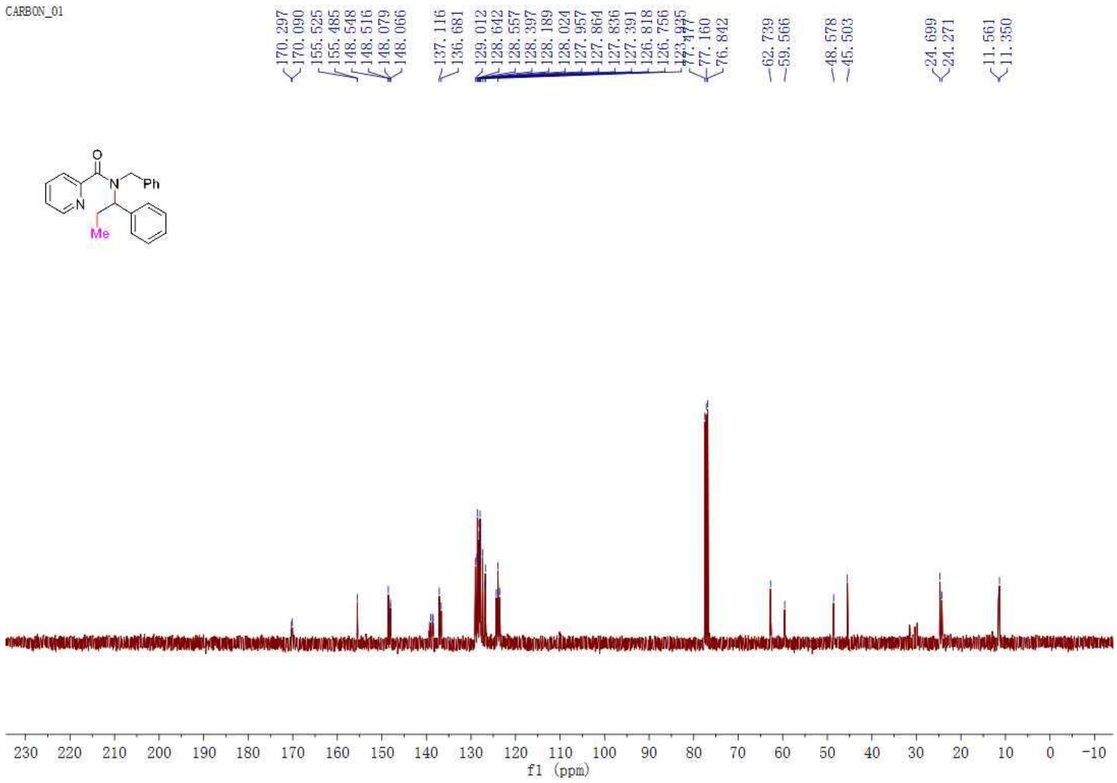

^{13}C NMR spectra for compound **7a** (100 MHz, $CDCl_3$)

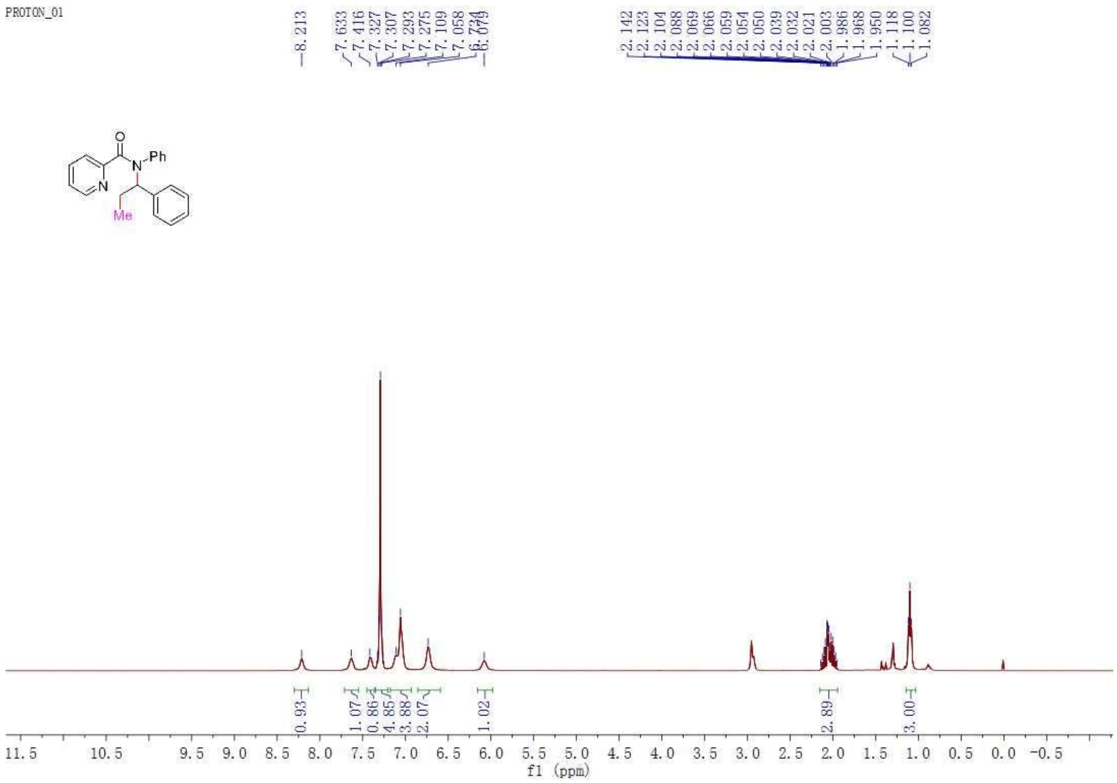

^{1}H NMR spectra for compound **7b** (400 MHz, Acetone-d_6)

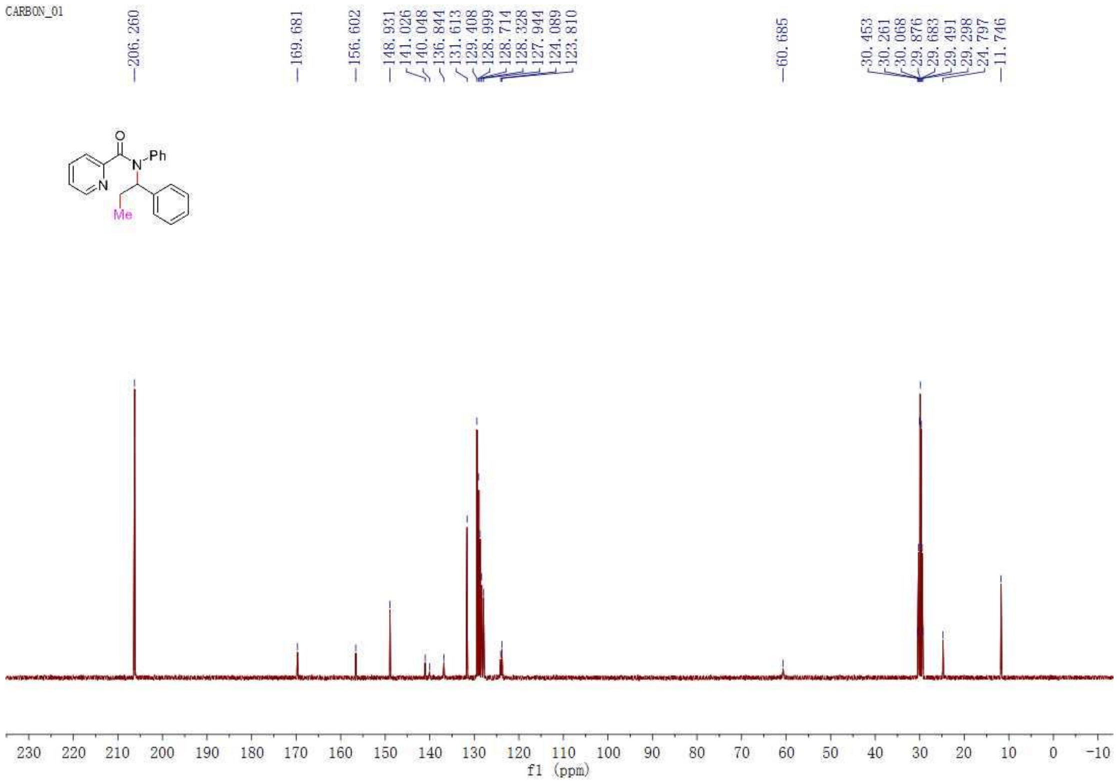

^{13}C NMR spectra for compound **7b** (100 MHz, Acetone-d_6)

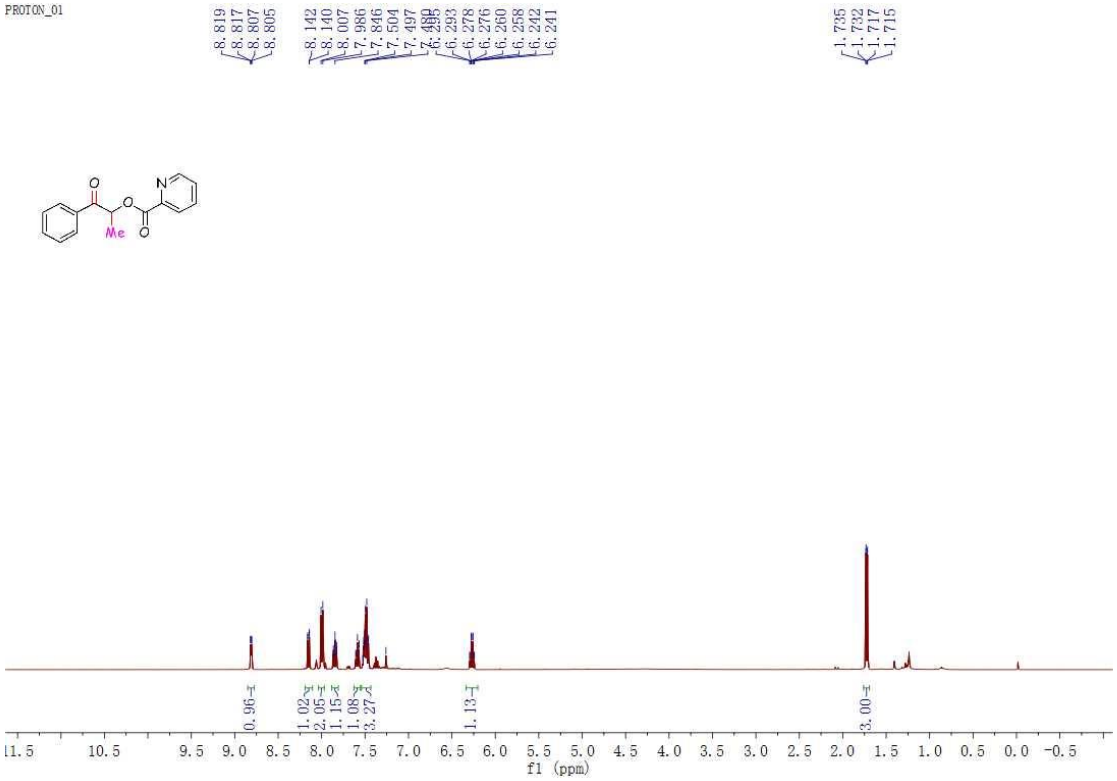

^{1}H NMR spectra for compound **7c** (400 MHz, $CDCl_3$)

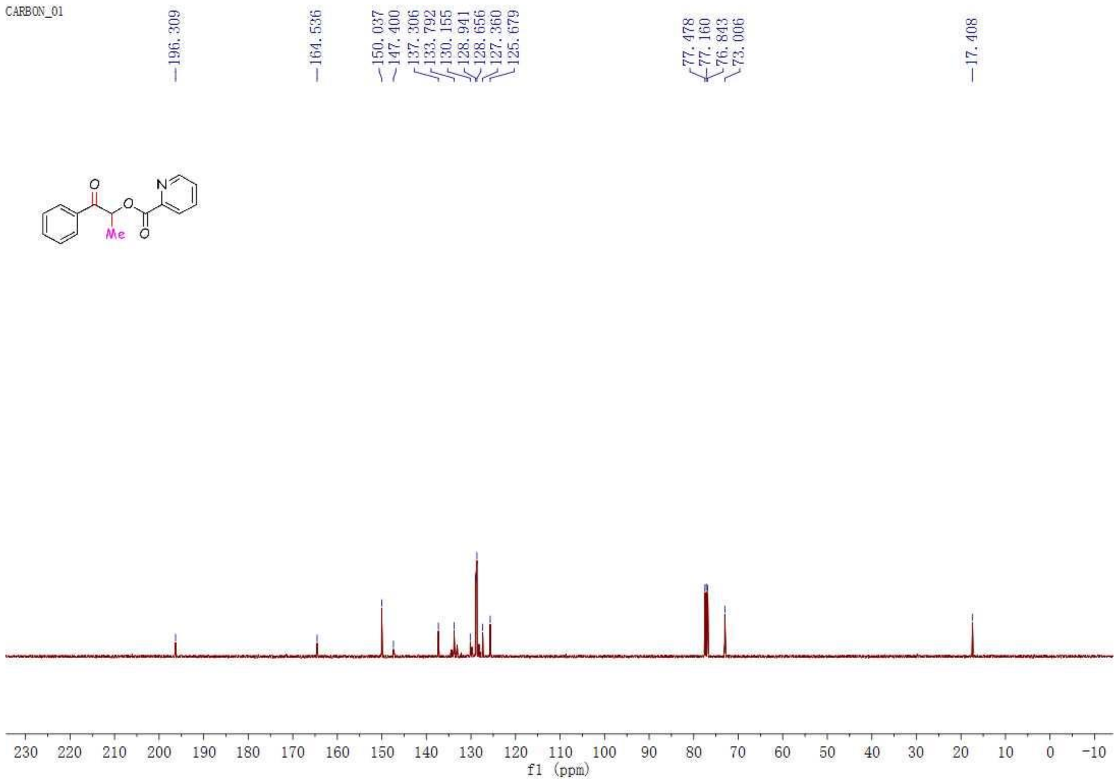

^{13}C NMR spectra for compound **7c** (100 MHz, $CDCl_3$)

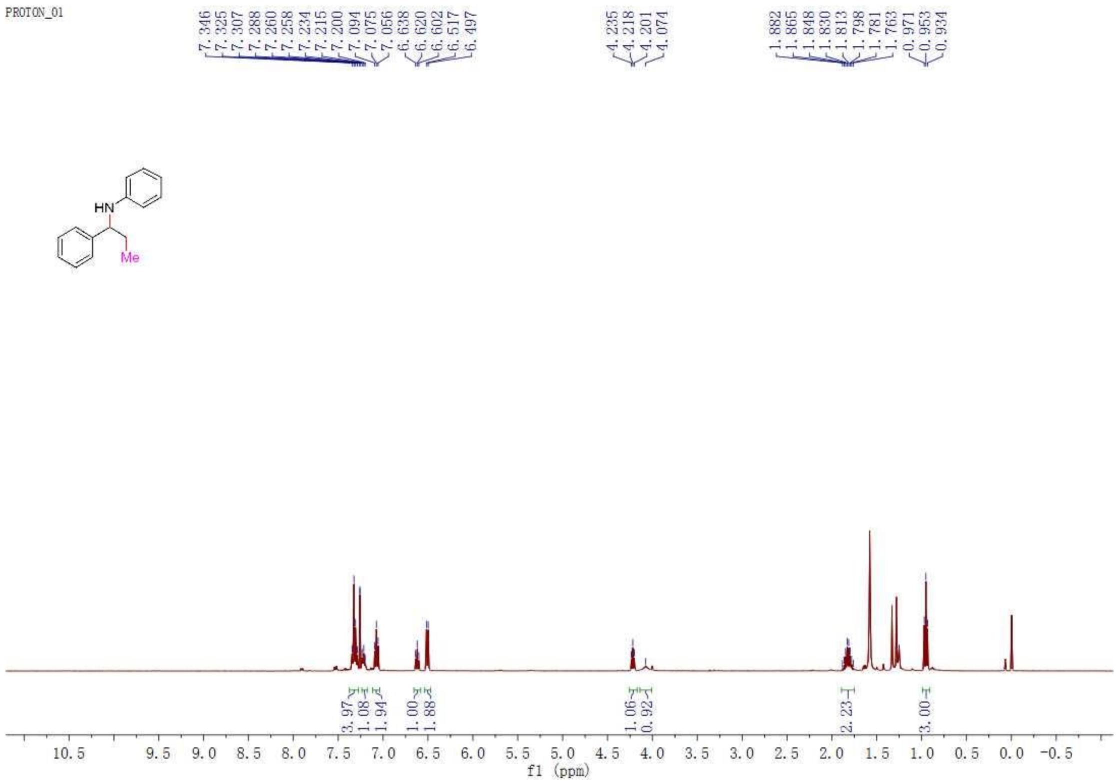

^{1}H NMR spectra for compound **7d** (400 MHz, $CDCl_3$)

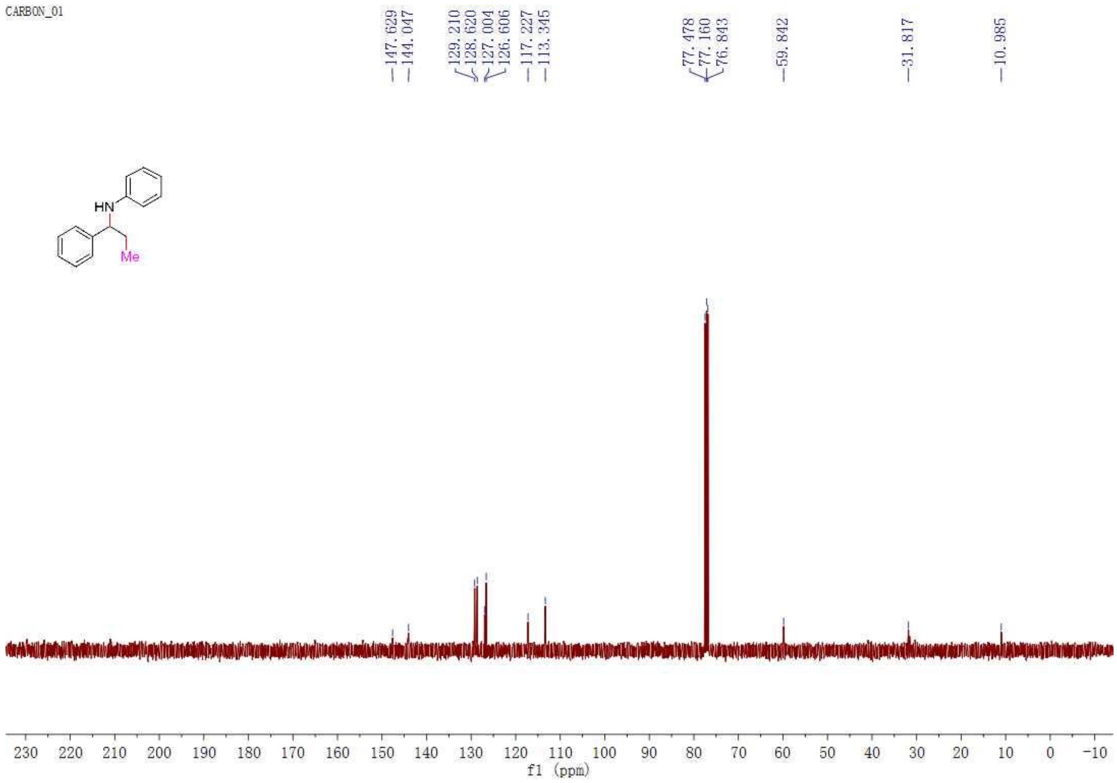

^{13}C NMR spectra for compound **7d** (100 MHz, $CDCl_3$)

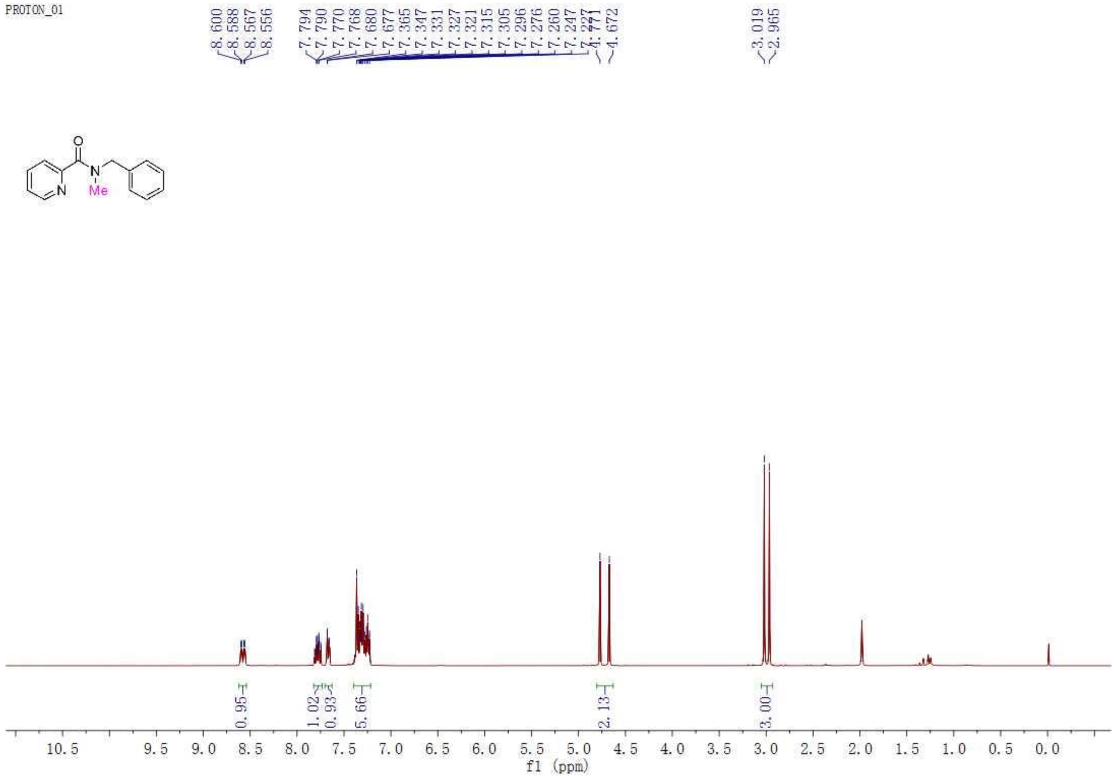

^{1}H NMR spectra for compound **3a-4** (400 MHz, $CDCl_3$)

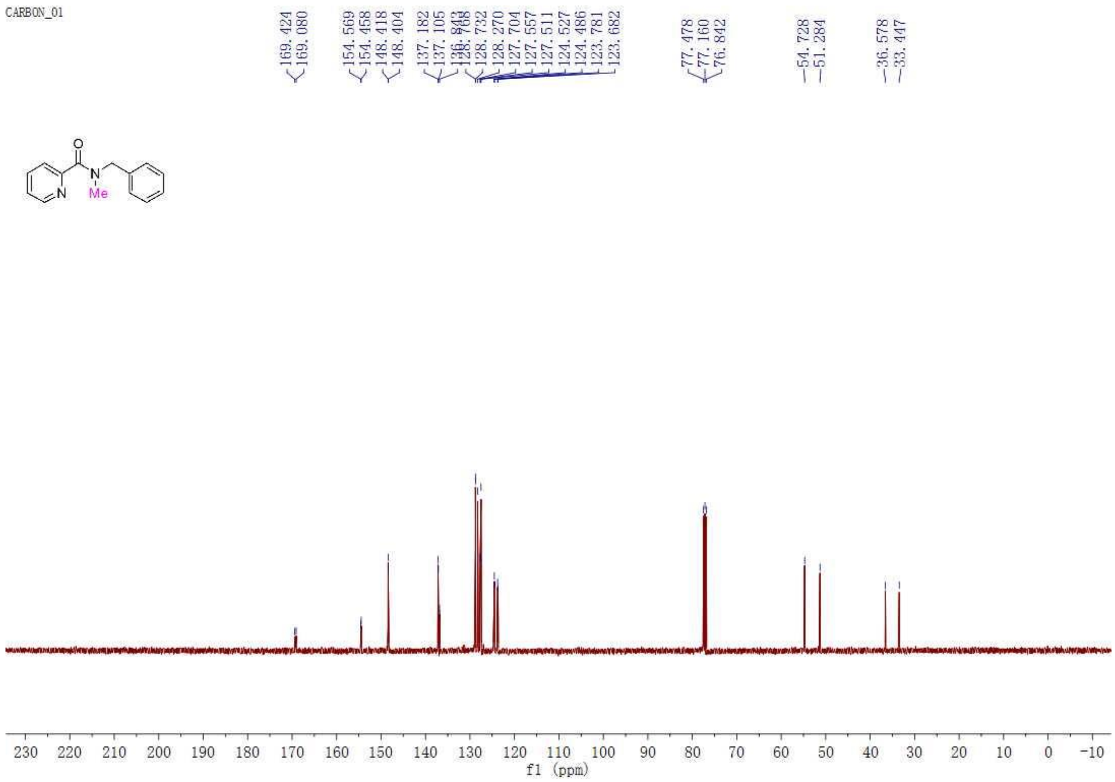

^{13}C NMR spectra for compound **3a-4** (100 MHz, $CDCl_3$)
